# Ciliary Neurotrophic Factor Induces Genes Associated with Inflammation and Gliosis in the Retina: A Gene Profiling Study of Flow-Sorted, Müller Cells

**DOI:** 10.1371/journal.pone.0020326

**Published:** 2011-05-26

**Authors:** Wei Xue, Radu I. Cojocaru, V. Joseph Dudley, Matthew Brooks, Anand Swaroop, Vijay P. Sarthy

**Affiliations:** 1 Department of Ophthalmology, Northwestern University Feinberg Medical School, Chicago, Illinois, United States of America; 2 Neurobiology, Neurodegeneration and Repair Laboratory, National Eye Institute, National Institutes of Health, Bethesda, Maryland, United States of America; University of Leuven, Rega Institute, Belgium

## Abstract

**Background:**

Ciliary neurotrophic factor (CNTF), a member of the interleukin-6 cytokine family, has been implicated in the development, differentiation and survival of retinal neurons. The mechanisms of CNTF action as well as its cellular targets in the retina are poorly understood. It has been postulated that some of the biological effects of CNTF are mediated through its action via retinal glial cells; however, molecular changes in retinal glia induced by CNTF have not been elucidated. We have, therefore, examined gene expression dynamics of purified Müller (glial) cells exposed to CNTF *in vivo*.

**Methodology/Principal Findings:**

Müller cells were flow-sorted from *mgfap-egfp* transgenic mice one or three days after intravitreal injection of CNTF. Microarray analysis using RNA from purified Müller cells showed differential expression of almost 1,000 transcripts with two- to seventeen-fold change in response to CNTF. A comparison of transcriptional profiles from Müller cells at one or three days after CNTF treatment showed an increase in the number of transcribed genes as well as a change in the expression pattern. Ingenuity Pathway Analysis showed that the differentially regulated genes belong to distinct functional types such as cytokines, growth factors, G-protein coupled receptors, transporters and ion channels. Interestingly, many genes induced by CNTF were also highly expressed in reactive Müller cells from mice with inherited or experimentally induced retinal degeneration. Further analysis of gene profiles revealed 20–30% overlap in the transcription pattern among Müller cells, astrocytes and the RPE.

**Conclusions/Significance:**

Our studies provide novel molecular insights into biological functions of Müller glial cells in mediating cytokine response. We suggest that CNTF remodels the gene expression profile of Müller cells leading to induction of networks associated with transcription, cell cycle regulation and inflammatory response. CNTF also appears to function as an inducer of gliosis in the retina.

## Introduction

Cytokines are secretory proteins that were initially characterized as immune modulators, but have been subsequently found to promote proliferation and differentiation in the nervous system [1]. The cytokine, ciliary neurotrophic factor (CNTF: NM_170786.2), belongs to the interleukin 6 (IL-6: NM_031168.1) family of cytokines that share one or more of the receptor subunit, glycoprotein 130 (gp130: NM_010560.3) [2], [3]. Activation by CNTF requires a heterotrimeric complex consisting of CNTF receptor α (CNTFRα: NM_001136056.2), leukemia inhibitory factor β (LIFRβ: NM_001113386.1) receptor and gp130 [2], [3]. CNTF acts on cells primarily by stimulating the Janus kinase-signal transducer and activator of transcription (JAK-STAT) signaling pathway [3]. Additionally, CNTF may stimulate cell survival, through MEK [extracellular signal-regulated kinase (ERK) kinase]/MAPK (mitogen activated protein kinase), Phosphoinositide 3-kinase (PI3-K)/Akt, and Nuclear factor kB (NF-kB) pathways [4]–[12].

CNTF promotes the survival of a variety of neurons and oligodendrocytes, and induces neurite outgrowth and axon regeneration in both developing and mature nervous system [13]–[18]. In addition, it appears to be an effective neuroprotective agent in animal models of CNS neurodegenerative diseases [19]. CNTF has also been reported to activate leptin-like pathways in the brain and reduce body fat and stress in a leptin-independent manner [20].

In the vertebrate retina, CNTF exhibits numerous effects on the development, differentiation and survival of retinal neurons [21]. CNTF appears to play a critical role in progenitor commitment to the rod photoreceptor cell fate and in photoreceptor differentiation [22]–[24]. It is reported to increase the long-term survival of retinal ganglion cells after axotomy [25], [26]. Furthermore, CNTF is capable of retarding retinal degeneration in several animal models of retinitis pigmentosa [27]–[36]. CNTF appears to be the most effective and mutation-independent, neuroprotective agent known. A recent phase I clinical trial demonstrated the safety of chronic CNTF delivery in patients with retinitis pigmentosa [37], and phase II trials have been completed for patients with retinitis pigmentosa (RP) and age-related macular degeneration (AMD).

Molecular mechanisms proposed to explain the neuroprotective role of CNTF in the retina include (i) direct action on photoreceptors to prevent their apoptosis (ii) stimulation of Müller (glial) cells to produce photoreceptor survival factors [38] (iii) enhanced synthesis or distribution of glutamate transporters, thereby improving glutamate handling, resulting in less excitotoxic damage to retinal neurons [39] and (iv) induction of metabolic plasticity and increased resistance to metabolic damage [40]. Nevertheless, these mechanisms remain to be evaluated.

A primary target of CNTF action in the retina is the Müller cell, a predominant glial cell that is responsible for maintaining the health and activity of retinal neurons [41], [42]. Müller cells contain CNTF receptors [19], and the JAK-STAT signaling pathway is rapidly activated in Müller cells in response to intravitreal CNTF injection [43]–[46]. Many of the biological effects of CNTF are proposed to be mediated through Müller cells [38]. Here, we have determined the global transcriptional response of Müller cells to CNTF *in vivo* with a goal to elucidate the molecular basis of its biological actions in the retina.

## Results

### Purification of Müller cells by flow-sorting

**Figure 1:**
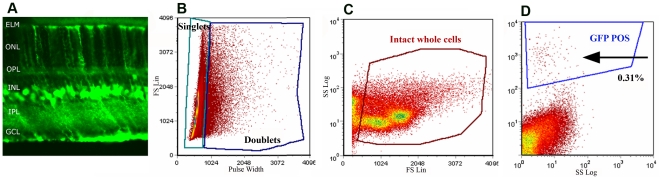
Flow-sorting Müller cells from mouse retina. (**a**) Transverse section of retina from mgfap-egfp transgenic mice. Note GFP expression in radially-oriented, Müller cell processes. (**b–c**) Scatter plot of flow-sorted, dissociated cells from mgfap-egfp transgenic mouse retina. The cells used for RNA preparation were collected from the enclosed area in F indicated by the arrow. Dissociated cells from non-transgenic mouse retina showed no GFP-fluorescence.

Müller (glial) cells constitute ∼2% of the cells in the mouse retina [47]. A major hurdle in studying CNTF action on Müller cells has been the lack of a reliable method to separate these cells away from retinal neurons. To circumvent this problem, we recently used Fluorescence Activated Cell Sorter (FACS) to purify Müller cells from *mgfap-egfp* (mouseGlial fibrillary acidic protein (NM_001131020.1)-enhanced green fluorescent protein) transgenic mice in which only Müller cells express GFP [48] (Fig. 1A). Retinas were dissociated into a single cell suspension following treatment with papain, and GFP^+^ (Müller) cells were enriched by FACS (Fig. 1B–D). During dissociation, Müller cells lose their radial processes and round up, but GFP remaining in the cell body is sufficient for flow sorting. We routinely obtained 30–50,000 viable cells per mouse retina. For microarray studies, we pooled retinas from 4–6 mice in each independent sample.

### Transcriptional analysis of Müller cells

**Figure 2:**
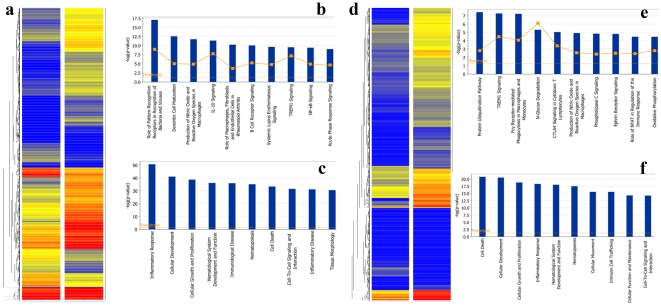
Microarray data analysis. (**a**) Hierarchical clustering showing 1261 probes that have a *P*-val ≤0.1 and a minimum 2-fold change between the CNTF and PBS samples at Day 1. Bright blue indicates lowest signal with increasing values indicated by yellow shading to bright red, representing peak signal. (**b**) Most significant 10 canonical pathways corresponding to Day 1. (**c**) Most significant biological functions for the same list of genes. (**d**) Hierarchical clustering showing 1541 probes that have a *P*-val ≤0.1 and a minimum 2-fold change between the CNTF and PBS samples at Day 3. (**e**) Most significant 10 canonical pathways corresponding to Day 3. (**f**) Most significant biological functions for the same list of genes.

To study the transcriptional response of Müller cells to CNTF, *mgfap-egfp* transgenic mice were intravitreally injected with CNTF or PBS (control). One day later, GFP^+^-Müller cells were flow-sorted, and total RNA was prepared for microarray analysis [49]. CNTF treatment resulted in differential expression of 923 transcripts that showed at least two-fold change (P-value <0.05). Of these, 691 transcripts exhibited 2-to 17-fold higher expression, whereas 232 transcripts revealed 2-to 5-fold reduction. We noticed, however, that genes such as *gfap* that are known to be induced by CNTF, were not detected. When the microarray data were analyzed with P-value<0.1, however, we found several genes of biological interest among 1261 differentially expressed transcripts. Of these, 939 transcripts were elevated 2- to 17-fold, and 322 transcripts were depressed 2- to 5-fold. The differentially expressed genes fell into several functional types (Table 1). A complete list of differentially regulated genes is presented in Table S1. A hierarchical cluster analysis also showed that genes with increased expression level outnumbered genes with decreased level, and that CNTF-induced genes fell into several clusters of co-expressed genes (Figure 2).

**Table 1:** List of differentially regulated genes in Müller cells one day after CNTF treatment.

Gene Symbol	Gene Name	Fold Change	Type
*Ccl6*	Chemokine ligand 6	13.5	Cytokine (19, 1)
*Ccl5*	Chemokine ligand 6	13.3	-
*Spp1*	Secreted phosphoprotein 1	10	-
*Tnf*	Tumor necrosis factor	7.8	-
*Il1rn*	Interleukin 1 receptor antagonist	7.5	-
*Av3*	Vav 3 guanine nucleotide exchange factor	-2.2	*-*
*Hmox1*	Heme oxygenase	15.9	Enzyme (115, 32)
*Iigp1*	Interferon inducible GTPase 1	8.8	-
*Ptgs2*	Prostaglandin-endoperoxide synthase 2	10.3	-
*Oasl*	2′-5′-oligoadenylate synthetase-like	6.9	-
*XDH*	Xanthine dehydrogenase	6.6	-
*Pafah1b1*	Platelet-activating factor acetylhydrolase 1b	-2.5	*-*
*Rab2b*	RAB2B, member RAS oncogene family	-2.5	*-*
*Rab3c*	RAB3C, member RAS oncogene family	-2.5	*-*
*Rims1*	Regulating synaptic membrane exocytosis 1	-2.6	*-*
*Scd2*	Stearoyl-Coenzyme A desaturase 2	-2.6	*-*
*Ccrl2*	Chemokine (C-C motif) receptor-like 2	12.5	G-protein (16, 9)
*Gpr109a*	G protein-coupled receptor 109A	9.4	-
*Ccr1*	Chemokine (C-C motif) receptor 1	8.9	-
*C5ar1*	Complement component 5a receptor 1	7.7	-
*Emr1*	Egf-like module, mucin-like chemokine-like recp 1	7.0	-
*Tm2d1*	TM2 domain containing 1	-2.0	*-*
*Xpr1*	Xenotropic and polytropic retrovirus receptor 1	-2.2	*-*
*Ptger3*	Prostaglandin E receptor 3 (subtype EP3)	-2.7	*-*
*Lphn3*	Latrophilin 3	-2.7	*-*
*Gpr158*	G protein-coupled receptor 158	-2.9	*-*
*Edn2*	Endothelin 2	6.7	Growth Factor (8, 2)
*Gmfg*	Glia maturation factor, gamma	5.3	-
*Nudt6*	Nudix-type motif 6	4.2	-
*Tgfb1*	Transforming growth factor, beta 1	3.8	-
*Gmfg*	Glia maturation factor, gamma	3.4	-
*Angpt1*	Angiopoietin 1	-2	*-*
*Fgf9*	Fibroblast growth factor 9 (glia-activating factor)	-2	*-*
*Kcnk6*	Potassium channel, subfamily K, member 6	4.5	Ion Channel (7, 11)
*P2rx7*	Purinergic receptor P2X, ligand-gated ion cha, 7	3.9	-
*Kctd12*	Potassium channel tetramerisation domain	3.2	-
*Kcnn4*	Potassium intermediate/small calcium-activated	2.7	-
*Hvcn1*	Hydrogen voltage-gated channel 1	2.4	-
*Kcnj6*	Potassium inwardly-rectifying channel	-2.3	*-*
*Kcnip1*	Kv channel interacting protein 1	-2.4	*-*
*Scn2b*	Sodium channel, voltage-gated, type II, beta	-2.4	*-*
*Kcnma1*	Potassium large conductance calcium-activated	-2.7	*-*
*Stx1b*	Syntaxin 1B	-3.1	*-*
*Vav1*	Vav 1 guanine nucleotide exchange factor	9.7	Transc. Regulator (78, 28)
*Egr2*	Early growth response 2	8.5	-
*Atf3*	Activating transcription factor 3	7.5	-
*Klf4*	Kruppel-like factor 4 (gut)	7.4	-
*Ifi16*	Interferon, gamma-inducible protein 16	7.3	-
*Ldb2*	LIM domain binding 2	-3.3	*-*
*Nfib*	Nuclear factor I/B	-3.3	*-*
*Nfia*	Nuclear factor I/A	-3.0	*-*
*Ccnt2*	Cyclin T2	-2.9	*-*
*Pcgf6*	Polycomb group ring finger 6	-2.5	*-*
*Cd14*	CD14 molecule	9.3	Transm. Receptor (50, 3)
*Il7r*	Interleukin 7 receptor	8.9	-
*Msr1*	Macrophage scavenger receptor 1	8.3	-
*Tlr2*	Toll-like receptor 2	8.0	-
*Cd69*	CD69 molecule	7.9	-
*Cd247*	CD247 molecule	-2.6	*-*
*Gpc6*	Glypican 6	-2.5	*-*
*Ifnar1*	Interferon (alpha, beta and omega) receptor 1	-2.1	*-*
*Slc11a1*	Solute carrier family 11	7.3	Transporters (33,15)
*Slc15a3*	Solute carrier family 15, member 3	5.6	-
*Slc3a3*	Solute carrier family 13	5.6	-
*Tap1*	Transporter 1, ATP-binding cassette, sub-family B	5.4	-
*Mcl1*	Myeloid cell leukemia sequence 1 (BCL2-related)	5.2	-
*Atp6v1b2*	ATPase, H+ transporting, lysosomal 56/58kDa	-2.7	*-*
*Sec62*	SEC62 homolog	-2.7	*-*
*Rph3a*	Rabphilin 3A homolog	-2.6	*-*
*Vps41*	Vacuolar protein sorting 41 homolog	-2.5	*-*
*Slc6a1*	Solute carrier family 6, member 1	-2.5	*-*

The table lists the top-five up- and down-regulated genes derived from IPA analysis. The total number of up- and down–regulated genes in each type are listed along with the functional types, in column 4.

### Validation of transcript level changes

**Figure 3:**
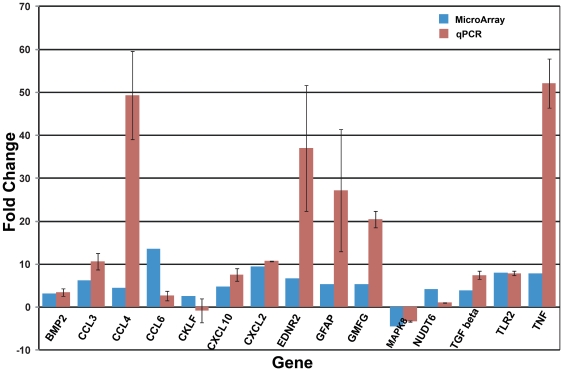
qRT-PCR validation of selected genes using independent biological samples. Predicted fold changes from microarray analysis and relative gene expression fold change from qRT-PCR for 3–6 independent biological replicates of flow-sorted Müller cells. Error bars indicate ±SEM.

Real time-PCR (qPCR) analysis of independent samples of FACS-purified Müller cells was carried out to validate the findings from the microarray data [23]. We performed primer design, one-step RT-PCR reaction, and qPCR analysis as previously described [49]. To determine the relative change in gene expression, we used the system software to compare the number of cycles (C_t_) needed to reach the midpoint of the linear phase. All observations were normalized to the housekeeping gene *Gapdh* (NM_008084.2). We observed that among the 15 transcripts (from different gene families) tested, 12 genes demonstrated higher transcript levels, consistent with the microarray data (Figure 3). Two transcripts (NUD6: NM_008006.2, CKLF: NM_029295.2) showed a negative trend in the qPCR assay (Figure 3), whereas one transcript (MAPK8: NM_016700.4) that exhibited reduced expression in the microarray data also showed a downward trend in the qPCR analysis. These results establish that transcript changes observed in the microarray data represent the gene expression changes in Müller cells.

### Pathways and biological processes associated with CNTF

Computational approaches were employed to identify biological pathways among the list of differentially expressed genes that respond to CNTF. We used the Ingenuity® Pathway Analysis (IPA) program (Ingenuity Systems, Red Wood City, CA), which sorts genes into canonical pathways based on the scientific literature and indicates significantly overrepresented pathways [www.ingenuity.com] (Figure 2). The top canonical pathways were concerned with the role of pattern recognition receptors, production of reactive oxygen species, IL10 signaling and role of macrophages in immune disease, consistent with the known roles of IL-6 family of cytokines (Table 2). The top biological functions were cellular development, cell death and cell- to -cell signaling (Fig. 2), which are related to immune response, immunological disease and inflammatory disease.

**Table 2:** Canonical Pathway analysis of CNTF-induced genes at Day 1 and Day 3.

Canonical Pathways- Day 1	CNTF–associated genes	p-value
Role of Pattern RecognitionReceptors of Bacteria andViruses	*Tlr1, Pik3r5, Oas1b, Ccl5, C1qb, Ifih1, C5ar1, Ticam1, Pik3cg, Creb1, Tlr7, Casp1, Oas1, C3, Nlrp3, Oas2, Nfkb2, Oas3 (includes EG:4940), Tlr2, Irf7, Clec7a, Syk, Ddx58, Tlr6, Pik3r6, Pik3cd, Elf2ak2, C3ar1, Tnf*	9.59E
Dendritic Cell Maturation	*B2m, Rac2, Il1a, Icam1, Nfkbie, Pik3r5, Hla-drb1, Hla-dmb, Fcgr2b, Fcgr1a, Col1a2, Nfkbia, Pik3cg, Creb1, Hla-b, Stat1, Tnfrsf1b, Fcgr3a, Hla-c, Tyrobp, Tnfrsf1a, Fcgr2a, Mapk8, Ikbke, Nfkb2, Tlr2, Col5a3, Il1rn, Pik3r6, Fcer1g, Cd86, Pik3cd, Irf8, Tnf, Ifnar1*	3.06E
Production of Nitric Oxide and Reactive Oxygen Species in Macrophages	*Rap1b, Rac2, Nfkbie, Pik3r5, Rhoh, Ppp1r14b, Spii1, Map3k10, Rhog, Nfkbia, Pik3cg, Cyba, Ppm1l, Cybb, Stat1, Tnfrsf1b, Map3k2, Ptpn6, Tnfrsf1A, Mapk8, Ikbke, Nfkb2, Ncf4, Irf1, Tlr2, Prkcd, Plcg2, Ncf2, Pik3r6, Map3k8, Pik3cd, Irf8, Jak3, Tnf*	1.93E
IL-10 Signaling	*Ccr1, Il18rap, Socs3, Ccr5, Il4r, Il1a, Il1rl1, Fcgr2a, Nfkbie, Mapk8, Ikbke, Nfkb2, Stat3, Fcgr2b, Il1r2, Hmox1, Nfkb, Il1rn, Il10ra, Il10rb, Cd14, Tnf*	4.67E
Role of Macrophages, Fibroblasts and Endothelial Cells in Rheumatiod Arthritis	*Il18rap, Rac2, Socs3, Tlr1, Fcgr1a, Il1r2, Tgfb1, Pik3cg, Osm, Wnt5b, Tnfsf13b, C1s, Il6r, Stat3, Apc, Tlr2, Il1rn, Cebpd, Plcg2, Prkcd, Pik3r6, Pik3cd, Tnf, Il1a, Fn1, Icam1, Il1rl1, Nfkbie, Pik3r5, Ccl5, Il17ra, C1r, Nfkbia, C5ar1, Ppp3cb, Creb1, Tlr7, Cebpa, Tnfrsf1b, Fcgr3a, Traf1, Tnfrsf1a, Ikbke, Cebpb, Irak3, Ripk1, Csf1, Tlr6, Tlr13*	6.21E

Canonical Pathways- Day 3	CNTF–associated genes	p-value
Protein Ubiquitination Pathway	*Usp14, Psmd7, Psma7, Ube2l3, Ube2v2, Dnajc10, Psmb8, Hspa5, Psmc5, Usp48, Uchl1, Uso1, Dnajc28, Hsp90b1, Hsp90ab1, Psmd10, Uchl5, Psmd14, Psma2, Psmc2, Birc3, Hspa4l, Hla-c, Psmb4, Psmb9, Psma6, Hsph1, Usp9x, Psmd6, Psma1, Dnajb9, Psmd8, Psmb7, Psmc1, Cul2, Psmb2, Psmd12, Hspa13, Psmb1, Dnajc5b, Smurf2, Uba1, Usp25, Dnajc7, Tceb1*	3.8E-08
TREM 1 Signaling	*Itgb1, Rac2, Tyrobp, Tlr8 (includes EG:51311), Cd83, Jak2, Stat3, Fcgr2b, Tlr4, Ccl2, Plcg2, Tlr6, Casp1, Tlr7, Cd86, Tlr13, Il1b, Itgax*	5.48E-08
Fcg Receptor-mediated Phagocytosis in Macrophages and Monocytes	*Actr2, Rac2, Gab2, Fcgr2a, Actb, Arpc5, Fcgr1a, Inpp5d, Pld4, Myo5a, Hmox1, Rab11b, Pik3cg, Prkcd, Syk, Arpc4, Vamp3, Lyn, Rab11a, Arpc3, Vav1, Fcgr3a, Lcp2, Prkcb*	6.07E-08
N-Glycan Degradation	*Chi3l4, Fuca2, Edem1, Gm2a, Man1a1, Hexb, Lct, Hexa, Man2b1, Man2a1, Chi3l3*	4.99E-06
CTLA4 Signaling in Cytotoxic T Lymphocyte	*Hla-dma, Ap2b1, Rac2, Ptpn6, Ppp2r2a, Ap1s2, Cltc, Hla-dqa11, Hla-drb1, Jak2, Hla-dqb1, Ppp2r1a, Syk, Pik3cg, Clta, Fcer1g, Cd86, Pik3cb, Lcp2, Ap1g1*	9.22E-06

The top-five pathways and associated genes are presented.

### Genes induced by CNTF

Cytokines represented one of the highly induced sets of genes in Müller cells after exposure to CNTF (Table 1,S1). Furthermore, many cytokines types including interleukins, chemokines and chemokine-like factors, tumor necrosis factor and colony stimulating factor were elevated 2 to 14-fold. Among chemokines, both C-C and CXC family members were detected. Intriguingly, multiple proinflammatory chemokines were induced. Several enzymes involved in intermediary, nucleic acid and lipid metabolism were also elevated. Heme oxygenase-1 (NM_010442.2) was induced at a high level, pointing to a role for CNTF in neuroprotection against oxidative damage. Interestingly, transcript levels for several chemokine receptors, CCRL2 (NM_017466.4), CCR1 (NM_009912.4), CXCR4 (NM_009911.3) and CCR5 (NM_009917.5) were increased in CNTF-treated Müller cells. The induction of chemokines and chemokine receptors suggests existence of paracrine loops that could further modify CNTF-induced gene expression pattern.

The well-known growth factor and anti-inflammatory agent, transforming growth factor ß (TGFß: NM_011577.1) was induced by CNTF. Expression of both TGFß and proinflammatory chemokines suggests that CNTF has complex effects on Müller cells. Surprisingly, endothelin 2 (NM_007902.2), a signaling molecule released by degenerating photoreceptors that initiates Müller cell gliosis [50], was strongly induced by CNTF; however, no change in endothelin receptor B (*endrB*: NM_001136061.1) expression was detected.

There were substantial changes in the expression of ion channels including the potassium channels, KCNK6 (NM_001033525.3) and KCNN4 (NM_001163510.1). We did not, however, detect transcript level changes for two potassium channels, Kir4.1 (NM_001039484.1) and Kir2.1 (NM_008425.4), which are known to be involved in potassium ion-homeostasis in Müller cells [42]. As CNTF treatment induces a large number of genes, it was not surprising that several transcriptional regulators such as CCAAT/enhancer binding proteins, early growth response proteins, and forkhead box proteins were elevated. In comparison, far fewer translational regulators were altered.

We also identified higher expression of several transmembrane receptors including cell adhesion molecules such as CD14 (NM_009841.3) and CD86 (NM_019388.3) although there was no change in CD44 (NM_009851.2), a hyaluronic acid receptor, expressed in Müller cells [41]. In addition, a large number of interleukin receptors were induced, suggesting the existence of feed back loops in CNTF-induced networks. Expression of Toll-like receptors 1 and 2 (TLR1: NM_030682.1 and TLR2: NM_011905.3) was increased following CNTF treatment (Table S1, Figure 3), suggesting that Müller cells are part of the innate immune response in the retina. One of the roles of Müller cells involves transport and export of nutrients and related small molecules in the retina [41], [4]. Induction of genes for a variety of transporters belonging to ABC and SLC superfamilies is in accord with the metabolic functions of Müller cells [41]. Interestingly, SLC11A1 (NM_013612.2) has been proposed as an autoimmunity susceptibility gene.

In general, only a few genes showed a decrease in transcript levels (Table 1, S1), with most showing a modest change of about two-fold.

### Long-time effects of CNTF

Cytokines are known for their rapid and transient action [1]. To examine whether transcripts induced initially by CNTF continue to be expressed or the gene expression pattern is further altered, we investigated transcriptional changes in Müller cells three days following CNTF treatment. The biological activity of CNTF appears to last 2–3 days, due perhaps both to degradation of the cytokine [43] and activation of suppressor of cytokine signaling systems [51]. We found that 1192 transcripts were increased 2- to 26-fold, and 349 transcripts were reduced 2- to 10-fold. (Table 3; see Table S2 for a complete list). A comparison of transcriptome data from Müller cells at one and three days after CNTF exposure showed that (i) there is an increase in the number of genes transcribed from 1261 to 1541; and (ii) the pattern of transcribed genes is changed, only 273 overlapping transcripts were detected at these two time-points.

**Table 3:** List of differentially regulated genes in Müller cells three days after CNTF treatment.

Gene Symbol	Gene Name	Fold Change	Type
*Spp1*	Secreted phosphoprotein 1	15.8	Cytokines (16, 4)
*Ccl3l3*	Chemokine (C-C motif) ligand 3-like 3	14.8	-
*Pf4*	Platelet factor 4	12.4	-
*Nampt*	Nicotinamide phosphoribosyltransferase	4.0	-
*Ccl13*	Chemokine (C-C motif) ligand 13	3.9	-
*Thpo*	Thrombopoietin	-2.0	*-*
*Prl*	Prolactin	-2.1	*-*
*Ifnk*	Interferon, kappa	-2.1	*-*
*Il1f8*	Interleukin 1 family, member 8 (eta)	-2.3	*-*
*Cybb*	Cytochrome b-245, beta polypeptide	14.9	Enzymes (271, 28)
*Gpnmb*	Glycoprotein (transmembrane) nmb	14.0	-
*Gatm*	Glycine amidinotransferase	11.2	-
*Ppib*	Peptidylprolyl isomerase B (cyclophilin B)	10.0	-
*Hpgds*	Hematopoietic prostaglandin D synthase	10.0	-
*Fut1*	Fucosyltransferase 1	-2.5	*-*
*Bche*	Butyrylcholinesterase	-2.5	*-*
*B3gat2*	Beta-1,3-glucuronyltransferase 2	-2.6	*-*
*Gng13*	Guanine nucleotide binding protein, gamma 13	-3.6	*-*
*Gcsh*	Glycine cleavage system protein H	-4.7	*-*
*C3ar1*	Complement component 3a receptor 1	20.4	G-proteins (14, 82)
*Emr1*	Egf-like module containing, mucin-like	9.2	-
*Cxcr4*	Chemokine (C-X-C motif) receptor 4	8.1	-
*Ccr5*	Chemokine (C-C motif) receptor 5	6.5	-
*Gpr65*	G protein-coupled receptor 65	5.8	-
*Olfr703*	Olfactory receptor 703	-3.4	*-*
*Olfr619*	Olfactory receptor 619	-3.5	*-*
*Olfr921*	Olfactory receptor 921	-3.9	*-*
*Olfr777*	Olfactory receptor 777	-4.2	*-*
*Olfr153*	Olfactory receptor 153	-4.8	*-*
*Grn*	Granulin	5.2	Growth Factors (3, 0)
*Gmfb*	Glia maturation factor, beta	3.2	-
*Hgf*	Hepatocyte growth factor (hepapoietin A)	2.8	-
*Clic1*	Chloride intracellular channel 1	2.7	Ion Channels (6, 0)
*Clic4*	Chloride intracellular channel 4	2.6	-
*Fxyd5*	FXYD domain containing ion transport regul 5	2.6	-
*P2rx4*	Purinergic receptor P2X, ligand-gated ion chan, 4	2.2	-
*P2rx7*	Purinergic receptor P2X, ligand-gated ion chan, 7	2.0	-
*Taf9*	TAF9 RNA polymerase II, TATA box binding protein	8.1	Transc. Reg. (80, 20)
*Sp100*	Protein SP100 nuclear antigen	7.3	-
*Tcea1*	Transcription elongation factor A (SII), 1	7.1	-
*Supt4h1*	Suppressor of Ty 4 homolog 1 (S. cerevisiae)	5.7	-
*Rel*	V-rel reticuloendotheliosis viral oncogene homolog	5.6	-
*Phb*	Prohibitin	-2.3	*-*
*Gli3*	GLI family zinc finger 3	-2.3	*-*
*Erg*	V-ets erythroblastosis virus E26 oncogene homolog	-2.4	*-*
*Abra*	Actin-binding Rho activating protein	-2.4	*-*
*Ankz1*	Ankyrin repeat and zinc finger domain	-2.5	*-*
*Hla-c*	Major histocompatibility complex, class I, C	17.0	Transm. Receptors (48, 1)
*Msr1*	Macrophage scavenger receptor 1	12.9	-
*Clec7a*	C-type lectin domain family 7, member A	10.2	-
*Fcrl2*	Fc receptor-like 2	7.1	-
*Tlr7*	Toll-like receptor 7	6.9	-
*Gfra2*	GDNF family receptor alpha 2	-2.0	*-*
*Saa1*	Serum amyloid A1	15.4	Transporters (101, 11)
*M6pr*	Mannose-6-phosphate receptor (cation dependent)	6.3	-
*Gabarap*	GABA(A) receptor-associated protein	5.9	-
*Mfsd1*	Major facilitator superfamily domain containing 1	5.7	-
*Atp6v1e1*	ATPase, H+ transporting, lysosomal 31kDa, V1, E1	5.4	-
*Timm9*	Translocase of inner mitochondrial membrane 9	-2.3	*-*
*Atp6ap2*	ATPase, H+ transporting, lysosomal protein 2	2.3	*-*
*Igfbp7*	Insulin-like growth factor binding protein 7	-2.5	*-*
*Apod*	Apolipoprotein D	-2.9	*-*
*Disp1*	Dispatched homolog	-3.9	*-*

The table lists the top-five up- and down-regulated genes derived from IPA analysis. The total number of up- and down –regulated genes in each type is listed along with the functional types, in column 4.

**Figure 4:**
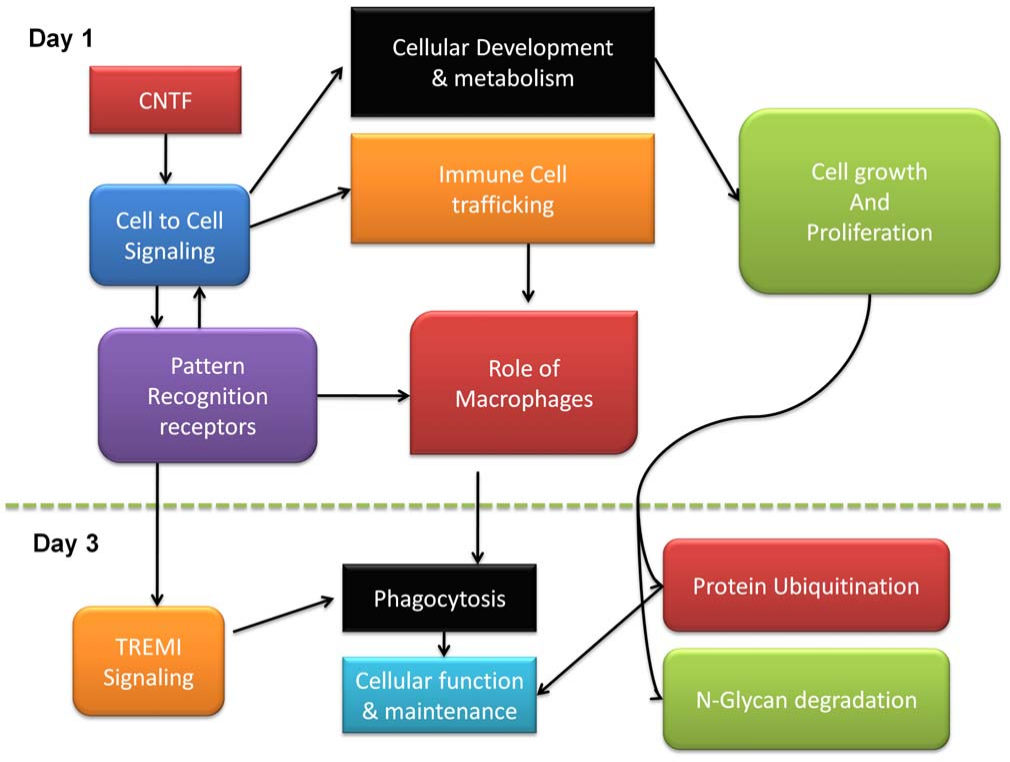
Changes in pathways and processes induced by CNTF. The diagram indicates potential biological processes and their inter-relationships in Müller cells at day 1 and day 3 after CNTF treatment. The processes and pathways were generated from IPA analysis.

IPA analysis was used to categorize genes according to canonical pathways and functions (Table 2). At day 3, the networks involved in CNTF signaling had changed and new canonical pathways and functional groups were operative (Figure 4). The top canonical pathways were now associated with protein ubiquitination, TREM1 signaling and receptor mediated phagocytosis. The molecular and cellular functions involved cell death, and cell growth and proliferation (Table 2). These network changes are likely to represent the altered role of Müller cells in CNTF signaling in the retina.

cThe differentially regulated transcripts could be classified into distinct functional types using IPA (Table 3). At three days, we found the induction of chemokine, CCl2 (NM_011333.3), which is known to be involved in the activation and chemoattraction of circulating macrophages into tissues [52]. Relatively few growth factors were induced at day three unlike at day one. We also observed a high induction of the glycoprotein, GPNMB (NM_053110.4), a melanosome-associated protein implicated in cancer metastasis [53]. The chemokine receptor, CCR5, continued to be expressed at 3 days. In support of the continuing role of Müller cells in innate immunity, we found a 14-fold increase in TLR13 (NM_205820.1) and 7-fold increase in TLR7 (NM_133211.3) levels in Müller cells.

### Networks induced by CNTF

**Figure 5:**
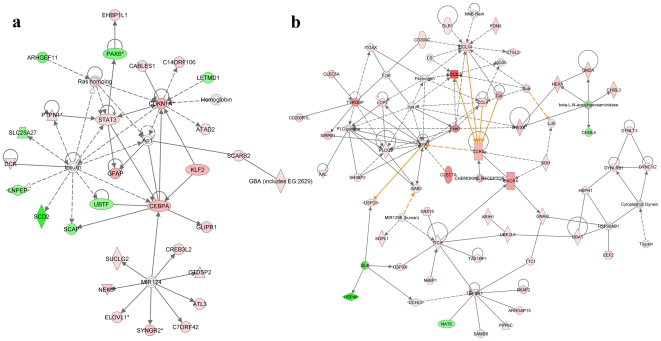
The most prominently affected gene networks generated by Ingenuity Pathway Analysis. (**a**) Day 1- Genes in this network are responsible for cellular development, connective tissue disorder and metabolic disease. (**b**) Day 3- Genes in this network are responsible for cellular function and maintenance, molecular transport and inflammatory response. Red color indicates induction, while green represents repression; color intensity correlates with fold change.

Next, we examined network(s) that contained genes relevant to CNTF action. Several networks were generated using Metacore (www.genego.com/metacore.php), and genes associated with one of the networks together with their direct and indirect interactions are presented in Fig. 5a. The following gene products emerged as hubs (at day one): STAT3 (NM_213659.2), a transcription factor involved in cytokine signaling; CDKN1A (NM_007669.4), cyclin-dependent kinase inhibitor 1, which inhibits cyclin-CDK2 (NM_016756.4) or CDK4 (NM_009870.3) and acts as a regulator of cell cycle; CEBPA (NM_007678.3), a bZIP transcription factor involved in cytokine (leptin) signaling; Insulin, a well known hormone associated with carbohydrate and fat metabolism; AP-1, a transcription factor stimulated by cytokines and growth factors; Ras homolog, which is involved in cell contraction and cytoskeleton rearrangement; and MIR124, a microRNA involved in brain-specific pre-mRNA splicing. Overall, genes associated with transcription, cell cycle regulation and inflammatory response appear to occupy prominent positions (hubs) in the network topology.

In accord with the changes in gene expression pattern, there were substantial changes in the CNTF-induced networks three days after CNTF treatment (Fig. 5b). The following gene products were found as hubs: CCL3 (NM_011337.2) and CCL4 (NM_013652.2), chemokines involved in the activation and recruitment of polymorphonuclear leukocytes; CCL13, a chemoattractant induced by IL1b (NM_008361.3) and TNF-a (NM_013693.2), and a ligand for CCR5 (NM_009917.5); CCR5 and CXCR4 (NM_009911.3), chemokine receptors that act as co-receptors for HIV entry into cells; TyroBP (NM_011662.2), a transmembrane signaling receptor with a role in inflammation; PLCG2 (NM_172285.1) and PLC gamma (NM_021280.3), phospholipases associated with cell signaling; SYK (NM_001198977.1), a non-receptor tyrosine kinase involved in B cell signaling; and TGFBR1 (NM_009370.2), transforming growth ß receptor 1.

### CNTF is an inducer of gliosis in Müller cells

CNTF can also act as an inducer of reactive gliosis in the CNS [41], [54]. Intravitreal injection of CNTF in rodents rapidly induces GFAP, a hallmark of gliosis in the nervous system [43], [46]. Virtually every retinal disease is associated with ‘activation’ of Müller cells and reactive gliosis [41], [42]; it is possible that CNTF might be one of the endogenous inducers. Moreover, CNTF is upregulated in response to retinal degeneration and injury [55], [56].

To further establish CNTF as a candidate inducer of gliosis in the retina, we compared transcripts with increased expression in CNTF-stimulated Müller cells with genes expressed in ‘reactive’ Müller cells. Rattner and Nathans [50] previously reported retinal transcriptional profile changes in two mouse models of retinal degeneration, the procadherin-knockout (proCAD) strain and mice with light damage [50]. Using *in situ* hybridization to localize some of the upregulated transcripts, they found that many of the upregulated genes were localized to Müller cells. A comparison of our microarray data with those of Rattner and Nathans [50] is presented in Table 4. All ten genes upregulated in light-damaged retina and six genes upregulated in proCAD mutant retina also showed increases in transcript levels, although the fold-changes differed. For example, ceruloplasmin (NM_001042611.1) increased ∼ 8-fold in light damaged retinas compared to 2-fold in CNTF-treated retina. These data show that CNTF-activated Müller cells express several genes, some of which are also induced in reactive Müller cells from proCAD mice or mice with light damage; thus, suggesting that CNTF is a likely to be an inducer of gliosis in the retina.

**Table 4:** Comparison of transcript level changes in retinas from procadherin-knockout (proCAD) and light damaged mice with Müller cells from CNTF-treated mouse retinas.

		Fold Change		
Gene name	Gene symbol	proCADmutant	Lightdamage	CNTF-treated
CCAT enhancer binding protein	*Cebpd*	5.7	19.3	4.26
Ceruloplasmin	*Cp*	0	8.2	2.43
Endothelin 2	*Edn2*	11.4	12.9	6.71
GFAP	*Gfap*	5	3.8	5.41
Lipocalin 2	*Lcn2*	0	17.9	2.63
Oncostatin M receptor	*Osmr*	3.4	8.2	3.5
S100 protein	*S100a6*	0	5.5	3.51
Serpin a3n	*Serpin a3n*	2.1	5.7	5.02
SOCS-3	*Socs3*	2.9	13.5	3.6
Soluble galactose binding protein	*Lgals3*	0	5.9	6.04

The pro-CAD and light damage data are from ref. 50.

### Comparison of gene profile of Müller cells with astrocytes and RPE

**Figure 6:**
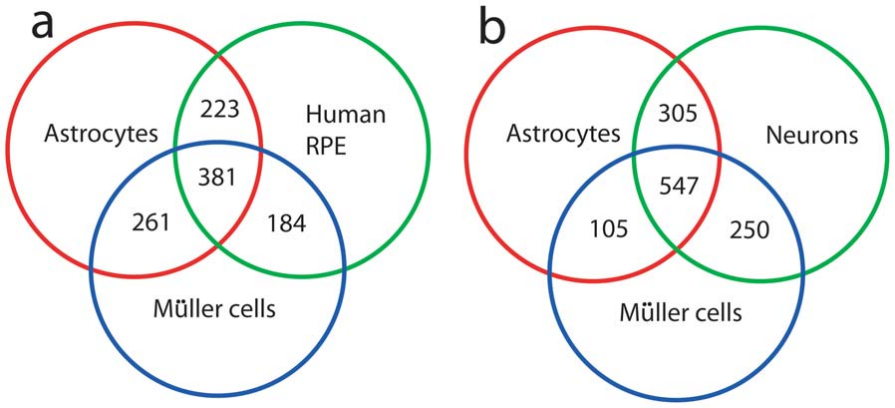
Overlapping genes in transcription profiles from Müller cells, astrocytes and retinal pigment epithelium (RPE). Intensity signals for the top 2000 probes were used to generate sets of overlapping genes. The astrocyte and RPE transcriptome data are derived from published studies (56,57). Müller cell data represent signals from cells not exposed to CNTF. (**a**) The diagram shows that 381 transcripts were common to the three cell types, while 642 were shared between Müller cells and astrocytes, 565 between Müller cells and the RPE, and 604 between astrocytes and RPE. (**b**) Diagram showing overlap of transcripts among astrocytes, Muller cells and neurons. The neuron transcriptome data was obtained from a published study (56).

Müller (glial) cells perform metabolic functions similar to astrocytes and RPE [41], [57]. To determine whether this similarity is also reflected in the gene expression profiles of these cell types, we compared the top 2000 highly expressed transcripts from Müller cells and astrocytes (Table S1), using previously published astrocyte transcriptome data from postnatal day-17 mice [58]. As shown in Figure 6, 642 genes were common to Müller cells and astrocytes yielding a 32% overlap in transcriptional pattern. In comparison, Müller cells and retinal pigment epithelial cells (RPE) have 565 genes in common. The RPE transcriptome data were obtained from a recent publication [59]. There is a 14% overlap (381 genes) in highly expressed genes among the three different cell types (Fig. 6a). When compared with transcriptional profiles of neurons, there was an overlap of 27% (Fig. 6b, 58).

**Figure 7:**
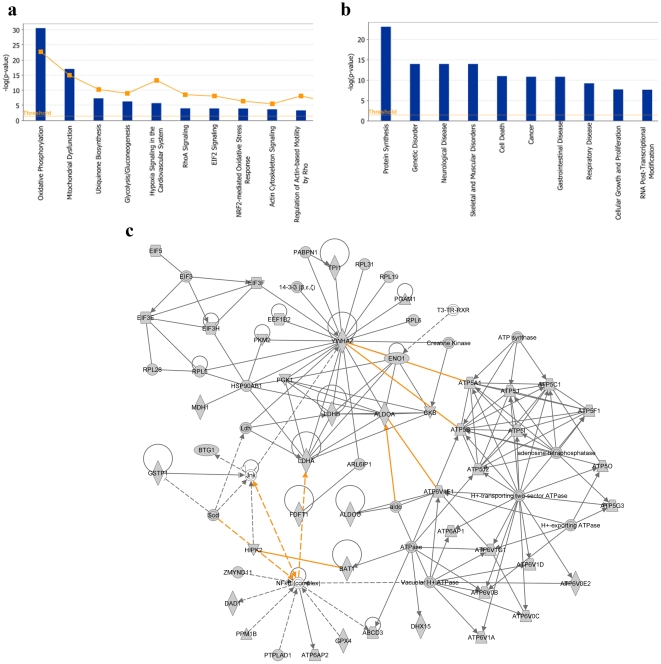
Gene networks common to Müller cells, astrocytes and RPE. (**a**) Most significant canonical pathways; (**b**) Most significant biological functions for the same list of genes; and (**c**) Genes in this network are responsible mitochondrial function and metabolism.

IPA of the 381 genes (common to Müller cells, astrocytes and RPE) showed that the top canonical pathways were concerned with oxidative phosphorylation, mitochondrial function, glycolysis/gluconeogenesis, hypoxia signaling and ubiquinone biosynthesis. This finding is in accord with the known supportive roles of the three cell types in energy metabolism in the retina [41], [57]. The top biological functions involve protein synthesis, RNA post-transcriptional modification, and energy production (Fig. 7).

Network analysis of the genes as well as their direct and indirect interactions (Fig. 7) revealed the following gene products emerged as hubs: ATP5I (NM_007507.2), mitochondrial ATP synthase; H^+^-transporting, two-sector ATPase; vacuolar H^+^-ATPase; LDHA (NM_001136069.2), lactate dehydrogenase; YWHAZ (NM_011740.3), an adapter protein implicated in several signal transduction pathways; and NFkB complex, which controls transcription and is involved in cellular response to cytokines. Overall, genes associated with carbohydrate metabolism, energy production, protein synthesis and nucleic acid metabolism appear to occupy prominent positions (hubs) in the network topology.

## Discussion

We report that CNTF induces rapid and extensive changes in the transcriptional profile of Müller (glial) cells *in vivo*. The activated genes involve growth factors, cytokines, ion channels, kinases, phosphatases, and G-protein coupled receptors that are components of cellular pathways concerned with cell- to -cell signaling, cell cycle and inflammation. The widespread genomic response of Müller cells is likely the basis for the numerous effects of CNTF on the development, differentiation and survival of retinal neurons.

Genes induced by CNTF are reported to carry a CNTF-response element, 5′-TTCC (N _3-5_) AA-3′ (CNTF-RE) in their 5′-flanking sequence [60]. We observed that a majority of upregulated genes had one or more (up to 6) CNTF-RE sites in their 5′-flanking sequence (∼1 kb). For example, among genes that are upregulated at least 6-fold (n = 51), 45 had the CNTF-RE on the direct or coding strand, one gene (Tnfaip2: NM_009396.2) did not have CNTF-RE site and four genes had a CNTF-RE site on the non-coding strand. Genes that carry the CNTF-RE are likely to be activated by the direct action of CNTF while genes without CNTF-RE element could be induced by secondary mechanisms.

### CNTF-mediated photoreceptor neuroprotection

CNTF acts as a neuroprotective agent in many animal models of retinal degeneration [27]–[36], [61]. One hypothesis postulates that CNTF acts directly on photoreceptors to promote their survival. The absence of CNTF receptors on rodent photoreceptors and the lack of STAT3 phosphorylation in photoreceptors following CNTF treatment have been advanced as evidence against this suggestion [38]. Recent studies with LIF (NM_001039537.1), a close relative of CNTF, show that this cytokine might directly act on photoreceptors [62]. In transgenic mice with conditional ablation of the gp130 gene in photoreceptors, LIF was unable to rescue light-induced photoreceptor degeneration whereas LIF was able to promote photoreceptor survival in mice with conditional inactivation of gp130 gene in Müller cells [62]. It could be argued that CNTF might act in a manner analogous to LIF.

A second hypothesis, termed the Müller cell hypothesis of neuroprotection, states that the CNTF effect on photoreceptors is indirect, and that CNTF mediates its neuroprotective effect by stimulating Müller cells [40]–[42]. Many lines of evidence support this hypothesis. CNTF receptors are found on Müller cells, and CNTF treatment rapidly stimulates the JAK-STAT pathway in Müller cells [43]–[46], [63]. Our experimental data clearly show that several growth factors and cytokines are induced by CNTF in Müller cells. However, none of the previously-tested, photoreceptor neuroprotective agents— Basic Fibroblast growth factor, bFGF (NM_008006.2); Brain-derived neurotrophic factor, BDNF (NM_001048139.1); Glia-derived neurotrophic factor, GDNF (NM_010275.2); Pigment epithelium-derived factor, PEDF (NM_011340.3); Lens epithelium-derived growth factor, LEDGF (NM_133948.4); Fibroblast growth factor-5, FGF-5 (NM_010203.4); Fibroblast growth factor-18, FGF-18 (NM_008005.1); Interleukin 1ß, IL1ß; Rod-derived cone survival factor, RDCVF (NM_145598.2); Insulin-like growth factor II, IGFII (NM_001122736.1); Colony stimulating factor, CSF-1 (NM_001113529.1); and LIF are induced in Müller cells by CNTF [64]–[72]. This observation may argue against the Müller cell hypothesis of neuroprotection. It is, nevertheless, possible that other growth factors or cytokines induced by CNTF (Tables S1, S2) are involved in photoreceptor neuroprotection.

Two other mechanisms have been implicated in CNTF-mediated neuronal survival. One mechanism states that CNTF might enhance synthesis or distribution of glutamate transporters, which could improve glutamate handling resulting in less excitotoxic damage to neurons. In the rat striatum, CNTF has been reported to increase glutamate uptake by redistribution of glutamate transporters [39]. Although we have not examined glutamate transporter distribution, our data does not reveal an increase in glutamate transporter transcripts in response to CNTF (Table S1). Another mechanism involves CNTF-induced metabolic changes that enhance resistance to severe metabolic insults [40]. In CNTF-treated Müller cells, there were no changes in the expression glucose or lactate transporters (Table S1, S2).

CNTF has been reported to increase the long-term survival of retinal ganglion cells after axotomy [25], [26]. The mechanism underlying this neuroprotective effect is not known. CNTF could directly act on retinal ganglion cells (RGCs) or it could act on Müller cells to trigger production on RGC survival factors [73]–[82]. Alternatively, CNTF injection could lead to production of Oncomodulin or another RGC survival factor from retinal microglia/macrophages [83].

One limitation of the present study is that gene expression profiling could be potentially affected by post-mortem changes in gene transcription during tissue removal, cell sorting and RNA preparation. Independent in vivo measurements of gene expression will be needed to understand in situ CNTF response. Another concern is that we cannot completely rule out the possibility that there might be a few GFP-positive astrocytes among flow-sorted cells from GFAP-GFP transgenic retinas. But the majority of cells in the flow-sorted population are likely to be Müller cells with <1% contribution from astrocytes. The density of Müller cells in the mouse retina is ∼12,000 per mm^2^ [84]. The density of astrocytes in the mammalian retina is ∼600 per mm^2^ [85], which is 5% of Müller cells. If 10% GFP-positive astrocytes remain in one-month old, GFAP-EGFP mouse retina, astrocyte contamination in flow-sorted cells would amount to <1%. Therefore, the gene expression profile we have studied is largely representative of transcription profile changes in Müller cells rather than astrocytes.

### CNTF is an inducer of reactive gliosis in Müller cells

A variety of agents including growth factors, cytokines, glutamate and purines have been suggested as inducers of reactive gliosis in the CNS [86]. In the retina, cytokines belonging to the IL-6 family as well as bFGF have been shown to induce gliosis (as determined by GFAP induction) in Müller cells [43], [46], [87]. We found that several genes that are induced by CNTF in quiescent Müller cells are also upregulated in gliotic Müller cells from inherited and experimentally-induced retinal degenerations [49]. These data strongly suggest that CNTF can act as an inducer of gliosis in the retina. This inference is supported by the finding that CNTF is upregulated in response to retinal degeneration and injury that results in Müller cell gliosis [55], [56].

### Müller cells, astrocytes and RPE serve support functions

It is generally accepted that Müller cells are functionally similar to astrocytes [42], although they are generated through different lineages [41]. Whereas Müller cells are derived from a retinal progenitor common to rods, bipolar cells and Müller cells [88], retinal astrocytes are generated from an oligodendrocyte/astrocyte precursor [89]. A comparison of transcript profiles of Müller cells and astrocytes shows that of the 2000 highly expressed genes, 642 genes (32%) were common to Müller cells and astrocytes. A closer examination shows that for many genes, the level of expression, however, is different between the two cell types, consistent with the previous transcriptome data on Müller cells [90]. In addition, there is a significant overlap (∼14%) among genes expressed by Müller cells, astrocytes and RPE. Muller cells and astrocytes are believed to have analogous functions in the CNS [41]. The RPE has been considered a separate entity. The present study, however, suggests that the RPE shares some metabolic pathways with Muller cells and astrocytes; it appears that Müller cells, astrocytes and RPE have evolved to support the metabolic needs of the neural retina.

In summary, the present study shows that (i) CNTF induces rapid and extensive changes in the transcriptional profile of Müller (glial) cells; (ii) that several genes induced by CNTF in normal Müller cells are also upregulated in gliotic Müller cells from inherited and experimentally-induced retinal degenerations, which suggests that CNTF is an inducer of gliosis in the retina; and (iii) the transcript profiles of Müller cells and astrocytes are similar although they are derived from distinct cell lineages. Finally, CNTF induces networks in which genes associated with transcription, cell cycle regulation and inflammatory response appear to occupy prominent positions (hubs) in the network topology.

## Materials and Methods

### Mice

All mice were used in accordance with the approved Northwestern University Institutional Animal Care and Use Committee (IACUC) protocol, 2008-1398 that specifically approved this study. Northwestern University Animal Welfare assurance is on file with the Office of Laboratory Animal Welfare (A3283-01).

### Purifying GFP^+^-Müller cells by FACS

We previously described a transgenic mouse line, *mgfap-egfp*, in which GFP is expressed only in retinal Müller cells [48]. We used the transgenic mice to purify Müller cells based on GFP-expression. CNTF or PBS was intravitreally injected into anesthetized, transgenic mice (one month old) as described earlier [42]. We used one eye for CNTF treatment and the fellow eye as PBS control. In order to ensure CNTF action, we routinely selected a few injected animals and examined retinal sections for GFAP expression in Müller cells [48]. Similarly, we also examined PBS injected eyes. We always found GFAP expression in Müller cells in the CNTF- but not PBS-injected eyes

Retinas from *mgfap-egfp* transgenic mice were treated with papain for 30 min, and dissociated into a single cell suspension. The cells were filtered through a 70 µm nytex mesh to get rid of cell clumps. GFP^+^-cells were enriched by FACS (MoFlo; Dako, Carpintaria, CA) at Northwestern Flow Cytometry Core facility.

### Microarray data procurement and analysis

Microarray analysis was carried out as described previously [49]. We performed total RNA extraction (TRIZOL; Invitrogen, Carlsbad, CA) from (∼50–100,000) flow-sorted cells, and confirmed the integrity of RNA with a bioanalyzer (model 2100; Agilent Technologies, Palo Alto, CA). All RNA samples had average RIN of 8.66±0.79 (12 samples). We synthesized, labeled, and hybridized cRNA onto arrays at Genome Explorations (Memphis, TN) according to standard Affymetrix methods, previously described [49]. We performed transcriptional profiling using mouse Affymetrix chips (mouse Expression Array 230 2.0). The experiments were done in biological triplicates and microarray data were analyzed as reported previously [49].

For Day 1, four biological replicates were used on Affymetrix GeneChip Mouse Genome MOE430 2.0. For Day 3 we also used 4 biological replicates on Affymetrix Exon Chip MoEx ST1.v1. Raw data were normalized and analyzed using GeneSpring GX 11.0.2 software (Silicon Genetics, Redwood City, CA). For normalization we used Robust Multichip Average (RMA) method. We performed background correction followed by quantile normalization and used the average mean for summarization. We performed a one-way ANOVA (a simple t-test for this simple situation) for comparison between CNTF and PBS at Day 1 and Day 3. We used an un-corrected p-value ≤0.1 which led to a total of 5336 probes for Day 1 and 6777 for Day 3. These two sets were subjected to a 2-fold change filter value between CNTF and PBS at Day 1 and Day 3. This resulted in a set of 1261 for Day 1 and 1541 probes for Day 3, respectively. Moreover, for Day 1 we obtained 1063 independent genes while for Day 3 the list contained 1530 genes.

### RNA preparation and Real-Time PCR Analysis (qPCR)

qPCR analysis of FACS-purified Müller cells was carried out to validate the findings from the microarray data [23]. We performed primer design, one-step RT-PCR reaction, and qPCR analysis as previously described [49]. Primers were designed using the PrimerQuest^SM^ IDT-DNA site. To eliminate genomic contamination, we treated total RNA with RQ1 RNase-free DNase (Promega, Madison, WI). We performed qPCR reaction in a thermocycler (iCycler; Bio-Rad, Richmond, CA), using the reagents in the SYBR Green iQ Real-time PCR kit; (Bio-Rad, Richmond, CA). To determine the relative change in gene expression, we used the system software to compare the number of cycles (C_t_) needed to reach the midpoint of the linear phase. All observations were normalized to the housekeeping gene *Gapdh*. We used actin, *hsp90* or *hprt1* as additional controls along with *Gapdh*. The qPCR data are from 3-6 biological replicates.

## Supporting Information

Table S1 Supporting table.
(DOC)

Table S2 Supporting table.
(DOC
