# Supplementary material for: Ciliary Neurotrophic Factor Induces Genes Associated with Inflammation and Gliosis in the Retina: A Gene Profiling Study of Flow-Sorted, Müller Cells
Source: PLoS One. 2011 May 26;6(5):e20326. doi: 10.1371/journal.pone.0020326 (PMC3102695; doi:10.1371/journal.pone.0020326)
Supplement: Table S1 — Supporting table. (DOC) [file pone.0020326.s001.doc]

| **Gene Symbol** | **RefSeq Transcript ID** | **Gene Title** | **Fold-Change(CNTF vs. PBS)** | **Fold-Change(CNTF vs. PBS) (Description)** |
| --- | --- | --- | --- | --- |
| Tgfbi | NM_009369 | transforming growth factor, beta induced | 17.37 | CNTF up vs PBS |
| Hmox1 | NM_010442 | heme oxygenase (decycling) 1 | 15.96 | CNTF up vs PBS |
| Tgfbi | NM_009369 | transforming growth factor, beta induced | 15.56 | CNTF up vs PBS |
| Tgfbi | NM_009369 | transforming growth factor, beta induced | 15.24 | CNTF up vs PBS |
| Tgfbi | NM_009369 | transforming growth factor, beta induced | 15.13 | CNTF up vs PBS |
| Gm11428 | NM_001081957 | predicted gene 11428 | 14.81 | CNTF up vs PBS |
| Ccl6 | NM_009139 | chemokine (C-C motif) ligand 6 | 13.58 | CNTF up vs PBS |
| Ccl5 | NM_013653 | chemokine (C-C motif) ligand 5 | 13.36 | CNTF up vs PBS |
| Ccrl2 | NM_017466 | chemokine (C-C motif) receptor-like 2 | 12.54 | CNTF up vs PBS |
| Clec7a | NM_020008 | C-type lectin domain family 7, member a | 12.03 | CNTF up vs PBS |
| 9330175E14Rik | NM_177146 /// NR_015514 | RIKEN cDNA 9330175E14 gene | 11.97 | CNTF up vs PBS |
| Gp49a /// Lilrb4 | NM_008147 /// NM_013532 | glycoprotein 49 A /// leukocyte immunoglobulin-like receptor, subfamily B, membe | 11.83 | CNTF up vs PBS |
| Ccl6 | NM_009139 | chemokine (C-C motif) ligand 6 | 11.17 | CNTF up vs PBS |
| Fcgr2b | NM_001077189 /// NM_010187 | Fc receptor, IgG, low affinity IIb | 11.05 | CNTF up vs PBS |
| Iigp1 | NM_001146275 /// NM_021792 | interferon inducible GTPase 1 | 11.00 | CNTF up vs PBS |
| Ifi205 /// Mnda | NM_001033450 /// NM_172648 | interferon activated gene 205 /// myeloid cell nuclear differentiation antigen | 10.82 | CNTF up vs PBS |
| Clec4e | NM_019948 | C-type lectin domain family 4, member e | 10.79 | CNTF up vs PBS |
| Myo1f | NM_053214 | myosin IF | 10.36 | CNTF up vs PBS |
| Ptgs2 | NM_011198 | prostaglandin-endoperoxide synthase 2 | 10.33 | CNTF up vs PBS |
| Spp1 | NM_009263 | secreted phosphoprotein 1 | 10.06 | CNTF up vs PBS |
| Cytip | NM_139200 | cytohesin 1 interacting protein | 9.93 | CNTF up vs PBS |
| AI662270 | NR_015519 /// XM_001474264 /// XM_001480989 | expressed sequence AI662270 | 9.93 | CNTF up vs PBS |
| Ms4a6d | NM_026835 | membrane-spanning 4-domains, subfamily A, member 6D | 9.93 | CNTF up vs PBS |
| Vav1 | NM_001163815 /// NM_001163816 /// NM_011691 | vav 1 oncogene | 9.73 | CNTF up vs PBS |
| Dab2 | NM_001008702 /// NM_001037905 /// NM_001102400 /// NM_023118 | disabled homolog 2 (Drosophila) | 9.71 | CNTF up vs PBS |
| Gm6377 | NM_001037917 | predicted gene 6377 | 9.68 | CNTF up vs PBS |
| Ms4a4c | NM_029499 | membrane-spanning 4-domains, subfamily A, member 4C | 9.62 | CNTF up vs PBS |
| Cxcl2 | NM_009140 | chemokine (C-X-C motif) ligand 2 | 9.44 | CNTF up vs PBS |
| Niacr1 | NM_030701 | niacin receptor 1 | 9.43 | CNTF up vs PBS |
| Cd14 | NM_009841 | CD14 antigen | 9.31 | CNTF up vs PBS |
| Ms4a6d | NM_026835 | membrane-spanning 4-domains, subfamily A, member 6D | 9.12 | CNTF up vs PBS |
| Il7r | NM_008372 | interleukin 7 receptor | 8.97 | CNTF up vs PBS |
| Ccr1 | NM_009912 | chemokine (C-C motif) receptor 1 | 8.92 | CNTF up vs PBS |
| Cdsn | NM_001008424 /// XM_001471477 | corneodesmosin | 8.91 | CNTF up vs PBS |
| Irg1 | NM_008392 /// XM_127883 /// XM_905897 | immunoresponsive gene 1 | 8.83 | CNTF up vs PBS |
| Srgn | NM_011157 | serglycin | 8.81 | CNTF up vs PBS |
| Egr2 | NM_010118 | early growth response 2 | 8.53 | CNTF up vs PBS |
| Cxcl1 | NM_008176 | chemokine (C-X-C motif) ligand 1 | 8.51 | CNTF up vs PBS |
| Msr1 | NM_001113326 /// NM_031195 | macrophage scavenger receptor 1 | 8.39 | CNTF up vs PBS |
| Rgs1 | NM_015811 | regulator of G-protein signaling 1 | 8.34 | CNTF up vs PBS |
| Ccl9 | NM_011338 | chemokine (C-C motif) ligand 9 | 8.10 | CNTF up vs PBS |
| Clec4d | NM_001163161 /// NM_010819 | C-type lectin domain family 4, member d | 8.08 | CNTF up vs PBS |
| Tlr2 | NM_011905 | toll-like receptor 2 | 8.06 | CNTF up vs PBS |
| Gpnmb | NM_053110 | glycoprotein (transmembrane) nmb | 8.05 | CNTF up vs PBS |
| Cd69 | NM_001033122 | CD69 antigen | 7.99 | CNTF up vs PBS |
| Tnf | NM_013693 | tumor necrosis factor | 7.88 | CNTF up vs PBS |
| Ccr1 | NM_009912 | chemokine (C-C motif) receptor 1 | 7.88 | CNTF up vs PBS |
| Clec4a3 | NM_153197 | C-type lectin domain family 4, member a3 | 7.88 | CNTF up vs PBS |
| Ncf4 | NM_008677 | neutrophil cytosolic factor 4 | 7.84 | CNTF up vs PBS |
| C5ar1 | NM_007577 | complement component 5a receptor 1 | 7.78 | CNTF up vs PBS |
| Emilin2 | NM_145158 | elastin microfibril interfacer 2 | 7.71 | CNTF up vs PBS |
| Egr2 | NM_010118 | early growth response 2 | 7.68 | CNTF up vs PBS |
| Tlr1 | NM_030682 | toll-like receptor 1 | 7.59 | CNTF up vs PBS |
| Emp1 | NM_010128 | epithelial membrane protein 1 | 7.58 | CNTF up vs PBS |
| Fcgr2b | NM_001077189 /// NM_010187 | Fc receptor, IgG, low affinity IIb | 7.54 | CNTF up vs PBS |
| Il1rn | NM_001039701 /// NM_001159562 /// NM_031167 | interleukin 1 receptor antagonist | 7.54 | CNTF up vs PBS |
| Atf3 | NM_007498 | activating transcription factor 3 | 7.51 | CNTF up vs PBS |
| Klf4 | NM_010637 | Kruppel-like factor 4 (gut) | 7.45 | CNTF up vs PBS |
| AI607873 | XM_001479984 /// XM_980696 | expressed sequence AI607873 | 7.45 | CNTF up vs PBS |
| Arhgap30 | NM_001005508 | Rho GTPase activating protein 30 | 7.45 | CNTF up vs PBS |
| Ifi44 | NM_133871 | interferon-induced protein 44 | 7.43 | CNTF up vs PBS |
| Gm885 | NM_001033435 | predicted gene 885 | 7.40 | CNTF up vs PBS |
| Itgb2 | NM_008404 | integrin beta 2 | 7.39 | CNTF up vs PBS |
| Acp5 | NM_001102404 /// NM_001102405 /// NM_007388 | acid phosphatase 5, tartrate resistant | 7.38 | CNTF up vs PBS |
| Il1rn | NM_001039701 /// NM_001159562 /// NM_031167 | interleukin 1 receptor antagonist | 7.37 | CNTF up vs PBS |
| Gm2785 /// Ifi203 /// Ifi204 /// Ifi205 /// LOC192690 /// LOC640890 /// Mnda | NM_001033450 /// NM_001045481 /// NM_008328 /// NM_008329 /// NM_172648 /// XM_0 | predicted gene 2785 /// interferon activated gene 203 /// interferon activated g | 7.31 | CNTF up vs PBS |
| Slc11a1 | NM_013612 | solute carrier family 11 (proton-coupled divalent metal ion transporters), membe | 7.30 | CNTF up vs PBS |
| Mpeg1 | NM_010821 | macrophage expressed gene 1 | 7.30 | CNTF up vs PBS |
| Lcp1 | NM_008879 | lymphocyte cytosolic protein 1 | 7.28 | CNTF up vs PBS |
| Csf2rb | NM_007780 | colony stimulating factor 2 receptor, beta, low-affinity (granulocyte-macrophage | 7.23 | CNTF up vs PBS |
| Rtp4 | NM_023386 | receptor transporter protein 4 | 7.22 | CNTF up vs PBS |
| Ifi203 | NM_001045481 /// NM_008328 | interferon activated gene 203 | 7.17 | CNTF up vs PBS |
| Hck | NM_010407 | hemopoietic cell kinase | 7.12 | CNTF up vs PBS |
| Capg | NM_001042534 /// NM_007599 | capping protein (actin filament), gelsolin-like | 7.10 | CNTF up vs PBS |
| Pik3ap1 | NM_031376 | phosphoinositide-3-kinase adaptor protein 1 | 7.04 | CNTF up vs PBS |
| Emr1 | NM_010130 | EGF-like module containing, mucin-like, hormone receptor-like sequence 1 | 7.03 | CNTF up vs PBS |
| Zbp1 | NM_001139519 /// NM_021394 | Z-DNA binding protein 1 | 6.99 | CNTF up vs PBS |
| Cmklr1 | NM_008153 | chemokine-like receptor 1 | 6.97 | CNTF up vs PBS |
| Fyb | NM_011815 | FYN binding protein | 6.93 | CNTF up vs PBS |
| Ptgs2 | NM_011198 | prostaglandin-endoperoxide synthase 2 | 6.93 | CNTF up vs PBS |
| Oasl1 | NM_145209 | 2'-5' oligoadenylate synthetase-like 1 | 6.91 | CNTF up vs PBS |
| Ifi27l2a | NM_029803 | interferon, alpha-inducible protein 27 like 2A | 6.89 | CNTF up vs PBS |
| Irak3 | NM_028679 | interleukin-1 receptor-associated kinase 3 | 6.88 | CNTF up vs PBS |
| Bcl3 | NM_033601 | B-cell leukemia/lymphoma 3 | 6.87 | CNTF up vs PBS |
| Tnfaip2 | NM_009396 | tumor necrosis factor, alpha-induced protein 2 | 6.83 | CNTF up vs PBS |
| Cxcl1 | NM_008176 | chemokine (C-X-C motif) ligand 1 | 6.76 | CNTF up vs PBS |
| Steap4 | NM_054098 | STEAP family member 4 | 6.76 | CNTF up vs PBS |
| Iigp1 | NM_001146275 /// NM_021792 | interferon inducible GTPase 1 | 6.72 | CNTF up vs PBS |
| Edn2 | NM_007902 | endothelin 2 | 6.72 | CNTF up vs PBS |
| Fcgr2b | NM_001077189 /// NM_010187 | Fc receptor, IgG, low affinity IIb | 6.64 | CNTF up vs PBS |
| Xdh | NM_011723 | xanthine dehydrogenase | 6.63 | CNTF up vs PBS |
| Pf4 | NM_019932 | platelet factor 4 | 6.59 | CNTF up vs PBS |
| Il1r2 | NM_010555 | interleukin 1 receptor, type II | 6.58 | CNTF up vs PBS |
| Fgr | NM_010208 | Gardner-Rasheed feline sarcoma viral (Fgr) oncogene homolog | 6.58 | CNTF up vs PBS |
| C3ar1 | NM_009779 | complement component 3a receptor 1 | 6.55 | CNTF up vs PBS |
| Ms4a6b | NM_027209 | membrane-spanning 4-domains, subfamily A, member 6B | 6.54 | CNTF up vs PBS |
| Csf2rb2 | NM_007781 | colony stimulating factor 2 receptor, beta 2, low-affinity (granulocyte-macropha | 6.53 | CNTF up vs PBS |
| Ptprc | NM_001111316 /// NM_011210 | protein tyrosine phosphatase, receptor type, C | 6.53 | CNTF up vs PBS |
| A130040M12Rik | NR_002860 | RIKEN cDNA A130040M12 gene | 6.50 | CNTF up vs PBS |
| Pyhin1 | NM_175026 | pyrin and HIN domain family, member 1 | 6.49 | CNTF up vs PBS |
| Ifi203 | NM_001045481 /// NM_008328 | interferon activated gene 203 | 6.44 | CNTF up vs PBS |
| Ggta1 | NM_001145821 /// NM_010283 | glycoprotein galactosyltransferase alpha 1, 3 | 6.43 | CNTF up vs PBS |
| Fgl2 | NM_008013 | fibrinogen-like protein 2 | 6.39 | CNTF up vs PBS |
| Ccl3 | NM_011337 | chemokine (C-C motif) ligand 3 | 6.38 | CNTF up vs PBS |
| Irf8 | NM_008320 | interferon regulatory factor 8 | 6.36 | CNTF up vs PBS |
| Edem1 | NM_138677 | ER degradation enhancer, mannosidase alpha-like 1 | 6.36 | CNTF up vs PBS |
| Gm3756 /// Gm5620 /// Gm7172 /// LOC100044416 /// LOC100045728 /// Tuba1a /// Tuba1b /// Tuba1c | NM_009448 /// NM_011653 /// NM_011654 /// XM_486246 /// XM_896498 /// XM_904657 | predicted gene 3756 /// predicted gene 5620 /// predicted gene 7172 /// similar | 6.35 | CNTF up vs PBS |
| Gvin1 | NM_001039160 /// NM_029000 | GTPase, very large interferon inducible 1 | 6.27 | CNTF up vs PBS |
| Plek | NM_019549 | pleckstrin | 6.19 | CNTF up vs PBS |
| Ms4a6c | NM_028595 | membrane-spanning 4-domains, subfamily A, member 6C | 6.17 | CNTF up vs PBS |
| Bcl2a1a /// Bcl2a1b /// Bcl2a1d | NM_007534 /// NM_007536 /// NM_009742 | B-cell leukemia/lymphoma 2 related protein A1a /// B-cell leukemia/lymphoma 2 re | 6.16 | CNTF up vs PBS |
| Ifi204 | NM_008329 | interferon activated gene 204 | 6.12 | CNTF up vs PBS |
| Msr1 | NM_001113326 /// NM_031195 | macrophage scavenger receptor 1 | 6.11 | CNTF up vs PBS |
| C3ar1 | NM_009779 | complement component 3a receptor 1 | 6.09 | CNTF up vs PBS |
| Clec4a2 /// Clec4b1 | NM_011999 /// NM_027218 | C-type lectin domain family 4, member a2 /// C-type lectin domain family 4, memb | 6.09 | CNTF up vs PBS |
| Tgm2 | NM_009373 | transglutaminase 2, C polypeptide | 6.09 | CNTF up vs PBS |
| Ncf1 | NM_010876 | neutrophil cytosolic factor 1 | 6.05 | CNTF up vs PBS |
| Lgals3 | NM_001145953 /// NM_010705 | lectin, galactose binding, soluble 3 | 6.05 | CNTF up vs PBS |
| Lst1 | NM_010734 | leukocyte specific transcript 1 | 6.02 | CNTF up vs PBS |
| Trim30 | NM_009099 | tripartite motif-containing 30 | 6.01 | CNTF up vs PBS |
| Fgr | NM_010208 | Gardner-Rasheed feline sarcoma viral (Fgr) oncogene homolog | 5.99 | CNTF up vs PBS |
| Rab32 | NM_026405 | RAB32, member RAS oncogene family | 5.98 | CNTF up vs PBS |
| Il13ra1 | NM_133990 | interleukin 13 receptor, alpha 1 | 5.92 | CNTF up vs PBS |
| Bst2 | NM_198095 | bone marrow stromal cell antigen 2 | 5.92 | CNTF up vs PBS |
| Fcgr4 | NM_144559 | Fc receptor, IgG, low affinity IV | 5.90 | CNTF up vs PBS |
| Lyz2 | NM_017372 | lysozyme 2 | 5.88 | CNTF up vs PBS |
| Slfn2 | NM_011408 | schlafen 2 | 5.88 | CNTF up vs PBS |
| Pmaip1 | NM_021451 | phorbol-12-myristate-13-acetate-induced protein 1 | 5.87 | CNTF up vs PBS |
| Naip5 | NM_010870 | NLR family, apoptosis inhibitory protein 5 | 5.86 | CNTF up vs PBS |
| Cd84 | NM_013489 | CD84 antigen | 5.81 | CNTF up vs PBS |
| Rbm47 | NM_001127382 /// NM_139065 /// NM_178446 | RNA binding motif protein 47 | 5.79 | CNTF up vs PBS |
| Fcer1g | NM_010185 | Fc receptor, IgE, high affinity I, gamma polypeptide | 5.73 | CNTF up vs PBS |
| Ugt1a1 /// Ugt1a10 /// Ugt1a2 /// Ugt1a5 /// Ugt1a6a /// Ugt1a6b /// Ugt1a7c /// Ugt1a9 | NM_013701 /// NM_145079 /// NM_201410 /// NM_201641 /// NM_201642 /// NM_201643 | UDP glucuronosyltransferase 1 family, polypeptide A1 /// UDP glycosyltransferase | 5.73 | CNTF up vs PBS |
| Cxcr4 | NM_009911 /// XM_001478244 | chemokine (C-X-C motif) receptor 4 | 5.67 | CNTF up vs PBS |
| B430306N03Rik | NM_177083 | RIKEN cDNA B430306N03 gene | 5.67 | CNTF up vs PBS |
| Cybb | NM_007807 | cytochrome b-245, beta polypeptide | 5.67 | CNTF up vs PBS |
| Slc15a3 | NM_023044 | solute carrier family 15, member 3 | 5.65 | CNTF up vs PBS |
| Slc13a3 | NM_054055 | solute carrier family 13 (sodium-dependent dicarboxylate transporter), member 3 | 5.62 | CNTF up vs PBS |
| Irgm2 | NM_019440 | immunity-related GTPase family M member 2 | 5.61 | CNTF up vs PBS |
| Pik3r6 | NM_001004435 /// NM_001081566 | phosphoinositide-3-kinase, regulatory subunit 6 | 5.61 | CNTF up vs PBS |
| Ikbke | NM_019777 | inhibitor of kappaB kinase epsilon | 5.57 | CNTF up vs PBS |
| AI451617 | NM_199146 | expressed sequence AI451617 | 5.51 | CNTF up vs PBS |
| Cd93 | NM_010740 | CD93 antigen | 5.51 | CNTF up vs PBS |
| Ikzf1 | NM_001025597 /// NM_009578 | IKAROS family zinc finger 1 | 5.50 | CNTF up vs PBS |
| 4632428N05Rik | NM_001159572 /// NM_028732 | RIKEN cDNA 4632428N05 gene | 5.50 | CNTF up vs PBS |
| Fcgr3 | NM_010188 | Fc receptor, IgG, low affinity III | 5.47 | CNTF up vs PBS |
| Syk | NM_011518 | spleen tyrosine kinase | 5.45 | CNTF up vs PBS |
| Tap1 | NM_001161730 /// NM_013683 | transporter 1, ATP-binding cassette, sub-family B (MDR/TAP) | 5.45 | CNTF up vs PBS |
| E330016A19Rik | NM_173386 | RIKEN cDNA E330016A19 gene | 5.44 | CNTF up vs PBS |
| Cd244 | NM_018729 | CD244 natural killer cell receptor 2B4 | 5.43 | CNTF up vs PBS |
| Gfap | NM_001131020 /// NM_010277 | glial fibrillary acidic protein | 5.42 | CNTF up vs PBS |
| Naip2 | NM_001126182 /// NM_010872 | NLR family, apoptosis inhibitory protein 2 | 5.39 | CNTF up vs PBS |
| Sla | NM_001029841 /// NM_009192 | src-like adaptor | 5.38 | CNTF up vs PBS |
| Itgax | NM_021334 | integrin alpha X | 5.38 | CNTF up vs PBS |
| AB124611 | NM_206536 | cDNA sequence AB124611 | 5.37 | CNTF up vs PBS |
| C130050O18Rik | NM_177000 | RIKEN cDNA C130050O18 gene | 5.36 | CNTF up vs PBS |
| Pilrb1 | NM_133209 | paired immunoglobin-like type 2 receptor beta 1 | 5.35 | CNTF up vs PBS |
| LOC100048346 /// Usp18 | NM_011909 /// XM_001480051 | similar to ubiquitin specific protease UBP43 /// ubiquitin specific peptidase 18 | 5.33 | CNTF up vs PBS |
| Cd53 | NM_007651 | CD53 antigen | 5.33 | CNTF up vs PBS |
| Ifit1 | NM_008331 | interferon-induced protein with tetratricopeptide repeats 1 | 5.33 | CNTF up vs PBS |
| Arpc1b | NM_023142 | actin related protein 2/3 complex, subunit 1B | 5.32 | CNTF up vs PBS |
| Gmfg | NM_001039192 /// NM_022024 | glia maturation factor, gamma | 5.32 | CNTF up vs PBS |
| Igsf6 | NM_030691 | immunoglobulin superfamily, member 6 | 5.32 | CNTF up vs PBS |
| Casp4 | NM_007609 | caspase 4, apoptosis-related cysteine peptidase | 5.32 | CNTF up vs PBS |
| Oas1a | NM_145211 | 2'-5' oligoadenylate synthetase 1A | 5.30 | CNTF up vs PBS |
| Birc1f | NM_010871 | baculoviral IAP repeat-containing 1f | 5.30 | CNTF up vs PBS |
| Tgm2 | NM_009373 | transglutaminase 2, C polypeptide | 5.27 | CNTF up vs PBS |
| Pilra | NM_153510 | paired immunoglobin-like type 2 receptor alpha | 5.25 | CNTF up vs PBS |
| Mcl1 | NM_008562 | myeloid cell leukemia sequence 1 | 5.25 | CNTF up vs PBS |
| Lcp1 | NM_008879 | lymphocyte cytosolic protein 1 | 5.23 | CNTF up vs PBS |
| Irgm1 | NM_008326 | immunity-related GTPase family M member 1 | 5.22 | CNTF up vs PBS |
| Trem1 | NM_021406 | triggering receptor expressed on myeloid cells 1 | 5.22 | CNTF up vs PBS |
| Casp8 | NM_001080126 /// NM_009812 | caspase 8 | 5.19 | CNTF up vs PBS |
| Baz1a | NM_013815 /// XM_885873 /// XM_915565 | bromodomain adjacent to zinc finger domain 1A | 5.18 | CNTF up vs PBS |
| Il1a | NM_010554 | interleukin 1 alpha | 5.16 | CNTF up vs PBS |
| Slpi | NM_011414 | secretory leukocyte peptidase inhibitor | 5.15 | CNTF up vs PBS |
| 1810011O10Rik | NM_026931 | RIKEN cDNA 1810011O10 gene | 5.15 | CNTF up vs PBS |
| Rrbp1 | NM_024281 /// NM_133626 | ribosome binding protein 1 | 5.13 | CNTF up vs PBS |
| Cd86 | NM_019388 | CD86 antigen | 5.09 | CNTF up vs PBS |
| Cd68 | NM_009853 | CD68 antigen | 5.09 | CNTF up vs PBS |
| Rsad2 | NM_021384 | radical S-adenosyl methionine domain containing 2 | 5.08 | CNTF up vs PBS |
| Icam1 | NM_010493 | intercellular adhesion molecule 1 | 5.08 | CNTF up vs PBS |
| Pycard | NM_023258 | PYD and CARD domain containing | 5.06 | CNTF up vs PBS |
| BC013712 | NM_001033308 | cDNA sequence BC013712 | 5.05 | CNTF up vs PBS |
| Hspa1a | NM_010479 | heat shock protein 1A | 5.04 | CNTF up vs PBS |
| Serpina3n | NM_009252 | serine (or cysteine) peptidase inhibitor, clade A, member 3N | 5.03 | CNTF up vs PBS |
| C3 | NM_009778 | complement component 3 | 5.01 | CNTF up vs PBS |
| Emp3 | NM_001146346 /// NM_010129 | epithelial membrane protein 3 | 5.01 | CNTF up vs PBS |
| Myo1f | NM_053214 | myosin IF | 5.00 | CNTF up vs PBS |
| Cdkn1a | NM_001111099 /// NM_007669 | cyclin-dependent kinase inhibitor 1A (P21) | 4.94 | CNTF up vs PBS |
| Anxa2 | NM_007585 | annexin A2 | 4.93 | CNTF up vs PBS |
| Ms4a4c | NM_029499 | membrane-spanning 4-domains, subfamily A, member 4C | 4.90 | CNTF up vs PBS |
| Lilrb3 | NM_011095 | leukocyte immunoglobulin-like receptor, subfamily B (with TM and ITIM domains), | 4.89 | CNTF up vs PBS |
| Slc13a3 | NM_054055 | solute carrier family 13 (sodium-dependent dicarboxylate transporter), member 3 | 4.89 | CNTF up vs PBS |
| Ms4a7 | NM_001025610 /// NM_027836 | membrane-spanning 4-domains, subfamily A, member 7 | 4.88 | CNTF up vs PBS |
| Igtp | NM_018738 | interferon gamma induced GTPase | 4.86 | CNTF up vs PBS |
| Ltbp2 | NM_013589 | latent transforming growth factor beta binding protein 2 | 4.86 | CNTF up vs PBS |
| Ifi47 | NM_008330 | interferon gamma inducible protein 47 | 4.86 | CNTF up vs PBS |
| Lyz1 | NM_013590 | lysozyme 1 | 4.85 | CNTF up vs PBS |
| Serp1 | NM_030685 | stress-associated endoplasmic reticulum protein 1 | 4.85 | CNTF up vs PBS |
| Tgm2 | NM_009373 | transglutaminase 2, C polypeptide | 4.84 | CNTF up vs PBS |
| Btk | NM_013482 | Bruton agammaglobulinemia tyrosine kinase | 4.84 | CNTF up vs PBS |
| Pcolce | NM_008788 | procollagen C-endopeptidase enhancer protein | 4.82 | CNTF up vs PBS |
| Saa1 | NM_009117 | serum amyloid A 1 | 4.82 | CNTF up vs PBS |
| Procr | NM_011171 | protein C receptor, endothelial | 4.81 | CNTF up vs PBS |
| Tlr13 | NM_205820 | toll-like receptor 13 | 4.81 | CNTF up vs PBS |
| Skap2 | NM_018773 | src family associated phosphoprotein 2 | 4.80 | CNTF up vs PBS |
| Dock2 | NM_033374 | dedicator of cyto-kinesis 2 | 4.80 | CNTF up vs PBS |
| Il2rg | NM_013563 | interleukin 2 receptor, gamma chain | 4.78 | CNTF up vs PBS |
| Tgm2 | NM_009373 | transglutaminase 2, C polypeptide | 4.76 | CNTF up vs PBS |
| Arhgap9 | NM_146011 | Rho GTPase activating protein 9 | 4.73 | CNTF up vs PBS |
| E2f8 | NM_001013368 | E2F transcription factor 8 | 4.72 | CNTF up vs PBS |
| Ptk2b | NM_001162365 /// NM_001162366 /// NM_172498 | PTK2 protein tyrosine kinase 2 beta | 4.70 | CNTF up vs PBS |
| Cxcl10 | NM_021274 | chemokine (C-X-C motif) ligand 10 | 4.69 | CNTF up vs PBS |
| Fn1 | NM_010233 | fibronectin 1 | 4.68 | CNTF up vs PBS |
| Vwf | NM_011708 | Von Willebrand factor homolog | 4.68 | CNTF up vs PBS |
| Gbp3 | NM_018734 | guanylate binding protein 3 | 4.66 | CNTF up vs PBS |
| Ncf2 | NM_010877 | neutrophil cytosolic factor 2 | 4.65 | CNTF up vs PBS |
| Rrbp1 | NM_024281 /// NM_133626 | ribosome binding protein 1 | 4.64 | CNTF up vs PBS |
| Cmtm7 | NM_133978 | CKLF-like MARVEL transmembrane domain containing 7 | 4.61 | CNTF up vs PBS |
| Gng12 | NM_025278 | guanine nucleotide binding protein (G protein), gamma 12 | 4.61 | CNTF up vs PBS |
| Cd48 | NM_007649 | CD48 antigen | 4.59 | CNTF up vs PBS |
| Plcg2 | NM_172285 | phospholipase C, gamma 2 | 4.59 | CNTF up vs PBS |
| Cd86 | NM_019388 | CD86 antigen | 4.56 | CNTF up vs PBS |
| Pik3cg | NM_001146200 /// NM_001146201 /// NM_020272 | phosphoinositide-3-kinase, catalytic, gamma polypeptide | 4.55 | CNTF up vs PBS |
| 5830462P14Rik | --- | RIKEN cDNA 5830462P14 gene | 4.55 | CNTF up vs PBS |
| C1r /// C1rb | NM_001113356 /// NM_023143 /// XM_001002804 /// XM_001480171 | complement component 1, r subcomponent /// complement component 1, r subcomponen | 4.55 | CNTF up vs PBS |
| Kcnk6 | NM_001033525 | potassium inwardly-rectifying channel, subfamily K, member 6 | 4.54 | CNTF up vs PBS |
| AF251705 | NM_134158 | cDNA sequence AF251705 | 4.53 | CNTF up vs PBS |
| Cybb | NM_007807 | cytochrome b-245, beta polypeptide | 4.53 | CNTF up vs PBS |
| Ptpn6 | NM_001077705 /// NM_013545 | protein tyrosine phosphatase, non-receptor type 6 | 4.52 | CNTF up vs PBS |
| Ugt1a1 /// Ugt1a10 /// Ugt1a2 /// Ugt1a5 /// Ugt1a6a /// Ugt1a6b /// Ugt1a7c /// Ugt1a9 | NM_013701 /// NM_145079 /// NM_201410 /// NM_201641 /// NM_201642 /// NM_201643 | UDP glucuronosyltransferase 1 family, polypeptide A1 /// UDP glycosyltransferase | 4.51 | CNTF up vs PBS |
| Tmem173 | NM_028261 /// XM_128954 /// XM_892499 /// XM_900126 /// XM_910347 /// XM_921603 | transmembrane protein 173 | 4.48 | CNTF up vs PBS |
| Was | NM_009515 | Wiskott-Aldrich syndrome homolog (human) | 4.48 | CNTF up vs PBS |
| Litaf | NM_019980 | LPS-induced TN factor | 4.48 | CNTF up vs PBS |
| P2ry6 | NM_183168 | pyrimidinergic receptor P2Y, G-protein coupled, 6 | 4.47 | CNTF up vs PBS |
| C920025E04Rik /// H2-T23 | NM_010398 /// XM_001473226 /// XM_975970 /// XM_992574 | RIKEN cDNA C920025E04 gene /// histocompatibility 2, T region locus 23 | 4.47 | CNTF up vs PBS |
| Ccl4 | NM_013652 | chemokine (C-C motif) ligand 4 | 4.46 | CNTF up vs PBS |
| Maff | NM_010755 | v-maf musculoaponeurotic fibrosarcoma oncogene family, protein F (avian) | 4.45 | CNTF up vs PBS |
| Ccr5 | NM_009917 | chemokine (C-C motif) receptor 5 | 4.44 | CNTF up vs PBS |
| Hk3 | NM_001033245 | hexokinase 3 | 4.43 | CNTF up vs PBS |
| Stab1 | NM_138672 | stabilin 1 | 4.41 | CNTF up vs PBS |
| Klf2 | NM_008452 | Kruppel-like factor 2 (lung) | 4.41 | CNTF up vs PBS |
| Arhgap9 | NM_146011 | Rho GTPase activating protein 9 | 4.41 | CNTF up vs PBS |
| Ncf1 | NM_010876 | neutrophil cytosolic factor 1 | 4.39 | CNTF up vs PBS |
| Lyz1 | NM_013590 | lysozyme 1 | 4.37 | CNTF up vs PBS |
| Ch25h | NM_009890 | cholesterol 25-hydroxylase | 4.36 | CNTF up vs PBS |
| Csf3r | NM_007782 | colony stimulating factor 3 receptor (granulocyte) | 4.35 | CNTF up vs PBS |
| Spsb1 | NM_029035 | SplA/ryanodine receptor domain and SOCS box containing 1 | 4.35 | CNTF up vs PBS |
| Sp100 | NM_013673 | nuclear antigen Sp100 | 4.34 | CNTF up vs PBS |
| Cd274 | NM_021893 | CD274 antigen | 4.32 | CNTF up vs PBS |
| Cd24a | NM_009846 | CD24a antigen | 4.32 | CNTF up vs PBS |
| Pon3 | NM_173006 | paraoxonase 3 | 4.30 | CNTF up vs PBS |
| Arhgap9 | NM_146011 | Rho GTPase activating protein 9 | 4.30 | CNTF up vs PBS |
| Slamf8 | NM_029084 /// XM_001480676 | SLAM family member 8 | 4.30 | CNTF up vs PBS |
| Nudt6 | NM_153561 | nudix (nucleoside diphosphate linked moiety X)-type motif 6 | 4.29 | CNTF up vs PBS |
| Abcc3 | NM_029600 | ATP-binding cassette, sub-family C (CFTR/MRP), member 3 | 4.29 | CNTF up vs PBS |
| Ugt1a1 /// Ugt1a10 /// Ugt1a2 /// Ugt1a5 /// Ugt1a6a /// Ugt1a6b /// Ugt1a7c /// Ugt1a9 | NM_013701 /// NM_145079 /// NM_201410 /// NM_201641 /// NM_201642 /// NM_201643 | UDP glucuronosyltransferase 1 family, polypeptide A1 /// UDP glycosyltransferase | 4.28 | CNTF up vs PBS |
| Tubb6 | NM_026473 | tubulin, beta 6 | 4.27 | CNTF up vs PBS |
| Il13ra1 | NM_133990 | interleukin 13 receptor, alpha 1 | 4.27 | CNTF up vs PBS |
| Cebpd | NM_007679 | CCAAT/enhancer binding protein (C/EBP), delta | 4.26 | CNTF up vs PBS |
| Cd74 | NM_001042605 /// NM_010545 | CD74 antigen (invariant polypeptide of major histocompatibility complex, class I | 4.25 | CNTF up vs PBS |
| Fcgr1 | NM_010186 | Fc receptor, IgG, high affinity I | 4.25 | CNTF up vs PBS |
| Galnt6 /// LOC100047499 | NM_001161767 /// NM_001161768 /// NM_172451 /// XR_034070 | UDP-N-acetyl-alpha-D-galactosamine:polypeptide N-acetylgalactosaminyltransferase | 4.24 | CNTF up vs PBS |
| Cebpa | NM_007678 | CCAAT/enhancer binding protein (C/EBP), alpha | 4.24 | CNTF up vs PBS |
| Gpr35 | NM_001104529 /// NM_022320 | G protein-coupled receptor 35 | 4.24 | CNTF up vs PBS |
| Ly9 | NM_008534 | lymphocyte antigen 9 | 4.22 | CNTF up vs PBS |
| Ptger4 | NM_001136079 /// NM_008965 | prostaglandin E receptor 4 (subtype EP4) | 4.22 | CNTF up vs PBS |
| Frrs1 /// LOC100046401 | NM_001113478 /// NM_009146 /// XR_032583 | ferric-chelate reductase 1 /// similar to SDR2 | 4.20 | CNTF up vs PBS |
| Cytip | NM_139200 | cytohesin 1 interacting protein | 4.19 | CNTF up vs PBS |
| C1r | NM_023143 | complement component 1, r subcomponent | 4.19 | CNTF up vs PBS |
| Spsb1 | NM_029035 | splA/ryanodine receptor domain and SOCS box containing 1 | 4.18 | CNTF up vs PBS |
| Lgals3bp | NM_011150 | lectin, galactoside-binding, soluble, 3 binding protein | 4.16 | CNTF up vs PBS |
| Aplnr | NM_011784 | apelin receptor | 4.15 | CNTF up vs PBS |
| Havcr2 | NM_134250 | hepatitis A virus cellular receptor 2 | 4.15 | CNTF up vs PBS |
| Tyrobp | NM_011662 | TYRO protein tyrosine kinase binding protein | 4.15 | CNTF up vs PBS |
| Clec5a | NM_001038604 /// NM_021364 | C-type lectin domain family 5, member a | 4.15 | CNTF up vs PBS |
| Rsad2 | NM_021384 | radical S-adenosyl methionine domain containing 2 | 4.13 | CNTF up vs PBS |
| Aif1 | NM_019467 | allograft inflammatory factor 1 | 4.13 | CNTF up vs PBS |
| AI429363 | --- | expressed sequence AI429363 | 4.12 | CNTF up vs PBS |
| Pyhin1 | NM_175026 | pyrin and HIN domain family, member 1 | 4.12 | CNTF up vs PBS |
| Tmem173 | NM_028261 /// XM_128954 /// XM_892499 /// XM_900126 /// XM_910347 /// XM_921603 | transmembrane protein 173 | 4.11 | CNTF up vs PBS |
| Plek | NM_019549 | pleckstrin | 4.09 | CNTF up vs PBS |
| Tspo | NM_009775 | translocator protein | 4.08 | CNTF up vs PBS |
| 4632428N05Rik | NM_001159572 /// NM_028732 | RIKEN cDNA 4632428N05 gene | 4.07 | CNTF up vs PBS |
| Evi2a | NM_001033711 /// NM_010161 | ecotropic viral integration site 2a | 4.05 | CNTF up vs PBS |
| Laptm5 | NM_010686 | lysosomal-associated protein transmembrane 5 | 4.04 | CNTF up vs PBS |
| Gm7669 /// Gm7901 /// Gm9347 /// Phgdh | NM_016966 /// XR_030810 /// XR_030923 /// XR_031967 /// XR_032429 /// XR_034648 | predicted gene 7669 /// 3-phosphoglycerate dehydrogenase pseudogene /// predicte | 4.03 | CNTF up vs PBS |
| Dusp2 | NM_010090 | dual specificity phosphatase 2 | 4.03 | CNTF up vs PBS |
| AU020206 | --- | expressed sequence AU020206 | 4.02 | CNTF up vs PBS |
| Irf2bp2 | XM_001002526 /// XM_284454 | interferon regulatory factor 2 binding protein 2 | 4.01 | CNTF up vs PBS |
| Osm | NM_001013365 | oncostatin M | 4.00 | CNTF up vs PBS |
| P2rx7 | NM_001038839 /// NM_001038845 /// NM_001038887 /// NM_011027 | purinergic receptor P2X, ligand-gated ion channel, 7 | 3.99 | CNTF up vs PBS |
| Parp9 | NM_030253 | poly (ADP-ribose) polymerase family, member 9 | 3.99 | CNTF up vs PBS |
| Tagln2 | NM_178598 | transgelin 2 | 3.98 | CNTF up vs PBS |
| Rac2 | NM_009008 | RAS-related C3 botulinum substrate 2 | 3.97 | CNTF up vs PBS |
| Ctsc | NM_009982 | cathepsin C | 3.97 | CNTF up vs PBS |
| A130040M12Rik | NR_002860 | RIKEN cDNA A130040M12 gene | 3.97 | CNTF up vs PBS |
| Evi2b | NM_001077496 /// NM_146023 | ecotropic viral integration site 2b | 3.96 | CNTF up vs PBS |
| Cd52 | NM_013706 | CD52 antigen | 3.95 | CNTF up vs PBS |
| Rab20 | NM_011227 | RAB20, member RAS oncogene family | 3.95 | CNTF up vs PBS |
| Ifi203 | NM_001045481 /// NM_008328 | interferon activated gene 203 | 3.94 | CNTF up vs PBS |
| Laptm5 | NM_010686 | lysosomal-associated protein transmembrane 5 | 3.93 | CNTF up vs PBS |
| Spata6 | NM_026470 | Spermatogenesis associated 6 | 3.93 | CNTF up vs PBS |
| Myo1f | NM_053214 | myosin IF | 3.92 | CNTF up vs PBS |
| Fam111a | NM_026640 | family with sequence similarity 111, member A | 3.91 | CNTF up vs PBS |
| Hspa1b | NM_010478 | heat shock protein 1B | 3.91 | CNTF up vs PBS |
| Irf8 | NM_008320 | interferon regulatory factor 8 | 3.91 | CNTF up vs PBS |
| Tspo | NM_009775 | translocator protein | 3.89 | CNTF up vs PBS |
| LOC676654 /// Lyn | NM_001111096 /// NM_010747 /// XM_991890 | similar to Yamaguchi sarcoma viral (v-yes-1) oncogene homolog /// Yamaguchi sarc | 3.89 | CNTF up vs PBS |
| Skap2 | NM_018773 | src family associated phosphoprotein 2 | 3.89 | CNTF up vs PBS |
| Mpa2l | NM_194336 | macrophage activation 2 like | 3.89 | CNTF up vs PBS |
| Zc3h12a | NM_153159 | zinc finger CCCH type containing 12A | 3.88 | CNTF up vs PBS |
| Atxn7l1 | NM_001033436 /// NM_028139 | ataxin 7-like 1 | 3.88 | CNTF up vs PBS |
| Tgfb1 | NM_011577 | transforming growth factor, beta 1 | 3.88 | CNTF up vs PBS |
| Sash3 | NM_028773 | SAM and SH3 domain containing 3 | 3.88 | CNTF up vs PBS |
| Zfp36 | NM_011756 | zinc finger protein 36 | 3.87 | CNTF up vs PBS |
| 1300002K09Rik | NM_028788 | RIKEN cDNA 1300002K09 gene | 3.84 | CNTF up vs PBS |
| Casp1 | NM_009807 | caspase 1 | 3.84 | CNTF up vs PBS |
| Fes | NM_010194 | feline sarcoma oncogene | 3.83 | CNTF up vs PBS |
| Cebpd | NM_007679 | CCAAT/enhancer binding protein (C/EBP), delta | 3.82 | CNTF up vs PBS |
| Inpp5d | NM_001110192 /// NM_001110193 /// NM_010566 | inositol polyphosphate-5-phosphatase D | 3.80 | CNTF up vs PBS |
| Spint1 | NM_016907 | serine protease inhibitor, Kunitz type 1 | 3.80 | CNTF up vs PBS |
| Apobec1 | NM_001134391 /// NM_031159 | apolipoprotein B mRNA editing enzyme, catalytic polypeptide 1 | 3.80 | CNTF up vs PBS |
| Gcnt1 | NM_001136484 /// NM_010265 /// NM_173442 | glucosaminyl (N-acetyl) transferase 1, core 2 | 3.79 | CNTF up vs PBS |
| Ripk1 | NM_009068 | receptor (TNFRSF)-interacting serine-threonine kinase 1 | 3.79 | CNTF up vs PBS |
| Irf7 | NM_016850 | interferon regulatory factor 7 | 3.78 | CNTF up vs PBS |
| Spata6 | NM_026470 | spermatogenesis associated 6 | 3.76 | CNTF up vs PBS |
| AI467606 | NM_178901 | expressed sequence AI467606 | 3.76 | CNTF up vs PBS |
| Cass4 | NM_001033538 /// NM_001080820 | Cas scaffolding protein family member 4 | 3.76 | CNTF up vs PBS |
| Tnfrsf1b | NM_011610 | tumor necrosis factor receptor superfamily, member 1b | 3.76 | CNTF up vs PBS |
| Zwilch | NM_026507 | Zwilch, kinetochore associated, homolog (Drosophila) | 3.75 | CNTF up vs PBS |
| Cd300a | NM_170758 | CD300A antigen | 3.75 | CNTF up vs PBS |
| A2m /// LOC677369 | NM_175628 /// XR_005046 | alpha-2-macroglobulin /// hypothetical protein LOC677369 | 3.75 | CNTF up vs PBS |
| Nckap1l | NM_153505 | NCK associated protein 1 like | 3.75 | CNTF up vs PBS |
| Alox5ap | NM_009663 | arachidonate 5-lipoxygenase activating protein | 3.74 | CNTF up vs PBS |
| Arhgap25 | NM_001037727 /// NM_175476 | Rho GTPase activating protein 25 | 3.73 | CNTF up vs PBS |
| Sfpi1 | NM_011355 | SFFV proviral integration 1 | 3.73 | CNTF up vs PBS |
| Pik3r6 | NM_001004435 /// NM_001081566 | phosphoinositide-3-kinase, regulatory subunit 6 | 3.72 | CNTF up vs PBS |
| Ddx58 | NM_172689 | DEAD (Asp-Glu-Ala-Asp) box polypeptide 58 | 3.72 | CNTF up vs PBS |
| Rel | NM_009044 | reticuloendotheliosis oncogene | 3.72 | CNTF up vs PBS |
| Pla2g4a | NM_008869 | phospholipase A2, group IVA (cytosolic, calcium-dependent) | 3.71 | CNTF up vs PBS |
| BC006779 | NM_183162 | cDNA sequence BC006779 | 3.70 | CNTF up vs PBS |
| Ang | NM_001161731 /// NM_007447 | angiogenin, ribonuclease, RNase A family, 5 | 3.69 | CNTF up vs PBS |
| Rin3 | NM_001161365 /// NM_177620 | Ras and Rab interactor 3 | 3.69 | CNTF up vs PBS |
| Cyp4f18 /// LOC100044439 | NM_024444 /// XM_001472253 | cytochrome P450, family 4, subfamily f, polypeptide 18 /// similar to cytochrome | 3.67 | CNTF up vs PBS |
| Lactb2 | NM_145381 | lactamase, beta 2 | 3.66 | CNTF up vs PBS |
| D14Ertd668e | NM_001164323 /// NM_001164324 /// NM_199015 | DNA segment, Chr 14, ERATO Doi 668, expressed | 3.65 | CNTF up vs PBS |
| 0610037M15Rik | XM_903697 | RIKEN cDNA 0610037M15 gene | 3.64 | CNTF up vs PBS |
| Zwint | NM_025635 | ZW10 interactor | 3.64 | CNTF up vs PBS |
| Muc1 | NM_013605 | mucin 1, transmembrane | 3.63 | CNTF up vs PBS |
| H2-DMb2 | NM_010388 | histocompatibility 2, class II, locus Mb2 | 3.63 | CNTF up vs PBS |
| Nuf2 | NM_023284 | NUF2, NDC80 kinetochore complex component, homolog (S. cerevisiae) | 3.62 | CNTF up vs PBS |
| H2-DMb2 | NM_010388 | histocompatibility 2, class II, locus Mb2 | 3.62 | CNTF up vs PBS |
| H2-Q7 | NM_010394 | histocompatibility 2, Q region locus 7 | 3.62 | CNTF up vs PBS |
| Zc3hav1 | NM_028421 /// NM_028864 | zinc finger CCCH type, antiviral 1 | 3.62 | CNTF up vs PBS |
| Parp10 | NM_001163575 /// NM_001163576 /// XM_001476518 /// XM_001476537 /// XM_001477922 | poly (ADP-ribose) polymerase family, member 10 | 3.62 | CNTF up vs PBS |
| Sqrdl | NM_001162503 /// NM_021507 /// NR_027888 | sulfide quinone reductase-like (yeast) | 3.62 | CNTF up vs PBS |
| Plek | NM_019549 | pleckstrin | 3.62 | CNTF up vs PBS |
| Cd72 | NM_001110320 /// NM_001110321 /// NM_001110322 /// NM_007654 | CD72 antigen | 3.61 | CNTF up vs PBS |
| Socs3 | NM_007707 | suppressor of cytokine signaling 3 | 3.61 | CNTF up vs PBS |
| Serping1 | NM_009776 | serine (or cysteine) peptidase inhibitor, clade G, member 1 | 3.61 | CNTF up vs PBS |
| Ptgfr | NM_008966 | prostaglandin F receptor | 3.59 | CNTF up vs PBS |
| 1200003I10Rik /// 1200015M12Rik /// 1200016E24Rik /// A130040M12Rik /// E430024C06Rik | NR_002860 /// XM_001472371 /// XM_001475766 | RIKEN cDNA 1200003I10 gene /// RIKEN cDNA 1200015M12 gene /// RIKEN cDNA 1200016 | 3.59 | CNTF up vs PBS |
| Csf1r | NM_001037859 | colony stimulating factor 1 receptor | 3.59 | CNTF up vs PBS |
| Birc3 | NM_007464 | baculoviral IAP repeat-containing 3 | 3.58 | CNTF up vs PBS |
| Cd22 | NM_001043317 /// NM_009845 | CD22 antigen | 3.58 | CNTF up vs PBS |
| Tmem106a | NM_144830 | transmembrane protein 106A | 3.55 | CNTF up vs PBS |
| 0610037M15Rik /// H2-gs10 | NM_001143689 /// XM_903697 /// XR_032175 /// XR_032223 /// XR_034205 | RIKEN cDNA 0610037M15 gene /// MHC class I like protein GS10 | 3.55 | CNTF up vs PBS |
| 4933413G19Rik /// Foxm1 /// Pebp1 | NM_008021 /// NM_018858 /// NM_027697 | RIKEN cDNA 4933413G19 gene /// forkhead box M1 /// phosphatidylethanolamine bind | 3.55 | CNTF up vs PBS |
| Parp3 | NM_145619 | poly (ADP-ribose) polymerase family, member 3 | 3.55 | CNTF up vs PBS |
| Slfn8 | NM_181545 | schlafen 8 | 3.54 | CNTF up vs PBS |
| 9530028C05 | --- | hypothetical protein 9530028C05 | 3.54 | CNTF up vs PBS |
| Fosl2 | NM_008037 | fos-like antigen 2 | 3.54 | CNTF up vs PBS |
| Mrc1 | NM_008625 | mannose receptor, C type 1 | 3.53 | CNTF up vs PBS |
| Nfkbiz | NM_001159394 /// NM_001159395 /// NM_030612 | nuclear factor of kappa light polypeptide gene enhancer in B-cells inhibitor, ze | 3.53 | CNTF up vs PBS |
| Osmr | NM_011019 | oncostatin M receptor | 3.53 | CNTF up vs PBS |
| Nfkbia | NM_010907 | nuclear factor of kappa light polypeptide gene enhancer in B-cells inhibitor, al | 3.53 | CNTF up vs PBS |
| Apob48r | NM_138310 | apolipoprotein B48 receptor | 3.52 | CNTF up vs PBS |
| Cd180 | NM_008533 | CD180 antigen | 3.52 | CNTF up vs PBS |
| S100a6 | NM_011313 | S100 calcium binding protein A6 (calcyclin) | 3.52 | CNTF up vs PBS |
| Samd9l | NM_010156 /// XM_620286 /// XM_908922 | sterile alpha motif domain containing 9-like | 3.51 | CNTF up vs PBS |
| Egr3 | NM_018781 | early growth response 3 | 3.51 | CNTF up vs PBS |
| Cotl1 | NM_028071 | coactosin-like 1 (Dictyostelium) | 3.51 | CNTF up vs PBS |
| Matn2 | NM_016762 | matrilin 2 | 3.50 | CNTF up vs PBS |
| Gsn | NM_146120 | gelsolin | 3.50 | CNTF up vs PBS |
| Fli1 | NM_008026 | Friend leukemia integration 1 | 3.50 | CNTF up vs PBS |
| Uhrf1 | NM_001111078 /// NM_001111079 /// NM_001111080 /// NM_010931 | ubiquitin-like, containing PHD and RING finger domains, 1 | 3.48 | CNTF up vs PBS |
| Lyl1 | NM_008535 /// XM_001473917 | lymphoblastomic leukemia 1 | 3.47 | CNTF up vs PBS |
| Gpnmb | NM_053110 | glycoprotein (transmembrane) nmb | 3.47 | CNTF up vs PBS |
| Gmfg | NM_001039192 /// NM_022024 | glia maturation factor, gamma | 3.46 | CNTF up vs PBS |
| Eif4ebp1 | NM_007918 | eukaryotic translation initiation factor 4E binding protein 1 | 3.45 | CNTF up vs PBS |
| Parp14 | NM_001039530 | poly (ADP-ribose) polymerase family, member 14 | 3.45 | CNTF up vs PBS |
| Ptpn6 | NM_001077705 /// NM_013545 | protein tyrosine phosphatase, non-receptor type 6 | 3.45 | CNTF up vs PBS |
| 9030625A04Rik | NM_172488 | RIKEN cDNA 9030625A04 gene | 3.44 | CNTF up vs PBS |
| Anxa3 | NM_013470 | annexin A3 | 3.44 | CNTF up vs PBS |
| Litaf | NM_019980 | LPS-induced TN factor | 3.43 | CNTF up vs PBS |
| Parp3 | NM_145619 | poly (ADP-ribose) polymerase family, member 3 | 3.42 | CNTF up vs PBS |
| Rnase4 | NM_021472 /// NM_201239 | ribonuclease, RNase A family 4 | 3.42 | CNTF up vs PBS |
| Socs3 | NM_007707 | suppressor of cytokine signaling 3 | 3.40 | CNTF up vs PBS |
| Tifab | NM_145976 | TRAF-interacting protein with forkhead-associated domain, family member B | 3.39 | CNTF up vs PBS |
| Rhog | NM_019566 | ras homolog gene family, member G | 3.39 | CNTF up vs PBS |
| 5430435G22Rik | NM_145509 | RIKEN cDNA 5430435G22 gene | 3.37 | CNTF up vs PBS |
| Nckap1l | NM_153505 | NCK associated protein 1 like | 3.37 | CNTF up vs PBS |
| Tnfaip2 | NM_009396 | tumor necrosis factor, alpha-induced protein 2 | 3.37 | CNTF up vs PBS |
| Chi3l1 | NM_007695 | chitinase 3-like 1 | 3.37 | CNTF up vs PBS |
| Pole | NM_011132 | polymerase (DNA directed), epsilon | 3.36 | CNTF up vs PBS |
| Gpr183 | NM_183031 | G protein-coupled receptor 183 | 3.35 | CNTF up vs PBS |
| Ifih1 | NM_001164477 /// NM_027835 | interferon induced with helicase C domain 1 | 3.35 | CNTF up vs PBS |
| Stat1 | NM_009283 | signal transducer and activator of transcription 1 | 3.34 | CNTF up vs PBS |
| Fcgr1 | NM_010186 | Fc receptor, IgG, high affinity I | 3.34 | CNTF up vs PBS |
| Tbxas1 | NM_011539 | thromboxane A synthase 1, platelet | 3.34 | CNTF up vs PBS |
| Ifitm3 | NM_025378 | interferon induced transmembrane protein 3 | 3.34 | CNTF up vs PBS |
| Map3k8 | NM_007746 | mitogen-activated protein kinase kinase kinase 8 | 3.34 | CNTF up vs PBS |
| Cep55 | NM_001164362 /// NM_028293 /// NM_028760 | centrosomal protein 55 | 3.34 | CNTF up vs PBS |
| Soat1 | NM_009230 | sterol O-acyltransferase 1 | 3.33 | CNTF up vs PBS |
| Gm9706 /// Isg15 | NM_015783 /// XM_001471685 /// XM_001471686 /// XR_005074 /// XR_034969 | predicted gene 9706 /// ISG15 ubiquitin-like modifier | 3.33 | CNTF up vs PBS |
| Slc37a2 | NM_001145960 /// NM_020258 | solute carrier family 37 (glycerol-3-phosphate transporter), member 2 | 3.33 | CNTF up vs PBS |
| Nek6 | NM_001159631 /// NM_021606 | NIMA (never in mitosis gene a)-related expressed kinase 6 | 3.32 | CNTF up vs PBS |
| Ahnak2 | NM_001033476 /// XM_001475946 | AHNAK nucleoprotein 2 | 3.32 | CNTF up vs PBS |
| Rpl7 | NM_011291 | Ribosomal protein L7 | 3.32 | CNTF up vs PBS |
| Gch1 | NM_008102 | GTP cyclohydrolase 1 | 3.31 | CNTF up vs PBS |
| Psmb8 | NM_010724 | proteasome (prosome, macropain) subunit, beta type 8 (large multifunctional pept | 3.31 | CNTF up vs PBS |
| Glipr1 | NM_028608 | GLI pathogenesis-related 1 (glioma) | 3.31 | CNTF up vs PBS |
| Syngr2 | NM_009304 | synaptogyrin 2 | 3.30 | CNTF up vs PBS |
| Psmb9 | NM_013585 | proteasome (prosome, macropain) subunit, beta type 9 (large multifunctional pept | 3.30 | CNTF up vs PBS |
| Gbp6 | NM_001083312 /// NM_145545 | guanylate binding protein 6 | 3.30 | CNTF up vs PBS |
| H2-T10 /// H2-T17 /// H2-T22 /// H2-T9 | NM_010395 /// NM_010397 /// NM_010399 | histocompatibility 2, T region locus 10 /// histocompatibility 2, T region locus | 3.30 | CNTF up vs PBS |
| 4930579C15Rik | NM_027089 | RIKEN cDNA 4930579C15 gene | 3.30 | CNTF up vs PBS |
| Laptm5 | NM_010686 | lysosomal-associated protein transmembrane 5 | 3.29 | CNTF up vs PBS |
| Fes | NM_010194 | feline sarcoma oncogene | 3.29 | CNTF up vs PBS |
| Msr1 | NM_001113326 /// NM_031195 | macrophage scavenger receptor 1 | 3.29 | CNTF up vs PBS |
| Lgals9 | NM_001159301 /// NM_010708 | lectin, galactose binding, soluble 9 | 3.28 | CNTF up vs PBS |
| Plin2 | NM_007408 | perilipin 2 | 3.28 | CNTF up vs PBS |
| Nfkbia | NM_010907 | nuclear factor of kappa light polypeptide gene enhancer in B-cells inhibitor, al | 3.28 | CNTF up vs PBS |
| Nfkbiz | NM_001159394 /// NM_001159395 /// NM_030612 | nuclear factor of kappa light polypeptide gene enhancer in B-cells inhibitor, ze | 3.27 | CNTF up vs PBS |
| Oasl2 | NM_011854 | 2'-5' oligoadenylate synthetase-like 2 | 3.27 | CNTF up vs PBS |
| Pim1 | NM_008842 | Proviral integration site 1 | 3.27 | CNTF up vs PBS |
| Pik3r5 | NM_177320 | phosphoinositide-3-kinase, regulatory subunit 5, p101 | 3.27 | CNTF up vs PBS |
| Trim34 | NM_030684 | tripartite motif-containing 34 | 3.26 | CNTF up vs PBS |
| Vav1 | NM_001163815 /// NM_001163816 /// NM_011691 | Vav 1 oncogene | 3.26 | CNTF up vs PBS |
| Cmtm6 | NM_026036 | CKLF-like MARVEL transmembrane domain containing 6 | 3.25 | CNTF up vs PBS |
| Klf4 | NM_010637 | Kruppel-like factor 4 (gut) | 3.25 | CNTF up vs PBS |
| Mvp | NM_080638 | major vault protein | 3.24 | CNTF up vs PBS |
| Gsn | NM_146120 | gelsolin | 3.23 | CNTF up vs PBS |
| Pim1 | NM_008842 | proviral integration site 1 | 3.23 | CNTF up vs PBS |
| 2610300M13Rik | --- | RIKEN cDNA 2610300M13 gene | 3.23 | CNTF up vs PBS |
| LOC676654 /// Lyn | NM_001111096 /// NM_010747 /// XM_991890 | similar to Yamaguchi sarcoma viral (v-yes-1) oncogene homolog /// Yamaguchi sarc | 3.22 | CNTF up vs PBS |
| Nlrp3 | NM_145827 | NLR family, pyrin domain containing 3 | 3.22 | CNTF up vs PBS |
| Nek6 | NM_001159631 /// NM_021606 | NIMA (never in mitosis gene a)-related expressed kinase 6 | 3.22 | CNTF up vs PBS |
| Kctd12 | NM_177715 | potassium channel tetramerisation domain containing 12 | 3.21 | CNTF up vs PBS |
| Cd93 | NM_010740 | CD93 antigen | 3.19 | CNTF up vs PBS |
| Tmem106a | NM_144830 | transmembrane protein 106A | 3.19 | CNTF up vs PBS |
| Lrrc33 | NM_146069 | leucine rich repeat containing 33 | 3.18 | CNTF up vs PBS |
| 2310008H04Rik | NM_146068 | RIKEN cDNA 2310008H04 gene | 3.18 | CNTF up vs PBS |
| Col4a1 | NM_009931 | collagen, type IV, alpha 1 | 3.17 | CNTF up vs PBS |
| Hspa1b | NM_010478 | heat shock protein 1B | 3.17 | CNTF up vs PBS |
| Bmp2 | NM_007553 | bone morphogenetic protein 2 | 3.17 | CNTF up vs PBS |
| Cyba | NM_007806 | cytochrome b-245, alpha polypeptide | 3.17 | CNTF up vs PBS |
| Fam129a | NM_022018 | family with sequence similarity 129, member A | 3.17 | CNTF up vs PBS |
| Mx2 | NM_013606 /// NR_003508 /// XR_035745 | myxovirus (influenza virus) resistance 2 | 3.15 | CNTF up vs PBS |
| Cnn2 | NM_007725 | calponin 2 | 3.14 | CNTF up vs PBS |
| Egln3 | NM_028133 | EGL nine homolog 3 (C. elegans) | 3.14 | CNTF up vs PBS |
| Krt80 | NM_028770 | keratin 80 | 3.14 | CNTF up vs PBS |
| Irf1 | NM_001159393 /// NM_001159396 /// NM_008390 | interferon regulatory factor 1 | 3.14 | CNTF up vs PBS |
| Fst | NM_008046 | follistatin | 3.13 | CNTF up vs PBS |
| Shisa5 | NM_025858 /// NM_026381 | shisa homolog 5 (Xenopus laevis) | 3.12 | CNTF up vs PBS |
| Csf1 | NM_001113529 /// NM_001113530 /// NM_007778 | colony stimulating factor 1 (macrophage) | 3.12 | CNTF up vs PBS |
| Lamp2 | NM_001017959 /// NM_010685 | lysosomal-associated membrane protein 2 | 3.11 | CNTF up vs PBS |
| Ifitm2 | NM_030694 | interferon induced transmembrane protein 2 | 3.11 | CNTF up vs PBS |
| Socs3 | NM_007707 | suppressor of cytokine signaling 3 | 3.10 | CNTF up vs PBS |
| Capg | NM_001042534 /// NM_007599 | capping protein (actin filament), gelsolin-like | 3.09 | CNTF up vs PBS |
| Gm6648 | XM_890619 /// XM_916418 | Predicted gene 6648 | 3.08 | CNTF up vs PBS |
| Baz1a | NM_013815 /// XM_885873 /// XM_915565 | bromodomain adjacent to zinc finger domain 1A | 3.08 | CNTF up vs PBS |
| Gpr84 | NM_030720 | G protein-coupled receptor 84 | 3.08 | CNTF up vs PBS |
| Il13ra1 | NM_133990 | interleukin 13 receptor, alpha 1 | 3.07 | CNTF up vs PBS |
| Adam8 | NM_007403 | a disintegrin and metallopeptidase domain 8 | 3.07 | CNTF up vs PBS |
| Treml4 | NM_001033922 /// NM_001163795 /// NM_001163796 /// NM_172623 | triggering receptor expressed on myeloid cells-like 4 | 3.07 | CNTF up vs PBS |
| Ctsz | NM_022325 | cathepsin Z | 3.07 | CNTF up vs PBS |
| Il6ra | NM_010559 | interleukin 6 receptor, alpha | 3.07 | CNTF up vs PBS |
| Apobec3 | NM_001160415 /// NM_030255 | apolipoprotein B mRNA editing enzyme, catalytic polypeptide 3 | 3.06 | CNTF up vs PBS |
| Gcnt1 | NM_001136484 /// NM_010265 /// NM_173442 | glucosaminyl (N-acetyl) transferase 1, core 2 | 3.06 | CNTF up vs PBS |
| 3830403N18Rik /// Xlr | NM_011725 /// NM_027510 | RIKEN cDNA 3830403N18 gene /// X-linked lymphocyte-regulated complex | 3.06 | CNTF up vs PBS |
| Pvt1 | NR_003368 | plasmacytoma variant translocation 1 | 3.05 | CNTF up vs PBS |
| Fam46c | NM_001142952 /// XR_001536 /// XR_002338 /// XR_005163 | family with sequence similarity 46, member C | 3.05 | CNTF up vs PBS |
| Ctsz | NM_022325 | cathepsin Z | 3.05 | CNTF up vs PBS |
| H2-K1 | NM_001001892 | histocompatibility 2, K1, K region | 3.05 | CNTF up vs PBS |
| Tlr7 | NM_133211 | toll-like receptor 7 | 3.04 | CNTF up vs PBS |
| Itga5 | NM_010577 | integrin alpha 5 (fibronectin receptor alpha) | 3.02 | CNTF up vs PBS |
| 1810033B17Rik | NM_026985 | RIKEN cDNA 1810033B17 gene | 3.02 | CNTF up vs PBS |
| Klf6 | NM_011803 | Kruppel-like factor 6 | 3.02 | CNTF up vs PBS |
| Ets1 | NM_001038642 /// NM_011808 | E26 avian leukemia oncogene 1, 5' domain | 3.02 | CNTF up vs PBS |
| Nfam1 | NM_028728 | Nfat activating molecule with ITAM motif 1 | 3.01 | CNTF up vs PBS |
| Sdpr | NM_138741 | serum deprivation response | 3.01 | CNTF up vs PBS |
| Ecm1 | NM_007899 | extracellular matrix protein 1 | 3.01 | CNTF up vs PBS |
| Wwtr1 | NM_133784 | WW domain containing transcription regulator 1 | 3.00 | CNTF up vs PBS |
| Tpx2 | NM_001141975 /// NM_001141976 /// NM_001141977 /// NM_001141978 /// NM_028109 | TPX2, microtubule-associated protein homolog (Xenopus laevis) | 2.99 | CNTF up vs PBS |
| Nr4a1 | NM_010444 | nuclear receptor subfamily 4, group A, member 1 | 2.99 | CNTF up vs PBS |
| Gsn | NM_146120 | gelsolin | 2.99 | CNTF up vs PBS |
| Cd109 | NM_153098 | CD109 antigen | 2.98 | CNTF up vs PBS |
| A630001G21Rik | NM_177055 /// XM_484889 /// XM_895419 /// XM_905026 /// XM_919139 | RIKEN cDNA A630001G21 gene | 2.98 | CNTF up vs PBS |
| Lpxn | NM_134152 | leupaxin | 2.98 | CNTF up vs PBS |
| B2m | NM_009735 | beta-2 microglobulin | 2.98 | CNTF up vs PBS |
| Ctsz | NM_022325 | cathepsin Z | 2.98 | CNTF up vs PBS |
| Erap1 | NM_030711 | endoplasmic reticulum aminopeptidase 1 | 2.97 | CNTF up vs PBS |
| H2-D1 | NM_010380 | histocompatibility 2, D region locus 1 | 2.97 | CNTF up vs PBS |
| Mvp | NM_080638 | major vault protein | 2.96 | CNTF up vs PBS |
| Ifi30 | NM_023065 | interferon gamma inducible protein 30 | 2.96 | CNTF up vs PBS |
| Cd300lf /// LOC100047115 | NM_145634 /// XR_033645 | CD300 antigen like family member F /// similar to CD300 antigen like family memb | 2.96 | CNTF up vs PBS |
| Gadd45b | NM_008655 | Growth arrest and DNA-damage-inducible 45 beta | 2.96 | CNTF up vs PBS |
| Sft2d2 | NM_145512 | SFT2 domain containing 2 | 2.95 | CNTF up vs PBS |
| Gypc | NM_001048207 | glycophorin C | 2.95 | CNTF up vs PBS |
| Arc | NM_018790 | activity regulated cytoskeletal-associated protein | 2.94 | CNTF up vs PBS |
| Dtx3l | NM_001013371 | deltex 3-like (Drosophila) | 2.94 | CNTF up vs PBS |
| Ppih | NM_001110129 /// NM_001110130 /// NM_028677 | peptidyl prolyl isomerase H | 2.93 | CNTF up vs PBS |
| Dock10 | NM_175291 /// XM_001472159 /// XM_891407 | dedicator of cytokinesis 10 | 2.93 | CNTF up vs PBS |
| Cd37 | NM_007645 | CD37 antigen | 2.93 | CNTF up vs PBS |
| Ier3 | NM_133662 | immediate early response 3 | 2.92 | CNTF up vs PBS |
| Ear2 | NM_007895 | eosinophil-associated, ribonuclease A family, member 2 | 2.92 | CNTF up vs PBS |
| Lcp2 | NM_010696 | lymphocyte cytosolic protein 2 | 2.92 | CNTF up vs PBS |
| Siglece | NM_031181 | sialic acid binding Ig-like lectin E | 2.92 | CNTF up vs PBS |
| Abi3 | NM_001163464 /// NM_025659 | ABI gene family, member 3 | 2.91 | CNTF up vs PBS |
| Rac2 | NM_009008 | RAS-related C3 botulinum substrate 2 | 2.91 | CNTF up vs PBS |
| Ang | NM_001161731 /// NM_007447 | angiogenin, ribonuclease, RNase A family, 5 | 2.90 | CNTF up vs PBS |
| Tlr7 | NM_133211 | toll-like receptor 7 | 2.90 | CNTF up vs PBS |
| AW112010 | XM_888885 /// XM_908564 | expressed sequence AW112010 | 2.90 | CNTF up vs PBS |
| Gbp2 | NM_010260 | guanylate binding protein 2 | 2.90 | CNTF up vs PBS |
| Ecscr | NM_001033141 | endothelial cell-specific chemotaxis regulator | 2.89 | CNTF up vs PBS |
| Saa1 | NM_009117 | serum amyloid A 1 | 2.89 | CNTF up vs PBS |
| Sdad1 | NM_172713 | SDA1 domain containing 1 | 2.89 | CNTF up vs PBS |
| Zc3hav1 | NM_028421 /// NM_028864 | zinc finger CCCH type, antiviral 1 | 2.89 | CNTF up vs PBS |
| H2-L | --- | histocompatibility 2, D region | 2.88 | CNTF up vs PBS |
| Syk | NM_011518 | spleen tyrosine kinase | 2.88 | CNTF up vs PBS |
| Birc3 | NM_007464 | baculoviral IAP repeat-containing 3 | 2.88 | CNTF up vs PBS |
| Cldn9 | NM_020293 | claudin 9 | 2.88 | CNTF up vs PBS |
| Tnfrsf1a | NM_011609 | tumor necrosis factor receptor superfamily, member 1a | 2.88 | CNTF up vs PBS |
| Ctsc | NM_009982 | cathepsin C | 2.87 | CNTF up vs PBS |
| Gadd45b | NM_008655 | growth arrest and DNA-damage-inducible 45 beta | 2.87 | CNTF up vs PBS |
| Mrpl15 | NM_025300 | mitochondrial ribosomal protein L15 | 2.87 | CNTF up vs PBS |
| Mx1 | NM_010846 /// NR_003520 /// XR_035486 | myxovirus (influenza virus) resistance 1 | 2.87 | CNTF up vs PBS |
| Shisa5 | NM_025858 /// NM_026381 | shisa homolog 5 (Xenopus laevis) | 2.87 | CNTF up vs PBS |
| Wwtr1 | NM_133784 | WW domain containing transcription regulator 1 | 2.85 | CNTF up vs PBS |
| Arhgap30 | NM_001005508 | Rho GTPase activating protein 30 | 2.84 | CNTF up vs PBS |
| Cyth4 | NM_028195 | cytohesin 4 | 2.84 | CNTF up vs PBS |
| Fosb | NM_008036 | FBJ osteosarcoma oncogene B | 2.83 | CNTF up vs PBS |
| Pld1 | NM_001164056 /// NM_008875 | phospholipase D1 | 2.83 | CNTF up vs PBS |
| Herc5 | NM_025992 /// XM_001478484 /// XM_001478497 /// XM_907983 | hect domain and RLD 5 | 2.82 | CNTF up vs PBS |
| Oas3 | NM_145226 | 2'-5' oligoadenylate synthetase 3 | 2.82 | CNTF up vs PBS |
| Diap2 | NM_017398 /// NM_172493 | diaphanous homolog 2 (Drosophila) | 2.82 | CNTF up vs PBS |
| Pik3cd | NM_001029837 /// NM_001164049 /// NM_001164050 /// NM_001164051 /// NM_001164052 | phosphatidylinositol 3-kinase catalytic delta polypeptide | 2.82 | CNTF up vs PBS |
| Ctss | NM_021281 | cathepsin S | 2.81 | CNTF up vs PBS |
| Nfkbid | NM_172142 | nuclear factor of kappa light polypeptide gene enhancer in B-cells inhibitor, de | 2.81 | CNTF up vs PBS |
| Il4ra | NM_001008700 | interleukin 4 receptor, alpha | 2.80 | CNTF up vs PBS |
| Fam129a | NM_022018 | family with sequence similarity 129, member A | 2.80 | CNTF up vs PBS |
| Seh1l | NM_001039088 /// NM_028112 | SEH1-like (S. cerevisiae | 2.80 | CNTF up vs PBS |
| Elk3 | NM_013508 /// NM_205536 | ELK3, member of ETS oncogene family | 2.80 | CNTF up vs PBS |
| Klf6 | NM_011803 | Kruppel-like factor 6 | 2.79 | CNTF up vs PBS |
| Abcc4 | NM_001033336 /// NM_001163675 /// NM_001163676 | ATP-binding cassette, sub-family C (CFTR/MRP), member 4 | 2.79 | CNTF up vs PBS |
| Nfkbia | NM_010907 | nuclear factor of kappa light polypeptide gene enhancer in B-cells inhibitor, al | 2.79 | CNTF up vs PBS |
| Nipsnap3a | NM_025623 | nipsnap homolog 3A (C. elegans) | 2.79 | CNTF up vs PBS |
| Ddx58 | NM_172689 | DEAD (Asp-Glu-Ala-Asp) box polypeptide 58 | 2.78 | CNTF up vs PBS |
| Ttc39b | NM_027238 | tetratricopeptide repeat domain 39B | 2.78 | CNTF up vs PBS |
| Zbp1 | NM_001139519 /// NM_021394 | Z-DNA binding protein 1 | 2.78 | CNTF up vs PBS |
| Arhgdib | NM_007486 | Rho, GDP dissociation inhibitor (GDI) beta | 2.77 | CNTF up vs PBS |
| Gm7669 /// Gm7901 /// Gm9347 /// Phgdh | NM_016966 /// XR_030810 /// XR_030923 /// XR_031967 /// XR_032429 /// XR_034648 | predicted gene 7669 /// 3-phosphoglycerate dehydrogenase pseudogene /// predicte | 2.77 | CNTF up vs PBS |
| Hn1l | NM_198937 | hematological and neurological expressed 1-like | 2.77 | CNTF up vs PBS |
| Angptl2 | NM_011923 | angiopoietin-like 2 | 2.77 | CNTF up vs PBS |
| B2m | NM_009735 | beta-2 microglobulin | 2.76 | CNTF up vs PBS |
| Rnf213 | NM_001040005 /// XM_001476651 /// XM_001477846 | ring finger protein 213 | 2.76 | CNTF up vs PBS |
| Elk3 | NM_013508 /// NM_205536 | ELK3, member of ETS oncogene family | 2.76 | CNTF up vs PBS |
| H2-Eb1 | NM_010382 | histocompatibility 2, class II antigen E beta | 2.75 | CNTF up vs PBS |
| Man1a | NM_008548 | mannosidase 1, alpha | 2.74 | CNTF up vs PBS |
| Bcl2a1a /// Bcl2a1b /// Bcl2a1d | NM_007534 /// NM_007536 /// NM_009742 | B-cell leukemia/lymphoma 2 related protein A1a /// B-cell leukemia/lymphoma 2 re | 2.74 | CNTF up vs PBS |
| Cebpb | NM_009883 | CCAAT/enhancer binding protein (C/EBP), beta | 2.74 | CNTF up vs PBS |
| Syngr2 | NM_009304 | synaptogyrin 2 | 2.74 | CNTF up vs PBS |
| Il2rg | NM_013563 | interleukin 2 receptor, gamma chain | 2.74 | CNTF up vs PBS |
| Phlda1 | NM_009344 | pleckstrin homology-like domain, family A, member 1 | 2.74 | CNTF up vs PBS |
| Cds2 | NM_138651 | CDP-diacylglycerol synthase (phosphatidate cytidylyltransferase) 2 | 2.74 | CNTF up vs PBS |
| 9530048O09Rik | NR_024078 /// XM_001477060 /// XR_035186 /// XR_035205 /// XR_035280 | RIKEN cDNA 9530048O09 gene | 2.73 | CNTF up vs PBS |
| Arhgap22 | NM_153800 | Rho GTPase activating protein 22 | 2.72 | CNTF up vs PBS |
| Abcb1b | NM_011075 | ATP-binding cassette, sub-family B (MDR/TAP), member 1B | 2.71 | CNTF up vs PBS |
| Nfkb2 | NM_019408 | nuclear factor of kappa light polypeptide gene enhancer in B-cells 2, p49/p100 | 2.71 | CNTF up vs PBS |
| Kcnn4 | NM_001163510 /// NM_008433 | potassium intermediate/small conductance calcium-activated channel, subfamily N, | 2.71 | CNTF up vs PBS |
| Ostf1 | NM_017375 | osteoclast stimulating factor 1 | 2.71 | CNTF up vs PBS |
| Cotl1 | NM_028071 | coactosin-like 1 (Dictyostelium) | 2.71 | CNTF up vs PBS |
| Enpp1 | NM_008813 | ectonucleotide pyrophosphatase/phosphodiesterase 1 | 2.70 | CNTF up vs PBS |
| Fermt3 | NM_153795 | fermitin family homolog 3 (Drosophila) | 2.69 | CNTF up vs PBS |
| Csf2rb | NM_007780 | colony stimulating factor 2 receptor, beta, low-affinity (granulocyte-macrophage | 2.69 | CNTF up vs PBS |
| Slc10a6 | NM_029415 | solute carrier family 10 (sodium/bile acid cotransporter family), member 6 | 2.69 | CNTF up vs PBS |
| H2-D1 | NM_010380 | histocompatibility 2, D region locus 1 | 2.68 | CNTF up vs PBS |
| Osgin1 | NM_027950 | oxidative stress induced growth inhibitor 1 | 2.68 | CNTF up vs PBS |
| 1700017B05Rik | NM_028820 | RIKEN cDNA 1700017B05 gene | 2.67 | CNTF up vs PBS |
| Pvt1 | NR_003368 | plasmacytoma variant translocation 1 | 2.67 | CNTF up vs PBS |
| C79407 | NM_172578 | expressed sequence C79407 | 2.67 | CNTF up vs PBS |
| Oas1b | NM_001083925 /// NR_003507 /// XM_001471672 /// XM_001471673 | 2'-5' oligoadenylate synthetase 1B | 2.66 | CNTF up vs PBS |
| B3gnt5 | NM_001159407 /// NM_001159408 /// NM_054052 | UDP-GlcNAc:betaGal beta-1,3-N-acetylglucosaminyltransferase 5 | 2.66 | CNTF up vs PBS |
| Gpr65 | NM_008152 | G-protein coupled receptor 65 | 2.66 | CNTF up vs PBS |
| EG627427 /// Gm13337 /// Gm5385 /// Gm7669 /// Gm7901 /// Gm8096 /// Gm8341 /// Gm9210 /// Gm9252 /// Gm9347 /// LOC630761 /// LOC630896 /// Phgdh | NM_016966 /// XR_001608 /// XR_001871 /// XR_001935 /// XR_030502 /// XR_030672 | predicted gene, EG627427 /// predicted gene 13337 /// predicted gene 5385 /// pr | 2.66 | CNTF up vs PBS |
| Entpd1 | NM_009848 | ectonucleoside triphosphate diphosphohydrolase 1 | 2.66 | CNTF up vs PBS |
| Tmem123 | NM_133739 | transmembrane protein 123 | 2.66 | CNTF up vs PBS |
| Gsn | NM_146120 | gelsolin | 2.65 | CNTF up vs PBS |
| A430104N18Rik | --- | RIKEN cDNA A430104N18 gene | 2.65 | CNTF up vs PBS |
| Adap2 | NM_172133 | ArfGAP with dual PH domains 2 | 2.65 | CNTF up vs PBS |
| Itpripl2 | NM_001033380 | inositol 1,4,5-triphosphate receptor interacting protein-like 2 | 2.64 | CNTF up vs PBS |
| C3ar1 | NM_009779 | complement component 3a receptor 1 | 2.64 | CNTF up vs PBS |
| Aspg | NM_001081169 | asparaginase homolog (S. cerevisiae) | 2.64 | CNTF up vs PBS |
| Col5a3 | NM_016919 | collagen, type V, alpha 3 | 2.64 | CNTF up vs PBS |
| Slc16a10 | NM_001114332 /// NM_028247 | solute carrier family 16 (monocarboxylic acid transporters), member 10 | 2.63 | CNTF up vs PBS |
| Raet1a /// Raet1b /// Raet1c /// Raet1d /// Raet1e | NM_009016 /// NM_009017 /// NM_009018 /// NM_020030 /// NM_198193 /// XM_0010062 | retinoic acid early transcript 1, alpha /// retinoic acid early transcript beta | 2.63 | CNTF up vs PBS |
| Lcn2 | NM_008491 | lipocalin 2 | 2.63 | CNTF up vs PBS |
| Gm5547 | XM_489019 /// XM_992555 | predicted gene 5547 | 2.62 | CNTF up vs PBS |
| Csf1r | NM_001037859 | colony stimulating factor 1 receptor | 2.62 | CNTF up vs PBS |
| 2310014H01Rik | NM_001146710 /// NM_001146711 /// NM_175242 /// XM_001003622 /// XM_001474189 | RIKEN cDNA 2310014H01 gene | 2.62 | CNTF up vs PBS |
| Stat6 | NM_009284 | signal transducer and activator of transcription 6 | 2.62 | CNTF up vs PBS |
| Lrrfip1 | NM_001111311 /// NM_001111312 /// NM_008515 | leucine rich repeat (in FLII) interacting protein 1 | 2.62 | CNTF up vs PBS |
| Hmha1 | NM_001142701 /// NM_027521 | histocompatibility (minor) HA-1 | 2.62 | CNTF up vs PBS |
| Myo1g | NM_178440 | myosin IG | 2.61 | CNTF up vs PBS |
| Pik3cg | NM_001146200 /// NM_001146201 /// NM_020272 | phosphoinositide-3-kinase, catalytic, gamma polypeptide | 2.60 | CNTF up vs PBS |
| Ehbp1l1 | NM_001114595 /// NM_001114596 /// NM_001114597 /// NM_053252 | EH domain binding protein 1-like 1 | 2.59 | CNTF up vs PBS |
| Dclk1 | NM_001111051 /// NM_001111052 /// NM_001111053 /// NM_019978 | doublecortin-like kinase 1 | 2.59 | CNTF up vs PBS |
| Slc40a1 | NM_016917 | solute carrier family 40 (iron-regulated transporter), member 1 | 2.59 | CNTF up vs PBS |
| Plk4 | NM_011495 | polo-like kinase 4 (Drosophila) | 2.59 | CNTF up vs PBS |
| Vwa5a | NM_001145957 /// NM_172767 | von Willebrand factor A domain containing 5A | 2.59 | CNTF up vs PBS |
| Nfe2l2 | NM_010902 | nuclear factor, erythroid derived 2, like 2 | 2.58 | CNTF up vs PBS |
| H2-D1 | NM_010380 | histocompatibility 2, D region locus 1 | 2.58 | CNTF up vs PBS |
| Arhgap11a | NM_181416 | Rho GTPase activating protein 11A | 2.58 | CNTF up vs PBS |
| Dock8 | NM_028785 | dedicator of cytokinesis 8 | 2.58 | CNTF up vs PBS |
| Bzw1 | NM_025824 | basic leucine zipper and W2 domains 1 | 2.57 | CNTF up vs PBS |
| Espl1 | NM_001014976 | extra spindle poles-like 1 (S. cerevisiae) | 2.57 | CNTF up vs PBS |
| Gm9861 /// Litaf | NM_019980 /// XM_619421 | predicted gene 9861 /// LPS-induced TN factor | 2.57 | CNTF up vs PBS |
| Metrnl | NM_144797 | meteorin, glial cell differentiation regulator-like | 2.57 | CNTF up vs PBS |
| Ripk3 | NM_001164107 /// NM_001164108 /// NM_019955 | receptor-interacting serine-threonine kinase 3 | 2.57 | CNTF up vs PBS |
| Tpm2 | NM_009416 | tropomyosin 2, beta | 2.57 | CNTF up vs PBS |
| 5430435G22Rik | NM_145509 | RIKEN cDNA 5430435G22 gene | 2.56 | CNTF up vs PBS |
| Csn3 | NM_007786 | casein kappa | 2.56 | CNTF up vs PBS |
| Eif2ak2 | NM_011163 | eukaryotic translation initiation factor 2-alpha kinase 2 | 2.56 | CNTF up vs PBS |
| Fgd5 | NM_172731 | FYVE, RhoGEF and PH domain containing 5 | 2.55 | CNTF up vs PBS |
| Tgfa | NM_031199 | transforming growth factor alpha | 2.55 | CNTF up vs PBS |
| Esyt1 | NM_011843 | extended synaptotagmin-like protein 1 | 2.54 | CNTF up vs PBS |
| Gcnt2 | NM_008105 /// NM_023887 /// NM_133219 | glucosaminyl (N-acetyl) transferase 2, I-branching enzyme | 2.54 | CNTF up vs PBS |
| LOC100038746 | --- | hypothetical LOC100038746 | 2.54 | CNTF up vs PBS |
| Lxn | NM_016753 | latexin | 2.54 | CNTF up vs PBS |
| Wdfy4 | NM_001146022 /// XM_619220 | WD repeat and FYVE domain containing 4 | 2.53 | CNTF up vs PBS |
| Bzw1 | NM_025824 | basic leucine zipper and W2 domains 1 | 2.53 | CNTF up vs PBS |
| Ulk2 | NM_013881 | Unc-51 like kinase 2 (C. elegans) | 2.53 | CNTF up vs PBS |
| Igf2bp2 | NM_183029 | insulin-like growth factor 2 mRNA binding protein 2 | 2.53 | CNTF up vs PBS |
| Il1rl1 | NM_001025602 /// NM_010743 | interleukin 1 receptor-like 1 | 2.53 | CNTF up vs PBS |
| Dnase1l1 | NM_027109 | deoxyribonuclease 1-like 1 | 2.52 | CNTF up vs PBS |
| Ucp2 | NM_011671 | uncoupling protein 2 (mitochondrial, proton carrier) | 2.52 | CNTF up vs PBS |
| Fgd4 | NM_139232 /// NM_139233 /// NM_139234 | FYVE, RhoGEF and PH domain containing 4 | 2.52 | CNTF up vs PBS |
| Nfkbia | NM_010907 | nuclear factor of kappa light polypeptide gene enhancer in B-cells inhibitor, al | 2.51 | CNTF up vs PBS |
| Zcchc6 | NM_153538 | zinc finger, CCHC domain containing 6 | 2.51 | CNTF up vs PBS |
| Hhex | NM_008245 | hematopoietically expressed homeobox | 2.51 | CNTF up vs PBS |
| Dennd1c | NM_153551 | DENN/MADD domain containing 1C | 2.51 | CNTF up vs PBS |
| Cd151 | NM_001111049 /// NM_001111050 /// NM_009842 | CD151 antigen | 2.50 | CNTF up vs PBS |
| 0610007L01Rik | NM_001081394 | RIKEN cDNA 0610007L01 gene | 2.50 | CNTF up vs PBS |
| Mapkapk3 | NM_178907 | mitogen-activated protein kinase-activated protein kinase 3 | 2.49 | CNTF up vs PBS |
| Cables1 | NM_001146287 /// NM_022021 | CDK5 and Abl enzyme substrate 1 | 2.49 | CNTF up vs PBS |
| Ccdc109b | NM_025779 | coiled-coil domain containing 109B | 2.49 | CNTF up vs PBS |
| Akap2 | NM_001035532 /// NM_001035533 /// NM_009649 | A kinase (PRKA) anchor protein 2 | 2.49 | CNTF up vs PBS |
| Gmds | NM_146041 | GDP-mannose 4, 6-dehydratase | 2.49 | CNTF up vs PBS |
| Smad5 | NM_001164041 /// NM_001164042 /// NM_008541 | MAD homolog 5 (Drosophila) | 2.49 | CNTF up vs PBS |
| LOC100045567 /// Pnp1 | NM_013632 /// XM_001474536 | similar to purine nucleoside phosphorylase /// purine-nucleoside phosphorylase 1 | 2.49 | CNTF up vs PBS |
| H2-D1 /// H2-K1 /// LOC100044874 | NM_001001892 /// NM_010380 /// XM_001473540 /// XM_001473561 /// XM_001473578 | histocompatibility 2, D region locus 1 /// histocompatibility 2, K1, K region // | 2.48 | CNTF up vs PBS |
| Arhgap9 | NM_146011 | Rho GTPase activating protein 9 | 2.48 | CNTF up vs PBS |
| Clip4 | NM_030179 /// NM_175378 | CAP-GLY domain containing linker protein family, member 4 | 2.47 | CNTF up vs PBS |
| Eif2ak2 | NM_011163 | eukaryotic translation initiation factor 2-alpha kinase 2 | 2.47 | CNTF up vs PBS |
| Htra3 | NM_001042615 /// NM_030127 | HtrA serine peptidase 3 | 2.46 | CNTF up vs PBS |
| Cklf | NM_001037841 /// NM_029295 | chemokine-like factor | 2.46 | CNTF up vs PBS |
| Stk10 | NM_009288 | serine/threonine kinase 10 | 2.45 | CNTF up vs PBS |
| Eif4e2 | NM_001039169 /// NM_001039170 /// NM_023314 | eukaryotic translation initiation factor 4E member 2 | 2.45 | CNTF up vs PBS |
| Csrnp1 | NM_153287 | cysteine-serine-rich nuclear protein 1 | 2.45 | CNTF up vs PBS |
| H2-K1 | NM_001001892 | histocompatibility 2, K1, K region | 2.44 | CNTF up vs PBS |
| Cp | NM_001042611 /// NM_007752 | ceruloplasmin | 2.44 | CNTF up vs PBS |
| Cks1b | NM_016904 | CDC28 protein kinase 1b | 2.44 | CNTF up vs PBS |
| Tmco4 | NM_029857 | transmembrane and coiled-coil domains 4 | 2.43 | CNTF up vs PBS |
| Jak3 | NM_010589 | Janus kinase 3 | 2.43 | CNTF up vs PBS |
| Il10rb | NM_008349 | interleukin 10 receptor, beta | 2.43 | CNTF up vs PBS |
| Rod1 | NM_144904 /// NM_178164 | ROD1 regulator of differentiation 1 (S. pombe) | 2.43 | CNTF up vs PBS |
| Wnt5b | NM_009525 | wingless-related MMTV integration site 5B | 2.43 | CNTF up vs PBS |
| Col1a2 | NM_007743 | collagen, type I, alpha 2 | 2.43 | CNTF up vs PBS |
| Nln | NM_029447 | neurolysin (metallopeptidase M3 family) | 2.43 | CNTF up vs PBS |
| Klf6 | NM_011803 | Kruppel-like factor 6 | 2.42 | CNTF up vs PBS |
| Hvcn1 | NM_001042489 /// NM_028752 | hydrogen voltage-gated channel 1 | 2.42 | CNTF up vs PBS |
| Col1a2 | NM_007743 | collagen, type I, alpha 2 | 2.42 | CNTF up vs PBS |
| Trim30 | NM_009099 | tripartite motif-containing 30 | 2.42 | CNTF up vs PBS |
| Iqgap1 | NM_016721 | IQ motif containing GTPase activating protein 1 | 2.41 | CNTF up vs PBS |
| Spata13 | NM_001033272 /// XM_901902 /// XM_923227 | spermatogenesis associated 13 | 2.41 | CNTF up vs PBS |
| Tbc1d1 | NM_019636 | TBC1 domain family, member 1 | 2.41 | CNTF up vs PBS |
| Ptplad2 | NM_025760 | protein tyrosine phosphatase-like A domain containing 2 | 2.41 | CNTF up vs PBS |
| Lrg1 | NM_029796 | leucine-rich alpha-2-glycoprotein 1 | 2.41 | CNTF up vs PBS |
| Stat1 | NM_009283 | signal transducer and activator of transcription 1 | 2.41 | CNTF up vs PBS |
| Fam23a | NM_001081310 | family with sequence similarity 23, member A | 2.41 | CNTF up vs PBS |
| Tirap | NM_054096 | toll-interleukin 1 receptor (TIR) domain-containing adaptor protein | 2.41 | CNTF up vs PBS |
| Tirap | NM_054096 | toll-interleukin 1 receptor (TIR) domain-containing adaptor protein | 2.41 | CNTF up vs PBS |
| Elovl1 | NM_001039175 /// NM_001039176 /// NM_019422 | elongation of very long chain fatty acids (FEN1/Elo2, SUR4/Elo3, yeast)-like 1 | 2.41 | CNTF up vs PBS |
| Ada | NM_007398 | adenosine deaminase | 2.41 | CNTF up vs PBS |
| Mthfs | NM_026829 | 5, 10-methenyltetrahydrofolate synthetase | 2.40 | CNTF up vs PBS |
| Mlkl | NM_029005 | mixed lineage kinase domain-like | 2.39 | CNTF up vs PBS |
| Pkib | NM_001039050 /// NM_001039051 /// NM_001039052 /// NM_001039053 /// NM_008863 | protein kinase inhibitor beta, cAMP dependent, testis specific | 2.39 | CNTF up vs PBS |
| Smpdl3a | NM_020561 | sphingomyelin phosphodiesterase, acid-like 3A | 2.39 | CNTF up vs PBS |
| Tec | NM_001113460 /// NM_001113461 /// NM_001113464 /// NM_013689 | tec protein tyrosine kinase | 2.39 | CNTF up vs PBS |
| Creb3l2 | NM_178661 | cAMP responsive element binding protein 3-like 2 | 2.39 | CNTF up vs PBS |
| Atl3 | NM_001163505 /// NM_146091 | atlastin GTPase 3 | 2.39 | CNTF up vs PBS |
| 1300014I06Rik | NM_025831 | RIKEN cDNA 1300014I06 gene | 2.39 | CNTF up vs PBS |
| Fxyd5 | NM_001111073 /// NM_008761 | FXYD domain-containing ion transport regulator 5 | 2.39 | CNTF up vs PBS |
| Zwint | NM_025635 | ZW10 interactor | 2.39 | CNTF up vs PBS |
| 1600010M07Rik | --- | RIKEN cDNA 1600010M07 gene | 2.38 | CNTF up vs PBS |
| Usp24 | XM_001473472 /// XM_001479517 /// XM_001481281 /// XM_915524 | ubiquitin specific peptidase 24 | 2.38 | CNTF up vs PBS |
| Gla | NM_013463 | galactosidase, alpha | 2.37 | CNTF up vs PBS |
| Ptgr1 | NM_025968 | prostaglandin reductase 1 | 2.37 | CNTF up vs PBS |
| Nfkbie | NM_008690 | nuclear factor of kappa light polypeptide gene enhancer in B-cells inhibitor, ep | 2.37 | CNTF up vs PBS |
| Arhgap4 | NM_001162423 /// NM_001162424 /// NM_138630 | Rho GTPase activating protein 4 | 2.37 | CNTF up vs PBS |
| Pign | NM_013784 | phosphatidylinositol glycan anchor biosynthesis, class N | 2.36 | CNTF up vs PBS |
| Apod | NM_007470 | apolipoprotein D | 2.36 | CNTF up vs PBS |
| Brca1 | NM_009764 | breast cancer 1 | 2.36 | CNTF up vs PBS |
| Dusp3 | NM_028207 | dual specificity phosphatase 3 (vaccinia virus phosphatase VH1-related) | 2.36 | CNTF up vs PBS |
| Il10ra | NM_008348 | interleukin 10 receptor, alpha | 2.36 | CNTF up vs PBS |
| Cd300lb | NM_199221 | CD300 antigen like family member B | 2.35 | CNTF up vs PBS |
| Galns | NM_016722 | galactosamine (N-acetyl)-6-sulfate sulfatase | 2.35 | CNTF up vs PBS |
| H2-D1 | NM_010380 | histocompatibility 2, D region locus 1 | 2.35 | CNTF up vs PBS |
| Cdt1 | NM_026014 | chromatin licensing and DNA replication factor 1 | 2.34 | CNTF up vs PBS |
| C1s /// LOC100044326 | NM_001097617 /// NM_144938 /// XR_030514 | complement component 1, s subcomponent /// similar to Complement component 1, s | 2.34 | CNTF up vs PBS |
| Fermt3 | NM_153795 | fermitin family homolog 3 (Drosophila) | 2.34 | CNTF up vs PBS |
| Adcy7 | NM_001037723 /// NM_001037724 /// NM_001109756 /// NM_007406 | adenylate cyclase 7 | 2.34 | CNTF up vs PBS |
| Isg20 | NM_001113527 /// NM_020583 | interferon-stimulated protein | 2.33 | CNTF up vs PBS |
| Trim25 | NM_009546 | tripartite motif-containing 25 | 2.33 | CNTF up vs PBS |
| Ticam1 | NM_174989 | toll-like receptor adaptor molecule 1 | 2.33 | CNTF up vs PBS |
| Il18rap | NM_010553 | interleukin 18 receptor accessory protein | 2.33 | CNTF up vs PBS |
| Treml2 | NM_001033405 | triggering receptor expressed on myeloid cells-like 2 | 2.32 | CNTF up vs PBS |
| Amz1 | NM_173405 | archaelysin family metallopeptidase 1 | 2.32 | CNTF up vs PBS |
| Tnfsf13b | NM_033622 | tumor necrosis factor (ligand) superfamily, member 13b | 2.32 | CNTF up vs PBS |
| Agtrap | NM_009642 | angiotensin II, type I receptor-associated protein | 2.32 | CNTF up vs PBS |
| Ppp1r3b | NM_177741 | protein phosphatase 1, regulatory (inhibitor) subunit 3B | 2.31 | CNTF up vs PBS |
| Epb4.1l4b | NM_019427 /// XM_001476161 /// XM_001476174 /// XM_980440 | erythrocyte protein band 4.1-like 4b | 2.31 | CNTF up vs PBS |
| Adcy7 | NM_001037723 /// NM_001037724 /// NM_001109756 /// NM_007406 | adenylate cyclase 7 | 2.31 | CNTF up vs PBS |
| Ddx60 | NM_001081215 | DEAD (Asp-Glu-Ala-Asp) box polypeptide 60 | 2.31 | CNTF up vs PBS |
| Ube2d3 | NM_025356 | ubiquitin-conjugating enzyme E2D 3 (UBC4/5 homolog, yeast) | 2.31 | CNTF up vs PBS |
| Ccna2 | NM_009828 | cyclin A2 | 2.30 | CNTF up vs PBS |
| Myo1e | NM_181072 | myosin IE | 2.30 | CNTF up vs PBS |
| Tst | NM_009437 | thiosulfate sulfurtransferase, mitochondrial | 2.30 | CNTF up vs PBS |
| Ddx60 | NM_001081215 | DEAD (Asp-Glu-Ala-Asp) box polypeptide 60 | 2.29 | CNTF up vs PBS |
| Junb | NM_008416 | Jun-B oncogene | 2.29 | CNTF up vs PBS |
| Lpar6 | NM_175116 | lysophosphatidic acid receptor 6 | 2.29 | CNTF up vs PBS |
| Clic4 | NM_013885 | chloride intracellular channel 4 (mitochondrial) | 2.29 | CNTF up vs PBS |
| Grn | NM_008175 | granulin | 2.29 | CNTF up vs PBS |
| Igfbp6 | NM_008344 | insulin-like growth factor binding protein 6 | 2.28 | CNTF up vs PBS |
| Tcirg1 | NM_001136091 /// NM_016921 | T-cell, immune regulator 1, ATPase, H+ transporting, lysosomal V0 protein A3 | 2.28 | CNTF up vs PBS |
| Intu | NM_175515 | inturned planar cell polarity effector homolog (Drosophila) | 2.28 | CNTF up vs PBS |
| Abca1 | NM_013454 | ATP-binding cassette, sub-family A (ABC1), member 1 | 2.28 | CNTF up vs PBS |
| Trim36 | NM_178872 | tripartite motif-containing 36 | 2.28 | CNTF up vs PBS |
| E2f3 | NM_010093 | E2F transcription factor 3 | 2.27 | CNTF up vs PBS |
| Stk17b | NM_133810 | serine/threonine kinase 17b (apoptosis-inducing) | 2.27 | CNTF up vs PBS |
| Entpd1 | NM_009848 | ectonucleoside triphosphate diphosphohydrolase 1 | 2.27 | CNTF up vs PBS |
| Mcm5 | NM_008566 | minichromosome maintenance deficient 5, cell division cycle 46 (S. cerevisiae) | 2.26 | CNTF up vs PBS |
| Man2b1 | NM_010764 | mannosidase 2, alpha B1 | 2.26 | CNTF up vs PBS |
| Plaur | NM_011113 | plasminogen activator, urokinase receptor | 2.26 | CNTF up vs PBS |
| Cdh13 | NM_019707 | cadherin 13 | 2.26 | CNTF up vs PBS |
| Pdcd6ip | NM_011052 | programmed cell death 6 interacting protein | 2.26 | CNTF up vs PBS |
| 4930413G21Rik | XM_001478471 /// XM_001479210 | RIKEN cDNA 4930413G21 gene | 2.26 | CNTF up vs PBS |
| Mcl1 | NM_008562 | myeloid cell leukemia sequence 1 | 2.26 | CNTF up vs PBS |
| Stat3 | NM_011486 /// NM_213659 /// NM_213660 | signal transducer and activator of transcription 3 | 2.25 | CNTF up vs PBS |
| Ube2l6 | NM_019949 | ubiquitin-conjugating enzyme E2L 6 | 2.25 | CNTF up vs PBS |
| Dlgap5 | NM_001145949 /// NM_144553 | discs, large (Drosophila) homolog-associated protein 5 | 2.25 | CNTF up vs PBS |
| Chek1 | NM_007691 | checkpoint kinase 1 homolog (S. pombe) | 2.25 | CNTF up vs PBS |
| Ckap2l | NM_181589 | cytoskeleton associated protein 2-like | 2.25 | CNTF up vs PBS |
| Ccdc93 | NM_001025156 /// NM_027567 /// NM_029955 | coiled-coil domain containing 93 | 2.25 | CNTF up vs PBS |
| C1qb | NM_009777 | complement component 1, q subcomponent, beta polypeptide | 2.25 | CNTF up vs PBS |
| Itprip | NM_001001738 | inositol 1,4,5-triphosphate receptor interacting protein | 2.25 | CNTF up vs PBS |
| Prkcd | NM_011103 | protein kinase C, delta | 2.25 | CNTF up vs PBS |
| Ly96 | NM_001159711 /// NM_016923 | lymphocyte antigen 96 | 2.25 | CNTF up vs PBS |
| Syngr1 | NM_009303 /// NM_207708 | synaptogyrin 1 | 2.24 | CNTF up vs PBS |
| Lmna | NM_001002011 /// NM_001111102 /// NM_019390 | lamin A | 2.24 | CNTF up vs PBS |
| Gpr137b /// Gpr137b-ps /// LOC100044979 | NM_031999 /// NR_003568 /// XM_001473441 /// XR_001658 | G protein-coupled receptor 137B /// G protein-coupled receptor 137B, pseudogene | 2.24 | CNTF up vs PBS |
| Slamf7 | NM_144539 | SLAM family member 7 | 2.23 | CNTF up vs PBS |
| Timp2 | NM_011594 | tissue inhibitor of metalloproteinase 2 | 2.23 | CNTF up vs PBS |
| Rtp4 | NM_023386 | receptor transporter protein 4 | 2.23 | CNTF up vs PBS |
| Bag3 | NM_013863 | BCL2-associated athanogene 3 | 2.23 | CNTF up vs PBS |
| Fam26e | NM_178908 | family with sequence similarity 26, member E | 2.23 | CNTF up vs PBS |
| Suclg2 | NM_011507 | succinate-Coenzyme A ligase, GDP-forming, beta subunit | 2.22 | CNTF up vs PBS |
| Batf | NM_016767 | basic leucine zipper transcription factor, ATF-like | 2.22 | CNTF up vs PBS |
| Cdkn1a | NM_001111099 /// NM_007669 | cyclin-dependent kinase inhibitor 1A (P21) | 2.22 | CNTF up vs PBS |
| Dclk1 | NM_001111051 /// NM_001111052 /// NM_001111053 /// NM_019978 | doublecortin-like kinase 1 | 2.22 | CNTF up vs PBS |
| Tspan14 | NM_145928 | tetraspanin 14 | 2.21 | CNTF up vs PBS |
| Tifab | NM_145976 | TRAF-interacting protein with forkhead-associated domain, family member B | 2.21 | CNTF up vs PBS |
| Il17ra | NM_008359 | interleukin 17 receptor A | 2.20 | CNTF up vs PBS |
| Rap1b | NM_024457 | RAS related protein 1b | 2.20 | CNTF up vs PBS |
| Adamts1 | NM_009621 | a disintegrin-like and metallopeptidase (reprolysin type) with thrombospondin ty | 2.20 | CNTF up vs PBS |
| Erap1 | NM_030711 | endoplasmic reticulum aminopeptidase 1 | 2.20 | CNTF up vs PBS |
| C1qb | NM_009777 | complement component 1, q subcomponent, beta polypeptide | 2.20 | CNTF up vs PBS |
| Lamp2 | NM_001017959 /// NM_010685 | lysosomal-associated membrane protein 2 | 2.20 | CNTF up vs PBS |
| Lsm12 | NM_172947 | LSM12 homolog (S. cerevisiae) | 2.19 | CNTF up vs PBS |
| Epsti1 | NM_029495 /// NM_178825 | epithelial stromal interaction 1 (breast) | 2.19 | CNTF up vs PBS |
| Ang | NM_001161731 /// NM_007447 | angiogenin, ribonuclease, RNase A family, 5 | 2.19 | CNTF up vs PBS |
| Fignl1 | NM_001163359 /// NM_001163360 /// NM_021891 | fidgetin-like 1 | 2.19 | CNTF up vs PBS |
| Grn | NM_008175 | granulin | 2.19 | CNTF up vs PBS |
| Cdh13 | NM_019707 | cadherin 13 | 2.19 | CNTF up vs PBS |
| Dbf4 | NM_013726 | DBF4 homolog (S. cerevisiae) | 2.18 | CNTF up vs PBS |
| 4930534B04Rik | NM_181815 | RIKEN cDNA 4930534B04 gene | 2.18 | CNTF up vs PBS |
| Plat | NM_008872 | plasminogen activator, tissue | 2.18 | CNTF up vs PBS |
| Dck | NM_007832 | deoxycytidine kinase | 2.18 | CNTF up vs PBS |
| Myh9 | NM_022410 | myosin, heavy polypeptide 9, non-muscle | 2.18 | CNTF up vs PBS |
| Fam176b | NM_172145 | family with sequence similarity 176, member B | 2.17 | CNTF up vs PBS |
| Gm5188 | XM_001477883 /// XM_356600 /// XM_914337 | predicted gene 5188 | 2.17 | CNTF up vs PBS |
| Abcg3 | NM_030239 | ATP-binding cassette, sub-family G (WHITE), member 3 | 2.17 | CNTF up vs PBS |
| Cds2 | NM_138651 | CDP-diacylglycerol synthase (phosphatidate cytidylyltransferase) 2 | 2.17 | CNTF up vs PBS |
| D7Bwg0826e | --- | DNA segment, Chr 7, Brigham & Women's Genetics 0826 expressed | 2.17 | CNTF up vs PBS |
| Slc10a3 | NM_145406 | solute carrier family 10 (sodium/bile acid cotransporter family), member 3 | 2.17 | CNTF up vs PBS |
| Ninl | NM_207204 | ninein-like | 2.17 | CNTF up vs PBS |
| Elovl1 | NM_001039175 /// NM_001039176 /// NM_019422 | elongation of very long chain fatty acids (FEN1/Elo2, SUR4/Elo3, yeast)-like 1 | 2.17 | CNTF up vs PBS |
| Gm6742 /// Gm7993 /// Gm9115 /// LOC677259 /// Odc1 | NM_013614 /// XR_001849 /// XR_002167 /// XR_030600 /// XR_031424 /// XR_031670 | predicted gene 6742 /// predicted gene 7993 /// predicted gene 9115 /// similar | 2.17 | CNTF up vs PBS |
| Lgmn | NM_011175 | legumain | 2.16 | CNTF up vs PBS |
| Mafb | NM_010658 | v-maf musculoaponeurotic fibrosarcoma oncogene family, protein B (avian) | 2.16 | CNTF up vs PBS |
| Nfkbia | NM_010907 | Nuclear factor of kappa light polypeptide gene enhancer in B-cells inhibitor, al | 2.16 | CNTF up vs PBS |
| Tgm2 | NM_009373 | transglutaminase 2, C polypeptide | 2.16 | CNTF up vs PBS |
| Arid5a | NM_145996 | AT rich interactive domain 5A (MRF1-like) | 2.16 | CNTF up vs PBS |
| 2210406H18Rik | --- | RIKEN cDNA 2210406H18 gene | 2.16 | CNTF up vs PBS |
| 1810019J16Rik | NM_001083916 /// NM_133707 | RIKEN cDNA 1810019J16 gene | 2.15 | CNTF up vs PBS |
| Irx3 | NM_008393 | Iroquois related homeobox 3 (Drosophila) | 2.15 | CNTF up vs PBS |
| Sdf2l1 | NM_022324 | stromal cell-derived factor 2-like 1 | 2.15 | CNTF up vs PBS |
| Rassf3 | NM_138956 | Ras association (RalGDS/AF-6) domain family member 3 | 2.15 | CNTF up vs PBS |
| Ddc | NM_016672 | dopa decarboxylase | 2.15 | CNTF up vs PBS |
| Ptpn1 | NM_011201 | protein tyrosine phosphatase, non-receptor type 1 | 2.15 | CNTF up vs PBS |
| Zfand3 | NM_148926 | zinc finger, AN1-type domain 3 | 2.15 | CNTF up vs PBS |
| Ucp2 | NM_011671 | uncoupling protein 2 (mitochondrial, proton carrier) | 2.15 | CNTF up vs PBS |
| Gm7325 | XM_001000918 | predicted gene 7325 | 2.14 | CNTF up vs PBS |
| Ifi35 | NM_027320 | interferon-induced protein 35 | 2.14 | CNTF up vs PBS |
| 2700007P21Rik | NM_001025102 /// NM_173750 | RIKEN cDNA 2700007P21 gene | 2.14 | CNTF up vs PBS |
| Clec2d | NM_053109 | C-type lectin domain family 2, member d | 2.14 | CNTF up vs PBS |
| Fermt3 | NM_153795 | fermitin family homolog 3 (Drosophila) | 2.14 | CNTF up vs PBS |
| Siah2 | NM_009174 | seven in absentia 2 | 2.14 | CNTF up vs PBS |
| Pdcd6ip | NM_011052 | Programmed cell death 6 interacting protein | 2.14 | CNTF up vs PBS |
| Trim21 | NM_001082552 /// NM_009277 | tripartite motif-containing 21 | 2.14 | CNTF up vs PBS |
| Snx6 | NM_026998 | sorting nexin 6 | 2.14 | CNTF up vs PBS |
| Shb | NM_001033306 /// XM_001474892 | src homology 2 domain-containing transforming protein B | 2.13 | CNTF up vs PBS |
| Aoah | NM_012054 | acyloxyacyl hydrolase | 2.13 | CNTF up vs PBS |
| Ctdsp2 /// Gm9770 | NM_001113470 /// NM_146012 /// XR_032943 /// XR_034953 | CTD (carboxy-terminal domain, RNA polymerase II, polypeptide A) small phosphatas | 2.13 | CNTF up vs PBS |
| Trim25 | NM_009546 | tripartite motif-containing 25 | 2.13 | CNTF up vs PBS |
| Clec14a | NM_025809 | C-type lectin domain family 14, member a | 2.13 | CNTF up vs PBS |
| Ptpn1 | NM_011201 | protein tyrosine phosphatase, non-receptor type 1 | 2.13 | CNTF up vs PBS |
| Nupr1 | NM_019738 | nuclear protein 1 | 2.13 | CNTF up vs PBS |
| Nfam1 | NM_028728 | Nfat activating molecule with ITAM motif 1 | 2.13 | CNTF up vs PBS |
| Grn | NM_008175 | granulin | 2.13 | CNTF up vs PBS |
| Trub2 | NM_145520 /// NR_027344 | TruB pseudouridine (psi) synthase homolog 2 (E. coli) | 2.12 | CNTF up vs PBS |
| Slc1a5 | NM_009201 | solute carrier family 1 (neutral amino acid transporter), member 5 | 2.12 | CNTF up vs PBS |
| Mtmr11 | NM_181409 | myotubularin related protein 11 | 2.12 | CNTF up vs PBS |
| St8sia4 | NM_001159745 /// NM_009183 | ST8 alpha-N-acetyl-neuraminide alpha-2,8-sialyltransferase 4 | 2.12 | CNTF up vs PBS |
| Entpd1 | NM_009848 | ectonucleoside triphosphate diphosphohydrolase 1 | 2.12 | CNTF up vs PBS |
| Kif18a | NM_139303 | kinesin family member 18A | 2.12 | CNTF up vs PBS |
| Ptprc | NM_001111316 /// NM_011210 | Protein tyrosine phosphatase, receptor type, C | 2.12 | CNTF up vs PBS |
| Dtl | NM_029766 | denticleless homolog (Drosophila) | 2.12 | CNTF up vs PBS |
| Ucp2 | NM_011671 | uncoupling protein 2 (mitochondrial, proton carrier) | 2.12 | CNTF up vs PBS |
| Pkp2 | NM_026163 | Plakophilin 2 | 2.12 | CNTF up vs PBS |
| Cdt1 | NM_026014 | chromatin licensing and DNA replication factor 1 | 2.11 | CNTF up vs PBS |
| Pycard | NM_023258 | PYD and CARD domain containing | 2.11 | CNTF up vs PBS |
| Pgpep1 | NM_023217 | pyroglutamyl-peptidase I | 2.11 | CNTF up vs PBS |
| Rgs19 | NM_026446 | regulator of G-protein signaling 19 | 2.11 | CNTF up vs PBS |
| Itgal | NM_008400 | integrin alpha L | 2.11 | CNTF up vs PBS |
| Hoxb4 | NM_010459 | homeo box B4 | 2.11 | CNTF up vs PBS |
| Rph3al | NM_029548 | rabphilin 3A-like (without C2 domains) | 2.11 | CNTF up vs PBS |
| Creg1 | NM_011804 | cellular repressor of E1A-stimulated genes 1 | 2.11 | CNTF up vs PBS |
| Traf1 | NM_009421 | TNF receptor-associated factor 1 | 2.11 | CNTF up vs PBS |
| Kpna1 | NM_008465 | karyopherin (importin) alpha 1 | 2.11 | CNTF up vs PBS |
| Rbl1 | NM_001139516 /// NM_011249 | retinoblastoma-like 1 (p107) | 2.11 | CNTF up vs PBS |
| Hlx | NM_008250 | H2.0-like homeobox | 2.11 | CNTF up vs PBS |
| Eya3 | NM_010166 /// NM_210071 /// NM_211357 | eyes absent 3 homolog (Drosophila) | 2.10 | CNTF up vs PBS |
| Foxj2 | NM_021899 | forkhead box J2 | 2.10 | CNTF up vs PBS |
| 4930547N16Rik | NM_029249 | RIKEN cDNA 4930547N16 gene | 2.10 | CNTF up vs PBS |
| Clec4a2 | NM_011999 | C-type lectin domain family 4, member a2 | 2.10 | CNTF up vs PBS |
| Cd97 | NM_001163029 /// NM_001163030 /// NM_001163031 /// NM_011925 | CD97 antigen | 2.10 | CNTF up vs PBS |
| Cklf | NM_001037841 /// NM_029295 | chemokine-like factor | 2.10 | CNTF up vs PBS |
| Anxa4 | NM_013471 | annexin A4 | 2.10 | CNTF up vs PBS |
| 9530048O09Rik | NR_024078 /// XM_001477060 /// XR_035186 /// XR_035205 /// XR_035280 | RIKEN cDNA 9530048O09 gene | 2.09 | CNTF up vs PBS |
| Rgs19 | NM_026446 | regulator of G-protein signaling 19 | 2.09 | CNTF up vs PBS |
| Snap23 | NM_009222 | synaptosomal-associated protein 23 | 2.09 | CNTF up vs PBS |
| Exoc6 | NM_175353 | exocyst complex component 6 | 2.09 | CNTF up vs PBS |
| Zfand5 | NM_009551 | zinc finger, AN1-type domain 5 | 2.09 | CNTF up vs PBS |
| Trub2 | NM_145520 /// NR_027344 | TruB pseudouridine (psi) synthase homolog 2 (E. coli) | 2.09 | CNTF up vs PBS |
| 4632434I11Rik | NM_001080995 | RIKEN cDNA 4632434I11 gene | 2.09 | CNTF up vs PBS |
| Elovl1 | NM_001039175 /// NM_001039176 /// NM_019422 | elongation of very long chain fatty acids (FEN1/Elo2, SUR4/Elo3, yeast)-like 1 | 2.09 | CNTF up vs PBS |
| Cp | NM_001042611 /// NM_007752 | ceruloplasmin | 2.08 | CNTF up vs PBS |
| Oas2 | NM_145227 | 2'-5' oligoadenylate synthetase 2 | 2.08 | CNTF up vs PBS |
| Gas2l3 | NM_001033331 /// NM_001079876 | growth arrest-specific 2 like 3 | 2.08 | CNTF up vs PBS |
| Tlr6 | NM_011604 | toll-like receptor 6 | 2.08 | CNTF up vs PBS |
| Asxl2 | NM_172421 | additional sex combs like 2 (Drosophila) | 2.08 | CNTF up vs PBS |
| Cd33 | NM_001111058 /// NM_021293 | CD33 antigen | 2.07 | CNTF up vs PBS |
| Seh1l | NM_001039088 /// NM_028112 | SEH1-like (S. cerevisiae | 2.07 | CNTF up vs PBS |
| Fhad1 | NM_028429 /// NM_177868 | forkhead-associated (FHA) phosphopeptide binding domain 1 | 2.07 | CNTF up vs PBS |
| Exoc6 | NM_175353 | exocyst complex component 6 | 2.07 | CNTF up vs PBS |
| Scarb2 | NM_007644 | scavenger receptor class B, member 2 | 2.07 | CNTF up vs PBS |
| Cotl1 | NM_028071 | coactosin-like 1 (Dictyostelium) | 2.07 | CNTF up vs PBS |
| Cpxm2 | NM_018867 | carboxypeptidase X 2 (M14 family) | 2.07 | CNTF up vs PBS |
| Ece2 | NM_025462 /// NM_139293 /// NM_177940 /// NM_177941 /// NM_177942 | endothelin converting enzyme 2 | 2.06 | CNTF up vs PBS |
| Nupr1 | NM_019738 | nuclear protein 1 | 2.06 | CNTF up vs PBS |
| Acsl5 | NM_027976 | acyl-CoA synthetase long-chain family member 5 | 2.06 | CNTF up vs PBS |
| Mdm4 | NM_008575 | transformed mouse 3T3 cell double minute 4 | 2.06 | CNTF up vs PBS |
| Cd200r1 | NM_021325 | CD200 receptor 1 | 2.06 | CNTF up vs PBS |
| Creb1 | NM_001037726 /// NM_009952 /// NM_133828 | cAMP responsive element binding protein 1 | 2.06 | CNTF up vs PBS |
| Gba | NM_001077411 /// NM_008094 | glucosidase, beta, acid | 2.05 | CNTF up vs PBS |
| Col4a1 | NM_009931 | collagen, type IV, alpha 1 | 2.05 | CNTF up vs PBS |
| Slc25a45 | NM_134154 | solute carrier family 25, member 45 | 2.05 | CNTF up vs PBS |
| LOC100047179 /// Tgm4 | NM_177911 /// XM_001477587 | similar to experimental autoimmune prostatitis antigen 1 /// transglutaminase 4 | 2.05 | CNTF up vs PBS |
| Tagln2 | NM_178598 | transgelin 2 | 2.04 | CNTF up vs PBS |
| Ttf2 | NM_001013026 | transcription termination factor, RNA polymerase II | 2.04 | CNTF up vs PBS |
| Me2 | NM_145494 | malic enzyme 2, NAD(+)-dependent, mitochondrial | 2.04 | CNTF up vs PBS |
| Sft2d2 | NM_145512 | SFT2 domain containing 2 | 2.04 | CNTF up vs PBS |
| 3110003A17Rik | NM_028440 /// XM_001473245 /// XM_912949 | RIKEN cDNA 3110003A17 gene | 2.04 | CNTF up vs PBS |
| Eri1 | NM_026067 | exoribonuclease 1 | 2.04 | CNTF up vs PBS |
| Atad2 | NM_027435 | ATPase family, AAA domain containing 2 | 2.04 | CNTF up vs PBS |
| Trim14 | NM_029077 | tripartite motif-containing 14 | 2.04 | CNTF up vs PBS |
| Fgfr1op2 | NM_026218 | FGFR1 oncogene partner 2 | 2.04 | CNTF up vs PBS |
| Rnf19a | NM_013923 | ring finger protein 19A | 2.04 | CNTF up vs PBS |
| Chl1 | NM_007697 | cell adhesion molecule with homology to L1CAM | 2.03 | CNTF up vs PBS |
| Dclk1 | NM_001111051 /// NM_001111052 /// NM_001111053 /// NM_019978 | doublecortin-like kinase 1 | 2.03 | CNTF up vs PBS |
| AI451617 /// Trim30 | NM_009099 /// NM_199146 | expressed sequence AI451617 /// tripartite motif-containing 30 | 2.03 | CNTF up vs PBS |
| Cbl | NM_007619 | Casitas B-lineage lymphoma | 2.03 | CNTF up vs PBS |
| Fancd2 | NM_001033244 | Fanconi anemia, complementation group D2 | 2.03 | CNTF up vs PBS |
| Ppp1r3e | XM_909597 /// XM_993154 | protein phosphatase 1, regulatory (inhibitor) subunit 3E | 2.03 | CNTF up vs PBS |
| Rgs4 | NM_009062 | regulator of G-protein signaling 4 | 2.03 | CNTF up vs PBS |
| AU020177 | --- | expressed sequence AU020177 | 2.03 | CNTF up vs PBS |
| D19Ertd386e | NM_177464 | DNA segment, Chr 19, ERATO Doi 386, expressed | 2.03 | CNTF up vs PBS |
| Sla | NM_001029841 /// NM_009192 | src-like adaptor | 2.03 | CNTF up vs PBS |
| Csf1 | NM_001113529 /// NM_001113530 /// NM_007778 | colony stimulating factor 1 (macrophage) | 2.03 | CNTF up vs PBS |
| Pde2a | NM_001008548 /// NM_001143848 /// NM_001143849 /// NR_026574 | phosphodiesterase 2A, cGMP-stimulated | 2.03 | CNTF up vs PBS |
| Eif4ebp1 | NM_007918 | eukaryotic translation initiation factor 4E binding protein 1 | 2.03 | CNTF up vs PBS |
| Sar1a | NM_009120 | SAR1 gene homolog A (S. cerevisiae) | 2.02 | CNTF up vs PBS |
| Hdlbp | NM_133808 | high density lipoprotein (HDL) binding protein | 2.02 | CNTF up vs PBS |
| Rhoh | NM_001081105 | ras homolog gene family, member H | 2.02 | CNTF up vs PBS |
| Cttnbp2nl | NM_001163332 /// NM_001163333 /// NM_030249 | CTTNBP2 N-terminal like | 2.02 | CNTF up vs PBS |
| Helb | NM_080446 | helicase (DNA) B | 2.02 | CNTF up vs PBS |
| Gm3756 /// Gm5620 /// Gm7172 /// LOC100044416 /// LOC100045728 /// Tuba1a /// Tuba1b /// Tuba1c | NM_009448 /// NM_011653 /// NM_011654 /// XM_486246 /// XM_896498 /// XM_904657 | predicted gene 3756 /// predicted gene 5620 /// predicted gene 7172 /// similar | 2.01 | CNTF up vs PBS |
| Nme2 | NM_001077529 /// NM_008705 | non-metastatic cells 2, protein (NM23B) expressed in | 2.01 | CNTF up vs PBS |
| LOC100045163 /// Plbd1 | NM_025806 /// XM_001473518 | similar to RIKEN cDNA 1100001H23 gene /// phospholipase B domain containing 1 | 2.01 | CNTF up vs PBS |
| Ero1l | NM_015774 | ERO1-like (S. cerevisiae) | 2.01 | CNTF up vs PBS |
| Ppp1r14b | NM_008889 | protein phosphatase 1, regulatory (inhibitor) subunit 14B | 2.01 | CNTF up vs PBS |
| 1200009F10Rik | NM_026166 /// NM_027078 | RIKEN cDNA 1200009F10 gene | 2.01 | CNTF up vs PBS |
| Lcp2 | NM_010696 | lymphocyte cytosolic protein 2 | 2.00 | CNTF up vs PBS |
| Plekha2 | NM_031257 | pleckstrin homology domain-containing, family A (phosphoinositide binding specif | 2.00 | CNTF up vs PBS |
| Ppic | NM_008908 | peptidylprolyl isomerase C | 2.00 | CNTF up vs PBS |
| Fgf9 | NM_013518 | fibroblast growth factor 9 | -2.00 | CNTF down vs PBS |
| Larp4 | NM_001024526 /// NM_001080948 | La ribonucleoprotein domain family, member 4 | -2.00 | CNTF down vs PBS |
| Ss18 | NM_001161369 /// NM_001161370 /// NM_001161371 /// NM_009280 | synovial sarcoma translocation, Chromosome 18 | -2.00 | CNTF down vs PBS |
| Dach2 | NM_001142570 /// NM_033605 | dachshund 2 (Drosophila) | -2.00 | CNTF down vs PBS |
| Ppp3cb | NM_008914 | protein phosphatase 3, catalytic subunit, beta isoform | -2.00 | CNTF down vs PBS |
| A730035I17Rik | --- | RIKEN cDNA A730035I17 gene | -2.01 | CNTF down vs PBS |
| Bat2d | NM_001081290 | BAT2 domain containing 1 | -2.01 | CNTF down vs PBS |
| Hpca | NM_001130419 /// NM_010471 | hippocalcin | -2.01 | CNTF down vs PBS |
| A430105D02Rik | --- | RIKEN cDNA A430105D02 gene | -2.01 | CNTF down vs PBS |
| Eif2c3 | NM_153402 | eukaryotic translation initiation factor 2C, 3 | -2.01 | CNTF down vs PBS |
| Gm15506 | XM_001472950 /// XM_001474991 | Predicted gene 15506 | -2.01 | CNTF down vs PBS |
| 2210416J07Rik | --- | RIKEN cDNA 2210416J07 gene | -2.01 | CNTF down vs PBS |
| Ubtf | NM_001044383 /// NM_011551 | upstream binding transcription factor, RNA polymerase I | -2.01 | CNTF down vs PBS |
| Baz1b | NM_011714 | Bromodomain adjacent to zinc finger domain, 1B | -2.02 | CNTF down vs PBS |
| Gas1 | NM_008086 | Growth arrest specific 1 | -2.02 | CNTF down vs PBS |
| Pebp4 | NM_028526 /// NM_028560 | phosphatidylethanolamine binding protein 4 | -2.02 | CNTF down vs PBS |
| 1110028C15Rik | NM_001122738 /// NM_177645 | RIKEN cDNA 1110028C15 gene | -2.02 | CNTF down vs PBS |
| Nfix | NM_001081981 /// NM_001081982 /// NM_010906 | nuclear factor I/X | -2.02 | CNTF down vs PBS |
| Gm4455 /// Zfp422-rs1 | NM_001142957 /// NM_029952 /// XM_001472768 /// XM_001480577 /// XM_001480580 | predicted gene 4455 /// zinc finger protein 422, related sequence 1 | -2.02 | CNTF down vs PBS |
| Atxn7 | NM_139227 | ataxin 7 | -2.02 | CNTF down vs PBS |
| LOC100045442 /// Stag1 | NM_009282 /// XM_001474252 | similar to Stromal antigen 1 /// stromal antigen 1 | -2.02 | CNTF down vs PBS |
| 8030425K09Rik | --- | RIKEN cDNA 8030425K09 gene | -2.03 | CNTF down vs PBS |
| Epha7 | NM_001122889 /// NM_010141 | Eph receptor A7 | -2.03 | CNTF down vs PBS |
| Apc | NM_007462 | adenomatosis polyposis coli | -2.03 | CNTF down vs PBS |
| Igfbpl1 | NM_018741 | insulin-like growth factor binding protein-like 1 | -2.03 | CNTF down vs PBS |
| Mtf2 | NM_013827 | Metal response element binding transcription factor 2 | -2.03 | CNTF down vs PBS |
| 2900075N08Rik /// Nlgn3 | NM_172932 /// XM_001478124 | RIKEN cDNA 2900075N08 gene /// neuroligin 3 | -2.03 | CNTF down vs PBS |
| Myt1l | NM_001093775 /// NM_001093776 /// NM_001093778 /// NM_008666 /// XM_001480703 | myelin transcription factor 1-like | -2.03 | CNTF down vs PBS |
| LOC100044751 /// Zfp207 | NM_001130169 /// NM_001130170 /// NM_001130171 /// NM_011751 /// XM_001473252 // | hypothetical protein LOC100044751 /// zinc finger protein 207 | -2.03 | CNTF down vs PBS |
| Dlgap1 | NM_001128180 /// NM_001128181 /// NM_027712 /// NM_177639 | discs, large (Drosophila) homolog-associated protein 1 | -2.03 | CNTF down vs PBS |
| Ttc3 | NM_009441 | tetratricopeptide repeat domain 3 | -2.03 | CNTF down vs PBS |
| Btbd7 | NM_172806 | BTB (POZ) domain containing 7 | -2.03 | CNTF down vs PBS |
| Pppde1 | NM_024282 | PPPDE peptidase domain containing 1 | -2.04 | CNTF down vs PBS |
| 6720427H10Rik | --- | RIKEN cDNA 6720427H10 gene | -2.04 | CNTF down vs PBS |
| BC002230 | NM_183155 /// XM_484171 /// XM_912077 | cDNA sequence BC002230 | -2.04 | CNTF down vs PBS |
| Map3k10 | NM_001081292 | Mitogen-activated protein kinase kinase kinase 10 | -2.04 | CNTF down vs PBS |
| Map4k3 | NM_001081357 | Mitogen-activated protein kinase kinase kinase kinase 3 | -2.04 | CNTF down vs PBS |
| Ddx6 | NM_001110826 /// NM_007841 /// NM_181324 | DEAD (Asp-Glu-Ala-Asp) box polypeptide 6 | -2.05 | CNTF down vs PBS |
| Bche | NM_009738 | butyrylcholinesterase | -2.05 | CNTF down vs PBS |
| 5830490A04Rik | --- | RIKEN cDNA 5830490A04 gene | -2.05 | CNTF down vs PBS |
| 2700049A03Rik | NM_001163378 /// NM_029818 /// XM_126944 /// XM_916714 | RIKEN cDNA 2700049A03 gene | -2.05 | CNTF down vs PBS |
| Kcnq1ot1 | NR_001461 | KCNQ1 overlapping transcript 1 | -2.05 | CNTF down vs PBS |
| Fam48a | NM_019995 | family with sequence similarity 48, member A | -2.05 | CNTF down vs PBS |
| Pafah1b1 | NM_013625 /// XM_001476465 | platelet-activating factor acetylhydrolase, isoform 1b, subunit 1 | -2.05 | CNTF down vs PBS |
| 4930583H14Rik | NM_026358 /// NR_028121 | RIKEN cDNA 4930583H14 gene | -2.05 | CNTF down vs PBS |
| Hira | NM_010435 | Histone cell cycle regulation defective homolog A (S. cerevisiae) | -2.05 | CNTF down vs PBS |
| Dhrs7 | NM_025522 | dehydrogenase/reductase (SDR family) member 7 | -2.05 | CNTF down vs PBS |
| Ebpl | NM_026598 | emopamil binding protein-like | -2.05 | CNTF down vs PBS |
| Srpk2 | NM_009274 | serine/arginine-rich protein specific kinase 2 | -2.05 | CNTF down vs PBS |
| Hmgcs1 | NM_145942 | 3-hydroxy-3-methylglutaryl-Coenzyme A synthase 1 | -2.05 | CNTF down vs PBS |
| Kcnj3 | NM_008426 | potassium inwardly-rectifying channel, subfamily J, member 3 | -2.05 | CNTF down vs PBS |
| Nfix | NM_001081981 /// NM_001081982 /// NM_010906 | nuclear factor I/X | -2.06 | CNTF down vs PBS |
| Apcdd1 | NM_133237 | adenomatosis polyposis coli down-regulated 1 | -2.06 | CNTF down vs PBS |
| 6330419E04Rik | --- | RIKEN cDNA 6330419E04 gene | -2.06 | CNTF down vs PBS |
| Plekha1 | NM_133942 | pleckstrin homology domain containing, family A (phosphoinositide binding specif | -2.06 | CNTF down vs PBS |
| Zbtb8b | NM_153541 | zinc finger and BTB domain containing 8b | -2.06 | CNTF down vs PBS |
| Rims1 | NM_001012623 /// NM_001012624 /// NM_001012625 /// NM_053270 /// NM_183018 | regulating synaptic membrane exocytosis 1 | -2.06 | CNTF down vs PBS |
| Senp7 | NM_001003971 /// NM_001003972 /// NM_001003973 /// NM_025483 | SUMO1/sentrin specific peptidase 7 | -2.06 | CNTF down vs PBS |
| E530001K10Rik | XM_001478872 /// XM_001479701 | RIKEN cDNA E530001K10 gene | -2.07 | CNTF down vs PBS |
| Dnm3 | NM_001038619 /// NM_172646 | dynamin 3 | -2.07 | CNTF down vs PBS |
| Gria2 | NM_001039195 /// NM_001083806 /// NM_013540 | glutamate receptor, ionotropic, AMPA2 (alpha 2) | -2.07 | CNTF down vs PBS |
| Lnpep | NM_172827 | leucyl/cystinyl aminopeptidase | -2.07 | CNTF down vs PBS |
| 2900092N22Rik | --- | RIKEN cDNA 2900092N22 gene | -2.07 | CNTF down vs PBS |
| 5330434G04Rik | NR_015552 /// XM_001474911 /// XM_001479629 | RIKEN cDNA 5330434G04 gene | -2.07 | CNTF down vs PBS |
| Klhl22 | NM_145479 /// XM_001475578 /// XM_001478907 | kelch-like 22 (Drosophila) | -2.08 | CNTF down vs PBS |
| A730017C20Rik | NM_173759 | RIKEN cDNA A730017C20 gene | -2.08 | CNTF down vs PBS |
| Zfp91 | NM_053009 | zinc finger protein 91 | -2.08 | CNTF down vs PBS |
| Slc25a27 | NM_028711 | Solute carrier family 25, member 27 | -2.08 | CNTF down vs PBS |
| Srrm2 | NM_175229 | serine/arginine repetitive matrix 2 | -2.08 | CNTF down vs PBS |
| Dis3l2 | NM_153530 | DIS3 mitotic control homolog (S. cerevisiae)-like 2 | -2.08 | CNTF down vs PBS |
| Dpysl4 | NM_011993 | dihydropyrimidinase-like 4 | -2.08 | CNTF down vs PBS |
| Tm2d1 | NM_053157 | TM2 domain containing 1 | -2.08 | CNTF down vs PBS |
| 2300009A05Rik | NM_027090 /// XM_204313 /// XM_898537 /// XM_925361 /// XM_925362 | RIKEN cDNA 2300009A05 gene | -2.08 | CNTF down vs PBS |
| Efna5 | NM_010109 /// NM_207654 | ephrin A5 | -2.08 | CNTF down vs PBS |
| Msi2 | NM_054043 /// XM_001471946 | Musashi homolog 2 (Drosophila) | -2.08 | CNTF down vs PBS |
| Angpt1 | NM_009640 | angiopoietin 1 | -2.09 | CNTF down vs PBS |
| Fam126b | NM_172513 | family with sequence similarity 126, member B | -2.09 | CNTF down vs PBS |
| Pum2 | NM_001160219 /// NM_001160220 /// NM_001160221 /// NM_001160222 /// NM_030723 // | pumilio 2 (Drosophila) | -2.09 | CNTF down vs PBS |
| Sfrs8 | NM_172276 | splicing factor, arginine/serine-rich 8 | -2.09 | CNTF down vs PBS |
| Phip | NM_001081216 | Pleckstrin homology domain interacting protein | -2.10 | CNTF down vs PBS |
| Ylpm1 | NM_178363 | YLP motif containing 1 | -2.10 | CNTF down vs PBS |
| Myt1 | NM_008665 | Myelin transcription factor 1 | -2.10 | CNTF down vs PBS |
| Psmd11 | NM_178616 | proteasome (prosome, macropain) 26S subunit, non-ATPase, 11 | -2.10 | CNTF down vs PBS |
| Trhde | NM_146241 | TRH-degrading enzyme | -2.10 | CNTF down vs PBS |
| Sdc2 | NM_008304 | syndecan 2 | -2.10 | CNTF down vs PBS |
| Hspa12a | NM_175199 | heat shock protein 12A | -2.10 | CNTF down vs PBS |
| Kcna2 | NM_008417 | potassium voltage-gated channel, shaker-related subfamily, member 2 | -2.10 | CNTF down vs PBS |
| Ankrd43 | NM_183173 | ankyrin repeat domain 43 | -2.10 | CNTF down vs PBS |
| Ppm1l | NM_178726 | protein phosphatase 1 (formerly 2C)-like | -2.11 | CNTF down vs PBS |
| Zc3h11a | NM_144530 | zinc finger CCCH type containing 11A | -2.11 | CNTF down vs PBS |
| Cpeb3 | NM_198300 | cytoplasmic polyadenylation element binding protein 3 | -2.11 | CNTF down vs PBS |
| Slc25a44 | NM_001145876 /// NM_001145877 /// NM_178696 /// NR_027357 | solute carrier family 25, member 44 | -2.11 | CNTF down vs PBS |
| Letmd1 | NM_134093 | LETM1 domain containing 1 | -2.11 | CNTF down vs PBS |
| Lrig3 | NM_177152 | leucine-rich repeats and immunoglobulin-like domains 3 | -2.11 | CNTF down vs PBS |
| Mapk8ip1 | NM_011162 | mitogen-activated protein kinase 8 interacting protein 1 | -2.11 | CNTF down vs PBS |
| Rbm12 | NM_029397 /// NM_170598 | RNA binding motif protein 12 | -2.11 | CNTF down vs PBS |
| Schip1 | NM_001113419 /// NM_001113420 /// NM_001113421 /// NM_013928 | Schwannomin interacting protein 1 | -2.11 | CNTF down vs PBS |
| Tbx3 | NM_011535 /// NM_198052 /// XM_001477632 | T-box 3 | -2.12 | CNTF down vs PBS |
| Rbm5 | NM_148930 | RNA binding motif protein 5 | -2.12 | CNTF down vs PBS |
| Foxn3 | NM_183186 /// XM_001473733 | forkhead box N3 | -2.12 | CNTF down vs PBS |
| Dach2 | NM_001142570 /// NM_033605 | dachshund 2 (Drosophila) | -2.12 | CNTF down vs PBS |
| Rabgap1 | NM_001033960 /// NM_146121 | RAB GTPase activating protein 1 | -2.12 | CNTF down vs PBS |
| Nt5dc2 | NM_027289 | 5'-nucleotidase domain containing 2 | -2.12 | CNTF down vs PBS |
| Arglu1 | NM_176849 | arginine and glutamate rich 1 | -2.12 | CNTF down vs PBS |
| 5033430J17Rik | --- | RIKEN cDNA 5033430J17 gene | -2.12 | CNTF down vs PBS |
| Mfsd11 | NM_178620 | major facilitator superfamily domain containing 11 | -2.13 | CNTF down vs PBS |
| Gas1 | NM_008086 | growth arrest specific 1 | -2.13 | CNTF down vs PBS |
| Trim71 | NM_001042503 | tripartite motif-containing 71 | -2.13 | CNTF down vs PBS |
| B230334C09Rik | --- | RIKEN cDNA B230334C09 gene | -2.13 | CNTF down vs PBS |
| Nfib | NM_001113209 /// NM_001113210 /// NM_008687 | nuclear factor I/B | -2.13 | CNTF down vs PBS |
| Usp47 | NM_133758 | ubiquitin specific peptidase 47 | -2.13 | CNTF down vs PBS |
| B130021B11Rik | --- | RIKEN cDNA B130021B11 gene | -2.13 | CNTF down vs PBS |
| 7-Mar | NM_020575 | membrane-associated ring finger (C3HC4) 7 | -2.14 | CNTF down vs PBS |
| 6430711C07Rik | --- | RIKEN cDNA 6430711C07 gene | -2.14 | CNTF down vs PBS |
| Bclaf1 | NM_001025392 /// NM_001025393 /// NM_153787 | BCL2-associated transcription factor 1 | -2.14 | CNTF down vs PBS |
| Tpp2 | NM_009418 | tripeptidyl peptidase II | -2.14 | CNTF down vs PBS |
| AI605517 | --- | expressed sequence AI605517 | -2.15 | CNTF down vs PBS |
| B4galt4 | NM_019804 | UDP-Gal:betaGlcNAc beta 1,4-galactosyltransferase, polypeptide 4 | -2.15 | CNTF down vs PBS |
| Lhx9 | NM_001025565 /// NM_001042577 /// NM_010714 | LIM homeobox protein 9 | -2.15 | CNTF down vs PBS |
| LOC667118 | XR_035385 /// XR_035411 | zinc finger, BED domain containing 4 pseudogene | -2.16 | CNTF down vs PBS |
| Mgat4a | NM_173870 | mannoside acetylglucosaminyltransferase 4, isoenzyme A | -2.16 | CNTF down vs PBS |
| B230339M05Rik | NM_177658 | RIKEN cDNA B230339M05 gene | -2.16 | CNTF down vs PBS |
| Tial1 | NM_009383 | Tia1 cytotoxic granule-associated RNA binding protein-like 1 | -2.16 | CNTF down vs PBS |
| C130015C19 | --- | hypothetical LOC403342 | -2.16 | CNTF down vs PBS |
| C130071C03Rik | NM_177100 /// NR_015561 | RIKEN cDNA C130071C03 gene | -2.17 | CNTF down vs PBS |
| Mtm1 | NM_001164190 /// NM_001164191 /// NM_001164192 /// NM_001164193 /// NM_019926 | X-linked myotubular myopathy gene 1 | -2.17 | CNTF down vs PBS |
| Ntrk2 | NM_001025074 /// NM_008745 | neurotrophic tyrosine kinase, receptor, type 2 | -2.17 | CNTF down vs PBS |
| St13 | NM_133726 | Suppression of tumorigenicity 13 | -2.17 | CNTF down vs PBS |
| A230048O21Rik | --- | RIKEN cDNA A230048O21 gene | -2.17 | CNTF down vs PBS |
| Zbtb20 | NM_019778 | zinc finger and BTB domain containing 20 | -2.17 | CNTF down vs PBS |
| Elavl2 | NM_010486 /// NM_207685 /// NM_207686 | ELAV (embryonic lethal, abnormal vision, Drosophila)-like 2 (Hu antigen B) | -2.17 | CNTF down vs PBS |
| A930018M24Rik | XM_907235 /// XM_988947 | RIKEN cDNA A930018M24 gene | -2.17 | CNTF down vs PBS |
| Med13 | NM_001080931 | mediator complex subunit 13 | -2.18 | CNTF down vs PBS |
| Cwf19l2 | NM_027545 | CWF19-like 2, cell cycle control (S. pombe) | -2.18 | CNTF down vs PBS |
| Mrpl15 | NM_025300 | Mitochondrial ribosomal protein L15 | -2.18 | CNTF down vs PBS |
| Ryr2 | NM_023868 | ryanodine receptor 2, cardiac | -2.18 | CNTF down vs PBS |
| Zfp316 | NM_017467 | zinc finger protein 316 | -2.18 | CNTF down vs PBS |
| Ccni | NM_017367 | cyclin I | -2.18 | CNTF down vs PBS |
| Acss2 | NM_019811 | acyl-CoA synthetase short-chain family member 2 | -2.18 | CNTF down vs PBS |
| AL023051 | --- | expressed sequence AL023051 | -2.19 | CNTF down vs PBS |
| Nf1 | NM_010897 | neurofibromatosis 1 | -2.19 | CNTF down vs PBS |
| Twf1 | NM_008971 | twinfilin, actin-binding protein, homolog 1 (Drosophila) | -2.19 | CNTF down vs PBS |
| Ifnar1 | NM_010508 | interferon (alpha and beta) receptor 1 | -2.19 | CNTF down vs PBS |
| Atad1 | NM_026487 | ATPase family, AAA domain containing 1 | -2.20 | CNTF down vs PBS |
| Zfp260 | NM_011981 | zinc finger protein 260 | -2.20 | CNTF down vs PBS |
| Sms | NM_009214 | spermine synthase | -2.20 | CNTF down vs PBS |
| Xpr1 | NM_011273 | xenotropic and polytropic retrovirus receptor 1 | -2.20 | CNTF down vs PBS |
| Fmr1 | NM_008031 | fragile X mental retardation syndrome 1 homolog | -2.20 | CNTF down vs PBS |
| Dnm1l | NM_001025947 /// NM_152816 | dynamin 1-like | -2.20 | CNTF down vs PBS |
| Dpp4 | NM_001159543 /// NM_010074 | dipeptidylpeptidase 4 | -2.21 | CNTF down vs PBS |
| Scai | NM_178778 | suppressor of cancer cell invasion | -2.21 | CNTF down vs PBS |
| Ptplad1 | NM_021345 | protein tyrosine phosphatase-like A domain containing 1 | -2.21 | CNTF down vs PBS |
| Son | NM_019973 /// NM_178880 | Son DNA binding protein | -2.21 | CNTF down vs PBS |
| Lrrtm2 | NM_178005 | leucine rich repeat transmembrane neuronal 2 | -2.21 | CNTF down vs PBS |
| 2310039F13Rik | --- | RIKEN cDNA 2310039F13 gene | -2.21 | CNTF down vs PBS |
| Cpsf6 | NM_001013391 | cleavage and polyadenylation specific factor 6 | -2.22 | CNTF down vs PBS |
| Npepps | NM_008942 | aminopeptidase puromycin sensitive | -2.22 | CNTF down vs PBS |
| 5430440L12Rik | --- | RIKEN cDNA 5430440L12 gene | -2.22 | CNTF down vs PBS |
| 9130213B05Rik | NM_145562 | RIKEN cDNA 9130213B05 gene | -2.22 | CNTF down vs PBS |
| Rbmx | NM_011252 | RNA binding motif protein, X chromosome | -2.22 | CNTF down vs PBS |
| Rian | NR_028261 /// XM_901568 /// XM_922645 | RNA imprinted and accumulated in nucleus | -2.22 | CNTF down vs PBS |
| Kif1b | NM_008441 /// NM_207682 | kinesin family member 1B | -2.23 | CNTF down vs PBS |
| Cdk7 | NM_009874 | cyclin-dependent kinase 7 (homolog of Xenopus MO15 cdk-activating kinase) | -2.23 | CNTF down vs PBS |
| Cdkn1b | NM_009875 | cyclin-dependent kinase inhibitor 1B | -2.23 | CNTF down vs PBS |
| A130082M07Rik /// Tcra | XM_001471582 | RIKEN cDNA A130082M07 gene /// T-cell receptor alpha chain | -2.23 | CNTF down vs PBS |
| Trpm7 | NM_001164325 /// NM_021450 | transient receptor potential cation channel, subfamily M, member 7 | -2.24 | CNTF down vs PBS |
| Kcnip1 | NM_027398 | Kv channel-interacting protein 1 | -2.24 | CNTF down vs PBS |
| Kcnd3 | NM_001039347 /// NM_019931 | potassium voltage-gated channel, Shal-related family, member 3 | -2.24 | CNTF down vs PBS |
| Ky | NM_024291 | kyphoscoliosis peptidase | -2.24 | CNTF down vs PBS |
| LOC100044883 /// Rab3c | NM_023852 /// XM_001473410 /// XM_001473430 | similar to RAB3C, member RAS oncogene family /// RAB3C, member RAS oncogene fami | -2.25 | CNTF down vs PBS |
| Clasp1 | NM_001081276 /// NM_029709 /// NM_177548 | CLIP associating protein 1 | -2.25 | CNTF down vs PBS |
| Vav3 | NM_020505 /// NM_146139 | vav 3 oncogene | -2.25 | CNTF down vs PBS |
| Ryr3 | NM_177652 | Ryanodine receptor 3 | -2.25 | CNTF down vs PBS |
| Mtcp1 | NM_001039373 /// NM_010839 | mature T-cell proliferation 1 | -2.25 | CNTF down vs PBS |
| Fam126b | NM_172513 | family with sequence similarity 126, member B | -2.25 | CNTF down vs PBS |
| Abcb9 | NM_019875 | ATP-binding cassette, sub-family B (MDR/TAP), member 9 | -2.26 | CNTF down vs PBS |
| Mycbp2 | NM_207215 | MYC binding protein 2 | -2.26 | CNTF down vs PBS |
| Zfp398 | NM_027477 /// NM_173034 | zinc finger protein 398 | -2.26 | CNTF down vs PBS |
| C030043A13Rik | --- | RIKEN cDNA C030043A13 gene | -2.27 | CNTF down vs PBS |
| Gas7 | NM_001109657 /// NM_008088 | Growth arrest specific 7 | -2.27 | CNTF down vs PBS |
| Hdac2 | NM_008229 | histone deacetylase 2 | -2.27 | CNTF down vs PBS |
| Scrn1 | NM_027268 | secernin 1 | -2.28 | CNTF down vs PBS |
| Sfrs8 | NM_172276 | splicing factor, arginine/serine-rich 8 | -2.28 | CNTF down vs PBS |
| Strn | NM_011500 | striatin, calmodulin binding protein | -2.28 | CNTF down vs PBS |
| Sox5 | NM_001113559 /// NM_011444 | SRY-box containing gene 5 | -2.29 | CNTF down vs PBS |
| Atxn2 | NM_009125 | Ataxin 2 | -2.29 | CNTF down vs PBS |
| D030002E05Rik | --- | RIKEN cDNA D030002E05 gene | -2.29 | CNTF down vs PBS |
| Prpf39 | NM_177806 | PRP39 pre-mRNA processing factor 39 homolog (yeast) | -2.29 | CNTF down vs PBS |
| Magi1 | NM_001029850 /// NM_001083320 /// NM_001083321 /// NM_010367 | membrane associated guanylate kinase, WW and PDZ domain containing 1 | -2.29 | CNTF down vs PBS |
| Adam19 /// LOC100045780 | NM_009616 /// XM_001475019 | a disintegrin and metallopeptidase domain 19 (meltrin beta) /// similar to metal | -2.30 | CNTF down vs PBS |
| Tatdn1 | NM_175151 | TatD DNase domain containing 1 | -2.30 | CNTF down vs PBS |
| Dpp4 | NM_001159543 /// NM_010074 | dipeptidylpeptidase 4 | -2.30 | CNTF down vs PBS |
| AU014972 | --- | expressed sequence AU014972 | -2.31 | CNTF down vs PBS |
| Dlg2 | NM_011807 | discs, large homolog 2 (Drosophila) | -2.31 | CNTF down vs PBS |
| Atp6v0a1 | NM_016920 | ATPase, H+ transporting, lysosomal V0 subunit A1 | -2.31 | CNTF down vs PBS |
| Slc38a9 | NM_178746 | solute carrier family 38, member 9 | -2.32 | CNTF down vs PBS |
| Zcchc7 | NM_138590 /// NM_177027 | zinc finger, CCHC domain containing 7 | -2.32 | CNTF down vs PBS |
| Scap | NM_001001144 /// NM_001103162 | SREBF chaperone | -2.32 | CNTF down vs PBS |
| Ube2b | NM_009458 | ubiquitin-conjugating enzyme E2B, RAD6 homology (S. cerevisiae) | -2.33 | CNTF down vs PBS |
| 9430019C24Rik | --- | RIKEN cDNA 9430019C24 gene | -2.34 | CNTF down vs PBS |
| Clmn | NM_001040682 /// NM_053155 | calmin | -2.34 | CNTF down vs PBS |
| Csrp3 | NM_013808 | cysteine and glycine-rich protein 3 | -2.34 | CNTF down vs PBS |
| AI314760 | --- | expressed sequence AI314760 | -2.34 | CNTF down vs PBS |
| A330076H08Rik | NR_015599 | RIKEN cDNA A330076H08 gene | -2.34 | CNTF down vs PBS |
| 2610011E03Rik | --- | RIKEN cDNA 2610011E03 gene | -2.34 | CNTF down vs PBS |
| Kcnj6 | NM_001025584 /// NM_001025585 /// NM_001025590 /// NM_010606 | potassium inwardly-rectifying channel, subfamily J, member 6 | -2.34 | CNTF down vs PBS |
| Napb | NM_019632 | N-ethylmaleimide sensitive fusion protein attachment protein beta | -2.34 | CNTF down vs PBS |
| Ggnbp2 | NM_153144 | gametogenetin binding protein 2 | -2.35 | CNTF down vs PBS |
| Luc7l2 | NM_138680 | LUC7-like 2 (S. cerevisiae) | -2.35 | CNTF down vs PBS |
| Synj2bp | NM_025292 | synaptojanin 2 binding protein | -2.36 | CNTF down vs PBS |
| Nrip3 | NM_020610 | nuclear receptor interacting protein 3 | -2.36 | CNTF down vs PBS |
| Pax6 | NM_013627 | paired box gene 6 | -2.36 | CNTF down vs PBS |
| Taf15 | NM_027427 | TAF15 RNA polymerase II, TATA box binding protein (TBP)-associated factor | -2.36 | CNTF down vs PBS |
| Baz2b | NM_001001182 | bromodomain adjacent to zinc finger domain, 2B | -2.37 | CNTF down vs PBS |
| Foxn2 | NM_180974 | forkhead box N2 | -2.38 | CNTF down vs PBS |
| D3Ertd108e | --- | DNA segment, Chr 3, ERATO Doi 108, expressed | -2.39 | CNTF down vs PBS |
| Slit2 | NM_178804 | slit homolog 2 (Drosophila) | -2.39 | CNTF down vs PBS |
| Kcnip1 | NM_027398 | Kv channel-interacting protein 1 | -2.39 | CNTF down vs PBS |
| 4632427E13Rik | NR_015510 /// XR_035233 /// XR_035450 | RIKEN cDNA 4632427E13 gene | -2.39 | CNTF down vs PBS |
| LOC552904 | --- | hypothetical LOC552904 | -2.39 | CNTF down vs PBS |
| Kif1b | NM_008441 /// NM_207682 | kinesin family member 1B | -2.40 | CNTF down vs PBS |
| Pdyn | NM_018863 | prodynorphin | -2.40 | CNTF down vs PBS |
| 9330199C07Rik | --- | RIKEN cDNA 9330199C07 gene | -2.40 | CNTF down vs PBS |
| Magi1 | NM_001029850 /// NM_001083320 /// NM_001083321 /// NM_010367 | membrane associated guanylate kinase, WW and PDZ domain containing 1 | -2.40 | CNTF down vs PBS |
| LOC100048559 /// Sfrs1 | NM_001078167 /// NM_173374 /// XM_001480897 | similar to splicing factor, arginine/serine-rich 1 (splicing factor 2, alternate | -2.41 | CNTF down vs PBS |
| Clock | NM_007715 | circadian locomoter output cycles kaput | -2.41 | CNTF down vs PBS |
| Zfp277 | NM_172575 /// NM_178845 | zinc finger protein 277 | -2.41 | CNTF down vs PBS |
| Slc8a1 | NM_001112798 /// NM_011406 | solute carrier family 8 (sodium/calcium exchanger), member 1 | -2.42 | CNTF down vs PBS |
| 8030462N17Rik | NM_178670 | RIKEN cDNA 8030462N17 gene | -2.44 | CNTF down vs PBS |
| 9330177L23Rik | --- | RIKEN cDNA 9330177L23 gene | -2.45 | CNTF down vs PBS |
| Pax6 | NM_013627 | paired box gene 6 | -2.45 | CNTF down vs PBS |
| Slc4a7 | NM_001033270 | solute carrier family 4, sodium bicarbonate cotransporter, member 7 | -2.46 | CNTF down vs PBS |
| Ddi2 | NM_001017966 | DNA-damage inducible protein 2 | -2.47 | CNTF down vs PBS |
| Zfp509 | NM_029162 | zinc finger protein 509 | -2.47 | CNTF down vs PBS |
| Cdh10 | NM_009865 | cadherin 10 | -2.47 | CNTF down vs PBS |
| Gdap1 | NM_010267 | ganglioside-induced differentiation-associated-protein 1 | -2.48 | CNTF down vs PBS |
| Cyp26b1 | NM_175475 | cytochrome P450, family 26, subfamily b, polypeptide 1 | -2.48 | CNTF down vs PBS |
| Taok1 | NM_144825 | TAO kinase 1 | -2.48 | CNTF down vs PBS |
| Dnm3 | NM_001038619 /// NM_172646 | dynamin 3 | -2.49 | CNTF down vs PBS |
| AU014876 | --- | expressed sequence AU014876 | -2.50 | CNTF down vs PBS |
| 3110047M12Rik | --- | RIKEN cDNA 3110047M12 gene | -2.51 | CNTF down vs PBS |
| Slc6a1 | NM_178703 | solute carrier family 6 (neurotransmitter transporter, GABA), member 1 | -2.51 | CNTF down vs PBS |
| Gm4210 | XM_001477784 /// XM_001479445 | Predicted gene 4210 | -2.52 | CNTF down vs PBS |
| Irs4 | NM_010572 | insulin receptor substrate 4 | -2.53 | CNTF down vs PBS |
| Sdcbp | NM_001098227 /// NM_016807 | syndecan binding protein | -2.53 | CNTF down vs PBS |
| Flnb | NM_134080 /// XM_001471989 /// XM_001472027 /// XM_001478129 /// XM_001478141 // | Filamin, beta | -2.53 | CNTF down vs PBS |
| Gpc6 | NM_001079844 /// NM_011821 | glypican 6 | -2.54 | CNTF down vs PBS |
| Tpp2 | NM_009418 | tripeptidyl peptidase II | -2.55 | CNTF down vs PBS |
| Ndufa12 | NM_025551 | NADH dehydrogenase (ubiquinone) 1 alpha subcomplex, 12 | -2.55 | CNTF down vs PBS |
| Fam59a | NM_001033445 | family with sequence similarity 59, member A | -2.56 | CNTF down vs PBS |
| Tia1 | NM_001164078 /// NM_001164079 /// NM_011585 | cytotoxic granule-associated RNA binding protein 1 | -2.56 | CNTF down vs PBS |
| Braf | NM_139294 | Braf transforming gene | -2.56 | CNTF down vs PBS |
| Vps41 | NM_172120 | vacuolar protein sorting 41 (yeast) | -2.57 | CNTF down vs PBS |
| Rbm25 | NM_025930 /// NM_027349 | RNA binding motif protein 25 | -2.57 | CNTF down vs PBS |
| Htt | NM_010414 | huntingtin | -2.58 | CNTF down vs PBS |
| Pcgf6 | NM_027654 | polycomb group ring finger 6 | -2.58 | CNTF down vs PBS |
| 6430704M03Rik | NM_001142965 /// XR_001563 /// XR_001596 | RIKEN cDNA 6430704M03 gene | -2.59 | CNTF down vs PBS |
| Glb1l3 | NM_001113323 /// XM_001476959 /// XM_983469 | galactosidase, beta 1 like 3 | -2.59 | CNTF down vs PBS |
| Arhgef11 | NM_001003912 | Rho guanine nucleotide exchange factor (GEF) 11 | -2.60 | CNTF down vs PBS |
| Rab2b | NM_172601 | RAB2B, member RAS oncogene family | -2.61 | CNTF down vs PBS |
| Fam19a3 | NM_183224 | family with sequence similarity 19, member A3 | -2.61 | CNTF down vs PBS |
| Rorb | NM_001043354 /// NM_146095 | RAR-related orphan receptor beta | -2.62 | CNTF down vs PBS |
| E530001K10Rik | XM_001478872 /// XM_001479701 | RIKEN cDNA E530001K10 gene | -2.62 | CNTF down vs PBS |
| Bcr | NM_001081412 | breakpoint cluster region | -2.62 | CNTF down vs PBS |
| LOC676974 | XM_001003154 | similar to Glucose-6-phosphate isomerase (GPI) (Phosphoglucose isomerase) (PGI) | -2.63 | CNTF down vs PBS |
| Rph3a | NM_011286 | rabphilin 3A | -2.64 | CNTF down vs PBS |
| Gdap1 | NM_010267 | ganglioside-induced differentiation-associated-protein 1 | -2.65 | CNTF down vs PBS |
| Cd247 | NM_001113391 /// NM_001113392 /// NM_001113393 /// NM_001113394 /// NM_031162 | CD247 antigen | -2.67 | CNTF down vs PBS |
| Dnm3 | NM_001038619 /// NM_172646 | dynamin 3 | -2.68 | CNTF down vs PBS |
| Cpsf7 | NM_001164272 /// NM_172302 | cleavage and polyadenylation specific factor 7 | -2.68 | CNTF down vs PBS |
| Zfp451 | NM_133817 | zinc finger protein 451 | -2.68 | CNTF down vs PBS |
| 5033421C21Rik | --- | RIKEN cDNA 5033421C21 gene | -2.69 | CNTF down vs PBS |
| Map3k2 | NM_011946 | mitogen-activated protein kinase kinase kinase 2 | -2.70 | CNTF down vs PBS |
| Ptger3 | NM_011196 | prostaglandin E receptor 3 (subtype EP3) | -2.71 | CNTF down vs PBS |
| Ptprd | NM_001014288 /// NM_011211 | protein tyrosine phosphatase, receptor type, D | -2.72 | CNTF down vs PBS |
| Sec62 | NM_027016 | SEC62 homolog (S. cerevisiae) | -2.72 | CNTF down vs PBS |
| Atp6v1b2 | NM_007509 | ATPase, H+ transporting, lysosomal V1 subunit B2 | -2.72 | CNTF down vs PBS |
| 1700080N15Rik | XM_001472795 /// XM_001474419 | RIKEN cDNA 1700080N15 gene | -2.75 | CNTF down vs PBS |
| Scn2b | NM_001014761 | sodium channel, voltage-gated, type II, beta | -2.76 | CNTF down vs PBS |
| Clmn | NM_001040682 /// NM_053155 | calmin | -2.76 | CNTF down vs PBS |
| Kcnma1 | NM_010610 | Potassium large conductance calcium-activated channel, subfamily M, alpha member | -2.77 | CNTF down vs PBS |
| 1110032A04Rik | NM_001164210 /// NM_133675 | RIKEN cDNA 1110032A04 gene | -2.77 | CNTF down vs PBS |
| Lphn3 | NM_198702 | latrophilin 3 | -2.77 | CNTF down vs PBS |
| Pak3 | NM_008778 | p21 protein (Cdc42/Rac)-activated kinase 3 | -2.78 | CNTF down vs PBS |
| Shc3 | NM_009167 | Src homology 2 domain-containing transforming protein C3 | -2.78 | CNTF down vs PBS |
| A230070E04Rik | --- | RIKEN cDNA A230070E04 gene | -2.78 | CNTF down vs PBS |
| Smg1 | NM_001031814 | SMG1 homolog, phosphatidylinositol 3-kinase-related kinase (C. elegans) | -2.79 | CNTF down vs PBS |
| Saps3 | NM_001164159 /// NM_028999 /// NM_029456 | SAPS domain family, member 3 | -2.82 | CNTF down vs PBS |
| Ttc14 | NM_025978 /// NM_027619 | tetratricopeptide repeat domain 14 | -2.83 | CNTF down vs PBS |
| 4732460I02Rik | --- | RIKEN cDNA 4732460I02 gene | -2.83 | CNTF down vs PBS |
| Rbm5 | NM_148930 | RNA binding motif protein 5 | -2.85 | CNTF down vs PBS |
| Dcaf12 | NM_026893 | DDB1 and CUL4 associated factor 12 | -2.86 | CNTF down vs PBS |
| Vopp1 | NM_146168 | Vesicular, overexpressed in cancer, prosurvival protein 1 | -2.87 | CNTF down vs PBS |
| Cabp5 | NM_013877 | calcium binding protein 5 | -2.88 | CNTF down vs PBS |
| St8sia2 | NM_009181 | ST8 alpha-N-acetyl-neuraminide alpha-2,8-sialyltransferase 2 | -2.89 | CNTF down vs PBS |
| Epha3 | NM_010140 | Eph receptor A3 | -2.91 | CNTF down vs PBS |
| Adamts18 | NM_172466 | a disintegrin-like and metallopeptidase (reprolysin type) with thrombospondin ty | -2.96 | CNTF down vs PBS |
| Gpr158 | NM_001004761 | G protein-coupled receptor 158 | -2.98 | CNTF down vs PBS |
| 5330430B06Rik | --- | RIKEN cDNA 5330430B06 gene | -2.99 | CNTF down vs PBS |
| Ccnt2 | NM_028399 | cyclin T2 | -3.00 | CNTF down vs PBS |
| Nfia | NM_001122952 /// NM_001122953 /// NM_010905 | nuclear factor I/A | -3.04 | CNTF down vs PBS |
| Stx1b | NM_024414 | syntaxin 1B | -3.10 | CNTF down vs PBS |
| Efr3a | NM_133766 | EFR3 homolog A (S. cerevisiae) | -3.13 | CNTF down vs PBS |
| Ccnl2 | NM_207678 | cyclin L2 | -3.13 | CNTF down vs PBS |
| B4galt6 | NM_019737 | UDP-Gal:betaGlcNAc beta 1,4-galactosyltransferase, polypeptide 6 | -3.13 | CNTF down vs PBS |
| Uba6 | NM_172712 | ubiquitin-like modifier activating enzyme 6 | -3.16 | CNTF down vs PBS |
| Ints10 | NM_027590 | integrator complex subunit 10 | -3.18 | CNTF down vs PBS |
| 9130009I01Rik | --- | RIKEN cDNA 9130009I01 gene | -3.27 | CNTF down vs PBS |
| Nfib | NM_001113209 /// NM_001113210 /// NM_008687 | nuclear factor I/B | -3.32 | CNTF down vs PBS |
| Ldb2 | NM_001077398 /// NM_010698 | LIM domain binding 2 | -3.32 | CNTF down vs PBS |
| Ndst3 | NM_031186 | N-deacetylase/N-sulfotransferase (heparan glucosaminyl) 3 | -3.48 | CNTF down vs PBS |
| Arhgef9 | NM_001033329 | CDC42 guanine nucleotide exchange factor (GEF) 9 | -3.48 | CNTF down vs PBS |
| 6030438J01 | --- | hypothetical protein 6030438J01 | -3.60 | CNTF down vs PBS |
| Cdh10 | NM_009865 | cadherin 10 | -3.61 | CNTF down vs PBS |
| A930009L07Rik | --- | RIKEN cDNA A930009L07 gene | -3.95 | CNTF down vs PBS |
| 5730526G10Rik | --- | RIKEN cDNA 5730526G10 gene | -4.06 | CNTF down vs PBS |
| Scd2 | NM_009128 | stearoyl-Coenzyme A desaturase 2 | -4.37 | CNTF down vs PBS |
| 9.13E+15 | --- | hypothetical 9130022E09 | -4.40 | CNTF down vs PBS |
| Mapk8 | NM_016700 | mitogen-activated protein kinase 8 | -4.44 | CNTF down vs PBS |
| Inpp4b | NM_001024617 | inositol polyphosphate-4-phosphatase, type II | -4.54 | CNTF down vs PBS |
| Opn1sw | NM_007538 | opsin 1 (cone pigments), short-wave-sensitive (color blindness, tritan) | -5.00 | CNTF down vs PBS |
| Opn1sw | NM_007538 | opsin 1 (cone pigments), short-wave-sensitive (color blindness, tritan) | -5.19 | CNTF down vs PBS |
