# Supplementary material for: Ciliary Neurotrophic Factor Induces Genes Associated with Inflammation and Gliosis in the Retina: A Gene Profiling Study of Flow-Sorted, Müller Cells
Source: PLoS One. 2011 May 26;6(5):e20326. doi: 10.1371/journal.pone.0020326 (PMC3102695; doi:10.1371/journal.pone.0020326)
Supplement: Table S2 — Supporting table. (DOC [file pone.0020326.s002.doc]

| **Gene Symbol** | **RefSeq** | **gene_assignment** | **Fold-Change(CNTF vs. PBS)** | **Fold-Change(CNTF vs. PBS) (Description)** |
| --- | --- | --- | --- | --- |
| Uts2 | NM_011910 | NM_011910 // Uts2 // urotensin 2 // 4 E1 // 24111 /// ENSMUST00000030803 // Uts2 | -2.39 | CNTF down vs PBS |
| Rod1 | NM_144904 | NM_144904 // Rod1 // ROD1 regulator of differentiation 1 (S. pombe) // 4 B3 // 2 | 5.25 | CNTF up vs PBS |
| Gsg1 | NM_010352 | NM_010352 // Gsg1 // germ cell-specific gene 1 // 6 G1|6 66.3 cM // 14840 /// NM | -2.21 | CNTF down vs PBS |
| Ppp1ca | NM_031868 | NM_031868 // Ppp1ca // protein phosphatase 1, catalytic subunit, alpha isoform / | 4.35 | CNTF up vs PBS |
| Slc25a11 | NM_024211 | NM_024211 // Slc25a11 // solute carrier family 25 (mitochondrial carrier oxoglut | 2.55 | CNTF up vs PBS |
| Esyt1 | NM_011843 | NM_011843 // Esyt1 // extended synaptotagmin-like protein 1 // 10 D3 // 23943 // | 2.37 | CNTF up vs PBS |
| Ndufb8 | NM_026061 | NM_026061 // Ndufb8 // NADH dehydrogenase (ubiquinone) 1 beta subcomplex 8 // 19 | 2.05 | CNTF up vs PBS |
| Mfsd1 | NM_025813 | NM_025813 // Mfsd1 // major facilitator superfamily domain containing 1 // 3 E2 | 5.70 | CNTF up vs PBS |
| Dstn | NM_019771 | NM_019771 // Dstn // destrin // 2 H1|2 81.4 cM // 56431 /// ENSMUST00000103172 / | 3.02 | CNTF up vs PBS |
| Spg21 | NM_138584 | NM_138584 // Spg21 // spastic paraplegia 21 homolog (human) // 9 C|9 40.0 cM // | 3.10 | CNTF up vs PBS |
| Tmem49 | NM_029478 | NM_029478 // Tmem49 // transmembrane protein 49 // 11 C // 75909 /// ENSMUST0000 | 3.20 | CNTF up vs PBS |
| Snx6 | NM_026998 | NM_026998 // Snx6 // sorting nexin 6 // 12 C1 // 72183 /// ENSMUST00000005798 // | 2.14 | CNTF up vs PBS |
| Msn | NM_010833 | NM_010833 // Msn // moesin // X C3 // 17698 /// ENSMUST00000117399 // Msn // moe | 5.72 | CNTF up vs PBS |
| Tiparp | NM_178892 | NM_178892 // Tiparp // TCDD-inducible poly(ADP-ribose) polymerase // 3 E1 // 999 | 2.17 | CNTF up vs PBS |
| Gtf2b | NM_145546 | NM_145546 // Gtf2b // general transcription factor IIB // 3 H1 // 229906 /// ENS | 4.09 | CNTF up vs PBS |
| Rap1b | NM_024457 | NM_024457 // Rap1b // RAS related protein 1b // 10 D2 // 215449 /// BC033382 // | 4.66 | CNTF up vs PBS |
| Anxa4 | NM_013471 | NM_013471 // Anxa4 // annexin A4 // 6 D1|6 38.0 cM // 11746 /// ENSMUST000001136 | 4.07 | CNTF up vs PBS |
| Csf2rb | NM_007780 | NM_007780 // Csf2rb // colony stimulating factor 2 receptor, beta, low-affinity | 3.62 | CNTF up vs PBS |
| Rab1 | NM_008996 | NM_008996 // Rab1 // RAB1, member RAS oncogene family // 11 A3.1|11 10.9 cM // 1 | 2.76 | CNTF up vs PBS |
| Arhgdib | NM_007486 | NM_007486 // Arhgdib // Rho, GDP dissociation inhibitor (GDI) beta // 6 G1 // 11 | 22.95 | CNTF up vs PBS |
| Trappc2l | NM_021502 | NM_021502 // Trappc2l // trafficking protein particle complex 2-like // 8 E1 // | 3.04 | CNTF up vs PBS |
| Rap2c | NM_172413 | NM_172413 // Rap2c // RAP2C, member of RAS oncogene family // X A5 // 72065 /// | 4.96 | CNTF up vs PBS |
| Snx2 | NM_026386 | NM_026386 // Snx2 // sorting nexin 2 // 18 D1 // 67804 /// ENSMUST00000037850 // | 2.36 | CNTF up vs PBS |
| Bglap2 | NM_001032298 | NM_001032298 // Bglap2 // bone gamma-carboxyglutamate protein 2 // 3 F1|3 42.6 c | -2.23 | CNTF down vs PBS |
| Sh3glb2 | NM_139302 | NM_139302 // Sh3glb2 // SH3-domain GRB2-like endophilin B2 // 2 B // 227700 /// | -2.12 | CNTF down vs PBS |
| Mmp14 | NM_008608 | NM_008608 // Mmp14 // matrix metallopeptidase 14 (membrane-inserted) // 14 C2|14 | 2.49 | CNTF up vs PBS |
| Tmem123 | NM_133739 | NM_133739 // Tmem123 // transmembrane protein 123 // 9 A1 // 71929 /// ENSMUST00 | 3.86 | CNTF up vs PBS |
| Lilrb4 | NM_013532 | NM_013532 // Lilrb4 // leukocyte immunoglobulin-like receptor, subfamily B, memb | 146.53 | CNTF up vs PBS |
| Lims1 | NM_026148 | NM_026148 // Lims1 // LIM and senescent cell antigen-like domains 1 // 10 B4|10 | 2.41 | CNTF up vs PBS |
| St6gal1 | NM_145933 | NM_145933 // St6gal1 // beta galactoside alpha 2,6 sialyltransferase 1 // 16 B1| | 2.24 | CNTF up vs PBS |
| Rexo2 | NM_024233 | NM_024233 // Rexo2 // REX2, RNA exonuclease 2 homolog (S. cerevisiae) // 9 A5.3 | 4.61 | CNTF up vs PBS |
| Pycard | NM_023258 | NM_023258 // Pycard // PYD and CARD domain containing // 7 F4 // 66824 /// ENSMU | 5.10 | CNTF up vs PBS |
| Gars | NM_180678 | NM_180678 // Gars // glycyl-tRNA synthetase // 6 B3|6 32.5 cM // 353172 /// ENSM | 2.51 | CNTF up vs PBS |
| Eif2s3x | NM_012010 | NM_012010 // Eif2s3x // eukaryotic translation initiation factor 2, subunit 3, s | 3.28 | CNTF up vs PBS |
| Lrp1 | NM_008512 | NM_008512 // Lrp1 // low density lipoprotein receptor-related protein 1 // 10 B2 | 2.02 | CNTF up vs PBS |
| Alox5ap | NM_009663 | NM_009663 // Alox5ap // arachidonate 5-lipoxygenase activating protein // --- // | 18.66 | CNTF up vs PBS |
| Cdv3 | NM_175565 | NM_175565 // Cdv3 // carnitine deficiency-associated gene expressed in ventricle | 2.34 | CNTF up vs PBS |
| Cd53 | NM_007651 | NM_007651 // Cd53 // CD53 antigen // 3 F2.3|3 50.5 cM // 12508 /// ENSMUST000000 | 20.38 | CNTF up vs PBS |
| Mgat4a | NM_173870 | NM_173870 // Mgat4a // mannoside acetylglucosaminyltransferase 4, isoenzyme A // | 3.59 | CNTF up vs PBS |
| Lamp1 | NM_010684 | NM_010684 // Lamp1 // lysosomal-associated membrane protein 1 // 8 A1.1|8 1.0 cM | 5.20 | CNTF up vs PBS |
| Snap23 | NM_009222 | NM_009222 // Snap23 // synaptosomal-associated protein 23 // 2 E5|2 61.8 cM // 2 | 3.78 | CNTF up vs PBS |
| Akr1a4 | NM_021473 | NM_021473 // Akr1a4 // aldo-keto reductase family 1, member A4 (aldehyde reducta | 7.52 | CNTF up vs PBS |
| Gnb2l1 | NM_008143 | NM_008143 // Gnb2l1 // guanine nucleotide binding protein (G protein), beta poly | 3.55 | CNTF up vs PBS |
| Arpc3 | NM_019824 | NM_019824 // Arpc3 // actin related protein 2/3 complex, subunit 3 // 5 F // 563 | 8.34 | CNTF up vs PBS |
| Sh3bp2 | NM_001145859 | NM_001145859 // Sh3bp2 // SH3-domain binding protein 2 // 5 B2 // 24055 /// NM_0 | 2.35 | CNTF up vs PBS |
| Sepx1 | NM_013759 | NM_013759 // Sepx1 // selenoprotein X 1 // 17 A3.3|17 10.0 cM // 27361 /// ENSMU | 3.86 | CNTF up vs PBS |
| C1qb | NM_009777 | NM_009777 // C1qb // complement component 1, q subcomponent, beta polypeptide // | 3.72 | CNTF up vs PBS |
| Dcaf12 | NM_026893 | NM_026893 // Dcaf12 // DDB1 and CUL4 associated factor 12 // 4 B1 // 68970 /// E | 2.11 | CNTF up vs PBS |
| Rpl8 | NM_012053 | NM_012053 // Rpl8 // ribosomal protein L8 // 15 E1 // 26961 /// ENSMUST000000040 | 2.62 | CNTF up vs PBS |
| Abca9 | NM_147220 | NM_147220 // Abca9 // ATP-binding cassette, sub-family A (ABC1), member 9 // 11 | 2.24 | CNTF up vs PBS |
| Ufc1 | NM_025388 | NM_025388 // Ufc1 // ubiquitin-fold modifier conjugating enzyme 1 // 1 H3|1 93.0 | 4.13 | CNTF up vs PBS |
| Gatm | NM_025961 | NM_025961 // Gatm // glycine amidinotransferase (L-arginine:glycine amidinotrans | 11.27 | CNTF up vs PBS |
| Igsf6 | NM_030691 | NM_030691 // Igsf6 // immunoglobulin superfamily, member 6 // 7 F2-F3 // 80719 / | 6.85 | CNTF up vs PBS |
| Gpr137b-ps | NR_003568 | NR_003568 // Gpr137b-ps // G protein-coupled receptor 137B, pseudogene // 13 A1 | 4.66 | CNTF up vs PBS |
| Rab8b | NM_173413 | NM_173413 // Rab8b // RAB8B, member RAS oncogene family // 9 C // 235442 /// ENS | 4.29 | CNTF up vs PBS |
| Stx12 | NM_133887 | NM_133887 // Stx12 // syntaxin 12 // 4 D2.3|4 60.0 cM // 100226 /// ENSMUST00000 | 2.03 | CNTF up vs PBS |
| Rhoa | NM_016802 | NM_016802 // Rhoa // ras homolog gene family, member A // 9 F2 // 11848 /// ENSM | 3.20 | CNTF up vs PBS |
| 9030624J02Rik | BC054720 | BC054720 // 9030624J02Rik // RIKEN cDNA 9030624J02 gene // 7 F3 // 71517 /// BC0 | 2.11 | CNTF up vs PBS |
| Olfr739 | NM_146668 | NM_146668 // Olfr739 // olfactory receptor 739 // --- // 258663 /// ENSMUST00000 | -2.35 | CNTF down vs PBS |
| Cap1 | NM_007598 | NM_007598 // Cap1 // CAP, adenylate cyclase-associated protein 1 (yeast) // 4 D1 | 4.13 | CNTF up vs PBS |
| Cml4 | NM_023455 | NM_023455 // Cml4 // camello-like 4 // 6 C3 // 68396 /// ENSMUST00000032073 // C | -2.25 | CNTF down vs PBS |
| Sqrdl | NM_021507 | NM_021507 // Sqrdl // sulfide quinone reductase-like (yeast) // 2 F2 // 59010 // | 3.09 | CNTF up vs PBS |
| G6pdx | NM_008062 | NM_008062 // G6pdx // glucose-6-phosphate dehydrogenase X-linked // X A2-A3|X 30 | 3.36 | CNTF up vs PBS |
| Smarcd3 | NM_025891 | NM_025891 // Smarcd3 // SWI/SNF related, matrix associated, actin dependent regu | -2.09 | CNTF down vs PBS |
| C3ar1 | NM_009779 | NM_009779 // C3ar1 // complement component 3a receptor 1 // 6 F1 // 12267 /// EN | 20.48 | CNTF up vs PBS |
| Hsp90ab1 | NM_008302 | NM_008302 // Hsp90ab1 // heat shock protein 90 alpha (cytosolic), class B member | 2.52 | CNTF up vs PBS |
| Pgd | NM_001081274 | NM_001081274 // Pgd // phosphogluconate dehydrogenase // 4 E2|4 77.6 cM // 11020 | 4.03 | CNTF up vs PBS |
| Gabarap | NM_019749 | NM_019749 // Gabarap // gamma-aminobutyric acid receptor associated protein // 1 | 5.92 | CNTF up vs PBS |
| Lgmn | NM_011175 | NM_011175 // Lgmn // legumain // 12 E // 19141 /// ENSMUST00000021607 // Lgmn // | 9.18 | CNTF up vs PBS |
| Man2a1 | NM_008549 | NM_008549 // Man2a1 // mannosidase 2, alpha 1 // 17 E1.2 // 17158 /// ENSMUST000 | 6.20 | CNTF up vs PBS |
| Fcer1g | NM_010185 | NM_010185 // Fcer1g // Fc receptor, IgE, high affinity I, gamma polypeptide // 1 | 9.39 | CNTF up vs PBS |
| Supt4h1 | NM_009296 | NM_009296 // Supt4h1 // suppressor of Ty 4 homolog 1 (S. cerevisiae) // 11 C|11 | 5.72 | CNTF up vs PBS |
| Hrnr | NM_133698 | NM_133698 // Hrnr // hornerin // --- // 68723 /// ENSMUST00000090856 // Hrnr // | -2.47 | CNTF down vs PBS |
| Hpgds | NM_019455 | NM_019455 // Hpgds // hematopoietic prostaglandin D synthase // 6 D-E // 54486 / | 10.00 | CNTF up vs PBS |
| Rlim | NM_011276 | NM_011276 // Rlim // ring finger protein, LIM domain interacting // X D|X 45.0 c | 3.85 | CNTF up vs PBS |
| Hexa | NM_010421 | NM_010421 // Hexa // hexosaminidase A // 9 B|9 29.0 cM // 15211 /// ENSMUST00000 | 4.52 | CNTF up vs PBS |
| Npc1 | NM_008720 | NM_008720 // Npc1 // Niemann Pick type C1 // 18 A1|18 4.0 cM // 18145 /// ENSMUS | 2.10 | CNTF up vs PBS |
| 2810407C02Rik | NM_001040396 | NM_001040396 // 2810407C02Rik // RIKEN cDNA 2810407C02 gene // 3 D // 69227 /// | 3.24 | CNTF up vs PBS |
| Skil | NM_011386 | NM_011386 // Skil // SKI-like // 3 A3|3 13.0 cM // 20482 /// NM_001039090 // Ski | 2.79 | CNTF up vs PBS |
| Mgst1 | NM_019946 | NM_019946 // Mgst1 // microsomal glutathione S-transferase 1 // 6 G1 // 56615 // | 4.73 | CNTF up vs PBS |
| Asna1 | NM_019652 | NM_019652 // Asna1 // arsA arsenite transporter, ATP-binding, homolog 1 (bacteri | 2.11 | CNTF up vs PBS |
| Dck | NM_007832 | NM_007832 // Dck // deoxycytidine kinase // --- // 13178 /// ENSMUST00000031311 | 2.25 | CNTF up vs PBS |
| Gdap2 | NM_010269 | NM_010269 // Gdap2 // ganglioside-induced differentiation-associated-protein 2 / | 2.19 | CNTF up vs PBS |
| Clta | NM_001080385 | NM_001080385 // Clta // clathrin, light polypeptide (Lca) // --- // 12757 /// NM | 2.27 | CNTF up vs PBS |
| Bax | NM_007527 | NM_007527 // Bax // BCL2-associated X protein // 7 B5|7 23.0 cM // 12028 /// ENS | 2.14 | CNTF up vs PBS |
| Spty2d1 | NM_175318 | NM_175318 // Spty2d1 // SPT2, Suppressor of Ty, domain containing 1 (S. cerevisi | 2.27 | CNTF up vs PBS |
| Man2b1 | NM_010764 | NM_010764 // Man2b1 // mannosidase 2, alpha B1 // 8 C2|8 37.0 cM // 17159 /// EN | 3.18 | CNTF up vs PBS |
| Pdia6 | NM_027959 | NM_027959 // Pdia6 // protein disulfide isomerase associated 6 // 12 A1.1 // 718 | 3.19 | CNTF up vs PBS |
| Sepw1 | NM_009156 | NM_009156 // Sepw1 // selenoprotein W, muscle 1 // 7 A2 // 20364 /// ENSMUST0000 | 5.82 | CNTF up vs PBS |
| Arpc5 | NM_026369 | NM_026369 // Arpc5 // actin related protein 2/3 complex, subunit 5 // 1 A3 // 67 | 3.60 | CNTF up vs PBS |
| Cstb | NM_007793 | NM_007793 // Cstb // cystatin B // 10 C1|10 42.0 cM // 13014 /// ENSMUST00000005 | 4.22 | CNTF up vs PBS |
| Speer4f | NM_027609 | NM_027609 // Speer4f // spermatogenesis associated glutamate (E)-rich protein 4f | -2.85 | CNTF down vs PBS |
| Vamp8 | NM_016794 | NM_016794 // Vamp8 // vesicle-associated membrane protein 8 // 6 C1|6 31.5 cM // | 7.84 | CNTF up vs PBS |
| Atp5b | NM_016774 | NM_016774 // Atp5b // ATP synthase, H+ transporting mitochondrial F1 complex, be | 2.68 | CNTF up vs PBS |
| Guca2a | NM_008190 | NM_008190 // Guca2a // guanylate cyclase activator 2a (guanylin) // 4 D2.1|4 57. | -2.00 | CNTF down vs PBS |
| Atp6v1a | NM_007508 | NM_007508 // Atp6v1a // ATPase, H+ transporting, lysosomal V1 subunit A // 16 B4 | 4.84 | CNTF up vs PBS |
| Crispld1 | NM_031402 | NM_031402 // Crispld1 // cysteine-rich secretory protein LCCL domain containing | -2.02 | CNTF down vs PBS |
| Tcn2 | NM_015749 | NM_015749 // Tcn2 // transcobalamin 2 // 11 A1|11 3.0 cM // 21452 /// NM_0011304 | 2.74 | CNTF up vs PBS |
| Gla | NM_013463 | NM_013463 // Gla // galactosidase, alpha // X E-F1|X 53.0 cM // 11605 /// ENSMUS | 9.54 | CNTF up vs PBS |
| Myd116 | NM_008654 | NM_008654 // Myd116 // myeloid differentiation primary response gene 116 // 7 B4 | 2.09 | CNTF up vs PBS |
| AB124611 | AB124611 | AB124611 // AB124611 // cDNA sequence AB124611 // 9 A3 // 382062 /// AB124612 // | 4.81 | CNTF up vs PBS |
| 4933400A11Rik | NR_003635 | NR_003635 // 4933400A11Rik // capping protein (actin filament) muscle Z-line, al | -2.28 | CNTF down vs PBS |
| Osbpl8 | NM_175489 | NM_175489 // Osbpl8 // oxysterol binding protein-like 8 // 10 D1 // 237542 /// N | 2.02 | CNTF up vs PBS |
| Apobec1 | NM_031159 | NM_031159 // Apobec1 // apolipoprotein B mRNA editing enzyme, catalytic polypept | 7.09 | CNTF up vs PBS |
| Cat | NM_009804 | NM_009804 // Cat // catalase // 2 E3|2 57.0 cM // 12359 /// ENSMUST00000028610 / | 2.79 | CNTF up vs PBS |
| Stxbp3a | NM_011504 | NM_011504 // Stxbp3a // syntaxin binding protein 3A // 3 F3 // 20912 /// NM_0010 | 2.49 | CNTF up vs PBS |
| Txndc14 | NM_025868 | NM_025868 // Txndc14 // thioredoxin domain containing 14 // 2 D // 66958 /// BC1 | 3.32 | CNTF up vs PBS |
| Snrpa1 | NM_021336 | NM_021336 // Snrpa1 // small nuclear ribonucleoprotein polypeptide A' // 7 C // | 2.75 | CNTF up vs PBS |
| Calr | NM_007591 | NM_007591 // Calr // calreticulin // 8 C3|8 37.0 cM // 12317 /// ENSMUST00000003 | 2.64 | CNTF up vs PBS |
| Seh1l | NM_001039088 | NM_001039088 // Seh1l // SEH1-like (S. cerevisiae // 18 E1 // 72124 /// NM_02811 | 2.46 | CNTF up vs PBS |
| Gpbp1l1 | NM_029868 | NM_029868 // Gpbp1l1 // GC-rich promoter binding protein 1-like 1 // 4 C7 // 771 | 2.15 | CNTF up vs PBS |
| D15Ertd621e | BC070426 | BC070426 // D15Ertd621e // DNA segment, Chr 15, ERATO Doi 621, expressed // 15 D | 3.68 | CNTF up vs PBS |
| Fuca2 | NM_025799 | NM_025799 // Fuca2 // fucosidase, alpha-L- 2, plasma // 10 A2 // 66848 /// ENSMU | 3.61 | CNTF up vs PBS |
| BC013529 | NM_145418 | NM_145418 // BC013529 // cDNA sequence BC013529 // 10 A1 // 215751 /// BC013529 | 2.83 | CNTF up vs PBS |
| Eef1b2 | NM_018796 | NM_018796 // Eef1b2 // eukaryotic translation elongation factor 1 beta 2 // 1 C2 | 5.28 | CNTF up vs PBS |
| V1rj2 | NM_134226 | NM_134226 // V1rj2 // vomeronasal 1 receptor, J2 // 7 A1 // 171260 /// ENSMUST00 | -2.74 | CNTF down vs PBS |
| Psmb4 | NM_008945 | NM_008945 // Psmb4 // proteasome (prosome, macropain) subunit, beta type 4 // 3 | 3.46 | CNTF up vs PBS |
| Prkcdbp | NM_028444 | NM_028444 // Prkcdbp // protein kinase C, delta binding protein // 7 F1 // 10904 | -2.45 | CNTF down vs PBS |
| Mbnl1 | NM_020007 | NM_020007 // Mbnl1 // muscleblind-like 1 (Drosophila) // --- // 56758 /// ENSMUS | 2.35 | CNTF up vs PBS |
| Uqcrq | NM_025352 | NM_025352 // Uqcrq // ubiquinol-cytochrome c reductase, complex III subunit VII | 2.56 | CNTF up vs PBS |
| Ssr4 | NM_009279 | NM_009279 // Ssr4 // signal sequence receptor, delta // X A7.3|X 29.5 cM // 2083 | 3.78 | CNTF up vs PBS |
| Pik3cb | NM_029094 | NM_029094 // Pik3cb // phosphatidylinositol 3-kinase, catalytic, beta polypeptid | 2.34 | CNTF up vs PBS |
| Kpna1 | NM_008465 | NM_008465 // Kpna1 // karyopherin (importin) alpha 1 // --- // 16646 /// ENSMUST | 2.13 | CNTF up vs PBS |
| AI428936 | NM_153577 | NM_153577 // AI428936 // expressed sequence AI428936 // 7 B1 // 233066 /// ENSMU | -2.08 | CNTF down vs PBS |
| Sepp1 | NM_009155 | NM_009155 // Sepp1 // selenoprotein P, plasma, 1 // 15 A1|15 5.9 cM // 20363 /// | 5.94 | CNTF up vs PBS |
| Pfn1 | NM_011072 | NM_011072 // Pfn1 // profilin 1 // 11 B4|11 42.0 cM // 18643 /// ENSMUST00000018 | 5.17 | CNTF up vs PBS |
| Elf5 | NM_010125 | NM_010125 // Elf5 // E74-like factor 5 // 2 E3 // 13711 /// NM_001145813 // Elf5 | -2.08 | CNTF down vs PBS |
| Tram1 | NM_028173 | NM_028173 // Tram1 // translocating chain-associating membrane protein 1 // 1 A3 | 3.60 | CNTF up vs PBS |
| Twf1 | NM_008971 | NM_008971 // Twf1 // twinfilin, actin-binding protein, homolog 1 (Drosophila) // | 2.29 | CNTF up vs PBS |
| Cmtm6 | NM_026036 | NM_026036 // Cmtm6 // CKLF-like MARVEL transmembrane domain containing 6 // 9 F3 | 3.13 | CNTF up vs PBS |
| Rps3 | NM_012052 | NM_012052 // Rps3 // ribosomal protein S3 // 7 F1|7 49.6 cM // 27050 /// ENSMUST | 26.16 | CNTF up vs PBS |
| Slfn4 | NM_011410 | NM_011410 // Slfn4 // schlafen 4 // 11 C // 20558 /// NM_011409 // Slfn3 // schl | 6.68 | CNTF up vs PBS |
| Syk | NM_011518 | NM_011518 // Syk // spleen tyrosine kinase // 13 B-C2|13 37.0 cM // 20963 /// EN | 2.89 | CNTF up vs PBS |
| Ly86 | NM_010745 | NM_010745 // Ly86 // lymphocyte antigen 86 // 13 A3.3 // 17084 /// ENSMUST000000 | 8.00 | CNTF up vs PBS |
| Ccdc57 | NM_027745 | NM_027745 // Ccdc57 // coiled-coil domain containing 57 // 11 E2 // 71276 /// EN | -2.07 | CNTF down vs PBS |
| Acsl4 | NM_207625 | NM_207625 // Acsl4 // acyl-CoA synthetase long-chain family member 4 // X F2 // | 2.50 | CNTF up vs PBS |
| Nmt1 | NM_008707 | NM_008707 // Nmt1 // N-myristoyltransferase 1 // 11 D // 18107 /// ENSMUST000000 | 2.09 | CNTF up vs PBS |
| Wdr1 | NM_011715 | NM_011715 // Wdr1 // WD repeat domain 1 // 5 B3|5 24.0 cM // 22388 /// ENSMUST00 | 2.01 | CNTF up vs PBS |
| Pnp1 | NM_013632 | NM_013632 // Pnp1 // purine-nucleoside phosphorylase 1 // 14 B-C1|14 19.5 cM // | 2.58 | CNTF up vs PBS |
| Cttnbp2nl | NM_030249 | NM_030249 // Cttnbp2nl // CTTNBP2 N-terminal like // 3 F2.2 // 80281 /// ENSMUST | 2.05 | CNTF up vs PBS |
| Ifrd1 | NM_013562 | NM_013562 // Ifrd1 // interferon-related developmental regulator 1 // 12 B1|12 2 | 3.42 | CNTF up vs PBS |
| Fcgr4 | NM_144559 | NM_144559 // Fcgr4 // Fc receptor, IgG, low affinity IV // 1 H3|1 92.29 cM // 24 | 6.61 | CNTF up vs PBS |
| Hisppd1 | NM_173760 | NM_173760 // Hisppd1 // histidine acid phosphatase domain containing 1 // 1 D // | 2.43 | CNTF up vs PBS |
| Ifngr2 | NM_008338 | NM_008338 // Ifngr2 // interferon gamma receptor 2 // 16 C3.3|16 63.3 cM // 1598 | 3.09 | CNTF up vs PBS |
| Atp6v0e | NM_025272 | NM_025272 // Atp6v0e // ATPase, H+ transporting, lysosomal V0 subunit E // 17 A3 | 4.16 | CNTF up vs PBS |
| Tm4sf19 | NM_001160402 | NM_001160402 // Tm4sf19 // transmembrane 4 L six family member 19 // 16 B3 // 27 | 2.30 | CNTF up vs PBS |
| Tmem108 | NM_178638 | NM_178638 // Tmem108 // transmembrane protein 108 // 9 F1 // 81907 /// ENSMUST00 | -2.16 | CNTF down vs PBS |
| Scpep1 | NM_029023 | NM_029023 // Scpep1 // serine carboxypeptidase 1 // 11 C // 74617 /// ENSMUST000 | 4.03 | CNTF up vs PBS |
| Cltc | NM_001003908 | NM_001003908 // Cltc // clathrin, heavy polypeptide (Hc) // 11 C // 67300 /// EN | 2.91 | CNTF up vs PBS |
| Tmem204 | NM_001001183 | NM_001001183 // Tmem204 // transmembrane protein 204 // 17 A3.3 // 407831 /// EN | -2.89 | CNTF down vs PBS |
| Surf4 | NM_011512 | NM_011512 // Surf4 // surfeit gene 4 // 2 A3|2 15.5 cM // 20932 /// ENSMUST00000 | 2.46 | CNTF up vs PBS |
| Ugp2 | NM_139297 | NM_139297 // Ugp2 // UDP-glucose pyrophosphorylase 2 // 11 A3.1|11 12.0 cM // 21 | 2.78 | CNTF up vs PBS |
| Alg5 | NM_025442 | NM_025442 // Alg5 // asparagine-linked glycosylation 5 homolog (yeast, dolichyl- | 2.68 | CNTF up vs PBS |
| Atp6ap1 | NM_018794 | NM_018794 // Atp6ap1 // ATPase, H+ transporting, lysosomal accessory protein 1 / | 3.64 | CNTF up vs PBS |
| Ireb2 | NM_022655 | NM_022655 // Ireb2 // iron responsive element binding protein 2 // 9 B|9 29.0 cM | 2.05 | CNTF up vs PBS |
| Unc93b1 | NM_019449 | NM_019449 // Unc93b1 // unc-93 homolog B1 (C. elegans) // 19 A // 54445 /// NM_0 | 2.72 | CNTF up vs PBS |
| Ypel5 | NM_027166 | NM_027166 // Ypel5 // yippee-like 5 (Drosophila) // 17 E2 // 383295 /// ENSMUST0 | 3.22 | CNTF up vs PBS |
| Rragc | NM_017475 | NM_017475 // Rragc // Ras-related GTP binding C // 4 D // 54170 /// ENSMUST00000 | 2.75 | CNTF up vs PBS |
| Sdcbp | NM_001098227 | NM_001098227 // Sdcbp // syndecan binding protein // 4 A1 // 53378 /// NM_016807 | 7.37 | CNTF up vs PBS |
| Nceh1 | NM_178772 | NM_178772 // Nceh1 // arylacetamide deacetylase-like 1 // 3 A3 // 320024 /// ENS | 3.13 | CNTF up vs PBS |
| Etf1 | NM_144866 | NM_144866 // Etf1 // eukaryotic translation termination factor 1 // 18 B1 // 225 | 3.05 | CNTF up vs PBS |
| Atp6ap2 | NM_027439 | NM_027439 // Atp6ap2 // ATPase, H+ transporting, lysosomal accessory protein 2 / | 2.12 | CNTF up vs PBS |
| Csf2rb2 | NM_007781 | NM_007781 // Csf2rb2 // colony stimulating factor 2 receptor, beta 2, low-affini | 3.76 | CNTF up vs PBS |
| Tcfec | NM_031198 | NM_031198 // Tcfec // transcription factor EC // 6 E4-G // 21426 /// ENSMUST0000 | 5.43 | CNTF up vs PBS |
| Gm2a | NM_010299 | NM_010299 // Gm2a // GM2 ganglioside activator protein // 11 B1.3|11 29.0 cM // | 4.27 | CNTF up vs PBS |
| Hgf | NM_010427 | NM_010427 // Hgf // hepatocyte growth factor // 5 4.0 cM // 15234 /// ENSMUST000 | 2.86 | CNTF up vs PBS |
| Arg1 | NM_007482 | NM_007482 // Arg1 // arginase, liver // 10 A4 // 11846 /// ENSMUST00000020161 // | 9.78 | CNTF up vs PBS |
| Arpc4 | NM_026552 | NM_026552 // Arpc4 // actin related protein 2/3 complex, subunit 4 // 6 E3 // 68 | 2.43 | CNTF up vs PBS |
| Ctsa | NM_008906 | NM_008906 // Ctsa // cathepsin A // 2 H3|2 96.0 cM // 19025 /// NM_001038492 // | 5.02 | CNTF up vs PBS |
| Plaa | NM_172695 | NM_172695 // Plaa // phospholipase A2, activating protein // 4 C5|4 44.5 cM // 1 | 2.82 | CNTF up vs PBS |
| Mettl6 | NM_025907 | NM_025907 // Mettl6 // methyltransferase like 6 // 14 B // 67011 /// ENSMUST0000 | 2.89 | CNTF up vs PBS |
| Pabpc1 | NM_008774 | NM_008774 // Pabpc1 // poly(A) binding protein, cytoplasmic 1 // 15 C // 18458 / | 3.34 | CNTF up vs PBS |
| Rasgef1b | NM_145839 | NM_145839 // Rasgef1b // RasGEF domain family, member 1B // 5 E3 // 320292 /// N | 3.58 | CNTF up vs PBS |
| Adam10 | NM_007399 | NM_007399 // Adam10 // a disintegrin and metallopeptidase domain 10 // 9 D|9 41. | 2.60 | CNTF up vs PBS |
| Mecom | NM_021442 | NM_021442 // Mecom // MDS1 and EVI1 complex locus // 3 A3|3 14.4 cM // 14013 | -2.03 | CNTF down vs PBS |
| Gyg | NM_013755 | NM_013755 // Gyg // glycogenin // 3 A2|3 12.5 cM // 27357 /// ENSMUST00000118015 | 2.11 | CNTF up vs PBS |
| Ahsa1 | NM_146036 | NM_146036 // Ahsa1 // AHA1, activator of heat shock protein ATPase homolog 1 (ye | 2.01 | CNTF up vs PBS |
| Gba | NM_008094 | NM_008094 // Gba // glucosidase, beta, acid // 3 F1|3 44.05 cM // 14466 /// NM_0 | 3.72 | CNTF up vs PBS |
| Josd1 | NM_028792 | NM_028792 // Josd1 // Josephin domain containing 1 // 15 E1 // 74158 /// BC08676 | 2.38 | CNTF up vs PBS |
| Ang | NM_007447 | NM_007447 // Ang // angiogenin, ribonuclease, RNase A family, 5 // 14 B-C1|14 18 | 5.57 | CNTF up vs PBS |
| C1qa | NM_007572 | NM_007572 // C1qa // complement component 1, q subcomponent, alpha polypeptide / | 7.63 | CNTF up vs PBS |
| Runx1 | NM_001111023 | NM_001111023 // Runx1 // runt related transcription factor 1 // 16 C4|16 62.2 cM | 2.25 | CNTF up vs PBS |
| Dclre1c | NM_146114 | NM_146114 // Dclre1c // DNA cross-link repair 1C, PSO2 homolog (S. cerevisiae) / | 2.35 | CNTF up vs PBS |
| Pdcd6ip | NM_011052 | NM_011052 // Pdcd6ip // programmed cell death 6 interacting protein // 9 F2 // 1 | 2.52 | CNTF up vs PBS |
| Bud31 | NM_001008705 | NM_001008705 // Bud31 // BUD31 homolog (yeast) // 5 G2 // 231889 /// NM_133735 / | 2.15 | CNTF up vs PBS |
| Gpnmb | NM_053110 | NM_053110 // Gpnmb // glycoprotein (transmembrane) nmb // 6 B2.3|6 21.0 cM // 93 | 14.04 | CNTF up vs PBS |
| Blvra | NM_026678 | NM_026678 // Blvra // biliverdin reductase A // 2 F3|2 62.0 cM // 109778 /// ENS | 2.50 | CNTF up vs PBS |
| Eef2 | NM_007907 | NM_007907 // Eef2 // eukaryotic translation elongation factor 2 // 10 C1 // 1362 | 3.29 | CNTF up vs PBS |
| Fundc2 | NM_026126 | NM_026126 // Fundc2 // FUN14 domain containing 2 // --- // 67391 /// ENSMUST0000 | 3.80 | CNTF up vs PBS |
| Ncoa3 | NM_008679 | NM_008679 // Ncoa3 // nuclear receptor coactivator 3 // 2 H2-H4 // 17979 /// ENS | 2.04 | CNTF up vs PBS |
| Actb | NM_007393 | NM_007393 // Actb // actin, beta // 5 G2|5 80.0 cM // 11461 /// ENSMUST000000315 | 6.36 | CNTF up vs PBS |
| Wsb1 | NM_019653 | NM_019653 // Wsb1 // WD repeat and SOCS box-containing 1 // 11 B5 // 78889 /// N | 2.61 | CNTF up vs PBS |
| Tlr13 | NM_205820 | NM_205820 // Tlr13 // toll-like receptor 13 // X D // 279572 /// ENSMUST00000040 | 14.23 | CNTF up vs PBS |
| Taf12 | NM_025579 | NM_025579 // Taf12 // TAF12 RNA polymerase II, TATA box binding protein (TBP)-as | 2.50 | CNTF up vs PBS |
| Wdr5b | NM_027113 | NM_027113 // Wdr5b // WD repeat domain 5B // 16 B3 // 69544 /// ENSMUST000000422 | -2.24 | CNTF down vs PBS |
| Ms4a6b | NM_027209 | NM_027209 // Ms4a6b // membrane-spanning 4-domains, subfamily A, member 6B // 19 | 13.50 | CNTF up vs PBS |
| Lyn | NM_001111096 | NM_001111096 // Lyn // Yamaguchi sarcoma viral (v-yes-1) oncogene homolog // 4 A | 7.53 | CNTF up vs PBS |
| Gng13 | NM_022422 | NM_022422 // Gng13 // guanine nucleotide binding protein (G protein), gamma 13 / | -3.62 | CNTF down vs PBS |
| Parl | NM_001005767 | NM_001005767 // Parl // presenilin associated, rhomboid-like // 16 A3|16 14.0 cM | 3.28 | CNTF up vs PBS |
| Lrfn2 | NM_027452 | NM_027452 // Lrfn2 // leucine rich repeat and fibronectin type III domain contai | -2.22 | CNTF down vs PBS |
| Ak2 | NM_016895 | NM_016895 // Ak2 // adenylate kinase 2 // 4 D2.2|4 61.0 cM // 11637 /// NM_00103 | 2.20 | CNTF up vs PBS |
| Cd24a | NM_009846 | NM_009846 // Cd24a // CD24a antigen // 10 B2|10 26.0 cM // 12484 /// BC075622 // | 2.92 | CNTF up vs PBS |
| Ppib | NM_011149 | NM_011149 // Ppib // peptidylprolyl isomerase B // 9 C // 19035 /// NM_001025612 | 10.04 | CNTF up vs PBS |
| Ddx52 | NM_030096 | NM_030096 // Ddx52 // DEAD (Asp-Glu-Ala-Asp) box polypeptide 52 // 11 B5 // 7839 | 2.21 | CNTF up vs PBS |
| Eaf2 | NM_134111 | NM_134111 // Eaf2 // ELL associated factor 2 // 16 B3 // 106389 /// NM_001113401 | -2.20 | CNTF down vs PBS |
| Gns | NM_029364 | NM_029364 // Gns // glucosamine (N-acetyl)-6-sulfatase // 10 D2 // 75612 /// ENS | 2.52 | CNTF up vs PBS |
| Clptm1l | NM_146047 | NM_146047 // Clptm1l // CLPTM1-like // 13 C1 // 218335 /// ENSMUST00000022102 // | 2.13 | CNTF up vs PBS |
| Cops6 | NM_012002 | NM_012002 // Cops6 // COP9 (constitutive photomorphogenic) homolog, subunit 6 (A | 2.80 | CNTF up vs PBS |
| Podn | NM_172874 | NM_172874 // Podn // podocan // 4 C7 // 242608 /// ENSMUST00000106709 // Podn // | -2.03 | CNTF down vs PBS |
| Stt3a | NM_008408 | NM_008408 // Stt3a // STT3, subunit of the oligosaccharyltransferase complex, ho | 4.24 | CNTF up vs PBS |
| 1110008F13Rik | NM_026124 | NM_026124 // 1110008F13Rik // RIKEN cDNA 1110008F13 gene // 2 H2 // 67388 /// NM | 2.88 | CNTF up vs PBS |
| Lamp2 | NM_010685 | NM_010685 // Lamp2 // lysosomal-associated membrane protein 2 // X A3.3|X 13.0 c | 3.40 | CNTF up vs PBS |
| Apcdd1 | NM_133237 | NM_133237 // Apcdd1 // adenomatosis polyposis coli down-regulated 1 // 18 E1 // | -4.23 | CNTF down vs PBS |
| Ppp1r11 | NM_029632 | NM_029632 // Ppp1r11 // protein phosphatase 1, regulatory (inhibitor) subunit 11 | 4.55 | CNTF up vs PBS |
| Dusp6 | NM_026268 | NM_026268 // Dusp6 // dual specificity phosphatase 6 // 10 C3 // 67603 /// ENSMU | 2.44 | CNTF up vs PBS |
| Ifngr1 | NM_010511 | NM_010511 // Ifngr1 // interferon gamma receptor 1 // 10 A3|10 15.0 cM // 15979 | 2.51 | CNTF up vs PBS |
| Mrpl13 | NM_026759 | NM_026759 // Mrpl13 // mitochondrial ribosomal protein L13 // 15 D2 // 68537 /// | 2.29 | CNTF up vs PBS |
| Hnrnpab | NM_001048061 | NM_001048061 // Hnrnpab // heterogeneous nuclear ribonucleoprotein A/B // 11 B1. | 2.38 | CNTF up vs PBS |
| Smpdl3a | NM_020561 | NM_020561 // Smpdl3a // sphingomyelin phosphodiesterase, acid-like 3A // 10 B4 / | 4.15 | CNTF up vs PBS |
| Acsl5 | NM_027976 | NM_027976 // Acsl5 // acyl-CoA synthetase long-chain family member 5 // 19 D2 // | 3.24 | CNTF up vs PBS |
| Atp6v1h | NM_133826 | NM_133826 // Atp6v1h // ATPase, H+ transporting, lysosomal V1 subunit H // 1 A1 | 2.47 | CNTF up vs PBS |
| Ei24 | NM_007915 | NM_007915 // Ei24 // etoposide induced 2.4 mRNA // 9 A4|9 14.0 cM // 13663 /// N | 3.66 | CNTF up vs PBS |
| Rapgef6 | NM_175258 | NM_175258 // Rapgef6 // Rap guanine nucleotide exchange factor (GEF) 6 // 11 B1. | 2.30 | CNTF up vs PBS |
| Ctsz | NM_022325 | NM_022325 // Ctsz // cathepsin Z // 2 H4|2 103.5 cM // 64138 /// ENSMUST00000016 | 5.55 | CNTF up vs PBS |
| Tpp2 | NM_009418 | NM_009418 // Tpp2 // tripeptidyl peptidase II // 1 C1.1|1 27.0 cM // 22019 /// E | 2.23 | CNTF up vs PBS |
| Sirpa | NM_007547 | NM_007547 // Sirpa // signal-regulatory protein alpha // 2 F3|2 73.1 cM // 19261 | 2.36 | CNTF up vs PBS |
| Fkbp1a | NM_008019 | NM_008019 // Fkbp1a // FK506 binding protein 1a // 2 G3 // 14225 /// ENSMUST0000 | 2.60 | CNTF up vs PBS |
| 2010106G01Rik | NM_023220 | NM_023220 // 2010106G01Rik // RIKEN cDNA 2010106G01 gene // 2 F3 // 66552 /// EN | 2.82 | CNTF up vs PBS |
| Cd300lf | NM_145634 | NM_145634 // Cd300lf // CD300 antigen like family member F // 11 E2 // 246746 // | 3.61 | CNTF up vs PBS |
| 2810055F11Rik | NM_026038 | NM_026038 // 2810055F11Rik // RIKEN cDNA 2810055F11 gene // 12 C3 // 67217 /// E | -2.45 | CNTF down vs PBS |
| Ddo | NM_027442 | NM_027442 // Ddo // D-aspartate oxidase // 10 B1 // 70503 /// ENSMUST00000019977 | -2.24 | CNTF down vs PBS |
| Ctsh | NM_007801 | NM_007801 // Ctsh // cathepsin H // 9 E3.1|9 50.0 cM // 13036 /// ENSMUST0000003 | 3.07 | CNTF up vs PBS |
| Il1f8 | NM_027163 | NM_027163 // Il1f8 // interleukin 1 family, member 8 // 2 A3|2 10.3 cM // 69677 | -2.33 | CNTF down vs PBS |
| Tmem77 | NM_026013 | NM_026013 // Tmem77 // transmembrane protein 77 // 3 F3 // 67171 /// NM_00102558 | 3.10 | CNTF up vs PBS |
| Eif5 | NM_173363 | NM_173363 // Eif5 // eukaryotic translation initiation factor 5 // 12 F1|12 57.0 | 2.46 | CNTF up vs PBS |
| Kazald1 | NM_178929 | NM_178929 // Kazald1 // Kazal-type serine peptidase inhibitor domain 1 // 19 C3 | -2.28 | CNTF down vs PBS |
| Clec7a | NM_020008 | NM_020008 // Clec7a // C-type lectin domain family 7, member a // --- // 56644 / | 10.29 | CNTF up vs PBS |
| Irak3 | NM_028679 | NM_028679 // Irak3 // interleukin-1 receptor-associated kinase 3 // 10 D2 // 739 | 2.41 | CNTF up vs PBS |
| Mpp1 | NM_008621 | NM_008621 // Mpp1 // membrane protein, palmitoylated // X A7.3|X 30.48 cM // 175 | 3.49 | CNTF up vs PBS |
| Chrm5 | NM_205783 | NM_205783 // Chrm5 // cholinergic receptor, muscarinic 5 // 2 E3 // 213788 /// E | -2.55 | CNTF down vs PBS |
| Rheb | NM_053075 | NM_053075 // Rheb // Ras homolog enriched in brain // 5 A3 // 19744 /// ENSMUST0 | 3.32 | CNTF up vs PBS |
| AI747699 | BC052506 | BC052506 // AI747699 // expressed sequence AI747699 // 19 C1 // 381236 /// NM_00 | 4.14 | CNTF up vs PBS |
| 5430435G22Rik | NM_145509 | NM_145509 // 5430435G22Rik // RIKEN cDNA 5430435G22 gene // 1 E4 // 226421 /// E | 3.37 | CNTF up vs PBS |
| Il4ra | NM_001008700 | NM_001008700 // Il4ra // interleukin 4 receptor, alpha // 7 F3|7 62.0 cM // 1619 | 2.63 | CNTF up vs PBS |
| Atp6v1e1 | NM_007510 | NM_007510 // Atp6v1e1 // ATPase, H+ transporting, lysosomal V1 subunit E1 // 6 F | 5.42 | CNTF up vs PBS |
| Cpd | NM_007754 | NM_007754 // Cpd // carboxypeptidase D // 11 B5|11 46.0 cM // 12874 /// ENSMUST0 | 3.21 | CNTF up vs PBS |
| Bcap29 | NM_007530 | NM_007530 // Bcap29 // B-cell receptor-associated protein 29 // 12 A3|12 17.0 cM | 2.12 | CNTF up vs PBS |
| Gm1019 | NM_001001650 | NM_001001650 // Gm1019 // predicted gene 1019 // 3 F1 // 368202 /// ENSMUST00000 | -2.93 | CNTF down vs PBS |
| Slc36a4 | NM_172289 | NM_172289 // Slc36a4 // solute carrier family 36 (proton/amino acid symporter), | 3.14 | CNTF up vs PBS |
| Anxa5 | NM_009673 | NM_009673 // Anxa5 // annexin A5 // 3 B|3 19.2 cM // 11747 /// ENSMUST0000002926 | 4.44 | CNTF up vs PBS |
| Nckap1l | NM_153505 | NM_153505 // Nckap1l // NCK associated protein 1 like // 15 F3 // 105855 /// ENS | 6.93 | CNTF up vs PBS |
| Rilpl2 | NM_030259 | NM_030259 // Rilpl2 // Rab interacting lysosomal protein-like 2 // 5 F // 80291 | 3.27 | CNTF up vs PBS |
| Vpreb3 | NM_009514 | NM_009514 // Vpreb3 // pre-B lymphocyte gene 3 // 10 C // 22364 /// ENSMUST00000 | -2.01 | CNTF down vs PBS |
| Cyfip1 | NM_011370 | NM_011370 // Cyfip1 // cytoplasmic FMR1 interacting protein 1 // 7 B5 // 20430 / | 2.60 | CNTF up vs PBS |
| H2-DMa | NM_010386 | NM_010386 // H2-DMa // histocompatibility 2, class II, locus DMa // 17 B1|17 18. | 2.47 | CNTF up vs PBS |
| 6330578E17Rik | NM_198006 | NM_198006 // 6330578E17Rik // RIKEN cDNA 6330578E17 gene // 1 B // 76178 /// ENS | 3.50 | CNTF up vs PBS |
| Tgfbi | NM_009369 | NM_009369 // Tgfbi // transforming growth factor, beta induced // 13 38.0 cM // | 7.62 | CNTF up vs PBS |
| D19Ertd386e | NM_177464 | NM_177464 // D19Ertd386e // DNA segment, Chr 19, ERATO Doi 386, expressed // 19 | 2.37 | CNTF up vs PBS |
| Mef2a | NM_001033713 | NM_001033713 // Mef2a // myocyte enhancer factor 2A // 7 C|7 33.0 cM // 17258 // | 3.11 | CNTF up vs PBS |
| Pomp | NM_025624 | NM_025624 // Pomp // proteasome maturation protein // 5 G3 // 66537 /// ENSMUST0 | 3.54 | CNTF up vs PBS |
| Prdx5 | NM_012021 | NM_012021 // Prdx5 // peroxiredoxin 5 // 19 A|19 0.5 cM // 54683 /// ENSMUST0000 | 4.39 | CNTF up vs PBS |
| A630005I04Rik | BC150970 | BC150970 // A630005I04Rik // RIKEN cDNA A630005I04 gene // 7 F1 // 320743 /// NM | -2.18 | CNTF down vs PBS |
| Skap2 | NM_018773 | NM_018773 // Skap2 // src family associated phosphoprotein 2 // 6 B3 // 54353 // | 4.30 | CNTF up vs PBS |
| Asah1 | NM_019734 | NM_019734 // Asah1 // N-acylsphingosine amidohydrolase 1 // 8 A4 // 11886 /// EN | 3.00 | CNTF up vs PBS |
| Ctsb | NM_007798 | NM_007798 // Ctsb // cathepsin B // 14 D1|14 28.0 cM // 13030 /// ENSMUST0000000 | 6.06 | CNTF up vs PBS |
| Derl2 | NM_033562 | NM_033562 // Derl2 // Der1-like domain family, member 2 // 11 B4|11 42.0 cM // 1 | 3.86 | CNTF up vs PBS |
| Acp2 | NM_007387 | NM_007387 // Acp2 // acid phosphatase 2, lysosomal // 2 E1|2 52.0 cM // 11432 // | 2.77 | CNTF up vs PBS |
| Prcp | NM_028243 | NM_028243 // Prcp // prolylcarboxypeptidase (angiotensinase C) // 7 E2 // 72461 | 5.77 | CNTF up vs PBS |
| Reep3 | NM_178606 | NM_178606 // Reep3 // receptor accessory protein 3 // 10 B5.1|10 30.0 cM // 2819 | 2.51 | CNTF up vs PBS |
| Xbp1 | NM_013842 | NM_013842 // Xbp1 // X-box binding protein 1 // 11 A1|11 3.0 cM // 22433 /// ENS | 2.26 | CNTF up vs PBS |
| Anxa2 | NM_007585 | NM_007585 // Anxa2 // annexin A2 // 9 C|9 37.0 cM // 12306 /// ENSMUST0000003475 | 4.07 | CNTF up vs PBS |
| 0610031J06Rik | NM_020003 | NM_020003 // 0610031J06Rik // RIKEN cDNA 0610031J06 gene // 3 F1 // 56700 /// EN | 2.00 | CNTF up vs PBS |
| Emcn | NM_016885 | NM_016885 // Emcn // endomucin // 3 G3 // 59308 /// ENSMUST00000119475 // Emcn / | -2.03 | CNTF down vs PBS |
| Arrdc4 | NM_001042592 | NM_001042592 // Arrdc4 // arrestin domain containing 4 // 7 D1 // 66412 /// NM_0 | 3.05 | CNTF up vs PBS |
| Rbm47 | NM_178446 | NM_178446 // Rbm47 // RNA binding motif protein 47 // 5 C3.1 // 245945 /// NM_00 | 2.67 | CNTF up vs PBS |
| 1110020C03Rik | BC062805 | BC062805 // 1110020C03Rik // RIKEN cDNA 1110020C03 gene // 4 D2.1 // 68625 /// E | -2.03 | CNTF down vs PBS |
| Atp6v0d1 | NM_013477 | NM_013477 // Atp6v0d1 // ATPase, H+ transporting, lysosomal V0 subunit D1 // 8 D | 4.90 | CNTF up vs PBS |
| Jak2 | NM_008413 | NM_008413 // Jak2 // Janus kinase 2 // 19 C1|19 24.0 cM // 16452 /// NM_00104817 | 2.27 | CNTF up vs PBS |
| Eef1e1 | NM_025380 | NM_025380 // Eef1e1 // eukaryotic translation elongation factor 1 epsilon 1 // 1 | 3.42 | CNTF up vs PBS |
| Mcm3 | NM_008563 | NM_008563 // Mcm3 // minichromosome maintenance deficient 3 (S. cerevisiae) // 1 | 2.08 | CNTF up vs PBS |
| Bche | NM_009738 | NM_009738 // Bche // butyrylcholinesterase // 3 E3 // 12038 /// ENSMUST000000293 | -2.56 | CNTF down vs PBS |
| Olfr609 | ENSMUST00000055787 | ENSMUST00000055787 // Olfr609 // olfactory receptor 609 // --- // 259086 | -2.57 | CNTF down vs PBS |
| Vps25 | NM_026776 | NM_026776 // Vps25 // vacuolar protein sorting 25 (yeast) // 11 D|11 60.0 cM // | 4.06 | CNTF up vs PBS |
| Pitpna | NM_008850 | NM_008850 // Pitpna // phosphatidylinositol transfer protein, alpha // 11 B5|11 | 2.28 | CNTF up vs PBS |
| Plek | NM_019549 | NM_019549 // Plek // pleckstrin // 11 A2|11 6.5 cM // 56193 /// NM_029861 // Cnr | 9.44 | CNTF up vs PBS |
| Mmp12 | NM_008605 | NM_008605 // Mmp12 // matrix metallopeptidase 12 // 9 A1|9 1.0 cM // 17381 /// E | 4.49 | CNTF up vs PBS |
| Rpn1 | NM_133933 | NM_133933 // Rpn1 // ribophorin I // 6 D1|6 38.0 cM // 103963 /// ENSMUST0000003 | 2.52 | CNTF up vs PBS |
| Ifi30 | NM_023065 | NM_023065 // Ifi30 // interferon gamma inducible protein 30 // 8 B3.3 // 65972 / | 4.50 | CNTF up vs PBS |
| Etv6 | NM_007961 | NM_007961 // Etv6 // ets variant gene 6 (TEL oncogene) // 6 G2|6 63.9 cM // 1401 | 2.19 | CNTF up vs PBS |
| Ndufs4 | NM_010887 | NM_010887 // Ndufs4 // NADH dehydrogenase (ubiquinone) Fe-S protein 4 // 13 D2.2 | 2.12 | CNTF up vs PBS |
| Atp6v1d | NM_023721 | NM_023721 // Atp6v1d // ATPase, H+ transporting, lysosomal V1 subunit D // 12 C3 | 2.89 | CNTF up vs PBS |
| Abr | NM_198018 | NM_198018 // Abr // active BCR-related gene // 11 B5|11 45.0 cM // 109934 /// NM | 2.08 | CNTF up vs PBS |
| Sla | NM_001029841 | NM_001029841 // Sla // src-like adaptor // 15 D2|15 37.5 cM // 20491 /// NM_0091 | 7.39 | CNTF up vs PBS |
| Prkcd | NM_011103 | NM_011103 // Prkcd // protein kinase C, delta // 14 B|14 11.0 cM // 18753 /// EN | 3.42 | CNTF up vs PBS |
| Laptm5 | NM_010686 | NM_010686 // Laptm5 // lysosomal-associated protein transmembrane 5 // 4 D2.3 // | 10.22 | CNTF up vs PBS |
| Fth1 | NM_010239 | NM_010239 // Fth1 // ferritin heavy chain 1 // 19 A-C|19 2.0 cM // 14319 /// ENS | 4.57 | CNTF up vs PBS |
| Tnfaip8 | NM_134131 | NM_134131 // Tnfaip8 // tumor necrosis factor, alpha-induced protein 8 // 18 D1 | 2.47 | CNTF up vs PBS |
| Snx18 | NM_130796 | NM_130796 // Snx18 // sorting nexin 18 // 13 D2.2 // 170625 /// ENSMUST000001092 | 3.41 | CNTF up vs PBS |
| Slfn1 | NM_011407 | NM_011407 // Slfn1 // schlafen 1 // 11 C|11 48.0 cM // 20555 /// ENSMUST00000037 | 3.82 | CNTF up vs PBS |
| Tsn | NM_011650 | NM_011650 // Tsn // translin // 1 E2.3 // 22099 /// ENSMUST00000027623 // Tsn // | 2.21 | CNTF up vs PBS |
| Olfr1043 | NM_146577 | NM_146577 // Olfr1043 // olfactory receptor 1043 // --- // 258570 /// ENSMUST000 | -2.15 | CNTF down vs PBS |
| Aloxe3 | NM_011786 | NM_011786 // Aloxe3 // arachidonate lipoxygenase 3 // 11 B3|11 37.0 cM // 23801 | -2.04 | CNTF down vs PBS |
| Atg5 | NM_053069 | NM_053069 // Atg5 // autophagy-related 5 (yeast) // 10 B2|10 26.0 cM // 11793 // | 2.36 | CNTF up vs PBS |
| Mtpn | NM_008098 | NM_008098 // Mtpn // myotrophin // 6 B1 // 14489 /// ENSMUST00000031866 // Mtpn | 2.21 | CNTF up vs PBS |
| 1500003O03Rik | NM_019769 | NM_019769 // 1500003O03Rik // RIKEN cDNA 1500003O03 gene // 2 E5 // 56398 /// BC | 2.73 | CNTF up vs PBS |
| Ptpn6 | NM_013545 | NM_013545 // Ptpn6 // protein tyrosine phosphatase, non-receptor type 6 // 6 F2| | 3.34 | CNTF up vs PBS |
| Gnai3 | NM_010306 | NM_010306 // Gnai3 // guanine nucleotide binding protein (G protein), alpha inhi | 2.43 | CNTF up vs PBS |
| Eif4g2 | NM_013507 | NM_013507 // Eif4g2 // eukaryotic translation initiation factor 4, gamma 2 // 7 | 2.11 | CNTF up vs PBS |
| Actr2 | NM_146243 | NM_146243 // Actr2 // ARP2 actin-related protein 2 homolog (yeast) // 11 A3.1|11 | 4.36 | CNTF up vs PBS |
| Pld4 | NM_178911 | NM_178911 // Pld4 // phospholipase D family, member 4 // 12 F1 // 104759 /// ENS | 5.05 | CNTF up vs PBS |
| Klk1b5 | NM_008456 | NM_008456 // Klk1b5 // kallikrein 1-related peptidase b5 // 7 B4|7 23.01 cM // 1 | -2.32 | CNTF down vs PBS |
| Erg | NM_133659 | NM_133659 // Erg // avian erythroblastosis virus E-26 (v-ets) oncogene related / | -2.41 | CNTF down vs PBS |
| Shcbp1 | NM_011369 | NM_011369 // Shcbp1 // Shc SH2-domain binding protein 1 // 8 A1.2 // 20419 /// E | 2.02 | CNTF up vs PBS |
| Ly6e | NM_008529 | NM_008529 // Ly6e // lymphocyte antigen 6 complex, locus E // 15 D3|15 41.7 cM / | 7.04 | CNTF up vs PBS |
| Rab31 | NM_133685 | NM_133685 // Rab31 // RAB31, member RAS oncogene family // 17 E1.1 // 106572 /// | 4.53 | CNTF up vs PBS |
| Arhgdia | NM_133796 | NM_133796 // Arhgdia // Rho GDP dissociation inhibitor (GDI) alpha // 11 E2 // 1 | 4.14 | CNTF up vs PBS |
| Anxa3 | NM_013470 | NM_013470 // Anxa3 // annexin A3 // 5 E3|5 54.0 cM // 11745 /// ENSMUST000000314 | 7.88 | CNTF up vs PBS |
| Casp8 | NM_009812 | NM_009812 // Casp8 // caspase 8 // 1 B|1 30.1 cM // 12370 /// NM_001080126 // Ca | 6.23 | CNTF up vs PBS |
| Gtf3c6 | NM_026113 | NM_026113 // Gtf3c6 // general transcription factor IIIC, polypeptide 6, alpha / | 2.21 | CNTF up vs PBS |
| Zswim6 | NM_145456 | NM_145456 // Zswim6 // zinc finger, SWIM domain containing 6 // 13 D2.1 // 67263 | 2.41 | CNTF up vs PBS |
| Sgpl1 | NM_009163 | NM_009163 // Sgpl1 // sphingosine phosphate lyase 1 // 10 B4|10 32.0 cM // 20397 | 3.27 | CNTF up vs PBS |
| BC049349 | BC049349 | BC049349 // BC049349 // cDNA sequence BC049349 // 8 B3.3 // 234413 /// BC065788 | -2.02 | CNTF down vs PBS |
| Ldhal6b | NM_175349 | NM_175349 // Ldhal6b // lactate dehydrogenase A-like 6B // 17 A1 // 106557 /// A | -2.22 | CNTF down vs PBS |
| Dynlrb1 | NM_025947 | NM_025947 // Dynlrb1 // dynein light chain roadblock-type 1 // 2 H1 // 67068 /// | 2.32 | CNTF up vs PBS |
| Serpinb9d | NM_011460 | NM_011460 // Serpinb9d // serine (or cysteine) peptidase inhibitor, clade B, mem | -2.29 | CNTF down vs PBS |
| Ptprj | NM_008982 | NM_008982 // Ptprj // protein tyrosine phosphatase, receptor type, J // 2 E1-2|2 | 2.44 | CNTF up vs PBS |
| Egfl6 | NM_019397 | NM_019397 // Egfl6 // EGF-like-domain, multiple 6 // X F5|X 71.5 cM // 54156 /// | -2.12 | CNTF down vs PBS |
| Plau | NM_008873 | NM_008873 // Plau // plasminogen activator, urokinase // 14 A3|14 2.5 cM // 1879 | 4.12 | CNTF up vs PBS |
| Ly9 | NM_008534 | NM_008534 // Ly9 // lymphocyte antigen 9 // 1 H3|1 93.3 cM // 17085 /// ENSMUST0 | 3.78 | CNTF up vs PBS |
| Zbtb1 | NM_178744 | NM_178744 // Zbtb1 // zinc finger and BTB domain containing 1 // 12 C3 // 268564 | 3.82 | CNTF up vs PBS |
| Abhd12 | NM_024465 | NM_024465 // Abhd12 // abhydrolase domain containing 12 // 2 G3 // 76192 /// ENS | 4.81 | CNTF up vs PBS |
| 5830427D03Rik | BC147361 | BC147361 // 5830427D03Rik // RIKEN cDNA 5830427D03 gene // 15 F1 // 76061 /// NM | -2.14 | CNTF down vs PBS |
| Stx4a | NM_009294 | NM_009294 // Stx4a // syntaxin 4A (placental) // 7 F4 // 20909 /// ENSMUST000001 | 4.13 | CNTF up vs PBS |
| Psma6 | NM_011968 | NM_011968 // Psma6 // proteasome (prosome, macropain) subunit, alpha type 6 // 1 | 3.30 | CNTF up vs PBS |
| Stx7 | NM_016797 | NM_016797 // Stx7 // syntaxin 7 // 10 A3 // 53331 /// ENSMUST00000020174 // Stx7 | 2.71 | CNTF up vs PBS |
| 1500032L24Rik | BC092006 | BC092006 // 1500032L24Rik // RIKEN cDNA 1500032L24 gene // 15 E2 // 69029 /// EN | 2.87 | CNTF up vs PBS |
| Rab8a | NM_023126 | NM_023126 // Rab8a // RAB8A, member RAS oncogene family // 8 B3.3|8 33.0 cM // 1 | 2.29 | CNTF up vs PBS |
| B4galt5 | NM_019835 | NM_019835 // B4galt5 // UDP-Gal:betaGlcNAc beta 1,4-galactosyltransferase, polyp | 2.01 | CNTF up vs PBS |
| Ints12 | NM_027927 | NM_027927 // Ints12 // integrator complex subunit 12 // 3 H2 // 71793 /// ENSMUS | 2.26 | CNTF up vs PBS |
| Cpeb2 | NM_175937 | NM_175937 // Cpeb2 // cytoplasmic polyadenylation element binding protein 2 // 5 | 3.05 | CNTF up vs PBS |
| Gusb | NM_010368 | NM_010368 // Gusb // glucuronidase, beta // 5 G1.3 // 110006 /// ENSMUST00000026 | 4.26 | CNTF up vs PBS |
| Pign | NM_013784 | NM_013784 // Pign // phosphatidylinositol glycan anchor biosynthesis, class N // | 2.23 | CNTF up vs PBS |
| P4hb | NM_011032 | NM_011032 // P4hb // prolyl 4-hydroxylase, beta polypeptide // 11 D-E|11 80.0 cM | 2.23 | CNTF up vs PBS |
| Pla2g7 | NM_013737 | NM_013737 // Pla2g7 // phospholipase A2, group VII (platelet-activating factor a | 2.87 | CNTF up vs PBS |
| Nampt | NM_021524 | NM_021524 // Nampt // nicotinamide phosphoribosyltransferase // 12 B1 // 59027 / | 4.07 | CNTF up vs PBS |
| D4Bwg0951e | BC021501 | BC021501 // D4Bwg0951e // DNA segment, Chr 4, Brigham & Women's Genetics 0951 ex | -2.01 | CNTF down vs PBS |
| Ifnar2 | NM_010509 | NM_010509 // Ifnar2 // interferon (alpha and beta) receptor 2 // 16 C3.3|16 63.1 | 3.17 | CNTF up vs PBS |
| Caprin1 | NM_016739 | NM_016739 // Caprin1 // cell cycle associated protein 1 // 2 E2 // 53872 /// NM_ | 2.32 | CNTF up vs PBS |
| Bzrap1 | NM_172449 | NM_172449 // Bzrap1 // benzodiazapine receptor associated protein 1 // 11 C // 2 | -2.00 | CNTF down vs PBS |
| Diap2 | NM_172493 | NM_172493 // Diap2 // diaphanous homolog 2 (Drosophila) // X E3 // 54004 /// NM_ | 2.48 | CNTF up vs PBS |
| Gdi2 | NM_008112 | NM_008112 // Gdi2 // guanosine diphosphate (GDP) dissociation inhibitor 2 // 13 | 2.11 | CNTF up vs PBS |
| Eif2s2 | NM_026030 | NM_026030 // Eif2s2 // eukaryotic translation initiation factor 2, subunit 2 (be | 2.09 | CNTF up vs PBS |
| Top2a | NM_011623 | NM_011623 // Top2a // topoisomerase (DNA) II alpha // 11 D|11 57.0 cM // 21973 / | 4.58 | CNTF up vs PBS |
| Dnajc5b | NM_025489 | NM_025489 // Dnajc5b // DnaJ (Hsp40) homolog, subfamily C, member 5 beta // 3 A3 | -2.04 | CNTF down vs PBS |
| Tmem87b | NM_028248 | NM_028248 // Tmem87b // transmembrane protein 87B // 2 F3 // 72477 /// ENSMUST00 | 2.30 | CNTF up vs PBS |
| Ets2 | NM_011809 | NM_011809 // Ets2 // E26 avian leukemia oncogene 2, 3' domain // 16 C3-qter|16 6 | 2.74 | CNTF up vs PBS |
| Adss | NM_007422 | NM_007422 // Adss // adenylosuccinate synthetase, non muscle // 1 H4 // 11566 // | 3.35 | CNTF up vs PBS |
| Bfar | NM_025976 | NM_025976 // Bfar // bifunctional apoptosis regulator // 16 A1 // 67118 /// ENSM | 2.02 | CNTF up vs PBS |
| Man1a | NM_008548 | NM_008548 // Man1a // mannosidase 1, alpha // 10 B3 // 17155 /// ENSMUST00000003 | 2.89 | CNTF up vs PBS |
| Taf9 | NM_027592 | NM_027592 // Taf9 // TAF9 RNA polymerase II, TATA box binding protein (TBP)-asso | 3.00 | CNTF up vs PBS |
| Cyba | NM_007806 | NM_007806 // Cyba // cytochrome b-245, alpha polypeptide // --- // 13057 /// ENS | 2.30 | CNTF up vs PBS |
| Nrp1 | NM_008737 | NM_008737 // Nrp1 // neuropilin 1 // 8 E|8 73.0 cM // 18186 /// ENSMUST000000269 | 2.81 | CNTF up vs PBS |
| Klf6 | NM_011803 | NM_011803 // Klf6 // Kruppel-like factor 6 // 13 A1 // 23849 /// ENSMUST00000000 | 2.27 | CNTF up vs PBS |
| Xrn2 | NM_011917 | NM_011917 // Xrn2 // 5'-3' exoribonuclease 2 // 2 H1 // 24128 /// ENSMUST0000002 | 2.48 | CNTF up vs PBS |
| Psmc2 | NM_011188 | NM_011188 // Psmc2 // proteasome (prosome, macropain) 26S subunit, ATPase 2 // 5 | 2.15 | CNTF up vs PBS |
| Syf2 | NM_026780 | NM_026780 // Syf2 // SYF2 homolog, RNA splicing factor (S. cerevisiae) // 4 D3|4 | 2.32 | CNTF up vs PBS |
| Hspa5 | NM_022310 | NM_022310 // Hspa5 // heat shock protein 5 // 2 B|2 22.5 cM // 14828 /// ENSMUST | 2.25 | CNTF up vs PBS |
| Rel | NM_009044 | NM_009044 // Rel // reticuloendotheliosis oncogene // 11 A3.2|11 13.0 cM // 1969 | 5.68 | CNTF up vs PBS |
| Ptp4a2 | NM_008974 | NM_008974 // Ptp4a2 // protein tyrosine phosphatase 4a2 // 4 D2.3 // 19244 /// E | 2.09 | CNTF up vs PBS |
| 4930473A06Rik | BC039810 | BC039810 // 4930473A06Rik // RIKEN cDNA 4930473A06 gene // 4 C3 // 320226 /// NM | -2.24 | CNTF down vs PBS |
| Tmem106a | NM_144830 | NM_144830 // Tmem106a // transmembrane protein 106A // 11 D // 217203 /// ENSMUS | 2.36 | CNTF up vs PBS |
| B3gat2 | NM_172124 | NM_172124 // B3gat2 // beta-1,3-glucuronyltransferase 2 (glucuronosyltransferase | -2.66 | CNTF down vs PBS |
| Itgb2 | NM_008404 | NM_008404 // Itgb2 // integrin beta 2 // 10 C1|10 41.5 cM // 16414 /// ENSMUST00 | 3.56 | CNTF up vs PBS |
| Muc2 | BC034197 | BC034197 // Muc2 // mucin 2 // 7 F5|7 68.99 cM // 17831 /// BC030862 // Muc2 // | -2.09 | CNTF down vs PBS |
| Ctsc | NM_009982 | NM_009982 // Ctsc // cathepsin C // 7 D3-E1.1 // 13032 /// ENSMUST00000032779 // | 3.46 | CNTF up vs PBS |
| Arpp19 | NM_001142655 | NM_001142655 // Arpp19 // cAMP-regulated phosphoprotein 19 // 9 E1 // 59046 /// | 4.54 | CNTF up vs PBS |
| Timm17a | NM_011590 | NM_011590 // Timm17a // translocase of inner mitochondrial membrane 17a // 1 F / | 3.78 | CNTF up vs PBS |
| Pxmp2 | NM_008993 | NM_008993 // Pxmp2 // peroxisomal membrane protein 2 // 5 F|5 59.0 cM // 19301 / | -2.63 | CNTF down vs PBS |
| Psmd12 | NM_025894 | NM_025894 // Psmd12 // proteasome (prosome, macropain) 26S subunit, non-ATPase, | 3.44 | CNTF up vs PBS |
| Ostf1 | NM_017375 | NM_017375 // Ostf1 // osteoclast stimulating factor 1 // 19 B|19 14.0 cM // 2040 | 2.88 | CNTF up vs PBS |
| Cd97 | NM_011925 | NM_011925 // Cd97 // CD97 antigen // 8 C2|8 38.0 cM // 26364 /// ENSMUST00000075 | 3.76 | CNTF up vs PBS |
| l7Rn6 | NM_026304 | NM_026304 // l7Rn6 // lethal, Chr 7, Rinchik 6 // 7 E1 // 67669 /// ENSMUST00000 | 2.28 | CNTF up vs PBS |
| Fam96a | NM_026635 | NM_026635 // Fam96a // family with sequence similarity 96, member A // 9 D // 68 | 5.22 | CNTF up vs PBS |
| Spcs3 | NM_029701 | NM_029701 // Spcs3 // signal peptidase complex subunit 3 homolog (S. cerevisiae) | 2.22 | CNTF up vs PBS |
| Rab5c | NM_024456 | NM_024456 // Rab5c // RAB5C, member RAS oncogene family // 11 D|11 60.0 cM // 19 | 2.13 | CNTF up vs PBS |
| Tgfbr1 | NM_009370 | NM_009370 // Tgfbr1 // transforming growth factor, beta receptor I // 4 B1|4 19. | 2.80 | CNTF up vs PBS |
| Kcmf1 | NM_019715 | NM_019715 // Kcmf1 // potassium channel modulatory factor 1 // 6 C3|6 30.5 cM // | 3.41 | CNTF up vs PBS |
| Clp1 | NM_133840 | NM_133840 // Clp1 // CLP1, cleavage and polyadenylation factor I subunit, homolo | 2.04 | CNTF up vs PBS |
| Nr4a1 | NM_010444 | NM_010444 // Nr4a1 // nuclear receptor subfamily 4, group A, member 1 // 15 F // | 2.16 | CNTF up vs PBS |
| Gm9918 | ENSMUST00000066324 | ENSMUST00000066324 // Gm9918 // predicted gene 9918 // 15 F3|15 // 432988 | -2.22 | CNTF down vs PBS |
| Mitf | NM_001113198 | NM_001113198 // Mitf // microphthalmia-associated transcription factor // 6 D3|6 | 2.80 | CNTF up vs PBS |
| Grn | NM_008175 | NM_008175 // Grn // granulin // 11 D|11 60.0 cM // 14824 /// ENSMUST00000049460 | 5.20 | CNTF up vs PBS |
| Sptlc2 | NM_011479 | NM_011479 // Sptlc2 // serine palmitoyltransferase, long chain base subunit 2 // | 3.58 | CNTF up vs PBS |
| Arl8b | NM_026011 | NM_026011 // Arl8b // ADP-ribosylation factor-like 8B // 6 E2 // 67166 /// ENSMU | 2.37 | CNTF up vs PBS |
| Ankzf1 | NM_026187 | NM_026187 // Ankzf1 // ankyrin repeat and zinc finger domain containing 1 // 1 C | -2.57 | CNTF down vs PBS |
| Ms4a6d | NM_026835 | NM_026835 // Ms4a6d // membrane-spanning 4-domains, subfamily A, member 6D // 19 | 7.45 | CNTF up vs PBS |
| Tufm | NM_172745 | NM_172745 // Tufm // Tu translation elongation factor, mitochondrial // 7 F3 // | -3.06 | CNTF down vs PBS |
| Golt1b | NM_025872 | NM_025872 // Golt1b // golgi transport 1 homolog B (S. cerevisiae) // 6 G1 // 66 | 4.63 | CNTF up vs PBS |
| Psap | NM_001146120 | NM_001146120 // Psap // prosaposin // 10 B4|10 35.0 cM // 19156 /// NM_011179 // | 5.18 | CNTF up vs PBS |
| Pik3cg | NM_020272 | NM_020272 // Pik3cg // phosphoinositide-3-kinase, catalytic, gamma polypeptide / | 4.14 | CNTF up vs PBS |
| Dync1i2 | NM_010064 | NM_010064 // Dync1i2 // dynein cytoplasmic 1 intermediate chain 2 // 2 C2|2 41.2 | 2.10 | CNTF up vs PBS |
| Ap2b1 | NM_001035854 | NM_001035854 // Ap2b1 // adaptor-related protein complex 2, beta 1 subunit // 11 | 2.90 | CNTF up vs PBS |
| Abcc3 | NM_029600 | NM_029600 // Abcc3 // ATP-binding cassette, sub-family C (CFTR/MRP), member 3 // | 3.64 | CNTF up vs PBS |
| Ogdh | NM_010956 | NM_010956 // Ogdh // oxoglutarate dehydrogenase (lipoamide) // 11 A1 // 18293 // | 2.25 | CNTF up vs PBS |
| Tlr4 | NM_021297 | NM_021297 // Tlr4 // toll-like receptor 4 // 4 C1|4 33.0 cM // 21898 /// ENSMUST | 3.70 | CNTF up vs PBS |
| Olfr1356 | NM_146308 | NM_146308 // Olfr1356 // olfactory receptor 1356 // --- // 258305 /// ENSMUST000 | -3.30 | CNTF down vs PBS |
| B4galt1 | NM_022305 | NM_022305 // B4galt1 // UDP-Gal:betaGlcNAc beta 1,4- galactosyltransferase, poly | 2.29 | CNTF up vs PBS |
| Tubb5 | NM_011655 | NM_011655 // Tubb5 // tubulin, beta 5 // 17 B1 // 22154 /// ENSMUST00000001566 / | 3.54 | CNTF up vs PBS |
| Lrrc2 | NM_028838 | NM_028838 // Lrrc2 // leucine rich repeat containing 2 // 9 F2|9 70.4 cM // 7424 | 5.16 | CNTF up vs PBS |
| Tnfrsf1b | NM_011610 | NM_011610 // Tnfrsf1b // tumor necrosis factor receptor superfamily, member 1b / | 2.49 | CNTF up vs PBS |
| Alg8 | NM_199035 | NM_199035 // Alg8 // asparagine-linked glycosylation 8 homolog (yeast, alpha-1,3 | 2.62 | CNTF up vs PBS |
| Lcor | NM_172154 | NM_172154 // Lcor // ligand dependent nuclear receptor corepressor // 19 C3 // 2 | 2.05 | CNTF up vs PBS |
| Galnt1 | NM_013814 | NM_013814 // Galnt1 // UDP-N-acetyl-alpha-D-galactosamine:polypeptide N-acetylga | 2.07 | CNTF up vs PBS |
| Dcun1d1 | NM_033623 | NM_033623 // Dcun1d1 // DCN1, defective in cullin neddylation 1, domain containi | 4.09 | CNTF up vs PBS |
| Sgk1 | NM_001161845 | NM_001161845 // Sgk1 // serum/glucocorticoid regulated kinase 1 // 10 A3 // 2039 | 2.11 | CNTF up vs PBS |
| Hiat1 | NM_008246 | NM_008246 // Hiat1 // hippocampus abundant gene transcript 1 // 3 G2 // 15247 // | 2.68 | CNTF up vs PBS |
| Apoe | NM_009696 | NM_009696 // Apoe // apolipoprotein E // 7 A3|7 4.0 cM // 11816 /// ENSMUST00000 | 2.75 | CNTF up vs PBS |
| Grcc10 | NM_013535 | NM_013535 // Grcc10 // gene rich cluster, C10 gene // 6 F2|6 60.22 cM // 14790 / | 2.78 | CNTF up vs PBS |
| Tmed9 | NM_026211 | NM_026211 // Tmed9 // transmembrane emp24 protein transport domain containing 9 | 2.15 | CNTF up vs PBS |
| Cept1 | NM_133869 | NM_133869 // Cept1 // choline/ethanolaminephosphotransferase 1 // 3 F2.3 // 9971 | 2.70 | CNTF up vs PBS |
| Mdh2 | NM_008617 | NM_008617 // Mdh2 // malate dehydrogenase 2, NAD (mitochondrial) // 5 G2|5 78.0 | 2.46 | CNTF up vs PBS |
| Slc8a1 | NM_011406 | NM_011406 // Slc8a1 // solute carrier family 8 (sodium/calcium exchanger), membe | 2.21 | CNTF up vs PBS |
| Ms4a6c | NM_028595 | NM_028595 // Ms4a6c // membrane-spanning 4-domains, subfamily A, member 6C // 19 | 12.00 | CNTF up vs PBS |
| Rdh16 | NM_009040 | NM_009040 // Rdh16 // retinol dehydrogenase 16 // 10 D3 // 19683 /// NM_080436 / | -2.14 | CNTF down vs PBS |
| Map3k7ip2 | NM_138667 | NM_138667 // Map3k7ip2 // mitogen-activated protein kinase kinase kinase 7 inter | 2.17 | CNTF up vs PBS |
| Rpl38 | NM_001048057 | NM_001048057 // Rpl38 // ribosomal protein L38 // 11 G1 // 67671 /// NM_023372 / | 6.02 | CNTF up vs PBS |
| Selenbp1 | NM_009150 | NM_009150 // Selenbp1 // selenium binding protein 1 // 3 F2.1|3 43.25 cM // 2034 | 2.88 | CNTF up vs PBS |
| Ly96 | NM_016923 | NM_016923 // Ly96 // lymphocyte antigen 96 // 1 A3 // 17087 /// NM_001159711 // | 3.12 | CNTF up vs PBS |
| Kars | NM_001130868 | NM_001130868 // Kars // lysyl-tRNA synthetase // 8 D3|8 55.0 cM // 85305 /// NM_ | 2.16 | CNTF up vs PBS |
| Jak1 | NM_146145 | NM_146145 // Jak1 // Janus kinase 1 // 4 C6|4 46.3 cM // 16451 /// ENSMUST000001 | 2.83 | CNTF up vs PBS |
| Psma1 | NM_011965 | NM_011965 // Psma1 // proteasome (prosome, macropain) subunit, alpha type 1 // 7 | 3.88 | CNTF up vs PBS |
| Elf1 | NM_007920 | NM_007920 // Elf1 // E74-like factor 1 // 14 D3 // 13709 /// NM_175554 // Clspn | 2.89 | CNTF up vs PBS |
| 9130011J15Rik | BC055692 | BC055692 // 9130011J15Rik // RIKEN cDNA 9130011J15 gene // 8 B3.3 // 66818 /// N | 3.78 | CNTF up vs PBS |
| Armc10 | NM_026034 | NM_026034 // Armc10 // armadillo repeat containing 10 // 5 A3 // 67211 /// ENSMU | 6.86 | CNTF up vs PBS |
| Cx3cr1 | NM_009987 | NM_009987 // Cx3cr1 // chemokine (C-X3-C) receptor 1 // 9 F4 // 13051 /// BC0126 | 3.60 | CNTF up vs PBS |
| Stard3nl | NM_024270 | NM_024270 // Stard3nl // STARD3 N-terminal like // 13 A3.1 // 76205 /// ENSMUST0 | 3.88 | CNTF up vs PBS |
| 1500011K16Rik | NR_015476 | NR_015476 // 1500011K16Rik // RIKEN cDNA 1500011K16 gene // 2 F1 // 67885 /// BC | 3.52 | CNTF up vs PBS |
| Morf4l1 | NM_001039147 | NM_001039147 // Morf4l1 // mortality factor 4 like 1 // 9 D1 // 21761 /// NM_024 | 7.10 | CNTF up vs PBS |
| Psmc1 | NM_008947 | NM_008947 // Psmc1 // protease (prosome, macropain) 26S subunit, ATPase 1 // 12 | 3.41 | CNTF up vs PBS |
| Cd68 | NM_009853 | NM_009853 // Cd68 // CD68 antigen // 11 B3|11 39.0 cM // 12514 /// ENSMUST000000 | 8.69 | CNTF up vs PBS |
| Itgb1 | NM_010578 | NM_010578 // Itgb1 // integrin beta 1 (fibronectin receptor beta) // 8 E2 // 164 | 3.13 | CNTF up vs PBS |
| Dcps | NM_027030 | NM_027030 // Dcps // decapping enzyme, scavenger // 9 A4 // 69305 /// ENSMUST000 | 2.38 | CNTF up vs PBS |
| Ssr2 | NM_025448 | NM_025448 // Ssr2 // signal sequence receptor, beta // 3 F1 // 66256 /// ENSMUST | 5.65 | CNTF up vs PBS |
| Hipk1 | NM_010432 | NM_010432 // Hipk1 // homeodomain interacting protein kinase 1 // 3 F3 // 15257 | 2.11 | CNTF up vs PBS |
| Tm6sf1 | NM_145375 | NM_145375 // Tm6sf1 // transmembrane 6 superfamily member 1 // 7 D1 // 107769 // | 5.17 | CNTF up vs PBS |
| Cdk3 | NR_004853 | NR_004853 // Cdk3 // cyclin-dependent kinase 3 // 11 E2 // 69681 /// ENSMUST0000 | -2.20 | CNTF down vs PBS |
| Olfr667 | NM_147060 | NM_147060 // Olfr667 // olfactory receptor 667 // --- // 259062 /// ENSMUST00000 | -2.12 | CNTF down vs PBS |
| 5730410E15Rik | NM_176998 | NM_176998 // 5730410E15Rik // RIKEN cDNA 5730410E15 gene // 15 B3.2 // 319613 // | -2.12 | CNTF down vs PBS |
| Zfp677 | NM_172486 | NM_172486 // Zfp677 // zinc finger protein 677 // 17 A3.2 // 210503 /// ENSMUST0 | 2.41 | CNTF up vs PBS |
| Defb6 | NM_054074 | NM_054074 // Defb6 // defensin beta 6 // 8 A1.3 // 116746 /// NM_001025351 // De | -2.19 | CNTF down vs PBS |
| Clec4d | NM_010819 | NM_010819 // Clec4d // C-type lectin domain family 4, member d // 6 F3|6 56.5 cM | 14.34 | CNTF up vs PBS |
| Lrrc10 | NM_146242 | NM_146242 // Lrrc10 // leucine rich repeat containing 10 // 10 D2 // 237560 /// | -2.26 | CNTF down vs PBS |
| Lcn8 | NM_033145 | NM_033145 // Lcn8 // lipocalin 8 // 2 A3 // 78076 /// ENSMUST00000038482 // Lcn8 | -2.03 | CNTF down vs PBS |
| Retn | NM_022984 | NM_022984 // Retn // resistin // 8 A1|8 0.37 cM // 57264 /// ENSMUST00000012849 | -2.29 | CNTF down vs PBS |
| Psma2 | NM_008944 | NM_008944 // Psma2 // proteasome (prosome, macropain) subunit, alpha type 2 // 1 | 9.39 | CNTF up vs PBS |
| D10Wsu52e | BC003288 | BC003288 // D10Wsu52e // DNA segment, Chr 10, Wayne State University 52, express | 3.79 | CNTF up vs PBS |
| Cops5 | NM_013715 | NM_013715 // Cops5 // COP9 (constitutive photomorphogenic) homolog, subunit 5 (A | 2.99 | CNTF up vs PBS |
| St13 | NM_133726 | NM_133726 // St13 // suppression of tumorigenicity 13 // 15 E2 // 70356 /// ENSM | 3.48 | CNTF up vs PBS |
| Zmpste24 | NM_172700 | NM_172700 // Zmpste24 // zinc metallopeptidase, STE24 homolog (S. cerevisiae) // | 3.70 | CNTF up vs PBS |
| Wasf2 | NM_153423 | NM_153423 // Wasf2 // WAS protein family, member 2 // 4 D2.3 // 242687 /// ENSMU | 2.77 | CNTF up vs PBS |
| Fam50a | NM_138607 | NM_138607 // Fam50a // family with sequence similarity 50, member A // X A7.3 // | 2.08 | CNTF up vs PBS |
| Mapre2 | NM_153058 | NM_153058 // Mapre2 // microtubule-associated protein, RP/EB family, member 2 // | 2.42 | CNTF up vs PBS |
| Cldnd1 | NM_171826 | NM_171826 // Cldnd1 // claudin domain containing 1 // 16 C1.2 // 224250 /// ENSM | 4.88 | CNTF up vs PBS |
| Il10ra | NM_008348 | NM_008348 // Il10ra // interleukin 10 receptor, alpha // 9 A5.2|9 26.0 cM // 161 | 2.53 | CNTF up vs PBS |
| Psmd8 | NM_026545 | NM_026545 // Psmd8 // proteasome (prosome, macropain) 26S subunit, non-ATPase, 8 | 2.17 | CNTF up vs PBS |
| Acss3 | NM_001142804 | NM_001142804 // Acss3 // acyl-CoA synthetase short-chain family member 3 // 10 D | -2.20 | CNTF down vs PBS |
| Gpr124 | NM_054044 | NM_054044 // Gpr124 // G protein-coupled receptor 124 // 8 A3 // 78560 /// ENSMU | -2.09 | CNTF down vs PBS |
| Taf9 | NM_027139 | NM_027139 // Taf9 // TAF9 RNA polymerase II, TATA box binding protein (TBP)-asso | 8.12 | CNTF up vs PBS |
| Cldn10 | NM_023878 | NM_023878 // Cldn10 // claudin 10 // 14 E4 // 58187 /// NM_001160096 // Cldn10 / | -2.19 | CNTF down vs PBS |
| Pdia3 | NM_007952 | NM_007952 // Pdia3 // protein disulfide isomerase associated 3 // 2 E5|2 69.0 cM | 2.23 | CNTF up vs PBS |
| Slc23a2 | NM_018824 | NM_018824 // Slc23a2 // solute carrier family 23 (nucleobase transporters), memb | 2.21 | CNTF up vs PBS |
| Ascc3 | NM_001146089 | NM_001146089 // Ascc3 // activating signal cointegrator 1 complex subunit 3 // 1 | 2.26 | CNTF up vs PBS |
| Necap2 | NM_025383 | NM_025383 // Necap2 // NECAP endocytosis associated 2 // 4 D3 // 66147 /// ENSMU | 2.43 | CNTF up vs PBS |
| Rpn2 | NM_019642 | NM_019642 // Rpn2 // ribophorin II // 2 H1|2 91.0 cM // 20014 /// NM_001039557 / | 2.28 | CNTF up vs PBS |
| Uso1 | NM_019490 | NM_019490 // Uso1 // USO1 homolog, vesicle docking protein (yeast) // 5 E3 // 56 | 2.01 | CNTF up vs PBS |
| Gm11428 | NM_001081957 | NM_001081957 // Gm11428 // predicted gene 11428 // 11 C|11 // 100034251 /// ENSM | 9.66 | CNTF up vs PBS |
| Idh1 | NM_001111320 | NM_001111320 // Idh1 // isocitrate dehydrogenase 1 (NADP+), soluble // 1 C2|1 29 | 4.63 | CNTF up vs PBS |
| Serpine2 | NM_009255 | NM_009255 // Serpine2 // serine (or cysteine) peptidase inhibitor, clade E, memb | 2.11 | CNTF up vs PBS |
| Cep250 | NM_001129999 | NM_001129999 // Cep250 // centrosomal protein 250 // 2 H1 // 16328 /// NM_001130 | -2.24 | CNTF down vs PBS |
| Fam188a | NM_024185 | NM_024185 // Fam188a // family with sequence similarity 188, member A // 2 A1 // | 2.64 | CNTF up vs PBS |
| Robld3 | NM_031248 | NM_031248 // Robld3 // roadblock domain containing 3 // 3 F1 // 83409 /// ENSMUS | 2.80 | CNTF up vs PBS |
| Soat1 | NM_009230 | NM_009230 // Soat1 // sterol O-acyltransferase 1 // 1 G3|1 81.6 cM // 20652 /// | 6.92 | CNTF up vs PBS |
| Stxbp4 | NM_011505 | NM_011505 // Stxbp4 // syntaxin binding protein 4 // 11 C // 20913 /// ENSMUST00 | -2.18 | CNTF down vs PBS |
| Ccl3 | NM_011337 | NM_011337 // Ccl3 // chemokine (C-C motif) ligand 3 // 11 C|11 47.59 cM // 20302 | 14.88 | CNTF up vs PBS |
| Vps26a | NM_133672 | NM_133672 // Vps26a // vacuolar protein sorting 26 homolog A (yeast) // 10 B3-B5 | 3.81 | CNTF up vs PBS |
| Saa1 | NM_009117 | NM_009117 // Saa1 // serum amyloid A 1 // 7 B4|7 23.5 cM // 20208 /// NM_011314 | -3.22 | CNTF down vs PBS |
| Mlkl | NM_029005 | NM_029005 // Mlkl // mixed lineage kinase domain-like // 8 D3 // 74568 /// ENSMU | 3.20 | CNTF up vs PBS |
| M6pr | NM_010749 | NM_010749 // M6pr // mannose-6-phosphate receptor, cation dependent // 6 F1|6 54 | 6.30 | CNTF up vs PBS |
| Nop58 | NM_018868 | NM_018868 // Nop58 // NOP58 ribonucleoprotein homolog (yeast) // 1 C1.3 // 55989 | 2.29 | CNTF up vs PBS |
| Hsp90b1 | NM_011631 | NM_011631 // Hsp90b1 // heat shock protein 90, beta (Grp94), member 1 // 10 C1|1 | 2.32 | CNTF up vs PBS |
| Zfp354c | NM_013922 | NM_013922 // Zfp354c // zinc finger protein 354C // 11 B1.3|11 25.0 cM // 30944 | -2.06 | CNTF down vs PBS |
| Slc30a5 | NM_022885 | NM_022885 // Slc30a5 // solute carrier family 30 (zinc transporter), member 5 // | 2.27 | CNTF up vs PBS |
| Samhd1 | NM_001139520 | NM_001139520 // Samhd1 // SAM domain and HD domain, 1 // 2 H2 // 56045 /// NM_01 | 2.35 | CNTF up vs PBS |
| Lig4 | NM_176953 | NM_176953 // Lig4 // ligase IV, DNA, ATP-dependent // 8 A1.1 // 319583 /// ENSMU | 2.09 | CNTF up vs PBS |
| Ap1g1 | NM_009677 | NM_009677 // Ap1g1 // adaptor protein complex AP-1, gamma 1 subunit // 8 D3|8 53 | 2.04 | CNTF up vs PBS |
| Slc35a5 | NM_028756 | NM_028756 // Slc35a5 // solute carrier family 35, member A5 // 16 B5|16 29.9 cM | 2.45 | CNTF up vs PBS |
| Slc17a3 | NM_134069 | NM_134069 // Slc17a3 // solute carrier family 17 (sodium phosphate), member 3 // | -2.27 | CNTF down vs PBS |
| Olfr76 | NM_146682 | NM_146682 // Olfr76 // olfactory receptor 76 // --- // 258677 /// ENSMUST0000008 | -2.48 | CNTF down vs PBS |
| Bub1 | NM_001113179 | NM_001113179 // Bub1 // budding uninhibited by benzimidazoles 1 homolog (S. cere | 2.35 | CNTF up vs PBS |
| Magt1 | NM_025952 | NM_025952 // Magt1 // magnesium transporter 1 // X D // 67075 /// DQ000005 // Ma | 4.10 | CNTF up vs PBS |
| Gna13 | NM_010303 | NM_010303 // Gna13 // guanine nucleotide binding protein, alpha 13 // 11 E1|11 6 | 2.45 | CNTF up vs PBS |
| Spop | NM_025287 | NM_025287 // Spop // speckle-type POZ protein // 11 D|11 55.6 cM // 20747 /// EN | 2.63 | CNTF up vs PBS |
| Edem1 | NM_138677 | NM_138677 // Edem1 // ER degradation enhancer, mannosidase alpha-like 1 // --- / | 3.43 | CNTF up vs PBS |
| Olfr67 | NM_013619 | NM_013619 // Olfr67 // olfactory receptor 67 // 7 E3 // 18368 /// ENSMUST0000006 | -2.15 | CNTF down vs PBS |
| Atp6v1g2 | NM_023179 | NM_023179 // Atp6v1g2 // ATPase, H+ transporting, lysosomal V1 subunit G2 // 17 | 2.16 | CNTF up vs PBS |
| Olfr392 | NM_147006 | NM_147006 // Olfr392 // olfactory receptor 392 // --- // 259008 /// ENSMUST00000 | -2.29 | CNTF down vs PBS |
| Hsph1 | NM_013559 | NM_013559 // Hsph1 // heat shock 105kDa/110kDa protein 1 // 5 G3|5 88.0 cM // 15 | 2.01 | CNTF up vs PBS |
| Srgn | NM_011157 | NM_011157 // Srgn // serglycin // 10 B4 // 19073 /// ENSMUST00000105446 // Srgn | 8.00 | CNTF up vs PBS |
| Abce1 | NM_015751 | NM_015751 // Abce1 // ATP-binding cassette, sub-family E (OABP), member 1 // 8 C | 2.43 | CNTF up vs PBS |
| Tas2r102 | NM_199153 | NM_199153 // Tas2r102 // taste receptor, type 2, member 102 // 6 G1|6 63.4 cM // | -2.60 | CNTF down vs PBS |
| Taldo1 | NM_011528 | NM_011528 // Taldo1 // transaldolase 1 // 7 F3-F4 // 21351 /// ENSMUST0000002657 | 2.86 | CNTF up vs PBS |
| Pank3 | NM_145962 | NM_145962 // Pank3 // pantothenate kinase 3 // 11 A4 // 211347 /// ENSMUST000000 | 5.13 | CNTF up vs PBS |
| Rnf4 | NM_011278 | NM_011278 // Rnf4 // ring finger protein 4 // 5 B2 // 19822 /// ENSMUST000000309 | 2.73 | CNTF up vs PBS |
| Mt4 | NM_008631 | NM_008631 // Mt4 // metallothionein 4 // 8 C5|8 45.0 cM // 17752 /// ENSMUST0000 | -2.84 | CNTF down vs PBS |
| Tcea1 | NM_011541 | NM_011541 // Tcea1 // transcription elongation factor A (SII) 1 // 1 A1 // 21399 | 7.11 | CNTF up vs PBS |
| Btk | NM_013482 | NM_013482 // Btk // Bruton agammaglobulinemia tyrosine kinase // X E3|X 51.0 cM | 3.69 | CNTF up vs PBS |
| Il1r2 | NM_010555 | NM_010555 // Il1r2 // interleukin 1 receptor, type II // 1 B|1 19.5 cM // 16178 | 3.28 | CNTF up vs PBS |
| Tm9sf3 | NM_133352 | NM_133352 // Tm9sf3 // transmembrane 9 superfamily member 3 // 19 C3 // 107358 / | 2.28 | CNTF up vs PBS |
| H2-Eb1 | NM_010382 | NM_010382 // H2-Eb1 // histocompatibility 2, class II antigen E beta // 17 B1|17 | 5.04 | CNTF up vs PBS |
| Galc | NM_008079 | NM_008079 // Galc // galactosylceramidase // 12 E|12 48.0 cM // 14420 /// NM_001 | 3.94 | CNTF up vs PBS |
| Il10rb | NM_008349 | NM_008349 // Il10rb // interleukin 10 receptor, beta // 16 C3.3|16 63.11 cM // 1 | 3.82 | CNTF up vs PBS |
| Slc35b1 | NM_016752 | NM_016752 // Slc35b1 // solute carrier family 35, member B1 // 11 D|11 55.5 cM / | 3.25 | CNTF up vs PBS |
| Il1b | NM_008361 | NM_008361 // Il1b // interleukin 1 beta // 2 F|2 73.0 cM // 16176 /// ENSMUST000 | 7.68 | CNTF up vs PBS |
| Shisa5 | NM_025858 | NM_025858 // Shisa5 // shisa homolog 5 (Xenopus laevis) // 9 F2 // 66940 /// NM_ | 2.80 | CNTF up vs PBS |
| 4933427E11Rik | ENSMUST00000056711 | ENSMUST00000056711 // 4933427E11Rik // RIKEN cDNA 4933427E11 gene // 15 D3 // 66 | -2.60 | CNTF down vs PBS |
| Lcp1 | NM_008879 | NM_008879 // Lcp1 // lymphocyte cytosolic protein 1 // 14 D3|14 42.0 cM // 18826 | 9.29 | CNTF up vs PBS |
| Ppp2r2a | NM_028032 | NM_028032 // Ppp2r2a // protein phosphatase 2 (formerly 2A), regulatory subunit | 2.77 | CNTF up vs PBS |
| 1810046J19Rik | NM_025559 | NM_025559 // 1810046J19Rik // RIKEN cDNA 1810046J19 gene // 11 D // 103742 /// E | 4.17 | CNTF up vs PBS |
| Nfe2l2 | NM_010902 | NM_010902 // Nfe2l2 // nuclear factor, erythroid derived 2, like 2 // 2 C3|2 45. | 2.78 | CNTF up vs PBS |
| Dld | NM_007861 | NM_007861 // Dld // dihydrolipoamide dehydrogenase // 12 A3|12 15.1 cM // 13382 | 2.97 | CNTF up vs PBS |
| E030010A14Rik | BC087877 | BC087877 // E030010A14Rik // RIKEN cDNA E030010A14 gene // 19 B // 226040 /// NM | -2.19 | CNTF down vs PBS |
| BC005537 | NM_024473 | NM_024473 // BC005537 // cDNA sequence BC005537 // 13 A3.2 // 79555 /// BC005537 | 3.89 | CNTF up vs PBS |
| Armcx6 | NM_001007578 | NM_001007578 // Armcx6 // armadillo repeat containing, X-linked 6 // X E3 // 278 | -2.30 | CNTF down vs PBS |
| Olfr777 | NM_146544 | NM_146544 // Olfr777 // olfactory receptor 777 // --- // 258537 /// NM_146548 // | -4.26 | CNTF down vs PBS |
| Clec4n | NM_020001 | NM_020001 // Clec4n // C-type lectin domain family 4, member n // 6 F3|6 55.0 cM | 10.34 | CNTF up vs PBS |
| H2-Aa | NM_010378 | NM_010378 // H2-Aa // histocompatibility 2, class II antigen A, alpha // 17 B1|1 | 8.48 | CNTF up vs PBS |
| Olfr291 | ENSMUST00000078172 | ENSMUST00000078172 // Olfr291 // olfactory receptor 291 // --- // 258410 | -2.03 | CNTF down vs PBS |
| Chi3l3 | NM_009892 | NM_009892 // Chi3l3 // chitinase 3-like 3 // 3 F2.2|3 50.5 cM // 12655 /// NM_14 | 4.35 | CNTF up vs PBS |
| Asf1a | NM_025541 | NM_025541 // Asf1a // ASF1 anti-silencing function 1 homolog A (S. cerevisiae) / | 2.73 | CNTF up vs PBS |
| A830039N20Rik | BC038501 | BC038501 // A830039N20Rik // RIKEN cDNA A830039N20 gene // 14 A3 // 268723 /// E | -5.04 | CNTF down vs PBS |
| Rgs1 | NM_015811 | NM_015811 // Rgs1 // regulator of G-protein signaling 1 // 1 F|1 78.0 cM // 5077 | 13.06 | CNTF up vs PBS |
| Sh3bgrl3 | NM_080559 | NM_080559 // Sh3bgrl3 // SH3 domain binding glutamic acid-rich protein-like 3 // | 4.22 | CNTF up vs PBS |
| Rsu1 | NM_009105 | NM_009105 // Rsu1 // Ras suppressor protein 1 // 2 A1 // 20163 /// ENSMUST000000 | 2.26 | CNTF up vs PBS |
| Spink12 | NM_030061 | NM_030061 // Spink12 // serine peptidase inhibitor, Kazal type 11 // 18 B3 // 78 | -2.50 | CNTF down vs PBS |
| B930011P16Rik | NM_207282 | NM_207282 // B930011P16Rik // RIKEN cDNA B930011P16 gene // 5 B1 // 403186 /// E | -2.21 | CNTF down vs PBS |
| Ptgs1 | NM_008969 | NM_008969 // Ptgs1 // prostaglandin-endoperoxide synthase 1 // 2 B|2 29.0 cM // | 2.82 | CNTF up vs PBS |
| Lipa | NM_021460 | NM_021460 // Lipa // lysosomal acid lipase A // 19 C1 // 16889 /// NM_001111100 | 9.74 | CNTF up vs PBS |
| Olfr149 | NM_207138 | NM_207138 // Olfr149 // olfactory receptor 149 // 9 A5.1 // 235256 /// ENSMUST00 | -2.22 | CNTF down vs PBS |
| Drg1 | NM_007879 | NM_007879 // Drg1 // developmentally regulated GTP binding protein 1 // 11 A1 // | 4.03 | CNTF up vs PBS |
| Lpcat2 | NM_173014 | NM_173014 // Lpcat2 // lysophosphatidylcholine acyltransferase 2 // 8 C5 // 2700 | 3.31 | CNTF up vs PBS |
| Dab2 | NM_023118 | NM_023118 // Dab2 // disabled homolog 2 (Drosophila) // 15 A|15 6.7 cM // 13132 | 6.72 | CNTF up vs PBS |
| Llph | NM_025431 | NM_025431 // Llph // LLP homolog, long-term synaptic facilitation (Aplysia) // 1 | 2.31 | CNTF up vs PBS |
| Cxcr4 | NM_009911 | NM_009911 // Cxcr4 // chemokine (C-X-C motif) receptor 4 // 1 E4|1 67.4 cM // 12 | 8.10 | CNTF up vs PBS |
| Npc2 | NM_023409 | NM_023409 // Npc2 // Niemann Pick type C2 // 12 D1 // 67963 /// ENSMUST000000216 | 5.58 | CNTF up vs PBS |
| Vwa5a | NM_172767 | NM_172767 // Vwa5a // von Willebrand factor A domain containing 5A // 9 B // 677 | 3.32 | CNTF up vs PBS |
| Stom | NM_013515 | NM_013515 // Stom // stomatin // 2 B-C1|2 24.0 cM // 13830 /// ENSMUST0000002824 | 2.24 | CNTF up vs PBS |
| Sod2 | NM_013671 | NM_013671 // Sod2 // superoxide dismutase 2, mitochondrial // 17 A1|17 7.6 cM // | 2.51 | CNTF up vs PBS |
| Car11 | NM_009800 | NM_009800 // Car11 // carbonic anhydrase 11 // 7 B2 // 12348 /// ENSMUST00000003 | -2.38 | CNTF down vs PBS |
| Lsr | NM_017405 | NM_017405 // Lsr // lipolysis stimulated lipoprotein receptor // 7 B1 // 54135 / | -2.03 | CNTF down vs PBS |
| Slc7a11 | NM_011990 | NM_011990 // Slc7a11 // solute carrier family 7 (cationic amino acid transporter | 4.89 | CNTF up vs PBS |
| Iqgap1 | NM_016721 | NM_016721 // Iqgap1 // IQ motif containing GTPase activating protein 1 // 7 D3|7 | 3.99 | CNTF up vs PBS |
| Csde1 | NM_144901 | NM_144901 // Csde1 // cold shock domain containing E1, RNA binding // 3 F2.2|3 4 | 3.36 | CNTF up vs PBS |
| Olfr608 | NM_146756 | NM_146756 // Olfr608 // olfactory receptor 608 // --- // 258751 /// ENSMUST00000 | -2.78 | CNTF down vs PBS |
| Gdap10 | BC052902 | BC052902 // Gdap10 // ganglioside-induced differentiation-associated-protein 10 | 4.40 | CNTF up vs PBS |
| Tbcb | NM_025548 | NM_025548 // Tbcb // tubulin folding cofactor B // 7 B1 // 66411 /// ENSMUST0000 | 2.53 | CNTF up vs PBS |
| Vps35 | NM_022997 | NM_022997 // Vps35 // vacuolar protein sorting 35 // 8 C3 // 65114 /// ENSMUST00 | 2.67 | CNTF up vs PBS |
| Abhd5 | NM_026179 | NM_026179 // Abhd5 // abhydrolase domain containing 5 // 9 F4 // 67469 /// ENSMU | 3.12 | CNTF up vs PBS |
| Uchl5 | NM_019562 | NM_019562 // Uchl5 // ubiquitin carboxyl-terminal esterase L5 // 1 F|1 77.8 cM / | 2.16 | CNTF up vs PBS |
| Fcrls | NM_030707 | NM_030707 // Fcrls // Fc receptor-like S, scavenger receptor // 3 F1 // 80891 // | 7.17 | CNTF up vs PBS |
| Pde3b | NM_011055 | NM_011055 // Pde3b // phosphodiesterase 3B, cGMP-inhibited // 7 F1|7 53.3 cM // | 2.43 | CNTF up vs PBS |
| Brcc3 | NM_145956 | NM_145956 // Brcc3 // BRCA1/BRCA2-containing complex, subunit 3 // X A7.3 // 210 | 3.45 | CNTF up vs PBS |
| Kynu | NM_027552 | NM_027552 // Kynu // kynureninase (L-kynurenine hydrolase) // 2 C1 // 70789 /// | 2.65 | CNTF up vs PBS |
| Cd38 | NM_007646 | NM_007646 // Cd38 // CD38 antigen // 5 B3|5 28.0 cM // 12494 /// ENSMUST00000030 | 3.86 | CNTF up vs PBS |
| Ccl2 | NM_011333 | NM_011333 // Ccl2 // chemokine (C-C motif) ligand 2 // 11 C-E1|11 46.5 cM // 202 | 3.95 | CNTF up vs PBS |
| Sae1 | NM_019748 | NM_019748 // Sae1 // SUMO1 activating enzyme subunit 1 // 7 A2|7 4.0 cM // 56459 | 2.18 | CNTF up vs PBS |
| Dpcd | NM_172639 | NM_172639 // Dpcd // deleted in primary ciliary dyskinesia // 19 C3 // 226162 // | 2.07 | CNTF up vs PBS |
| Cetn2 | NM_019405 | NM_019405 // Cetn2 // centrin 2 // X B|X 28.85 cM // 26370 /// ENSMUST0000011455 | 3.90 | CNTF up vs PBS |
| Patl1 | NM_172635 | NM_172635 // Patl1 // protein associated with topoisomerase II homolog 1 (yeast) | 2.03 | CNTF up vs PBS |
| Ppp6c | NM_024209 | NM_024209 // Ppp6c // protein phosphatase 6, catalytic subunit // 2 B // 67857 / | 2.23 | CNTF up vs PBS |
| Emp1 | NM_010128 | NM_010128 // Emp1 // epithelial membrane protein 1 // 6 G1|6 65.0 cM // 13730 // | 2.89 | CNTF up vs PBS |
| Litaf | NM_019980 | NM_019980 // Litaf // LPS-induced TN factor // 16 B1-B3 // 56722 /// ENSMUST0000 | 3.69 | CNTF up vs PBS |
| Tbc1d15 | NM_025706 | NM_025706 // Tbc1d15 // TBC1 domain family, member 15 // 10 D2 // 66687 /// ENSM | 3.34 | CNTF up vs PBS |
| Sh3kbp1 | NM_001135727 | NM_001135727 // Sh3kbp1 // SH3-domain kinase binding protein 1 // X F4 // 58194 | 2.57 | CNTF up vs PBS |
| Gtf2h2 | NM_022011 | NM_022011 // Gtf2h2 // general transcription factor II H, polypeptide 2 // 13 D1 | 2.47 | CNTF up vs PBS |
| Arl15 | NM_172595 | NM_172595 // Arl15 // ADP-ribosylation factor-like 15 // 13 D2.2 // 218639 /// E | 2.54 | CNTF up vs PBS |
| Glrx | NM_053108 | NM_053108 // Glrx // glutaredoxin // 13 C1|13 44.0 cM // 93692 /// ENSMUST000000 | 3.35 | CNTF up vs PBS |
| Sec24d | NM_027135 | NM_027135 // Sec24d // Sec24 related gene family, member D (S. cerevisiae) // 3 | 2.06 | CNTF up vs PBS |
| Nras | NM_010937 | NM_010937 // Nras // neuroblastoma ras oncogene // 3 F2.2|3 48.5 cM // 18176 /// | 5.01 | CNTF up vs PBS |
| Mcts2 | NM_025543 | NM_025543 // Mcts2 // malignant T cell amplified sequence 2 // 2 H1 // 66405 /// | -2.28 | CNTF down vs PBS |
| Obfc2a | NM_028696 | NM_028696 // Obfc2a // oligonucleotide/oligosaccharide-binding fold containing 2 | 3.24 | CNTF up vs PBS |
| Umps | NM_009471 | NM_009471 // Umps // uridine monophosphate synthetase // 16 B3 // 22247 /// ENSM | 2.54 | CNTF up vs PBS |
| Ndufa9 | NM_025358 | NM_025358 // Ndufa9 // NADH dehydrogenase (ubiquinone) 1 alpha subcomplex, 9 // | 3.57 | CNTF up vs PBS |
| Lrat | NM_023624 | NM_023624 // Lrat // lecithin-retinol acyltransferase (phosphatidylcholine-retin | -2.29 | CNTF down vs PBS |
| Lgals8 | NM_018886 | NM_018886 // Lgals8 // lectin, galactose binding, soluble 8 // 13 A1|13 8.0 cM / | 3.05 | CNTF up vs PBS |
| Ifit2 | NM_008332 | NM_008332 // Ifit2 // interferon-induced protein with tetratricopeptide repeats | 3.50 | CNTF up vs PBS |
| Anpep | NM_008486 | NM_008486 // Anpep // alanyl (membrane) aminopeptidase // 7 D3 // 16790 /// ENSM | 4.05 | CNTF up vs PBS |
| Lct | NM_001081078 | NM_001081078 // Lct // lactase // 1 E4 // 226413 /// ENSMUST00000073490 // Lct / | -2.09 | CNTF down vs PBS |
| Cript | NM_019936 | NM_019936 // Cript // cysteine-rich PDZ-binding protein // 17 E4 // 56724 /// EN | 3.30 | CNTF up vs PBS |
| Cs | NM_026444 | NM_026444 // Cs // citrate synthase // 10 D3 // 12974 /// NM_027945 // Csl // ci | 2.64 | CNTF up vs PBS |
| Gmfb | NM_022023 | NM_022023 // Gmfb // glia maturation factor, beta // 14 C1|14 18.0 cM // 63985 / | 3.26 | CNTF up vs PBS |
| F13a1 | NM_028784 | NM_028784 // F13a1 // coagulation factor XIII, A1 subunit // 13 A3.3 // 74145 // | 8.39 | CNTF up vs PBS |
| Xrcc4 | NM_028012 | NM_028012 // Xrcc4 // X-ray repair complementing defective repair in Chinese ham | 3.06 | CNTF up vs PBS |
| Pla2g4a | NM_008869 | NM_008869 // Pla2g4a // phospholipase A2, group IVA (cytosolic, calcium-dependen | 3.39 | CNTF up vs PBS |
| Csf1r | NM_001037859 | NM_001037859 // Csf1r // colony stimulating factor 1 receptor // 18 D|18 30.0 cM | 5.32 | CNTF up vs PBS |
| St8sia4 | NM_009183 | NM_009183 // St8sia4 // ST8 alpha-N-acetyl-neuraminide alpha-2,8-sialyltransfera | 3.11 | CNTF up vs PBS |
| Egr2 | NM_010118 | NM_010118 // Egr2 // early growth response 2 // 10 B5|10 35.0 cM // 13654 /// EN | 4.61 | CNTF up vs PBS |
| Art2b | NM_019915 | NM_019915 // Art2b // ADP-ribosyltransferase 2b // 7 E3|7 49.0 cM // 11872 /// N | 2.68 | CNTF up vs PBS |
| Msr1 | NM_031195 | NM_031195 // Msr1 // macrophage scavenger receptor 1 // 8 A4|8 20.0 cM // 20288 | 12.99 | CNTF up vs PBS |
| Psmd10 | NM_016883 | NM_016883 // Psmd10 // proteasome (prosome, macropain) 26S subunit, non-ATPase, | 3.91 | CNTF up vs PBS |
| Mrps23 | NM_024174 | NM_024174 // Mrps23 // mitochondrial ribosomal protein S23 // 11 C|11 48.0 cM // | 2.32 | CNTF up vs PBS |
| B3gnt4 | NM_198611 | NM_198611 // B3gnt4 // UDP-GlcNAc:betaGal beta-1,3-N-acetylglucosaminyltransfera | -2.19 | CNTF down vs PBS |
| Fgfbp1 | NM_008009 | NM_008009 // Fgfbp1 // fibroblast growth factor binding protein 1 // 5 B3 // 141 | -2.14 | CNTF down vs PBS |
| Ghitm | NM_078478 | NM_078478 // Ghitm // growth hormone inducible transmembrane protein // 14 B|14 | 2.95 | CNTF up vs PBS |
| Btg1 | NM_007569 | NM_007569 // Btg1 // B-cell translocation gene 1, anti-proliferative // 10 C3 // | 2.47 | CNTF up vs PBS |
| Emr1 | NM_010130 | NM_010130 // Emr1 // EGF-like module containing, mucin-like, hormone receptor-li | 9.24 | CNTF up vs PBS |
| Plxdc2 | NM_026162 | NM_026162 // Plxdc2 // plexin domain containing 2 // 2 A2-A3 // 67448 /// ENSMUS | 2.41 | CNTF up vs PBS |
| Uvrag | NM_178635 | NM_178635 // Uvrag // UV radiation resistance associated gene // 7 E2 // 78610 / | 2.56 | CNTF up vs PBS |
| Olfr1009 | NM_146572 | NM_146572 // Olfr1009 // olfactory receptor 1009 // --- // 258565 /// ENSMUST000 | -2.10 | CNTF down vs PBS |
| Ctnnb1 | NM_007614 | NM_007614 // Ctnnb1 // catenin (cadherin associated protein), beta 1 // 9 F4|9 7 | 2.20 | CNTF up vs PBS |
| Dek | NM_025900 | NM_025900 // Dek // DEK oncogene (DNA binding) // 13 B1|13 28.0 cM // 110052 /// | 2.34 | CNTF up vs PBS |
| Gnal | NM_177137 | NM_177137 // Gnal // guanine nucleotide binding protein, alpha stimulating, olfa | 2.01 | CNTF up vs PBS |
| Tlr8 | NM_133212 | NM_133212 // Tlr8 // toll-like receptor 8 // X F5 // 170744 /// ENSMUST000001121 | 2.41 | CNTF up vs PBS |
| Gpr137c | NM_027518 | NM_027518 // Gpr137c // G protein-coupled receptor 137C // 14 C1 // 70713 /// EN | -2.10 | CNTF down vs PBS |
| Pdia4 | NM_009787 | NM_009787 // Pdia4 // protein disulfide isomerase associated 4 // 6 B2.3|6 18.0 | 3.65 | CNTF up vs PBS |
| Cct4 | NM_009837 | NM_009837 // Cct4 // chaperonin containing Tcp1, subunit 4 (delta) // 11 A3.2|11 | 2.07 | CNTF up vs PBS |
| Eif5b | NM_198303 | NM_198303 // Eif5b // eukaryotic translation initiation factor 5B // 1 B // 2269 | 2.49 | CNTF up vs PBS |
| Phb2 | NM_007531 | NM_007531 // Phb2 // prohibitin 2 // 6 F2|6 60.22 cM // 12034 /// ENSMUST0000000 | 2.18 | CNTF up vs PBS |
| Olfr320 | NM_207230 | NM_207230 // Olfr320 // olfactory receptor 320 // 11 B1.3 // 216783 /// NM_00101 | -2.55 | CNTF down vs PBS |
| Leprotl1 | NM_026609 | NM_026609 // Leprotl1 // leptin receptor overlapping transcript-like 1 // 8 A4 / | 2.49 | CNTF up vs PBS |
| Sp100 | NM_013673 | NM_013673 // Sp100 // nuclear antigen Sp100 // 1 C5|1 50.0 cM // 20684 /// ENSMU | 7.37 | CNTF up vs PBS |
| Agps | NM_172666 | NM_172666 // Agps // alkylglycerone phosphate synthase // 2 C3 // 228061 /// ENS | 2.50 | CNTF up vs PBS |
| Cdkn1a | NM_007669 | NM_007669 // Cdkn1a // cyclin-dependent kinase inhibitor 1A (P21) // 17 A3.3|17 | 3.45 | CNTF up vs PBS |
| Cflar | NM_207653 | NM_207653 // Cflar // CASP8 and FADD-like apoptosis regulator // 1 C1.3|1 30.1 c | 2.31 | CNTF up vs PBS |
| Atp6v1c1 | NM_025494 | NM_025494 // Atp6v1c1 // ATPase, H+ transporting, lysosomal V1 subunit C1 // 15 | 3.12 | CNTF up vs PBS |
| Mrpl50 | NM_178603 | NM_178603 // Mrpl50 // mitochondrial ribosomal protein L50 // 4 B1|4 24.0 cM // | 2.83 | CNTF up vs PBS |
| Prpf38a | NM_172697 | NM_172697 // Prpf38a // PRP38 pre-mRNA processing factor 38 (yeast) domain conta | 2.12 | CNTF up vs PBS |
| Efhd2 | NM_025994 | NM_025994 // Efhd2 // EF hand domain containing 2 // 4 E1|4 69.8 cM // 27984 /// | 2.01 | CNTF up vs PBS |
| Rps9 | NM_029767 | NM_029767 // Rps9 // ribosomal protein S9 // 7 A1 // 76846 /// ENSMUST0000010862 | 3.16 | CNTF up vs PBS |
| Ston1 | NM_029858 | NM_029858 // Ston1 // stonin 1 // 17 E5 // 77057 /// ENSMUST00000064035 // Ston1 | -2.12 | CNTF down vs PBS |
| Rnf130 | NM_021540 | NM_021540 // Rnf130 // ring finger protein 130 // 11 B1.2 // 59044 /// ENSMUST00 | 2.81 | CNTF up vs PBS |
| Prelid1 | NM_025596 | NM_025596 // Prelid1 // PRELI domain containing 1 // 13 B2 // 66494 /// BC098241 | 3.23 | CNTF up vs PBS |
| B230220N19Rik | AK045674 | AK045674 // B230220N19Rik // RIKEN cDNA B230220N19 gene // 4 A5 // 319321 | -4.53 | CNTF down vs PBS |
| Lrrc33 | NM_146069 | NM_146069 // Lrrc33 // leucine rich repeat containing 33 // 16 B2 // 224109 /// | 2.94 | CNTF up vs PBS |
| Rps5 | NM_009095 | NM_009095 // Rps5 // ribosomal protein S5 // 7 A1 // 20103 /// ENSMUST0000010853 | 8.48 | CNTF up vs PBS |
| Ankfy1 | NM_009671 | NM_009671 // Ankfy1 // ankyrin repeat and FYVE domain containing 1 // 11 B4 // 1 | 2.45 | CNTF up vs PBS |
| Birc3 | NM_007464 | NM_007464 // Birc3 // baculoviral IAP repeat-containing 3 // 9 A2 // 11796 /// E | 2.97 | CNTF up vs PBS |
| Slc3a2 | NM_001161413 | NM_001161413 // Slc3a2 // solute carrier family 3 (activators of dibasic and neu | 3.29 | CNTF up vs PBS |
| Rps14 | NM_020600 | NM_020600 // Rps14 // ribosomal protein S14 // 18 E1|18 30.0 cM // 20044 /// ENS | 10.65 | CNTF up vs PBS |
| Psmb1 | NM_011185 | NM_011185 // Psmb1 // proteasome (prosome, macropain) subunit, beta type 1 // 17 | 2.51 | CNTF up vs PBS |
| Xkrx | NM_183319 | NM_183319 // Xkrx // X Kell blood group precursor related X linked // X E3 // 33 | -2.15 | CNTF down vs PBS |
| Wdr61 | NM_001025375 | NM_001025375 // Wdr61 // WD repeat domain 61 // 9 C // 66317 /// NM_023191 // Wd | 2.22 | CNTF up vs PBS |
| Degs1 | NM_007853 | NM_007853 // Degs1 // degenerative spermatocyte homolog 1 (Drosophila) // 1 H5 / | 2.28 | CNTF up vs PBS |
| Gcnt1 | NM_173442 | NM_173442 // Gcnt1 // glucosaminyl (N-acetyl) transferase 1, core 2 // 19 B|19 1 | 2.93 | CNTF up vs PBS |
| Ap1s2 | NM_026887 | NM_026887 // Ap1s2 // adaptor-related protein complex 1, sigma 2 subunit // X F5 | 2.93 | CNTF up vs PBS |
| Cwc15 | NM_023153 | NM_023153 // Cwc15 // CWC15 homolog (S. cerevisiae) // 9 A3 // 66070 /// ENSMUST | 3.11 | CNTF up vs PBS |
| 6530401N04Rik | BC089589 | BC089589 // 6530401N04Rik // RIKEN cDNA 6530401N04 gene // 12 C1 // 328092 /// N | 2.30 | CNTF up vs PBS |
| Psors1c2 | NM_020576 | NM_020576 // Psors1c2 // psoriasis susceptibility 1 candidate 2 (human) // 17 B1 | -2.14 | CNTF down vs PBS |
| Canx | NM_007597 | NM_007597 // Canx // calnexin // 11 B1.3|11 30.0 cM // 12330 /// NM_001110499 // | 2.04 | CNTF up vs PBS |
| Myo1f | NM_053214 | NM_053214 // Myo1f // myosin IF // 17 B-C|17 17.5 cM // 17916 /// ENSMUST0000008 | 4.03 | CNTF up vs PBS |
| Rab11a | NM_017382 | NM_017382 // Rab11a // RAB11a, member RAS oncogene family // 9 C // 53869 /// EN | 2.42 | CNTF up vs PBS |
| Elk4 | NM_007923 | NM_007923 // Elk4 // ELK4, member of ETS oncogene family // 1 E3-G // 13714 /// | 2.31 | CNTF up vs PBS |
| Olfr1030 | NM_146588 | NM_146588 // Olfr1030 // olfactory receptor 1030 // --- // 258581 /// ENSMUST000 | -2.59 | CNTF down vs PBS |
| Vps29 | NM_019780 | NM_019780 // Vps29 // vacuolar protein sorting 29 (S. pombe) // 5 F // 56433 /// | 2.62 | CNTF up vs PBS |
| Ctss | NM_021281 | NM_021281 // Ctss // cathepsin S // 3 F2.1|3 42.7 cM // 13040 /// ENSMUST0000001 | 8.42 | CNTF up vs PBS |
| Park7 | NM_020569 | NM_020569 // Park7 // Parkinson disease (autosomal recessive, early onset) 7 // | 4.17 | CNTF up vs PBS |
| Ocm | NM_033039 | NM_033039 // Ocm // oncomodulin // 5 G1-3|5 84.0 cM // 18261 | -2.11 | CNTF down vs PBS |
| Pik3ap1 | NM_031376 | NM_031376 // Pik3ap1 // phosphoinositide-3-kinase adaptor protein 1 // 19 D1 // | 5.25 | CNTF up vs PBS |
| Itga4 | NM_010576 | NM_010576 // Itga4 // integrin alpha 4 // 2 C3|2 46.0 cM // 16401 /// BC068313 / | 3.49 | CNTF up vs PBS |
| Xdh | NM_011723 | NM_011723 // Xdh // xanthine dehydrogenase // 17 E2|17 45.3 cM // 22436 /// ENSM | 2.87 | CNTF up vs PBS |
| Il2rg | NM_013563 | NM_013563 // Il2rg // interleukin 2 receptor, gamma chain // X D|X 38.0 cM // 16 | 4.66 | CNTF up vs PBS |
| Cd300a | NM_170758 | NM_170758 // Cd300a // CD300A antigen // 11 E2|11 78.0 cM // 217303 /// ENSMUST0 | 2.88 | CNTF up vs PBS |
| Unc50 | NM_026123 | NM_026123 // Unc50 // unc-50 homolog (C. elegans) // 1 B // 67387 /// ENSMUST000 | 2.07 | CNTF up vs PBS |
| Arhgef6 | NM_152801 | NM_152801 // Arhgef6 // Rac/Cdc42 guanine nucleotide exchange factor (GEF) 6 // | 2.55 | CNTF up vs PBS |
| Txnl4b | NM_175646 | NM_175646 // Txnl4b // thioredoxin-like 4B // 8 D3 // 234723 /// ENSMUST00000034 | 4.25 | CNTF up vs PBS |
| Acp5 | NM_001102404 | NM_001102404 // Acp5 // acid phosphatase 5, tartrate resistant // 9 A3|9 6.0 cM | 4.92 | CNTF up vs PBS |
| Olfr1243 | NM_146969 | NM_146969 // Olfr1243 // olfactory receptor 1243 // --- // 258971 /// ENSMUST000 | -2.41 | CNTF down vs PBS |
| Mobkl1b | NM_145571 | NM_145571 // Mobkl1b // MOB1, Mps One Binder kinase activator-like 1B (yeast) // | 4.97 | CNTF up vs PBS |
| Zc3h6 | NM_178404 | NM_178404 // Zc3h6 // zinc finger CCCH type containing 6 // 2 F1 // 78751 /// EN | -2.02 | CNTF down vs PBS |
| Cybb | NM_007807 | NM_007807 // Cybb // cytochrome b-245, beta polypeptide // X A1.1 // 13058 /// E | 14.96 | CNTF up vs PBS |
| Slc11a1 | NM_013612 | NM_013612 // Slc11a1 // solute carrier family 11 (proton-coupled divalent metal | 2.34 | CNTF up vs PBS |
| Ncstn | NM_021607 | NM_021607 // Ncstn // nicastrin // 1 H3|1 93.5 cM // 59287 /// ENSMUST0000000355 | 2.11 | CNTF up vs PBS |
| Erlin2 | NM_153592 | NM_153592 // Erlin2 // ER lipid raft associated 2 // 8 A2 // 244373 /// ENSMUST0 | 2.99 | CNTF up vs PBS |
| Ndufs2 | NM_153064 | NM_153064 // Ndufs2 // NADH dehydrogenase (ubiquinone) Fe-S protein 2 // 1 H3 // | 2.41 | CNTF up vs PBS |
| Tpm4 | NM_001001491 | NM_001001491 // Tpm4 // tropomyosin 4 // 8 B3.3 // 326618 /// ENSMUST00000003575 | 2.58 | CNTF up vs PBS |
|  | --- | --- | -2.36 | CNTF down vs PBS |
| 1110004F10Rik | NM_019772 | NM_019772 // 1110004F10Rik // RIKEN cDNA 1110004F10 gene // 7 F3 // 56372 /// EN | 3.25 | CNTF up vs PBS |
| Smu1 | NM_021535 | NM_021535 // Smu1 // smu-1 suppressor of mec-8 and unc-52 homolog (C. elegans) / | 2.62 | CNTF up vs PBS |
| Tagln2 | NM_178598 | NM_178598 // Tagln2 // transgelin 2 // 1 H3|1 94.2 cM // 21346 /// ENSMUST000001 | 2.19 | CNTF up vs PBS |
| H2-D1 | NM_010380 | NM_010380 // H2-D1 // histocompatibility 2, D region locus 1 // 17 B1|17 19.09 c | 17.09 | CNTF up vs PBS |
| Clec4e | NM_019948 | NM_019948 // Clec4e // C-type lectin domain family 4, member e // 6 F3|6 59.6 cM | 4.91 | CNTF up vs PBS |
| Trappc6b | NM_030057 | NM_030057 // Trappc6b // trafficking protein particle complex 6B // 12 C2 // 782 | 2.15 | CNTF up vs PBS |
| Aif1 | NM_019467 | NM_019467 // Aif1 // allograft inflammatory factor 1 // 17 B1|17 19.05 cM // 116 | 5.70 | CNTF up vs PBS |
| Carhsp1 | NM_025821 | NM_025821 // Carhsp1 // calcium regulated heat stable protein 1 // 16 A1|16 1.7 | 2.77 | CNTF up vs PBS |
| 1500035H01Rik | NM_023831 | NM_023831 // 1500035H01Rik // RIKEN cDNA 1500035H01 gene // 9 A5.2 // 76568 /// | 2.34 | CNTF up vs PBS |
| Oxa1l | NM_026936 | NM_026936 // Oxa1l // oxidase assembly 1-like // 14 C2 // 69089 /// NM_011405 // | 2.43 | CNTF up vs PBS |
| Cnpy2 | NM_019953 | NM_019953 // Cnpy2 // canopy 2 homolog (zebrafish) // 10 D3|10 72.0 cM // 56530 | 2.08 | CNTF up vs PBS |
| Fut1 | NM_008051 | NM_008051 // Fut1 // fucosyltransferase 1 // 7 B4|7 23.2 cM // 14343 /// ENSMUST | -2.52 | CNTF down vs PBS |
| Clic1 | NM_033444 | NM_033444 // Clic1 // chloride intracellular channel 1 // 17 B1|17 19.0 cM // 11 | 2.82 | CNTF up vs PBS |
| Otub1 | NM_134150 | NM_134150 // Otub1 // OTU domain, ubiquitin aldehyde binding 1 // 19 A // 107260 | 2.69 | CNTF up vs PBS |
| Mapk6 | NM_015806 | NM_015806 // Mapk6 // mitogen-activated protein kinase 6 // 9 D|9 38.0 cM // 507 | 2.19 | CNTF up vs PBS |
| Ywhae | NM_009536 | NM_009536 // Ywhae // tyrosine 3-monooxygenase/tryptophan 5-monooxygenase activa | 2.25 | CNTF up vs PBS |
| Myl1 | NM_021285 | NM_021285 // Myl1 // myosin, light polypeptide 1 // 1 C3|1 34.1 cM // 17901 /// | -2.04 | CNTF down vs PBS |
| 1700080E11Rik | NM_028562 | NM_028562 // 1700080E11Rik // RIKEN cDNA 1700080E11 gene // 9 F1 // 73532 /// EN | -2.01 | CNTF down vs PBS |
| Clcc1 | NM_145543 | NM_145543 // Clcc1 // chloride channel CLIC-like 1 // 3 F3 // 229725 /// ENSMUST | 2.28 | CNTF up vs PBS |
| Ctsd | NM_009983 | NM_009983 // Ctsd // cathepsin D // 7 F5 // 13033 /// ENSMUST00000066401 // Ctsd | 8.79 | CNTF up vs PBS |
| Tmem53 | NM_026837 | NM_026837 // Tmem53 // transmembrane protein 53 // 4 D1 // 68777 /// ENSMUST0000 | -2.36 | CNTF down vs PBS |
| Nrp2 | NM_001077403 | NM_001077403 // Nrp2 // neuropilin 2 // 1 C2 // 18187 /// NM_001077404 // Nrp2 / | 2.24 | CNTF up vs PBS |
| Cd33 | NM_001111058 | NM_001111058 // Cd33 // CD33 antigen // 7 B4|7 23.0 cM // 12489 /// NM_021293 // | 3.25 | CNTF up vs PBS |
| Etfb | NM_026695 | NM_026695 // Etfb // electron transferring flavoprotein, beta polypeptide // 7 B | 2.55 | CNTF up vs PBS |
| Emp3 | NM_010129 | NM_010129 // Emp3 // epithelial membrane protein 3 // 7 B4|7 24.5 cM // 13732 // | 3.19 | CNTF up vs PBS |
| Klk1b27 | NM_020268 | NM_020268 // Klk1b27 // kallikrein 1-related peptidase b27 // 7 B4|7 23.11 cM // | -2.25 | CNTF down vs PBS |
| Elovl7 | NM_029001 | NM_029001 // Elovl7 // ELOVL family member 7, elongation of long chain fatty aci | 2.16 | CNTF up vs PBS |
| Tpd52 | NM_001025261 | NM_001025261 // Tpd52 // tumor protein D52 // 3 A1-A2 // 21985 /// NM_001025263 | 3.60 | CNTF up vs PBS |
|  | --- | --- | -2.12 | CNTF down vs PBS |
| Sfxn1 | NM_027324 | NM_027324 // Sfxn1 // sideroflexin 1 // 13 B1|13 35.0 cM // 14057 /// ENSMUST000 | 2.05 | CNTF up vs PBS |
| Nfil3 | NM_017373 | NM_017373 // Nfil3 // nuclear factor, interleukin 3, regulated // 13 B1|13 32.0 | 2.37 | CNTF up vs PBS |
| Itgb5 | NM_001145884 | NM_001145884 // Itgb5 // integrin beta 5 // 16 B3 // 16419 /// NM_010580 // Itgb | 2.16 | CNTF up vs PBS |
| 1700019N12Rik | NM_001039494 | NM_001039494 // 1700019N12Rik // RIKEN cDNA 1700019N12 gene // 19 A // 67077 /// | -2.59 | CNTF down vs PBS |
| Klhl24 | NM_029436 | NM_029436 // Klhl24 // kelch-like 24 (Drosophila) // 16 B1 // 75785 /// ENSMUST0 | 3.37 | CNTF up vs PBS |
| Lrrc48 | NM_029044 | NM_029044 // Lrrc48 // leucine rich repeat containing 48 // 11 B2 // 74665 /// E | -2.34 | CNTF down vs PBS |
| Serinc3 | NM_012032 | NM_012032 // Serinc3 // serine incorporator 3 // 2 H3 // 26943 /// ENSMUST000000 | 3.79 | CNTF up vs PBS |
| Mapkapk3 | NM_178907 | NM_178907 // Mapkapk3 // mitogen-activated protein kinase-activated protein kina | 2.24 | CNTF up vs PBS |
| Fam49b | NM_144846 | NM_144846 // Fam49b // family with sequence similarity 49, member B // 15 D1 // | 2.80 | CNTF up vs PBS |
| Clec5a | NM_001038604 | NM_001038604 // Clec5a // C-type lectin domain family 5, member a // 6 B2 // 238 | 4.35 | CNTF up vs PBS |
| Olfr703 | NM_146596 | NM_146596 // Olfr703 // olfactory receptor 703 // --- // 258589 /// ENSMUST00000 | -3.42 | CNTF down vs PBS |
| Ptgr1 | NM_025968 | NM_025968 // Ptgr1 // prostaglandin reductase 1 // 4 C1 // 67103 /// ENSMUST0000 | 3.79 | CNTF up vs PBS |
| Ptgs2 | NM_011198 | NM_011198 // Ptgs2 // prostaglandin-endoperoxide synthase 2 // 1 H1|1 76.2 cM // | 5.41 | CNTF up vs PBS |
| Olfr866 | NM_146558 | NM_146558 // Olfr866 // olfactory receptor 866 // --- // 258551 /// ENSMUST00000 | -2.13 | CNTF down vs PBS |
| Trappc3 | NM_013718 | NM_013718 // Trappc3 // trafficking protein particle complex 3 // 4 D2.2 // 2709 | 4.68 | CNTF up vs PBS |
| Azin1 | NM_001102458 | NM_001102458 // Azin1 // antizyme inhibitor 1 // 15 C // 54375 /// NM_018745 // | 2.24 | CNTF up vs PBS |
| Mospd2 | NM_029730 | NM_029730 // Mospd2 // motile sperm domain containing 2 // X F5 // 76763 /// ENS | 2.96 | CNTF up vs PBS |
| Wrb | NM_207301 | NM_207301 // Wrb // tryptophan rich basic protein // 16 C4 // 71446 /// ENSMUST0 | 3.41 | CNTF up vs PBS |
| Zfp819 | NM_028913 | NM_028913 // Zfp819 // zinc finger protein 819 // 7 B4 // 74400 /// ENSMUST00000 | -2.14 | CNTF down vs PBS |
| Uchl1 | NM_011670 | NM_011670 // Uchl1 // ubiquitin carboxy-terminal hydrolase L1 // 5 C3.1|5 36.0 c | 2.28 | CNTF up vs PBS |
| Tug1 | NR_002322 | NR_002322 // Tug1 // taurine upregulated gene 1 // 11 A1 // 544752 /// NR_002321 | 3.30 | CNTF up vs PBS |
| Fndc3a | NM_207636 | NM_207636 // Fndc3a // fibronectin type III domain containing 3A // 14 D2 // 319 | 2.54 | CNTF up vs PBS |
| Cdc42 | NM_009861 | NM_009861 // Cdc42 // cell division cycle 42 homolog (S. cerevisiae) // 4 D3|4 6 | 4.56 | CNTF up vs PBS |
| Tyrobp | NM_011662 | NM_011662 // Tyrobp // TYRO protein tyrosine kinase binding protein // 7 B|7 10. | 9.15 | CNTF up vs PBS |
| Bcas2 | NM_026602 | NM_026602 // Bcas2 // breast carcinoma amplified sequence 2 // 3 F2.2 // 68183 / | 3.91 | CNTF up vs PBS |
| Saa3 | NM_011315 | NM_011315 // Saa3 // serum amyloid A 3 // 7 B4|7 23.5 cM // 20210 /// ENSMUST000 | 15.45 | CNTF up vs PBS |
| Pltp | NM_011125 | NM_011125 // Pltp // phospholipid transfer protein // 2 H3|2 93.0 cM // 18830 // | 3.17 | CNTF up vs PBS |
| Ctsl | NM_009984 | NM_009984 // Ctsl // cathepsin L // 13 B3|13 30.0 cM // 13039 /// ENSMUST0000002 | 2.68 | CNTF up vs PBS |
| Dom3z | NM_033613 | NM_033613 // Dom3z // DOM-3 homolog Z (C. elegans) // 17 B1 // 112403 /// ENSMUS | -2.13 | CNTF down vs PBS |
| Ankrd34c | NM_207260 | NM_207260 // Ankrd34c // ankyrin repeat domain 34C // 9 E3.1 // 330998 /// ENSMU | -2.19 | CNTF down vs PBS |
| Hspa13 | NR_027492 | NR_027492 // Hspa13 // heat shock protein 70 family, member 13 // 16 47.2 cM // | 4.46 | CNTF up vs PBS |
| Ip6k2 | NM_029634 | NM_029634 // Ip6k2 // inositol hexaphosphate kinase 2 // 9 F2 // 76500 /// BC039 | -2.05 | CNTF down vs PBS |
| Commd8 | NM_178599 | NM_178599 // Commd8 // COMM domain containing 8 // 5 C3.2|5 40.0 cM // 27784 /// | 2.10 | CNTF up vs PBS |
| Copa | NM_009938 | NM_009938 // Copa // coatomer protein complex subunit alpha // 1 H3|1 93.5 cM // | 2.49 | CNTF up vs PBS |
| Arf4 | NM_007479 | NM_007479 // Arf4 // ADP-ribosylation factor 4 // 14 A3|14 8.5 cM // 11843 /// N | 2.25 | CNTF up vs PBS |
| BC028528 | BC028528 | BC028528 // BC028528 // cDNA sequence BC028528 // 3 F2.1 // 229600 /// AF534879 | 2.81 | CNTF up vs PBS |
| Txnip | NM_001009935 | NM_001009935 // Txnip // thioredoxin interacting protein // 3 47.1 cM // 56338 / | 2.57 | CNTF up vs PBS |
| Ell2 | NM_138953 | NM_138953 // Ell2 // elongation factor RNA polymerase II 2 // 13 C1 // 192657 // | 2.06 | CNTF up vs PBS |
| Mr1 | NM_008209 | NM_008209 // Mr1 // major histocompatibility complex, class I-related // 1 H1 // | 2.06 | CNTF up vs PBS |
| Glod4 | NM_026029 | NM_026029 // Glod4 // glyoxalase domain containing 4 // 11 B5 // 67201 /// ENSMU | 3.04 | CNTF up vs PBS |
| Tcl1b4 | NM_013774 | NM_013774 // Tcl1b4 // T-cell leukemia/lymphoma 1B, 4 // 12 E // 27380 /// ENSMU | -2.46 | CNTF down vs PBS |
| Clec12a | NM_177686 | NM_177686 // Clec12a // C-type lectin domain family 12, member a // 6 F3 // 2324 | 4.13 | CNTF up vs PBS |
| Mbtps2 | NM_172307 | NM_172307 // Mbtps2 // membrane-bound transcription factor peptidase, site 2 // | 2.46 | CNTF up vs PBS |
| Fcgr3 | NM_010188 | NM_010188 // Fcgr3 // Fc receptor, IgG, low affinity III // 1 H3|1 92.3 cM // 14 | 4.09 | CNTF up vs PBS |
| Psma7 | NM_011969 | NM_011969 // Psma7 // proteasome (prosome, macropain) subunit, alpha type 7 // 2 | 3.03 | CNTF up vs PBS |
| Frrs1 | NM_001113478 | NM_001113478 // Frrs1 // ferric-chelate reductase 1 // 3 G1 // 20321 /// NM_0091 | 3.21 | CNTF up vs PBS |
| Sri | NM_001080974 | NM_001080974 // Sri // sorcin // 5 A1-h|5 1.0 cM // 109552 /// NM_025618 // Sri | 2.54 | CNTF up vs PBS |
| Ehd4 | NM_133838 | NM_133838 // Ehd4 // EH-domain containing 4 // 2 E5 // 98878 /// ENSMUST00000028 | 2.10 | CNTF up vs PBS |
| Cd93 | NM_010740 | NM_010740 // Cd93 // CD93 antigen // 2 G3|2 84.0 cM // 17064 /// ENSMUST00000099 | 6.77 | CNTF up vs PBS |
| Snx5 | NM_024225 | NM_024225 // Snx5 // sorting nexin 5 // 2 H1|2 80.0 cM // 69178 /// ENSMUST00000 | 2.87 | CNTF up vs PBS |
| Lgals12 | NM_019516 | NM_019516 // Lgals12 // lectin, galactose binding, soluble 12 // 19 A // 56072 / | -2.44 | CNTF down vs PBS |
| AA986860 | NM_177604 | NM_177604 // AA986860 // expressed sequence AA986860 // 1 E4 // 212439 /// ENSMU | -2.01 | CNTF down vs PBS |
| Rab11b | NM_008997 | NM_008997 // Rab11b // RAB11B, member RAS oncogene family // 17 B1|17 18.3 cM // | -2.45 | CNTF down vs PBS |
| N4bp1 | NM_030563 | NM_030563 // N4bp1 // NEDD4 binding protein 1 // 8 C3 // 80750 /// ENSMUST000000 | 2.15 | CNTF up vs PBS |
| Hsd17b11 | NM_053262 | NM_053262 // Hsd17b11 // hydroxysteroid (17-beta) dehydrogenase 11 // 5 E4 // 11 | 2.26 | CNTF up vs PBS |
| 2-Sep | NM_001159719 | NM_001159719 // Sept2 // septin 2 // 1 D // 18000 /// NM_010891 // Sept2 // sept | 2.97 | CNTF up vs PBS |
| 4921509C19Rik | BC119071 | BC119071 // 4921509C19Rik // RIKEN cDNA 4921509C19 gene // 2 G3 // 381393 /// NM | -2.75 | CNTF down vs PBS |
| Ucp2 | NM_011671 | NM_011671 // Ucp2 // uncoupling protein 2 (mitochondrial, proton carrier) // 7 E | 3.07 | CNTF up vs PBS |
| Olr1 | NM_138648 | NM_138648 // Olr1 // oxidized low density lipoprotein (lectin-like) receptor 1 / | 2.42 | CNTF up vs PBS |
| Psmb2 | NM_011970 | NM_011970 // Psmb2 // proteasome (prosome, macropain) subunit, beta type 2 // 4 | 3.04 | CNTF up vs PBS |
| Cul2 | NM_029402 | NM_029402 // Cul2 // cullin 2 // 18 A1 // 71745 /// ENSMUST00000080089 // Cul2 / | 2.08 | CNTF up vs PBS |
| Ganab | NM_008060 | NM_008060 // Ganab // alpha glucosidase 2 alpha neutral subunit // 19 A // 14376 | 2.01 | CNTF up vs PBS |
| Hexb | NM_010422 | NM_010422 // Hexb // hexosaminidase B // 13 D1|13 46.0 cM // 15212 /// ENSMUST00 | 5.36 | CNTF up vs PBS |
| Fbxl6 | NM_013909 | NM_013909 // Fbxl6 // F-box and leucine-rich repeat protein 6 // 15 D3 // 30840 | -2.09 | CNTF down vs PBS |
| Ppt1 | NM_008917 | NM_008917 // Ppt1 // palmitoyl-protein thioesterase 1 // 4 D1-D3|4 59.1 cM // 19 | 3.68 | CNTF up vs PBS |
| Spic | NM_011461 | NM_011461 // Spic // Spi-C transcription factor (Spi-1/PU.1 related) // 10 C // | 2.99 | CNTF up vs PBS |
| Eif3h | NM_080635 | NM_080635 // Eif3h // eukaryotic translation initiation factor 3, subunit H // 1 | 6.97 | CNTF up vs PBS |
| E430025E21Rik | BC067035 | BC067035 // E430025E21Rik // RIKEN cDNA E430025E21 gene // 15 D1 // 223593 /// B | 2.15 | CNTF up vs PBS |
| Psen1 | NM_008943 | NM_008943 // Psen1 // presenilin 1 // 12 D1|12 37.0 cM // 19164 /// ENSMUST00000 | 2.47 | CNTF up vs PBS |
| Abra | NM_175456 | NM_175456 // Abra // actin-binding Rho activating protein // 15 B3.1 // 223513 / | -2.43 | CNTF down vs PBS |
| Agfg1 | NM_010472 | NM_010472 // Agfg1 // ArfGAP with FG repeats 1 // 1 C5 // 15463 /// ENSMUST00000 | 2.21 | CNTF up vs PBS |
| Rpl23 | NM_022891 | NM_022891 // Rpl23 // ribosomal protein L23 // 11 D|11 58.0 cM // 65019 /// ENSM | 4.83 | CNTF up vs PBS |
| Gspt1 | NM_146066 | NM_146066 // Gspt1 // G1 to S phase transition 1 // 16 A1|16 3.8 cM // 14852 /// | 2.54 | CNTF up vs PBS |
| Olfr102 | NM_001011721 | NM_001011721 // Olfr102 // olfactory receptor 102 // 17 B1 // 258218 /// ENSMUST | -2.27 | CNTF down vs PBS |
| Naip5 | NM_010870 | NM_010870 // Naip5 // NLR family, apoptosis inhibitory protein 5 // 13 D1|13 55. | 2.32 | CNTF up vs PBS |
| Arih1 | NM_019927 | NM_019927 // Arih1 // ariadne ubiquitin-conjugating enzyme E2 binding protein ho | 2.44 | CNTF up vs PBS |
| Tmem85 | NM_026519 | NM_026519 // Tmem85 // transmembrane protein 85 // 2 E4 // 68032 /// NM_133648 / | 2.08 | CNTF up vs PBS |
| Tgm2 | NM_009373 | NM_009373 // Tgm2 // transglutaminase 2, C polypeptide // 2 H1|2 89.0 cM // 2181 | 2.27 | CNTF up vs PBS |
| Lce3f | NM_001018079 | NM_001018079 // Lce3f // late cornified envelope 3F // 3 F1 // 69520 /// ENSMUST | -2.16 | CNTF down vs PBS |
| Ypel1 | NM_023249 | NM_023249 // Ypel1 // yippee-like 1 (Drosophila) // 16 A3 // 106369 /// ENSMUST0 | -2.19 | CNTF down vs PBS |
| P2rx7 | NM_011027 | NM_011027 // P2rx7 // purinergic receptor P2X, ligand-gated ion channel, 7 // -- | 2.68 | CNTF up vs PBS |
| Cse1l | NM_023565 | NM_023565 // Cse1l // chromosome segregation 1-like (S. cerevisiae) // 2 H3|2 74 | 2.10 | CNTF up vs PBS |
| Cyp4f18 | NM_024444 | NM_024444 // Cyp4f18 // cytochrome P450, family 4, subfamily f, polypeptide 18 / | 3.45 | CNTF up vs PBS |
| Slc35f5 | NM_028787 | NM_028787 // Slc35f5 // solute carrier family 35, member F5 // 1 E3 // 74150 /// | 2.45 | CNTF up vs PBS |
| Vrk2 | NM_027260 | NM_027260 // Vrk2 // vaccinia related kinase 2 // 11 A3.3 // 69922 /// NM_025923 | 2.17 | CNTF up vs PBS |
| Snx13 | NM_001014973 | NM_001014973 // Snx13 // sorting nexin 13 // 12 A3 // 217463 /// ENSMUST00000048 | 2.10 | CNTF up vs PBS |
| Cd9 | NM_007657 | NM_007657 // Cd9 // CD9 antigen // 6 F3|6 58.0 cM // 12527 /// ENSMUST0000003249 | 2.66 | CNTF up vs PBS |
| Alg11 | NM_183142 | NM_183142 // Alg11 // asparagine-linked glycosylation 11 homolog (yeast, alpha-1 | 3.56 | CNTF up vs PBS |
| Tsen15 | NM_025677 | NM_025677 // Tsen15 // tRNA splicing endonuclease 15 homolog (S. cerevisiae) // | -2.71 | CNTF down vs PBS |
| Rnmt | NM_026440 | NM_026440 // Rnmt // RNA (guanine-7-) methyltransferase // 18 E1 // 67897 /// EN | 3.47 | CNTF up vs PBS |
| Slfn8 | NM_181545 | NM_181545 // Slfn8 // schlafen 8 // 11 C // 276950 /// NM_172796 // Slfn9 // sch | 3.52 | CNTF up vs PBS |
| Irf2bp1 | NM_178757 | NM_178757 // Irf2bp1 // interferon regulatory factor 2 binding protein 1 // 7 A3 | -2.03 | CNTF down vs PBS |
| Myo5a | NM_010864 | NM_010864 // Myo5a // myosin VA // 9 D|9 42.0 cM // 17918 /// ENSMUST00000036772 | 2.26 | CNTF up vs PBS |
| B630005N14Rik | NM_175312 | NM_175312 // B630005N14Rik // RIKEN cDNA B630005N14 gene // 6 A1 // 101148 /// E | 2.67 | CNTF up vs PBS |
| Eif2a | NM_001005509 | NM_001005509 // Eif2a // eukaryotic translation initiation factor 2a // 3 D|3 37 | 2.02 | CNTF up vs PBS |
| Gatad1 | NM_026033 | NM_026033 // Gatad1 // GATA zinc finger domain containing 1 // 5 A2 // 67210 /// | 2.01 | CNTF up vs PBS |
| Cd200r4 | NM_207244 | NM_207244 // Cd200r4 // CD200 receptor 4 // 16 B4 // 239849 /// NM_206535 // Cd2 | 3.58 | CNTF up vs PBS |
| Ranbp2 | NM_011240 | NM_011240 // Ranbp2 // RAN binding protein 2 // 10 B4|10 30.0 cM // 19386 /// EN | 2.28 | CNTF up vs PBS |
| Dctn3 | NM_016890 | NM_016890 // Dctn3 // dynactin 3 // 4 B1 // 53598 /// NM_001159565 // Dctn3 // d | 2.42 | CNTF up vs PBS |
| Ubl4 | NM_145405 | NM_145405 // Ubl4 // ubiquitin-like 4 // X A7.3|X 29.9 cM // 27643 /// ENSMUST00 | 2.75 | CNTF up vs PBS |
| Awat2 | NM_177746 | NM_177746 // Awat2 // acyl-CoA wax alcohol acyltransferase 2 // X C3 // 245532 / | -2.20 | CNTF down vs PBS |
| Anp32e | NM_023210 | NM_023210 // Anp32e // acidic (leucine-rich) nuclear phosphoprotein 32 family, m | 2.20 | CNTF up vs PBS |
| Rac2 | NM_009008 | NM_009008 // Rac2 // RAS-related C3 botulinum substrate 2 // 15 E1 // 19354 /// | 2.65 | CNTF up vs PBS |
| Med28 | NM_025895 | NM_025895 // Med28 // mediator of RNA polymerase II transcription, subunit 28 ho | 4.67 | CNTF up vs PBS |
| Ube2d3 | NM_025356 | NM_025356 // Ube2d3 // ubiquitin-conjugating enzyme E2D 3 (UBC4/5 homolog, yeast | 2.07 | CNTF up vs PBS |
| Plin2 | NM_007408 | NM_007408 // Plin2 // perilipin 2 // 4 C4|4 38.9 cM // 11520 /// ENSMUST00000000 | 7.91 | CNTF up vs PBS |
| Serp1 | NM_030685 | NM_030685 // Serp1 // stress-associated endoplasmic reticulum protein 1 // 3 D|3 | 3.67 | CNTF up vs PBS |
| Psmd6 | NM_025550 | NM_025550 // Psmd6 // proteasome (prosome, macropain) 26S subunit, non-ATPase, 6 | 2.09 | CNTF up vs PBS |
| Olfr594 | NM_207143 | NM_207143 // Olfr594 // olfactory receptor 594 // 7 E3 // 258246 /// ENSMUST0000 | -2.21 | CNTF down vs PBS |
| Negr1 | NM_001039094 | NM_001039094 // Negr1 // neuronal growth regulator 1 // 3 H4 // 320840 /// NM_17 | -2.21 | CNTF down vs PBS |
| Sh2d1a | NM_011364 | NM_011364 // Sh2d1a // SH2 domain protein 1A // X A5 // 20400 /// ENSMUST0000000 | -2.14 | CNTF down vs PBS |
| Entpd1 | NM_009848 | NM_009848 // Entpd1 // ectonucleoside triphosphate diphosphohydrolase 1 // 19 C3 | 3.33 | CNTF up vs PBS |
| Vamp3 | NM_009498 | NM_009498 // Vamp3 // vesicle-associated membrane protein 3 // 4 E1 // 22319 /// | 2.79 | CNTF up vs PBS |
| Tlr7 | NM_133211 | NM_133211 // Tlr7 // toll-like receptor 7 // X F5 // 170743 /// ENSMUST000001121 | 6.90 | CNTF up vs PBS |
| Bcap31 | NM_012060 | NM_012060 // Bcap31 // B-cell receptor-associated protein 31 // X A6 // 27061 // | 3.86 | CNTF up vs PBS |
| Ankrd13a | NM_026718 | NM_026718 // Ankrd13a // ankyrin repeat domain 13a // 5 F // 68420 /// NR_027872 | 2.25 | CNTF up vs PBS |
| Bst1 | NM_009763 | NM_009763 // Bst1 // bone marrow stromal cell antigen 1 // 5 B3|5 25.0 cM // 121 | 2.49 | CNTF up vs PBS |
| Wdyhv1 | NM_029734 | NM_029734 // Wdyhv1 // WDYHV motif containing 1 // 15 D2|15 // 76773 /// ENSMUST | 2.17 | CNTF up vs PBS |
| Hprt1 | NM_013556 | NM_013556 // Hprt1 // hypoxanthine guanine phosphoribosyl transferase 1 // X A6| | 2.21 | CNTF up vs PBS |
| Fam18b | BC115504 | BC115504 // Fam18b // family with sequence similarity 18, member B // 11 B2 // 6 | 2.08 | CNTF up vs PBS |
| Hsbp1 | NM_024219 | NM_024219 // Hsbp1 // heat shock factor binding protein 1 // 8 E1 // 68196 /// E | 2.87 | CNTF up vs PBS |
| Bxdc2 | NM_026396 | NM_026396 // Bxdc2 // brix domain containing 2 // 15 A1 // 67832 /// ENSMUST0000 | 4.35 | CNTF up vs PBS |
| Cfdp1 | NM_011801 | NM_011801 // Cfdp1 // craniofacial development protein 1 // 8 E1 // 23837 /// EN | 2.21 | CNTF up vs PBS |
| Cldn15 | NM_021719 | NM_021719 // Cldn15 // claudin 15 // 5 G2 // 60363 /// ENSMUST00000111093 // Cld | -2.03 | CNTF down vs PBS |
| Cd83 | NM_009856 | NM_009856 // Cd83 // CD83 antigen // 13 A4-5|13 24.0 cM // 12522 /// ENSMUST0000 | 3.25 | CNTF up vs PBS |
| Pafah1b2 | NM_008775 | NM_008775 // Pafah1b2 // platelet-activating factor acetylhydrolase, isoform 1b, | 2.33 | CNTF up vs PBS |
| Bccip | NM_025392 | NM_025392 // Bccip // BRCA2 and CDKN1A interacting protein // 7 F4 // 66165 /// | 5.03 | CNTF up vs PBS |
| Cfl2 | NM_007688 | NM_007688 // Cfl2 // cofilin 2, muscle // 12 C1 // 12632 /// ENSMUST00000078124 | 2.05 | CNTF up vs PBS |
| Mon1a | NM_028369 | NM_028369 // Mon1a // MON1 homolog A (yeast) // 9 F1 // 72825 /// NM_011251 // R | -2.06 | CNTF down vs PBS |
| Magea9 | NM_181855 | NM_181855 // Magea9 // melanoma antigen, family A, 9 // X A7.3 // 195772 /// ENS | -2.25 | CNTF down vs PBS |
| Hpse | NM_152803 | NM_152803 // Hpse // heparanase // 5 E4 // 15442 /// ENSMUST00000045617 // Hpse | 4.44 | CNTF up vs PBS |
| Ndufc2 | NM_024220 | NM_024220 // Ndufc2 // NADH dehydrogenase (ubiquinone) 1, subcomplex unknown, 2 | 2.48 | CNTF up vs PBS |
| Mdfic | NM_175088 | NM_175088 // Mdfic // MyoD family inhibitor domain containing // 6 A1 // 16543 / | 2.61 | CNTF up vs PBS |
| Ncapg2 | NM_133762 | NM_133762 // Ncapg2 // non-SMC condensin II complex, subunit G2 // 12 F2 // 7604 | 2.68 | CNTF up vs PBS |
| Rax | NM_013833 | NM_013833 // Rax // retina and anterior neural fold homeobox // 18 E1|18 38.0 cM | -2.12 | CNTF down vs PBS |
| Fgl2 | NM_008013 | NM_008013 // Fgl2 // fibrinogen-like protein 2 // 5 A3|5 7.0 cM // 14190 /// ENS | 2.77 | CNTF up vs PBS |
| Nop56 | NM_024193 | NM_024193 // Nop56 // NOP56 ribonucleoprotein homolog (yeast) // 2 F3 // 67134 / | 2.22 | CNTF up vs PBS |
| Rnft1 | NM_029788 | NM_029788 // Rnft1 // ring finger protein, transmembrane 1 // 11 C // 76892 /// | 2.78 | CNTF up vs PBS |
| P2ry6 | NM_183168 | NM_183168 // P2ry6 // pyrimidinergic receptor P2Y, G-protein coupled, 6 // 7 E3 | 2.65 | CNTF up vs PBS |
| Gm13103 | NM_177571 | NM_177571 // Gm13103 // predicted gene 13103 // 4 E1|4 // 194225 /// ENSMUST0000 | -2.38 | CNTF down vs PBS |
| Cd74 | NM_001042605 | NM_001042605 // Cd74 // CD74 antigen (invariant polypeptide of major histocompat | 4.10 | CNTF up vs PBS |
| Axl | NM_009465 | NM_009465 // Axl // AXL receptor tyrosine kinase // 7 A3-B1|7 6.0 cM // 26362 // | 2.02 | CNTF up vs PBS |
| Rai14 | NM_030690 | NM_030690 // Rai14 // retinoic acid induced 14 // 15 A2 // 75646 /// ENSMUST0000 | 2.12 | CNTF up vs PBS |
| Deb1 | NM_026794 | NM_026794 // Deb1 // differentially expressed in B16F10 1 // 9 F4 // 26901 /// E | 2.26 | CNTF up vs PBS |
| Zfp830 | NM_025884 | NM_025884 // Zfp830 // zinc finger protein 830 // 11 C // 66983 /// ENSMUST00000 | -2.08 | CNTF down vs PBS |
| BC006965 | NR_024085 | NR_024085 // BC006965 // cDNA sequence BC006965 // 11 E2 // 217294 | -3.38 | CNTF down vs PBS |
| Lgals9 | NM_010708 | NM_010708 // Lgals9 // lectin, galactose binding, soluble 9 // 11 B5 // 16859 // | 2.95 | CNTF up vs PBS |
| Olfr525 | NM_146956 | NM_146956 // Olfr525 // olfactory receptor 525 // 7 F4 // 258958 /// NM_028440 / | -2.25 | CNTF down vs PBS |
| Islr | NM_012043 | NM_012043 // Islr // immunoglobulin superfamily containing leucine-rich repeat / | -2.31 | CNTF down vs PBS |
| Pisd-ps1 | NR_003517 | NR_003517 // Pisd-ps1 // phosphatidylserine decarboxylase, pseudogene 1 // 11 A1 | -3.92 | CNTF down vs PBS |
| Uba3 | NM_011666 | NM_011666 // Uba3 // ubiquitin-like modifier activating enzyme 3 // 6 D3 // 2220 | 3.22 | CNTF up vs PBS |
| Rpa3 | NM_026632 | NM_026632 // Rpa3 // replication protein A3 // 6 A1 // 68240 /// ENSMUST00000012 | 3.55 | CNTF up vs PBS |
| Pilra | NM_153510 | NM_153510 // Pilra // paired immunoglobin-like type 2 receptor alpha // 5 G2 // | 4.16 | CNTF up vs PBS |
| Ccdc132 | NM_024260 | NM_024260 // Ccdc132 // coiled-coil domain containing 132 // 6 A1 // 73288 /// E | 2.24 | CNTF up vs PBS |
| Gpr177 | NM_026582 | NM_026582 // Gpr177 // G protein-coupled receptor 177 // 3 H4 // 68151 /// ENSMU | 2.36 | CNTF up vs PBS |
| Ppp1r7 | NM_023200 | NM_023200 // Ppp1r7 // protein phosphatase 1, regulatory (inhibitor) subunit 7 / | 3.31 | CNTF up vs PBS |
| Olfr153 | NM_206823 | NM_206823 // Olfr153 // olfactory receptor 153 // --- // 110511 /// NM_146658 // | -4.88 | CNTF down vs PBS |
| Olfr781 | NM_146728 | NM_146728 // Olfr781 // olfactory receptor 781 // --- // 258723 /// ENSMUST00000 | -2.11 | CNTF down vs PBS |
| Spp1 | NM_009263 | NM_009263 // Spp1 // secreted phosphoprotein 1 // 5 E5|5 56.0 cM // 20750 /// EN | 15.89 | CNTF up vs PBS |
| Arl6ip1 | NM_019419 | NM_019419 // Arl6ip1 // ADP-ribosylation factor-like 6 interacting protein 1 // | 4.08 | CNTF up vs PBS |
| Steap1 | NM_027399 | NM_027399 // Steap1 // six transmembrane epithelial antigen of the prostate 1 // | -2.03 | CNTF down vs PBS |
| Fn1 | NM_010233 | NM_010233 // Fn1 // fibronectin 1 // 1 C1-C5|1 36.1 cM // 14268 /// ENSMUST00000 | 3.87 | CNTF up vs PBS |
| Scamp2 | NM_022813 | NM_022813 // Scamp2 // secretory carrier membrane protein 2 // 9 B // 24044 /// | 2.12 | CNTF up vs PBS |
| Api5 | NM_007466 | NM_007466 // Api5 // apoptosis inhibitor 5 // 2 E1 // 11800 /// ENSMUST000000286 | 2.03 | CNTF up vs PBS |
| 2900010J23Rik | BC021748 | BC021748 // 2900010J23Rik // RIKEN cDNA 2900010J23 gene // 2 B // 72931 /// ENSM | 3.17 | CNTF up vs PBS |
| Sfpi1 | NM_011355 | NM_011355 // Sfpi1 // SFFV proviral integration 1 // 2 E3|2 47.5 cM // 20375 /// | 2.11 | CNTF up vs PBS |
| Atp6v0d2 | NM_175406 | NM_175406 // Atp6v0d2 // ATPase, H+ transporting, lysosomal V0 subunit D2 // 4 A | 4.36 | CNTF up vs PBS |
| Ptprc | NM_001111316 | NM_001111316 // Ptprc // protein tyrosine phosphatase, receptor type, C // 1 E4| | 6.52 | CNTF up vs PBS |
| Actr10 | NM_019785 | NM_019785 // Actr10 // ARP10 actin-related protein 10 homolog (S. cerevisiae) // | 2.25 | CNTF up vs PBS |
| Ube2l3 | NM_009456 | NM_009456 // Ube2l3 // ubiquitin-conjugating enzyme E2L 3 // 16 A3|16 10.2 cM // | 2.53 | CNTF up vs PBS |
| Nmd3 | NM_133787 | NM_133787 // Nmd3 // NMD3 homolog (S. cerevisiae) // 3 E1 // 97112 /// ENSMUST00 | 2.71 | CNTF up vs PBS |
| Fli1 | NM_008026 | NM_008026 // Fli1 // Friend leukemia integration 1 // 9 A4|9 16.0 cM // 14247 // | 3.64 | CNTF up vs PBS |
| Wipf1 | NM_153138 | NM_153138 // Wipf1 // WAS/WASL interacting protein family, member 1 // 2 C3|2 43 | 2.51 | CNTF up vs PBS |
| A930001N09Rik | BC113191 | BC113191 // A930001N09Rik // RIKEN cDNA A930001N09 gene // 17 B1 // 77128 /// NM | 2.53 | CNTF up vs PBS |
| 5730528L13Rik | NM_001145925 | NM_001145925 // 5730528L13Rik // RIKEN cDNA 5730528L13 gene // 4 B2 // 66665 /// | -2.11 | CNTF down vs PBS |
| Havcr2 | NM_134250 | NM_134250 // Havcr2 // hepatitis A virus cellular receptor 2 // 11 B1.1 // 17128 | 3.79 | CNTF up vs PBS |
| Itch | NM_008395 | NM_008395 // Itch // itchy, E3 ubiquitin protein ligase // 2 H1|2 89.0 cM // 163 | 2.08 | CNTF up vs PBS |
| Olfr380 | NM_147025 | NM_147025 // Olfr380 // olfactory receptor 380 // --- // 259027 /// ENSMUST00000 | -2.31 | CNTF down vs PBS |
| Tmem14c | NM_025387 | NM_025387 // Tmem14c // transmembrane protein 14C // 13 A3.3 // 66154 /// ENSMUS | 2.44 | CNTF up vs PBS |
| Penk | NM_001002927 | NM_001002927 // Penk // preproenkephalin // 4 A1|4 0.8 cM // 18619 /// ENSMUST00 | -2.22 | CNTF down vs PBS |
| C1galt1 | NM_052993 | NM_052993 // C1galt1 // core 1 synthase, glycoprotein-N-acetylgalactosamine 3-be | 2.69 | CNTF up vs PBS |
| Ctrl | NM_023182 | NM_023182 // Ctrl // chymotrypsin-like // 8 D3|8 53.0 cM // 109660 /// ENSMUST00 | -2.08 | CNTF down vs PBS |
| Clec4a1 | NM_199311 | NM_199311 // Clec4a1 // C-type lectin domain family 4, member a1 // 6 F2 // 2697 | 4.29 | CNTF up vs PBS |
| Rgs2 | NM_009061 | NM_009061 // Rgs2 // regulator of G-protein signaling 2 // 1 F|1 78.0 cM // 1973 | 3.63 | CNTF up vs PBS |
| Sar1b | NM_025535 | NM_025535 // Sar1b // SAR1 gene homolog B (S. cerevisiae) // 11 B1.3 // 66397 // | 3.42 | CNTF up vs PBS |
| Ufd1l | NM_011672 | NM_011672 // Ufd1l // ubiquitin fusion degradation 1 like // 16 B1-B4|16 11.75 c | 2.49 | CNTF up vs PBS |
| Refbp2 | NM_019484 | NM_019484 // Refbp2 // RNA and export factor binding protein 2 // 1 H3 // 56009 | 2.34 | CNTF up vs PBS |
| Ghrh | NM_010285 | NM_010285 // Ghrh // growth hormone releasing hormone // 2 H1|2 89.0 cM // 14601 | -2.29 | CNTF down vs PBS |
| D17Wsu104e | NM_080837 | NM_080837 // D17Wsu104e // DNA segment, Chr 17, Wayne State University 104, expr | 2.26 | CNTF up vs PBS |
| 1110018G07Rik | NM_178065 | NM_178065 // 1110018G07Rik // RIKEN cDNA 1110018G07 gene // 12 D2 // 68497 /// E | 2.11 | CNTF up vs PBS |
| Ccng1 | NM_009831 | NM_009831 // Ccng1 // cyclin G1 // 11 B1.1 // 12450 /// ENSMUST00000020576 // Cc | 2.57 | CNTF up vs PBS |
| Ssh2 | NM_177710 | NM_177710 // Ssh2 // slingshot homolog 2 (Drosophila) // 11 B5 // 237860 /// ENS | 2.41 | CNTF up vs PBS |
| Pon3 | NM_173006 | NM_173006 // Pon3 // paraoxonase 3 // 6 A1|6 0.5 cM // 269823 /// ENSMUST0000003 | 3.83 | CNTF up vs PBS |
| Rnd3 | NM_028810 | NM_028810 // Rnd3 // Rho family GTPase 3 // 2 C1.1 // 74194 /// ENSMUST000000172 | 2.71 | CNTF up vs PBS |
| Ugcg | NM_011673 | NM_011673 // Ugcg // UDP-glucose ceramide glucosyltransferase // 4 B3|4 32.0 cM | 2.14 | CNTF up vs PBS |
| Cd164 | NM_016898 | NM_016898 // Cd164 // CD164 antigen // 10 B2|10 25.0 cM // 53599 /// ENSMUST0000 | 2.73 | CNTF up vs PBS |
| Mrc1 | NM_008625 | NM_008625 // Mrc1 // mannose receptor, C type 1 // 2 A2|2 5.0 cM // 17533 /// EN | 3.29 | CNTF up vs PBS |
| Cish | NM_009895 | NM_009895 // Cish // cytokine inducible SH2-containing protein // 9 F1|9 59.0 cM | -2.18 | CNTF down vs PBS |
| Mff | NM_029409 | NM_029409 // Mff // mitochondrial fission factor // 1 C5 // 75734 /// ENSMUST000 | 2.33 | CNTF up vs PBS |
| Fkbp2 | NM_008020 | NM_008020 // Fkbp2 // FK506 binding protein 2 // 19 A // 14227 /// ENSMUST000000 | 3.39 | CNTF up vs PBS |
| Metap2 | NM_019648 | NM_019648 // Metap2 // methionine aminopeptidase 2 // 10 C2 // 56307 /// ENSMUST | 2.94 | CNTF up vs PBS |
| Rep15 | NM_025620 | NM_025620 // Rep15 // RAB15 effector protein // 6 G3 // 66532 /// ENSMUST0000003 | -2.66 | CNTF down vs PBS |
| Zfp580 | BC066001 | BC066001 // Zfp580 // zinc finger protein 580 // 7 A1 // 68992 /// ENSMUST000000 | -2.59 | CNTF down vs PBS |
| Dars | NM_177445 | NM_177445 // Dars // aspartyl-tRNA synthetase // 1 E4 // 226414 /// NM_145507 // | 2.21 | CNTF up vs PBS |
| V1ri1 | NM_145845 | NM_145845 // V1ri1 // vomeronasal 1 receptor, I1 // 13 A3.1 // 252907 /// NM_134 | -2.26 | CNTF down vs PBS |
| Spata17 | NM_028848 | NM_028848 // Spata17 // spermatogenesis associated 17 // 1 H5 // 74717 /// NM_02 | -2.10 | CNTF down vs PBS |
| Olfr544 | NM_020289 | NM_020289 // Olfr544 // olfactory receptor 544 // 7 E3 // 257926 /// ENSMUST0000 | -2.16 | CNTF down vs PBS |
| Naip2 | NM_010872 | NM_010872 // Naip2 // NLR family, apoptosis inhibitory protein 2 // 13 D1|13 54. | 3.58 | CNTF up vs PBS |
| Dcaf13 | NM_198606 | NM_198606 // Dcaf13 // DDB1 and CUL4 associated factor 13 // 15 B3.1 // 223499 / | 2.75 | CNTF up vs PBS |
| Tbx15 | NM_009323 | NM_009323 // Tbx15 // T-box 15 // 3 F2.2|3 49.0 cM // 21384 /// ENSMUST000000294 | -2.04 | CNTF down vs PBS |
| Capza2 | NM_007604 | NM_007604 // Capza2 // capping protein (actin filament) muscle Z-line, alpha 2 / | 3.07 | CNTF up vs PBS |
| Enpp1 | NM_008813 | NM_008813 // Enpp1 // ectonucleotide pyrophosphatase/phosphodiesterase 1 // 10 A | 3.33 | CNTF up vs PBS |
| V1rc15 | NM_134170 | NM_134170 // V1rc15 // vomeronasal 1 receptor, C15 // 6 C1 // 171188 /// ENSMUST | -2.17 | CNTF down vs PBS |
| Gli3 | NM_008130 | NM_008130 // Gli3 // GLI-Kruppel family member GLI3 // 13 A2|13 14.0 cM // 14634 | -2.33 | CNTF down vs PBS |
| 6720456B07Rik | BC010582 | BC010582 // 6720456B07Rik // RIKEN cDNA 6720456B07 gene // 6 E3 // 101314 /// EN | 2.21 | CNTF up vs PBS |
| Rpl29 | NM_009082 | NM_009082 // Rpl29 // ribosomal protein L29 // 9 F2-F4 // 19944 /// NR_027478 // | 7.24 | CNTF up vs PBS |
| Pnma1 | NM_027438 | NM_027438 // Pnma1 // paraneoplastic antigen MA1 // 12 D3 // 70481 /// ENSMUST00 | -2.00 | CNTF down vs PBS |
| Cd36 | NM_007643 | NM_007643 // Cd36 // CD36 antigen // 5 A3|5 2.0 cM // 12491 /// NM_001159555 // | 4.92 | CNTF up vs PBS |
| Ipo11 | NM_029665 | NM_029665 // Ipo11 // importin 11 // 13 D2.1 // 76582 /// ENSMUST00000080856 // | 2.13 | CNTF up vs PBS |
| Arf3 | NM_007478 | NM_007478 // Arf3 // ADP-ribosylation factor 3 // 15 F2 // 11842 /// ENSMUST0000 | 2.35 | CNTF up vs PBS |
| Slc9a9 | NM_177909 | NM_177909 // Slc9a9 // solute carrier family 9 (sodium/hydrogen exchanger), memb | 2.26 | CNTF up vs PBS |
| Taf3 | NM_027748 | NM_027748 // Taf3 // TAF3 RNA polymerase II, TATA box binding protein (TBP)-asso | 2.97 | CNTF up vs PBS |
| Rp2h | NM_133669 | NM_133669 // Rp2h // retinitis pigmentosa 2 homolog (human) // X A2 // 19889 /// | 2.67 | CNTF up vs PBS |
| Gpr84 | NM_030720 | NM_030720 // Gpr84 // G protein-coupled receptor 84 // 15 F3 // 80910 /// ENSMUS | 2.14 | CNTF up vs PBS |
| Olfr1501 | NM_146633 | NM_146633 // Olfr1501 // olfactory receptor 1501 // --- // 258626 /// ENSMUST000 | -2.63 | CNTF down vs PBS |
| Gab2 | NM_010248 | NM_010248 // Gab2 // growth factor receptor bound protein 2-associated protein 2 | 2.09 | CNTF up vs PBS |
| St8sia2 | NM_009181 | NM_009181 // St8sia2 // ST8 alpha-N-acetyl-neuraminide alpha-2,8-sialyltransfera | -2.32 | CNTF down vs PBS |
| 0610009B22Rik | BC024353 | BC024353 // 0610009B22Rik // RIKEN cDNA 0610009B22 gene // 11 B1.3 // 66050 /// | 4.79 | CNTF up vs PBS |
| Ddx20 | NM_017397 | NM_017397 // Ddx20 // DEAD (Asp-Glu-Ala-Asp) box polypeptide 20 // 3 F3 // 53975 | 2.16 | CNTF up vs PBS |
| 1700037C18Rik | NM_028484 | NM_028484 // 1700037C18Rik // RIKEN cDNA 1700037C18 gene // 16 A1 // 73261 /// N | -2.37 | CNTF down vs PBS |
| Abca1 | NM_013454 | NM_013454 // Abca1 // ATP-binding cassette, sub-family A (ABC1), member 1 // 4 A | 3.39 | CNTF up vs PBS |
| Ccl4 | NM_013652 | NM_013652 // Ccl4 // chemokine (C-C motif) ligand 4 // 11 C|11 47.6 cM // 20303 | 3.65 | CNTF up vs PBS |
| F11r | NM_172647 | NM_172647 // F11r // F11 receptor // 1 H2|1 93.3 cM // 16456 /// ENSMUST00000043 | 2.88 | CNTF up vs PBS |
| H2-K1 | NM_001001892 | NM_001001892 // H2-K1 // histocompatibility 2, K1, K region // 17 B1|17 18.44 cM | 2.55 | CNTF up vs PBS |
| Zscan12 | NM_016684 | NM_016684 // Zscan12 // zinc finger and SCAN domain containing 12 // 13 A3.1 // | -2.03 | CNTF down vs PBS |
| Med11 | NM_025397 | NM_025397 // Med11 // mediator of RNA polymerase II transcription, subunit 11 ho | 2.13 | CNTF up vs PBS |
| Gpr65 | NM_008152 | NM_008152 // Gpr65 // G-protein coupled receptor 65 // 12 E // 14744 /// ENSMUST | 5.85 | CNTF up vs PBS |
| Gfod2 | NM_027469 | NM_027469 // Gfod2 // glucose-fructose oxidoreductase domain containing 2 // 8 D | -2.15 | CNTF down vs PBS |
| Ext1 | NM_010162 | NM_010162 // Ext1 // exostoses (multiple) 1 // 15 C|15 26.55 cM // 14042 /// ENS | 2.47 | CNTF up vs PBS |
| Tceb1 | NM_026456 | NM_026456 // Tceb1 // transcription elongation factor B (SIII), polypeptide 1 // | 2.14 | CNTF up vs PBS |
| Ipo7 | NM_181517 | NM_181517 // Ipo7 // importin 7 // 7 F1 // 233726 /// ENSMUST00000084731 // Ipo7 | 2.28 | CNTF up vs PBS |
| Lcp2 | NM_010696 | NM_010696 // Lcp2 // lymphocyte cytosolic protein 2 // 11 A4 // 16822 /// ENSMUS | 3.24 | CNTF up vs PBS |
| Glyr1 | NM_001079814 | NM_001079814 // Glyr1 // glyoxylate reductase 1 homolog (Arabidopsis) // 16 A1|1 | 2.08 | CNTF up vs PBS |
| Orm1 | NM_008768 | NM_008768 // Orm1 // orosomucoid 1 // 4 B3|4 31.4 cM // 18405 /// NM_011016 // O | -2.04 | CNTF down vs PBS |
| Rtn4ip1 | NM_130892 | NM_130892 // Rtn4ip1 // reticulon 4 interacting protein 1 // 10 B2|10 29.0 cM // | 2.16 | CNTF up vs PBS |
| Cmklr1 | NM_008153 | NM_008153 // Cmklr1 // chemokine-like receptor 1 // 5 F // 14747 /// ENSMUST0000 | 2.89 | CNTF up vs PBS |
| Dock11 | NM_001009947 | NM_001009947 // Dock11 // dedicator of cytokinesis 11 // X A3.2 // 75974 /// ENS | 2.68 | CNTF up vs PBS |
| Psmc5 | NM_008950 | NM_008950 // Psmc5 // protease (prosome, macropain) 26S subunit, ATPase 5 // 11 | 3.17 | CNTF up vs PBS |
| Klra2 | NM_008462 | NM_008462 // Klra2 // killer cell lectin-like receptor, subfamily A, member 2 // | 2.95 | CNTF up vs PBS |
| 0610007C21Rik | NM_027855 | NM_027855 // 0610007C21Rik // RIKEN cDNA 0610007C21 gene // 5 B1 // 381629 /// N | 2.27 | CNTF up vs PBS |
| Stat3 | NM_213659 | NM_213659 // Stat3 // signal transducer and activator of transcription 3 // 11 D | 2.86 | CNTF up vs PBS |
| Ddx1 | NM_134040 | NM_134040 // Ddx1 // DEAD (Asp-Glu-Ala-Asp) box polypeptide 1 // 12 A1.1 // 1047 | 2.33 | CNTF up vs PBS |
| Psmb8 | NM_010724 | NM_010724 // Psmb8 // proteasome (prosome, macropain) subunit, beta type 8 (larg | 2.32 | CNTF up vs PBS |
| 2810417H13Rik | NM_026515 | NM_026515 // 2810417H13Rik // RIKEN cDNA 2810417H13 gene // 9 C // 68026 /// ENS | 3.28 | CNTF up vs PBS |
| Thbs1 | NM_011580 | NM_011580 // Thbs1 // thrombospondin 1 // 2 F1-F3|2 65.0 cM // 21825 /// ENSMUST | 8.16 | CNTF up vs PBS |
| Olfr290 | NM_146416 | NM_146416 // Olfr290 // olfactory receptor 290 // --- // 258411 /// NM_146415 // | -2.25 | CNTF down vs PBS |
| Casp1 | NM_009807 | NM_009807 // Casp1 // caspase 1 // 9 A1|9 1.0 cM // 12362 /// BC008152 // Casp1 | 3.71 | CNTF up vs PBS |
| Csnk2a2 | NM_009974 | NM_009974 // Csnk2a2 // casein kinase 2, alpha prime polypeptide // 8 D1|8 50.0 | 3.18 | CNTF up vs PBS |
| Fcrlb | NM_001029984 | NM_001029984 // Fcrlb // Fc receptor-like B // 1 H3 // 435653 /// ENSMUST0000009 | -2.04 | CNTF down vs PBS |
| Sfrs7 | NM_146083 | NM_146083 // Sfrs7 // splicing factor, arginine/serine-rich 7 // 17 E3 // 225027 | 3.18 | CNTF up vs PBS |
| Ktelc1 | NM_172380 | NM_172380 // Ktelc1 // KTEL (Lys-Tyr-Glu-Leu) containing 1 // 16 B4 // 224143 // | 2.07 | CNTF up vs PBS |
| Inpp5d | NM_010566 | NM_010566 // Inpp5d // inositol polyphosphate-5-phosphatase D // 1 C5|1 57.0 cM | 3.32 | CNTF up vs PBS |
| Slc7a8 | NM_016972 | NM_016972 // Slc7a8 // solute carrier family 7 (cationic amino acid transporter, | 2.34 | CNTF up vs PBS |
| Mmadhc | NM_133839 | NM_133839 // Mmadhc // methylmalonic aciduria (cobalamin deficiency) cblD type, | 2.76 | CNTF up vs PBS |
| Dnajc28 | NM_001099738 | NM_001099738 // Dnajc28 // DnaJ (Hsp40) homolog, subfamily C, member 28 // 16 C3 | -2.31 | CNTF down vs PBS |
| Usp48 | NM_130879 | NM_130879 // Usp48 // ubiquitin specific peptidase 48 // 4 D3 // 170707 /// ENSM | -4.61 | CNTF down vs PBS |
| Myd88 | NM_010851 | NM_010851 // Myd88 // myeloid differentiation primary response gene 88 // 9 F3|9 | 2.76 | CNTF up vs PBS |
| Phb | NM_008831 | NM_008831 // Phb // prohibitin // 11 D|11 55.6 cM // 18673 /// ENSMUST0000003637 | -2.32 | CNTF down vs PBS |
| Arhgap15 | NM_153820 | NM_153820 // Arhgap15 // Rho GTPase activating protein 15 // 2 B // 76117 /// NM | 2.83 | CNTF up vs PBS |
| Itga6 | NM_008397 | NM_008397 // Itga6 // integrin alpha 6 // 2 C2-C3|2 38.0 cM // 16403 /// ENSMUST | 3.45 | CNTF up vs PBS |
| Tank | NM_011529 | NM_011529 // Tank // TRAF family member-associated Nf-kappa B activator // 2 C1. | 2.25 | CNTF up vs PBS |
| Cox7a2 | NM_009945 | NM_009945 // Cox7a2 // cytochrome c oxidase, subunit VIIa 2 // 9 E1|9 42.0 cM // | 2.95 | CNTF up vs PBS |
| Txnrd1 | NM_001042523 | NM_001042523 // Txnrd1 // thioredoxin reductase 1 // 10 C1 // 50493 /// NM_00104 | 2.30 | CNTF up vs PBS |
| Rps6ka3 | NM_148945 | NM_148945 // Rps6ka3 // ribosomal protein S6 kinase polypeptide 3 // X F4|X 65.7 | 2.84 | CNTF up vs PBS |
| Ccrl2 | NM_017466 | NM_017466 // Ccrl2 // chemokine (C-C motif) receptor-like 2 // 9 F|9 70.1 cM // | 5.67 | CNTF up vs PBS |
| Copb2 | NM_015827 | NM_015827 // Copb2 // coatomer protein complex, subunit beta 2 (beta prime) // 9 | 2.17 | CNTF up vs PBS |
| Fcgr1 | NM_010186 | NM_010186 // Fcgr1 // Fc receptor, IgG, high affinity I // 3 F2.1|3 45.2 cM // 1 | 2.80 | CNTF up vs PBS |
| Fcgr2b | NM_001077189 | NM_001077189 // Fcgr2b // Fc receptor, IgG, low affinity IIb // 1 H3|1 92.3 cM / | 2.34 | CNTF up vs PBS |
| Gjb4 | NM_008127 | NM_008127 // Gjb4 // gap junction protein, beta 4 // 4 D1-D3|4 57.5 cM // 14621 | -2.03 | CNTF down vs PBS |
| Cbwd1 | NM_146097 | NM_146097 // Cbwd1 // COBW domain containing 1 // 19 B // 226043 /// ENSMUST0000 | 2.07 | CNTF up vs PBS |
| Cct2 | NM_007636 | NM_007636 // Cct2 // chaperonin containing Tcp1, subunit 2 (beta) // 10 D2 // 12 | 2.70 | CNTF up vs PBS |
| Zfp784 | NM_001039532 | NM_001039532 // Zfp784 // zinc finger protein 784 // 7 A1 // 654801 /// ENSMUST0 | -2.07 | CNTF down vs PBS |
| Rgr | NM_021340 | NM_021340 // Rgr // retinal G protein coupled receptor // 14 B // 57811 /// ENSM | -2.12 | CNTF down vs PBS |
| Hp1bp3 | NM_001122897 | NM_001122897 // Hp1bp3 // heterochromatin protein 1, binding protein 3 // 4 D3 / | 5.31 | CNTF up vs PBS |
| Sar1a | NM_009120 | NM_009120 // Sar1a // SAR1 gene homolog A (S. cerevisiae) // 10 B4 // 20224 /// | 2.26 | CNTF up vs PBS |
| Ugt2b5 | NM_009467 | NM_009467 // Ugt2b5 // UDP glucuronosyltransferase 2 family, polypeptide B5 // 5 | -2.01 | CNTF down vs PBS |
| Cmpk1 | NM_025647 | NM_025647 // Cmpk1 // cytidine monophosphate (UMP-CMP) kinase 1 // 4 C7 // 66588 | 2.65 | CNTF up vs PBS |
| Kifc1 | NM_053173 | NM_053173 // Kifc1 // kinesin family member C1 // 17 A3.3 // 16580 /// ENSMUST00 | -2.26 | CNTF down vs PBS |
| Prl | NM_011164 | NM_011164 // Prl // prolactin // 13 A3.1|13 14.0 cM // 19109 /// ENSMUST00000018 | -2.11 | CNTF down vs PBS |
| Olfr921 | NM_146782 | NM_146782 // Olfr921 // olfactory receptor 921 // --- // 258778 /// ENSMUST00000 | -3.91 | CNTF down vs PBS |
| Aim2 | NM_001013779 | NM_001013779 // Aim2 // absent in melanoma 2 // 1 H3 // 383619 /// ENSMUST000000 | 8.35 | CNTF up vs PBS |
| Snx3 | NM_017472 | NM_017472 // Snx3 // sorting nexin 3 // 10 B2|10 25.5 cM // 54198 /// ENSMUST000 | 2.49 | CNTF up vs PBS |
| Atox1 | NM_009720 | NM_009720 // Atox1 // ATX1 (antioxidant protein 1) homolog 1 (yeast) // 11 B1.3 | 3.08 | CNTF up vs PBS |
| Chrm4 | NM_007699 | NM_007699 // Chrm4 // cholinergic receptor, muscarinic 4 // 2 E1|2 49.0 cM // 12 | -2.08 | CNTF down vs PBS |
| Ndufa2 | NM_010885 | NM_010885 // Ndufa2 // NADH dehydrogenase (ubiquinone) 1 alpha subcomplex, 2 // | 2.54 | CNTF up vs PBS |
| Zfp617 | NM_133358 | NM_133358 // Zfp617 // zinc finger protein 617 // 8 B3.3 // 170938 /// ENSMUST00 | -2.44 | CNTF down vs PBS |
| Casp8ap2 | NM_011997 | NM_011997 // Casp8ap2 // caspase 8 associated protein 2 // 4 A5|4 11.4 cM // 268 | 2.06 | CNTF up vs PBS |
| Ttc1 | NM_133795 | NM_133795 // Ttc1 // tetratricopeptide repeat domain 1 // 11 B1.1 // 66827 /// E | 2.41 | CNTF up vs PBS |
| Slu7 | NM_148673 | NM_148673 // Slu7 // SLU7 splicing factor homolog (S. cerevisiae) // 11 A5-B1.1 | 2.15 | CNTF up vs PBS |
| Hpgd | NM_008278 | NM_008278 // Hpgd // hydroxyprostaglandin dehydrogenase 15 (NAD) // 8 B3.2 // 15 | 2.39 | CNTF up vs PBS |
| Tax1bp1 | NM_025816 | NM_025816 // Tax1bp1 // Tax1 (human T-cell leukemia virus type I) binding protei | 2.23 | CNTF up vs PBS |
| Itgam | NM_001082960 | NM_001082960 // Itgam // integrin alpha M // 7 F4 // 16409 /// NM_008401 // Itga | 5.28 | CNTF up vs PBS |
| Olfr167 | NM_146935 | NM_146935 // Olfr167 // olfactory receptor 167 // --- // 258937 /// ENSMUST00000 | -2.06 | CNTF down vs PBS |
| Psmd7 | NM_010817 | NM_010817 // Psmd7 // proteasome (prosome, macropain) 26S subunit, non-ATPase, 7 | 2.54 | CNTF up vs PBS |
| Olfr774 | NM_207620 | NM_207620 // Olfr774 // olfactory receptor 774 // 10 D3 // 258232 /// NM_207559 | -2.10 | CNTF down vs PBS |
| Pfkfb3 | NM_133232 | NM_133232 // Pfkfb3 // 6-phosphofructo-2-kinase/fructose-2,6-biphosphatase 3 // | 2.42 | CNTF up vs PBS |
| Dynlt3 | NM_025975 | NM_025975 // Dynlt3 // dynein light chain Tctex-type 3 // X A1.1 // 67117 /// NM | 3.22 | CNTF up vs PBS |
| Tmem50b | NM_030018 | NM_030018 // Tmem50b // transmembrane protein 50B // 16 C3.3 // 77975 /// ENSMUS | 4.84 | CNTF up vs PBS |
| 1700102P08Rik | NM_053216 | NM_053216 // 1700102P08Rik // RIKEN cDNA 1700102P08 gene // 9 F2 // 112418 /// E | -2.14 | CNTF down vs PBS |
| Olfr912 | NM_146810 | NM_146810 // Olfr912 // olfactory receptor 912 // --- // 258806 /// NM_146811 // | -3.17 | CNTF down vs PBS |
| Arhgap11a | NM_181416 | NM_181416 // Arhgap11a // Rho GTPase activating protein 11A // 2 E4 // 228482 // | 2.55 | CNTF up vs PBS |
| Wnk2 | NM_029361 | NM_029361 // Wnk2 // WNK lysine deficient protein kinase 2 // 13 B1 // 75607 /// | -2.02 | CNTF down vs PBS |
| Prkar1a | NM_021880 | NM_021880 // Prkar1a // protein kinase, cAMP dependent regulatory, type I, alpha | 2.93 | CNTF up vs PBS |
| Itfg1 | NM_028007 | NM_028007 // Itfg1 // integrin alpha FG-GAP repeat containing 1 // 8 C4|8 39.0 c | 2.10 | CNTF up vs PBS |
| Ilk | NM_010562 | NM_010562 // Ilk // integrin linked kinase // 7 E1 // 16202 /// NM_001161724 // | 2.14 | CNTF up vs PBS |
| Narg1 | NM_053089 | NM_053089 // Narg1 // NMDA receptor-regulated gene 1 // 3 D // 74838 /// ENSMUST | 2.31 | CNTF up vs PBS |
| Ddx3x | NM_010028 | NM_010028 // Ddx3x // DEAD/H (Asp-Glu-Ala-Asp/His) box polypeptide 3, X-linked / | 2.20 | CNTF up vs PBS |
| Rab24 | NM_009000 | NM_009000 // Rab24 // RAB24, member RAS oncogene family // 13 B1|13 36.0 cM // 1 | 2.05 | CNTF up vs PBS |
| Rps8 | NM_009098 | NM_009098 // Rps8 // ribosomal protein S8 // 4 D1 // 20116 /// BC081465 // Rps8 | -2.23 | CNTF down vs PBS |
| Olfr780 | NM_146284 | NM_146284 // Olfr780 // olfactory receptor 780 // --- // 258281 /// ENSMUST00000 | -2.36 | CNTF down vs PBS |
| Il1rn | NM_031167 | NM_031167 // Il1rn // interleukin 1 receptor antagonist // 2 A3|2 10.0 cM // 161 | 2.43 | CNTF up vs PBS |
| Mcart1 | NM_001009949 | NM_001009949 // Mcart1 // mitochondrial carrier triple repeat 1 // 4 B1 // 23012 | 2.76 | CNTF up vs PBS |
| Zfp148 | NM_011749 | NM_011749 // Zfp148 // zinc finger protein 148 // 16 B3|16 21.1 cM // 22661 /// | 2.01 | CNTF up vs PBS |
| EG634650 | NM_001039647 | NM_001039647 // EG634650 // predicted gene, EG634650 // 5 E5 // 634650 /// NM_19 | -2.12 | CNTF down vs PBS |
| Plk1 | NM_011121 | NM_011121 // Plk1 // polo-like kinase 1 (Drosophila) // 7 F3|7 59.0 cM // 18817 | -2.47 | CNTF down vs PBS |
| Olfr519 | NM_207160 | NM_207160 // Olfr519 // olfactory receptor 519 // 7 E3 // 277935 /// ENSMUST0000 | -2.62 | CNTF down vs PBS |
| Zdhhc21 | NM_026647 | NM_026647 // Zdhhc21 // zinc finger, DHHC domain containing 21 // 4 C3 // 68268 | 3.21 | CNTF up vs PBS |
| Nmb | NM_026523 | NM_026523 // Nmb // neuromedin B // 7 D3 // 68039 /// ENSMUST00000026817 // Nmb | -2.56 | CNTF down vs PBS |
| Igfbp7 | NM_001159518 | NM_001159518 // Igfbp7 // insulin-like growth factor binding protein 7 // 5 C3.3 | -2.58 | CNTF down vs PBS |
| Psmb7 | NM_011187 | NM_011187 // Psmb7 // proteasome (prosome, macropain) subunit, beta type 7 // 2 | 4.89 | CNTF up vs PBS |
| Ier3ip1 | NM_025409 | NM_025409 // Ier3ip1 // immediate early response 3 interacting protein 1 // 18 E | 2.33 | CNTF up vs PBS |
| Cpsf7 | NM_172302 | NM_172302 // Cpsf7 // cleavage and polyadenylation specific factor 7 // 19 A // | 2.00 | CNTF up vs PBS |
| Gfra2 | NM_008115 | NM_008115 // Gfra2 // glial cell line derived neurotrophic factor family recepto | -2.09 | CNTF down vs PBS |
| Olfr24 | NM_146606 | NM_146606 // Olfr24 // olfactory receptor 24 // 9 A3 // 18322 /// ENSMUST0000006 | -2.24 | CNTF down vs PBS |
| Matr3 | NM_010771 | NM_010771 // Matr3 // matrin 3 // 18 C|18 15.0 cM // 17184 /// ENSMUST0000004151 | 2.95 | CNTF up vs PBS |
| Sec11c | NM_025468 | NM_025468 // Sec11c // SEC11 homolog C (S. cerevisiae) // 18 E1 // 66286 /// ENS | 2.18 | CNTF up vs PBS |
|  | --- | --- | -2.12 | CNTF down vs PBS |
| Atp6v1b2 | NM_007509 | NM_007509 // Atp6v1b2 // ATPase, H+ transporting, lysosomal V1 subunit B2 // 8 B | 2.66 | CNTF up vs PBS |
| Atp6v0b | NM_033617 | NM_033617 // Atp6v0b // ATPase, H+ transporting, lysosomal V0 subunit B // 4 D2. | 2.62 | CNTF up vs PBS |
| 6030445D17Rik | ENSMUST00000084593 | ENSMUST00000084593 // 6030445D17Rik // RIKEN cDNA 6030445D17 gene // 4 D3 // 320 | -2.01 | CNTF down vs PBS |
| Kank1 | NM_181404 | NM_181404 // Kank1 // KN motif and ankyrin repeat domains 1 // 19 B // 107351 // | -2.16 | CNTF down vs PBS |
| Olfr344 | NM_146628 | NM_146628 // Olfr344 // olfactory receptor 344 // --- // 258621 /// ENSMUST00000 | -2.34 | CNTF down vs PBS |
| A530079E22Rik | AK080193 | AK080193 // A530079E22Rik // RIKEN cDNA A530079E22 gene // 1 D // 319977 | -2.06 | CNTF down vs PBS |
| Tmed10 | NM_026775 | NM_026775 // Tmed10 // transmembrane emp24-like trafficking protein 10 (yeast) / | 2.18 | CNTF up vs PBS |
| 3632451O06Rik | BC023359 | BC023359 // 3632451O06Rik // RIKEN cDNA 3632451O06 gene // 14 C1 // 67419 /// NM | -2.35 | CNTF down vs PBS |
| Dpagt1 | NM_007875 | NM_007875 // Dpagt1 // dolichyl-phosphate (UDP-N-acetylglucosamine) acetylglucos | 2.04 | CNTF up vs PBS |
| Rnasek | NM_173742 | NM_173742 // Rnasek // ribonuclease, RNase K // 11 B3|11 37.0 cM // 52898 /// EN | 2.20 | CNTF up vs PBS |
| Ift52 | NM_172150 | NM_172150 // Ift52 // intraflagellar transport 52 homolog (Chlamydomonas) // 2 H | 2.53 | CNTF up vs PBS |
| Coro1c | NM_011779 | NM_011779 // Coro1c // coronin, actin binding protein 1C // --- // 23790 /// ENS | 2.79 | CNTF up vs PBS |
| Slco1c1 | NM_021471 | NM_021471 // Slco1c1 // solute carrier organic anion transporter family, member | -2.30 | CNTF down vs PBS |
| Prkcb | NM_008855 | NM_008855 // Prkcb // protein kinase C, beta // 7 F3|7 60.0 cM // 18751 /// ENSM | 3.42 | CNTF up vs PBS |
| Lbh | NM_029999 | NM_029999 // Lbh // limb-bud and heart // --- // 77889 /// BC052470 // Lbh // li | 3.08 | CNTF up vs PBS |
| Ccr5 | NM_009917 | NM_009917 // Ccr5 // chemokine (C-C motif) receptor 5 // 9 72.0 cM // 12774 /// | 6.52 | CNTF up vs PBS |
| Coro1a | NM_009898 | NM_009898 // Coro1a // coronin, actin binding protein 1A // 7 F3|7 62.5 cM // 12 | 3.02 | CNTF up vs PBS |
| Rnasel | NM_011882 | NM_011882 // Rnasel // ribonuclease L (2', 5'-oligoisoadenylate synthetase-depen | 3.18 | CNTF up vs PBS |
|  | --- | --- | -2.29 | CNTF down vs PBS |
| Olfr893 | NM_146336 | NM_146336 // Olfr893 // olfactory receptor 893 // --- // 258333 /// ENSMUST00000 | -2.18 | CNTF down vs PBS |
| E330009P21Rik | NM_177069 | NM_177069 // E330009P21Rik // RIKEN cDNA E330009P21 gene // 9 F2 // 320082 /// E | -2.06 | CNTF down vs PBS |
| Tmed4 | NM_134020 | NM_134020 // Tmed4 // transmembrane emp24 protein transport domain containing 4 | 2.42 | CNTF up vs PBS |
| 1700034I23Rik | BC117739 | BC117739 // 1700034I23Rik // RIKEN cDNA 1700034I23 gene // 3 B // 73297 /// NM_0 | -3.25 | CNTF down vs PBS |
| 6330578E17Rik | NM_198006 | NM_198006 // 6330578E17Rik // RIKEN cDNA 6330578E17 gene // 1 B // 76178 /// ENS | 6.65 | CNTF up vs PBS |
| Snx1 | NM_019727 | NM_019727 // Snx1 // sorting nexin 1 // --- // 56440 /// ENSMUST00000034946 // S | 2.16 | CNTF up vs PBS |
| Olfr234 | NM_001001807 | NM_001001807 // Olfr234 // olfactory receptor 234 // --- // 258502 /// ENSMUST00 | -2.81 | CNTF down vs PBS |
| Nrd1 | NM_146150 | NM_146150 // Nrd1 // nardilysin, N-arginine dibasic convertase, NRD convertase 1 | 2.37 | CNTF up vs PBS |
| Chm | NM_018818 | NM_018818 // Chm // choroidermia // X E1 // 12662 /// ENSMUST00000026607 // Chm | 2.48 | CNTF up vs PBS |
| Snrpd3 | NM_026095 | NM_026095 // Snrpd3 // small nuclear ribonucleoprotein D3 // 10 C1 // 67332 /// | 2.78 | CNTF up vs PBS |
| Gpi1 | NM_008155 | NM_008155 // Gpi1 // glucose phosphate isomerase 1 // 7 B1|7 11.0 cM // 14751 // | 4.55 | CNTF up vs PBS |
| Neil1 | NM_028347 | NM_028347 // Neil1 // nei endonuclease VIII-like 1 (E. coli) // 9 C // 72774 /// | -2.07 | CNTF down vs PBS |
| Epha10 | NM_177671 | NM_177671 // Epha10 // Eph receptor A10 // 4 D2.2 // 230735 /// ENSMUST000000593 | -2.76 | CNTF down vs PBS |
| Gpx1 | NM_008160 | NM_008160 // Gpx1 // glutathione peroxidase 1 // 9 F1|9 57.0 cM // 14775 /// ENS | 3.24 | CNTF up vs PBS |
| Dnajc10 | NM_024181 | NM_024181 // Dnajc10 // DnaJ (Hsp40) homolog, subfamily C, member 10 // 2 D // 6 | 2.29 | CNTF up vs PBS |
| Usp14 | NM_021522 | NM_021522 // Usp14 // ubiquitin specific peptidase 14 // 18 A1|18 2.0 cM // 5902 | 2.12 | CNTF up vs PBS |
| D030056L22Rik | BC020125 | BC020125 // D030056L22Rik // RIKEN cDNA D030056L22 gene // 19 B // 225995 /// NM | 3.52 | CNTF up vs PBS |
| Cox4i1 | NM_009941 | NM_009941 // Cox4i1 // cytochrome c oxidase subunit IV isoform 1 // 8 E1|8 64.0 | 3.77 | CNTF up vs PBS |
| Prpf8 | NM_138659 | NM_138659 // Prpf8 // pre-mRNA processing factor 8 // 11 B5|11 45.0 cM // 192159 | 2.01 | CNTF up vs PBS |
| Gclc | NM_010295 | NM_010295 // Gclc // glutamate-cysteine ligase, catalytic subunit // 9 D-E|9 42. | 2.52 | CNTF up vs PBS |
| Hist1h4i | NM_175656 | NM_175656 // Hist1h4i // histone cluster 1, H4i // --- // 319158 /// BC092144 // | -2.85 | CNTF down vs PBS |
| Rhox6 | NM_008955 | NM_008955 // Rhox6 // reproductive homeobox 6 // X A3.3 // 19202 /// NM_00102508 | -2.01 | CNTF down vs PBS |
| Ergic2 | NM_026168 | NM_026168 // Ergic2 // ERGIC and golgi 2 // 6 G3 // 67456 /// NM_026355 // Ergic | 3.09 | CNTF up vs PBS |
| Hist1h2aa | NM_175658 | NM_175658 // Hist1h2aa // histone cluster 1, H2aa // --- // 319163 /// ENSMUST00 | -2.24 | CNTF down vs PBS |
| Ddb1 | NM_015735 | NM_015735 // Ddb1 // damage specific DNA binding protein 1 // 19 centromere|19 5 | 2.22 | CNTF up vs PBS |
| Olfr1378 | NM_146910 | NM_146910 // Olfr1378 // olfactory receptor 1378 // --- // 258912 /// ENSMUST000 | -2.29 | CNTF down vs PBS |
| BC065397 | BC065397 | BC065397 // BC065397 // cDNA sequence BC065397 // X F1 // 436230 /// ENSMUST0000 | -2.21 | CNTF down vs PBS |
| Atp6ap2 | NM_027439 | NM_027439 // Atp6ap2 // ATPase, H+ transporting, lysosomal accessory protein 2 / | -2.37 | CNTF down vs PBS |
| Rps25 | NM_024266 | NM_024266 // Rps25 // ribosomal protein S25 // 9 A5.2 // 75617 /// ENSMUST000000 | -3.02 | CNTF down vs PBS |
| Akr1c13 | NM_013778 | NM_013778 // Akr1c13 // aldo-keto reductase family 1, member C13 // 13 A1 // 273 | 3.75 | CNTF up vs PBS |
| Ndufa10 | NM_024197 | NM_024197 // Ndufa10 // NADH dehydrogenase (ubiquinone) 1 alpha subcomplex 10 // | 2.44 | CNTF up vs PBS |
| Samd8 | NM_026283 | NM_026283 // Samd8 // sterile alpha motif domain containing 8 // 14 B // 67630 / | 2.21 | CNTF up vs PBS |
| Hbxip | NM_026774 | NM_026774 // Hbxip // hepatitis B virus x interacting protein // 3 F3 // 68576 / | 17.01 | CNTF up vs PBS |
| Cdc27 | NM_145436 | NM_145436 // Cdc27 // cell division cycle 27 homolog (S. cerevisiae) // 11 E1|11 | 2.13 | CNTF up vs PBS |
| Tox4 | NM_023434 | NM_023434 // Tox4 // TOX high mobility group box family member 4 // 14 C2 // 268 | -3.25 | CNTF down vs PBS |
| Dnajc7 | NM_019795 | NM_019795 // Dnajc7 // DnaJ (Hsp40) homolog, subfamily C, member 7 // 11 D|11 60 | 2.57 | CNTF up vs PBS |
| Sry | NM_011564 | NM_011564 // Sry // sex determining region of Chr Y // Y A1 // 21674 /// ENSMUST | -2.10 | CNTF down vs PBS |
| Ergic3 | NM_025516 | NM_025516 // Ergic3 // ERGIC and golgi 3 // 2 H2|2 92.0 cM // 66366 /// ENSMUST0 | 2.76 | CNTF up vs PBS |
| Arrdc3 | NM_001042591 | NM_001042591 // Arrdc3 // arrestin domain containing 3 // 13 C3 // 105171 /// EN | 2.33 | CNTF up vs PBS |
| Apol9b | NM_173743 | NM_173743 // Apol9b // apolipoprotein L 9b // 15 E1 // 71898 /// NM_173786 // Ap | -2.22 | CNTF down vs PBS |
| Rnpep | NM_145417 | NM_145417 // Rnpep // arginyl aminopeptidase (aminopeptidase B) // 1 E4 // 21561 | 2.28 | CNTF up vs PBS |
| Vps36 | NM_027338 | NM_027338 // Vps36 // vacuolar protein sorting 36 (yeast) // 8 A3 // 70160 /// E | 2.07 | CNTF up vs PBS |
| Dhx15 | NM_007839 | NM_007839 // Dhx15 // DEAH (Asp-Glu-Ala-His) box polypeptide 15 // 5 C1 // 13204 | 2.42 | CNTF up vs PBS |
| Lmbrd1 | NM_026719 | NM_026719 // Lmbrd1 // LMBR1 domain containing 1 // 1 A5 // 68421 /// ENSMUST000 | 3.17 | CNTF up vs PBS |
| Cd84 | NM_013489 | NM_013489 // Cd84 // CD84 antigen // 1 H3|1 93.3 cM // 12523 /// ENSMUST00000042 | 7.27 | CNTF up vs PBS |
| Fam159b | NM_029984 | NM_029984 // Fam159b // family with sequence similarity 159, member B // 13 D1 / | -3.15 | CNTF down vs PBS |
| Fam105a | BC052328 | BC052328 // Fam105a // family with sequence similarity 105, member A // 15 B1 // | 4.71 | CNTF up vs PBS |
| Ero1l | NM_015774 | NM_015774 // Ero1l // ERO1-like (S. cerevisiae) // 14 C-D // 50527 /// ENSMUST00 | 3.12 | CNTF up vs PBS |
| Tmem68 | NM_028097 | NM_028097 // Tmem68 // transmembrane protein 68 // 4 A1 // 72098 /// NM_00111109 | 2.98 | CNTF up vs PBS |
| Gstm3 | NM_010359 | NM_010359 // Gstm3 // glutathione S-transferase, mu 3 // --- // 14864 /// ENSMUS | -2.05 | CNTF down vs PBS |
| Rbm39 | NM_133242 | NM_133242 // Rbm39 // RNA binding motif protein 39 // 2 H1 // 170791 /// ENSMUST | 2.99 | CNTF up vs PBS |
| Sdha | NM_023281 | NM_023281 // Sdha // succinate dehydrogenase complex, subunit A, flavoprotein (F | 2.25 | CNTF up vs PBS |
| Capg | NM_007599 | NM_007599 // Capg // capping protein (actin filament), gelsolin-like // 6 C3 // | 2.28 | CNTF up vs PBS |
| Hist1h1c | NM_015786 | NM_015786 // Hist1h1c // histone cluster 1, H1c // 13 A2-A3 // 50708 /// ENSMUST | 2.52 | CNTF up vs PBS |
| 6330403K07Rik | NM_134022 | NM_134022 // 6330403K07Rik // RIKEN cDNA 6330403K07 gene // 11 B4 // 103712 /// | 2.08 | CNTF up vs PBS |
| 4930579C15Rik | NM_027089 | NM_027089 // 4930579C15Rik // RIKEN cDNA 4930579C15 gene // 4 C5 // 67753 /// EN | -2.44 | CNTF down vs PBS |
| Gins4 | NM_024240 | NM_024240 // Gins4 // GINS complex subunit 4 (Sld5 homolog) // 8 A3 // 109145 // | 2.11 | CNTF up vs PBS |
| Yme1l1 | NM_013771 | NM_013771 // Yme1l1 // YME1-like 1 (S. cerevisiae) // 2 A3 // 27377 /// ENSMUST0 | 2.07 | CNTF up vs PBS |
| Ecm1 | NM_007899 | NM_007899 // Ecm1 // extracellular matrix protein 1 // 3 F2.1|3 45.4 cM // 13601 | 2.36 | CNTF up vs PBS |
| Hmgb3 | NM_008253 | NM_008253 // Hmgb3 // high mobility group box 3 // X A1 // 15354 /// ENSMUST0000 | -2.01 | CNTF down vs PBS |
| Pla2g1b | NM_011107 | NM_011107 // Pla2g1b // phospholipase A2, group IB, pancreas // 5 F1-G1.1 // 187 | -2.07 | CNTF down vs PBS |
| Cmtm7 | NM_133978 | NM_133978 // Cmtm7 // CKLF-like MARVEL transmembrane domain containing 7 // 9 F3 | 2.12 | CNTF up vs PBS |
| H2-Ab1 | NM_207105 | NM_207105 // H2-Ab1 // histocompatibility 2, class II antigen A, beta 1 // 17 B1 | 3.95 | CNTF up vs PBS |
| Shfm1 | NM_009169 | NM_009169 // Shfm1 // split hand/foot malformation (ectrodactyly) type 1 // 6 A2 | 2.22 | CNTF up vs PBS |
| Rassf2 | NM_175445 | NM_175445 // Rassf2 // Ras association (RalGDS/AF-6) domain family member 2 // 2 | 2.25 | CNTF up vs PBS |
| Dpy30 | NM_001146222 | NM_001146222 // Dpy30 // dpy-30 homolog (C. elegans) // 17 E2 // 66310 /// NM_00 | 3.70 | CNTF up vs PBS |
| Klhl28 | NM_025707 | NM_025707 // Klhl28 // kelch-like 28 (Drosophila) // 12 C1 // 66689 /// ENSMUST0 | 2.78 | CNTF up vs PBS |
| Sqstm1 | NM_011018 | NM_011018 // Sqstm1 // sequestosome 1 // 11 B1.2 // 18412 /// ENSMUST00000102774 | 2.16 | CNTF up vs PBS |
| Arhgap30 | NM_001005508 | NM_001005508 // Arhgap30 // Rho GTPase activating protein 30 // 1 H3 // 226652 / | 2.52 | CNTF up vs PBS |
| Ipp | NM_008389 | NM_008389 // Ipp // IAP promoted placental gene // 4 D1|4 51.4 cM // 16351 /// N | 2.02 | CNTF up vs PBS |
| Eid3 | NM_025499 | NM_025499 // Eid3 // EP300 interacting inhibitor of differentiation 3 // 10 C1 / | 3.56 | CNTF up vs PBS |
| Itm2b | NM_008410 | NM_008410 // Itm2b // integral membrane protein 2B // 14 D3|14 32.5 cM // 16432 | 2.22 | CNTF up vs PBS |
| Psmb9 | NM_013585 | NM_013585 // Psmb9 // proteasome (prosome, macropain) subunit, beta type 9 (larg | 5.64 | CNTF up vs PBS |
| Chmp5 | NM_029814 | NM_029814 // Chmp5 // chromatin modifying protein 5 // 4 A5 // 76959 /// ENSMUST | 2.06 | CNTF up vs PBS |
| Rell2 | NM_153793 | NM_153793 // Rell2 // RELT-like 2 // 18 B3 // 225392 /// NM_175684 // Fchsd1 // | -2.07 | CNTF down vs PBS |
| Aoah | NM_012054 | NM_012054 // Aoah // acyloxyacyl hydrolase // 13 A2 // 27052 /// ENSMUST00000021 | 4.70 | CNTF up vs PBS |
| Fkbpl | NM_019873 | NM_019873 // Fkbpl // FK506 binding protein-like // 17 B1 // 56299 /// ENSMUST00 | -2.21 | CNTF down vs PBS |
| Lin52 | NM_173756 | NM_173756 // Lin52 // lin-52 homolog (C. elegans) // 12 D1 // 217708 /// BC12049 | 2.15 | CNTF up vs PBS |
| Gnai2 | NM_008138 | NM_008138 // Gnai2 // guanine nucleotide binding protein (G protein), alpha inhi | 2.46 | CNTF up vs PBS |
| 15-Sep | NM_053102 | NM_053102 // Sep15 // selenoprotein // 3 H3 // 93684 /// ENSMUST00000082437 // S | 2.28 | CNTF up vs PBS |
| 0610009D07Rik | NM_025323 | NM_025323 // 0610009D07Rik // RIKEN cDNA 0610009D07 gene // 12 A1.1 // 66055 /// | 2.15 | CNTF up vs PBS |
| Gng2 | NM_010315 | NM_010315 // Gng2 // guanine nucleotide binding protein (G protein), gamma 2 // | 2.27 | CNTF up vs PBS |
| Ms4a7 | NM_027836 | NM_027836 // Ms4a7 // membrane-spanning 4-domains, subfamily A, member 7 // 19 A | 4.47 | CNTF up vs PBS |
| Adam8 | NM_007403 | NM_007403 // Adam8 // a disintegrin and metallopeptidase domain 8 // 7 F3-F5 // | 2.04 | CNTF up vs PBS |
| Lmbrd1 | NM_026719 | NM_026719 // Lmbrd1 // LMBR1 domain containing 1 // 1 A5 // 68421 /// ENSMUST000 | 2.20 | CNTF up vs PBS |
| Slc40a1 | NM_016917 | NM_016917 // Slc40a1 // solute carrier family 40 (iron-regulated transporter), m | 3.11 | CNTF up vs PBS |
| Arl9 | NM_206935 | NM_206935 // Arl9 // ADP-ribosylation factor-like 9 // 5 C3.3 // 384185 /// ENSM | -2.00 | CNTF down vs PBS |
| Elavl1 | NM_010485 | NM_010485 // Elavl1 // ELAV (embryonic lethal, abnormal vision, Drosophila)-like | 2.21 | CNTF up vs PBS |
| Tmx1 | NM_028339 | NM_028339 // Tmx1 // thioredoxin-related transmembrane protein 1 // 12 C2 // 727 | 2.47 | CNTF up vs PBS |
| Apbb1ip | NM_019456 | NM_019456 // Apbb1ip // amyloid beta (A4) precursor protein-binding, family B, m | 4.03 | CNTF up vs PBS |
| Casp4 | NM_007609 | NM_007609 // Casp4 // caspase 4, apoptosis-related cysteine peptidase // 9 A1|9 | 3.97 | CNTF up vs PBS |
| 7-Sep | NM_009859 | NM_009859 // Sept7 // septin 7 // 9 A4 // 235072 /// ENSMUST00000115272 // Sept7 | 2.07 | CNTF up vs PBS |
| Hcls1 | NM_008225 | NM_008225 // Hcls1 // hematopoietic cell specific Lyn substrate 1 // 16 B // 151 | 2.77 | CNTF up vs PBS |
| Cep350 | BC089561 | BC089561 // Cep350 // centrosomal protein 350 // 1 G3 // 74081 /// AK172956 // C | -2.78 | CNTF down vs PBS |
| Snap25 | NM_011428 | NM_011428 // Snap25 // synaptosomal-associated protein 25 // 2 F3|2 78.2 cM // 2 | 2.44 | CNTF up vs PBS |
| Fbxo28 | NM_175127 | NM_175127 // Fbxo28 // F-box protein 28 // 1 H5|1 98.7 cM // 67948 /// ENSMUST00 | 2.45 | CNTF up vs PBS |
| Pcyt1a | NM_009981 | NM_009981 // Pcyt1a // phosphate cytidylyltransferase 1, choline, alpha isoform | 2.35 | CNTF up vs PBS |
| Metap2 | NM_019648 | NM_019648 // Metap2 // methionine aminopeptidase 2 // 10 C2 // 56307 /// NM_0168 | 2.53 | CNTF up vs PBS |
| Nat13 | NM_028108 | NM_028108 // Nat13 // N-acetyltransferase 13 // 16 B4 // 72117 /// ENSMUST000000 | 3.01 | CNTF up vs PBS |
| Ppp2r1a | NM_016891 | NM_016891 // Ppp2r1a // protein phosphatase 2 (formerly 2A), regulatory subunit | 2.59 | CNTF up vs PBS |
| Lnpep | NM_172827 | NM_172827 // Lnpep // leucyl/cystinyl aminopeptidase // 17 A3.2 // 240028 /// EN | 2.46 | CNTF up vs PBS |
| Ifnk | NM_199157 | NM_199157 // Ifnk // interferon kappa // 4 A5 // 387510 /// ENSMUST00000058595 / | -2.16 | CNTF down vs PBS |
| 9330133O14Rik | ENSMUST00000059529 | ENSMUST00000059529 // 9330133O14Rik // RIKEN cDNA 9330133O14 gene // 8 E1 // 319 | -2.34 | CNTF down vs PBS |
| Ccdc56 | NM_026618 | NM_026618 // Ccdc56 // coiled-coil domain containing 56 // 11 D|11 60.0 cM // 52 | 2.07 | CNTF up vs PBS |
| Slc25a17 | NM_011399 | NM_011399 // Slc25a17 // solute carrier family 25 (mitochondrial carrier, peroxi | 2.55 | CNTF up vs PBS |
| Sec11a | NM_019951 | NM_019951 // Sec11a // SEC11 homolog A (S. cerevisiae) // 7 D3 // 56529 /// ENSM | 2.43 | CNTF up vs PBS |
| Clec4a3 | NM_153197 | NM_153197 // Clec4a3 // C-type lectin domain family 4, member a3 // 6 F2 // 7314 | 6.54 | CNTF up vs PBS |
| Defb19 | NM_145157 | NM_145157 // Defb19 // defensin beta 19 // 2 H1 // 246700 /// ENSMUST00000053180 | -2.61 | CNTF down vs PBS |
| Atad2 | NM_027435 | NM_027435 // Atad2 // ATPase family, AAA domain containing 2 // 15 D2 // 70472 / | 2.10 | CNTF up vs PBS |
| Selm | NM_053267 | NM_053267 // Selm // selenoprotein M // --- // 114679 /// ENSMUST00000094469 // | -3.55 | CNTF down vs PBS |
| Ric3 | NM_001038624 | NM_001038624 // Ric3 // resistance to inhibitors of cholinesterase 3 homolog (C. | -2.01 | CNTF down vs PBS |
| Lyrm4 | NM_201358 | NM_201358 // Lyrm4 // LYR motif containing 4 // 13 A3.3 // 380840 /// ENSMUST000 | 4.03 | CNTF up vs PBS |
| Lgals3bp | NM_011150 | NM_011150 // Lgals3bp // lectin, galactoside-binding, soluble, 3 binding protein | 2.82 | CNTF up vs PBS |
| Uba1 | NM_009457 | NM_009457 // Uba1 // ubiquitin-like modifier activating enzyme 1 // X A2-A3|X 5. | 2.06 | CNTF up vs PBS |
| Tmem50a | NM_027935 | NM_027935 // Tmem50a // transmembrane protein 50A // 4 D3|4 65.71 cM // 71817 // | 3.11 | CNTF up vs PBS |
| Yipf5 | NM_023311 | NM_023311 // Yipf5 // Yip1 domain family, member 5 // 18 B3 // 67180 /// ENSMUST | 2.67 | CNTF up vs PBS |
| 5133401N09Rik | NM_198004 | NM_198004 // 5133401N09Rik // RIKEN cDNA 5133401N09 gene // 13 B1 // 75731 /// N | 2.55 | CNTF up vs PBS |
| Cct8 | NM_009840 | NM_009840 // Cct8 // chaperonin containing Tcp1, subunit 8 (theta) // 16 C3.3 // | 2.57 | CNTF up vs PBS |
| Fh1 | NM_010209 | NM_010209 // Fh1 // fumarate hydratase 1 // 1 H4 // 14194 /// ENSMUST00000027810 | 2.24 | CNTF up vs PBS |
| Tbcc | NM_178385 | NM_178385 // Tbcc // tubulin-specific chaperone C // 17 C // 72726 /// ENSMUST00 | -2.59 | CNTF down vs PBS |
| Ipo5 | NM_023579 | NM_023579 // Ipo5 // importin 5 // 14 E5 // 70572 /// ENSMUST00000032898 // Ipo5 | 2.52 | CNTF up vs PBS |
| Sirpb1 | NM_001002898 | NM_001002898 // Sirpb1 // signal-regulatory protein beta 1 // 3 A1 // 320832 /// | 4.64 | CNTF up vs PBS |
| Fbxo25 | NM_025785 | NM_025785 // Fbxo25 // F-box protein 25 // 8 A1.1 // 66822 /// ENSMUST0000004352 | 2.11 | CNTF up vs PBS |
| Cd180 | NM_008533 | NM_008533 // Cd180 // CD180 antigen // 13 D1 // 17079 /// ENSMUST00000022124 // | 8.76 | CNTF up vs PBS |
| Nr4a2 | NM_001139509 | NM_001139509 // Nr4a2 // nuclear receptor subfamily 4, group A, member 2 // 2 C1 | 2.49 | CNTF up vs PBS |
| Gprc5a | NM_181444 | NM_181444 // Gprc5a // G protein-coupled receptor, family C, group 5, member A / | -2.17 | CNTF down vs PBS |
| Olfr1370 | NM_146535 | NM_146535 // Olfr1370 // olfactory receptor 1370 // --- // 258528 /// ENSMUST000 | -2.34 | CNTF down vs PBS |
| Ccl12 | NM_011331 | NM_011331 // Ccl12 // chemokine (C-C motif) ligand 12 // 11 C|11 47.0 cM // 2029 | 3.52 | CNTF up vs PBS |
| Arcn1 | NM_145985 | NM_145985 // Arcn1 // archain 1 // 9 A5.2 // 213827 /// ENSMUST00000034607 // Ar | 2.48 | CNTF up vs PBS |
| V1rb4 | NM_053227 | NM_053227 // V1rb4 // vomeronasal 1 receptor, B4 // 6 D1 // 113854 /// ENSMUST00 | -2.60 | CNTF down vs PBS |
| Il13ra1 | NM_133990 | NM_133990 // Il13ra1 // interleukin 13 receptor, alpha 1 // X A3.3|X 12.5 cM // | 3.43 | CNTF up vs PBS |
| Agtr1a | NM_177322 | NM_177322 // Agtr1a // angiotensin II receptor, type 1a // 13 A3.2|13 16.0 cM // | -2.00 | CNTF down vs PBS |
| Tlr6 | NM_011604 | NM_011604 // Tlr6 // toll-like receptor 6 // 5 C3.1|5 37.0 cM // 21899 /// NM_03 | 2.14 | CNTF up vs PBS |
| Blmh | NM_178645 | NM_178645 // Blmh // bleomycin hydrolase // 11 B5 // 104184 /// ENSMUST000000211 | 2.25 | CNTF up vs PBS |
| Tpsb2 | X78542 | X78542 // Tpsb2 // tryptase beta 2 // 17 A3.3|17 10.39 cM // 17229 /// ENSMUST00 | -2.89 | CNTF down vs PBS |
| Sostdc1 | NM_025312 | NM_025312 // Sostdc1 // sclerostin domain containing 1 // 12 B2 // 66042 /// ENS | -2.15 | CNTF down vs PBS |
| Tas2r117 | NM_207021 | NM_207021 // Tas2r117 // taste receptor, type 2, member 117 // 6 G1|6 63.5 cM // | -2.67 | CNTF down vs PBS |
| Gsr | NM_010344 | NM_010344 // Gsr // glutathione reductase // 8 A4|8 18.0 cM // 14782 /// ENSMUST | 2.24 | CNTF up vs PBS |
| Impact | NM_008378 | NM_008378 // Impact // imprinted and ancient // 18 A2-B2 // 16210 /// ENSMUST000 | 2.78 | CNTF up vs PBS |
| Renbp | NM_023132 | NM_023132 // Renbp // renin binding protein // X A7.3|X 29.53 cM // 19703 /// EN | 2.40 | CNTF up vs PBS |
| Pon2 | NM_183308 | NM_183308 // Pon2 // paraoxonase 2 // 6 A1 // 330260 /// ENSMUST00000057792 // P | 2.05 | CNTF up vs PBS |
| Bmp2k | NM_080708 | NM_080708 // Bmp2k // BMP2 inducible kinase // 5 E3 // 140780 /// ENSMUST0000003 | 2.42 | CNTF up vs PBS |
| Cd86 | NM_019388 | NM_019388 // Cd86 // CD86 antigen // 16 B5|16 26.9 cM // 12524 /// ENSMUST000000 | 4.26 | CNTF up vs PBS |
| Cd52 | NM_013706 | NM_013706 // Cd52 // CD52 antigen // 4 D3|4 73.5 cM // 23833 /// ENSMUST00000000 | 6.65 | CNTF up vs PBS |
| Zdhhc13 | NM_028031 | NM_028031 // Zdhhc13 // zinc finger, DHHC domain containing 13 // 7 B4 // 243983 | 2.06 | CNTF up vs PBS |
| Gm3604 | NM_001162910 | NM_001162910 // Gm3604 // predicted gene 3604 // 13 B3|13 // 100041979 /// NM_17 | -9.95 | CNTF down vs PBS |
| Olfr558 | NM_147093 | NM_147093 // Olfr558 // olfactory receptor 558 // --- // 259097 /// ENSMUST00000 | -2.37 | CNTF down vs PBS |
| B830004H01Rik | AK046779 | AK046779 // B830004H01Rik // RIKEN cDNA B830004H01 gene // 4 E1 // 320289 /// AK | -2.93 | CNTF down vs PBS |
| Hmgb1 | NM_010439 | NM_010439 // Hmgb1 // high mobility group box 1 // 5 G3|5 83.0 cM // 15289 /// B | 12.98 | CNTF up vs PBS |
| Hspa4l | NM_011020 | NM_011020 // Hspa4l // heat shock protein 4 like // 3 B // 18415 /// ENSMUST0000 | 2.36 | CNTF up vs PBS |
| Gm3604 | NM_001162910 | NM_001162910 // Gm3604 // predicted gene 3604 // 13 B3|13 // 100041979 /// NM_17 | -2.05 | CNTF down vs PBS |
| Tardbp | NM_145556 | NM_145556 // Tardbp // TAR DNA binding protein // 4 E2 // 230908 /// NM_00100854 | 2.81 | CNTF up vs PBS |
| Ptplad2 | NM_025760 | NM_025760 // Ptplad2 // protein tyrosine phosphatase-like A domain containing 2 | 2.57 | CNTF up vs PBS |
| Pcdhb18 | NM_053143 | NM_053143 // Pcdhb18 // protocadherin beta 18 // 18 B3 // 93889 /// ENSMUST00000 | 2.10 | CNTF up vs PBS |
| Ccl6 | NM_009139 | NM_009139 // Ccl6 // chemokine (C-C motif) ligand 6 // 11 C|11 47.51 cM // 20305 | 3.46 | CNTF up vs PBS |
| Rhog | NM_019566 | NM_019566 // Rhog // ras homolog gene family, member G // 7 F1 // 56212 /// ENSM | 2.11 | CNTF up vs PBS |
| Ccdc142 | NM_001081266 | NM_001081266 // Ccdc142 // coiled-coil domain containing 142 // 6 C3 // 243510 / | -2.12 | CNTF down vs PBS |
| Usp9x | NM_009481 | NM_009481 // Usp9x // ubiquitin specific peptidase 9, X chromosome // X A1.1|X 5 | 2.12 | CNTF up vs PBS |
| Prdx3 | NM_007452 | NM_007452 // Prdx3 // peroxiredoxin 3 // 19 D3|19 50.0 cM // 11757 /// ENSMUST00 | 3.38 | CNTF up vs PBS |
| Bst2 | NM_198095 | NM_198095 // Bst2 // bone marrow stromal cell antigen 2 // 8 B3.3 // 69550 /// E | 2.34 | CNTF up vs PBS |
| Disp1 | NM_026866 | NM_026866 // Disp1 // dispatched homolog 1 (Drosophila) // 1|1 101.2 cM // 68897 | -3.92 | CNTF down vs PBS |
| Abhd2 | NM_018811 | NM_018811 // Abhd2 // abhydrolase domain containing 2 // 7 D2 // 54608 /// ENSMU | 2.28 | CNTF up vs PBS |
| Pcdhb12 | NM_053137 | NM_053137 // Pcdhb12 // protocadherin beta 12 // 18 B3 // 93883 /// ENSMUST00000 | -2.84 | CNTF down vs PBS |
| Frat1 | NM_008043 | NM_008043 // Frat1 // frequently rearranged in advanced T-cell lymphomas // 19 C | -2.01 | CNTF down vs PBS |
| Cyth4 | NM_028195 | NM_028195 // Cyth4 // cytohesin 4 // 15 E1 // 72318 /// ENSMUST00000043069 // Cy | 3.25 | CNTF up vs PBS |
| P4ha1 | NM_011030 | NM_011030 // P4ha1 // procollagen-proline, 2-oxoglutarate 4-dioxygenase (proline | 2.40 | CNTF up vs PBS |
| Epha2 | BC006954 | BC006954 // Epha2 // Eph receptor A2 // 4 D-E|4 73.2 cM // 13836 | -2.41 | CNTF down vs PBS |
| Hist3h2a | NM_178218 | NM_178218 // Hist3h2a // histone cluster 3, H2a // --- // 319162 /// ENSMUST0000 | 2.06 | CNTF up vs PBS |
| Olfr1106 | NM_146752 | NM_146752 // Olfr1106 // olfactory receptor 1106 // --- // 258747 /// ENSMUST000 | -2.04 | CNTF down vs PBS |
| P2rx4 | NM_011026 | NM_011026 // P2rx4 // purinergic receptor P2X, ligand-gated ion channel 4 // 5 F | 2.64 | CNTF up vs PBS |
| Picalm | NM_146194 | NM_146194 // Picalm // phosphatidylinositol binding clathrin assembly protein // | 3.79 | CNTF up vs PBS |
| 5330431K02Rik | AK019926 | AK019926 // 5330431K02Rik // RIKEN cDNA 5330431K02 gene // 13 D1 // 68189 | -2.37 | CNTF down vs PBS |
| Cypt2 | NM_173436 | NM_173436 // Cypt2 // cysteine-rich perinuclear theca 2 // X D // 245566 /// ENS | -2.08 | CNTF down vs PBS |
| Got1 | NM_010324 | NM_010324 // Got1 // glutamate oxaloacetate transaminase 1, soluble // 19 C3|19 | 2.65 | CNTF up vs PBS |
| Dbil5 | NM_021294 | NM_021294 // Dbil5 // diazepam binding inhibitor-like 5 // 11 B5 // 13168 /// EN | -2.35 | CNTF down vs PBS |
| Cdc42se2 | NM_178626 | NM_178626 // Cdc42se2 // CDC42 small effector 2 // 11 B1.3 // 72729 /// ENSMUST0 | 2.37 | CNTF up vs PBS |
| Olfr672 | NM_146760 | NM_146760 // Olfr672 // olfactory receptor 672 // --- // 258755 /// ENSMUST00000 | -2.68 | CNTF down vs PBS |
| Sec61a1 | NM_016906 | NM_016906 // Sec61a1 // Sec61 alpha 1 subunit (S. cerevisiae) // 6 D1 // 53421 / | 2.37 | CNTF up vs PBS |
| Vti1b | NM_016800 | NM_016800 // Vti1b // vesicle transport through interaction with t-SNAREs 1B hom | 2.55 | CNTF up vs PBS |
| Olfr552 | NM_147102 | NM_147102 // Olfr552 // olfactory receptor 552 // --- // 259106 /// ENSMUST00000 | -2.16 | CNTF down vs PBS |
| Slc35a3 | NM_144902 | NM_144902 // Slc35a3 // solute carrier family 35 (UDP-N-acetylglucosamine (UDP-G | 2.23 | CNTF up vs PBS |
| Dock8 | NM_028785 | NM_028785 // Dock8 // dedicator of cytokinesis 8 // 19 B // 76088 /// ENSMUST000 | 2.67 | CNTF up vs PBS |
| Atp6v0a1 | NM_016920 | NM_016920 // Atp6v0a1 // ATPase, H+ transporting, lysosomal V0 subunit A1 // 11 | 2.15 | CNTF up vs PBS |
| Plcg2 | NM_172285 | NM_172285 // Plcg2 // phospholipase C, gamma 2 // 8 E1 // 234779 /// ENSMUST0000 | 2.18 | CNTF up vs PBS |
| Ebpl | NM_026598 | NM_026598 // Ebpl // emopamil binding protein-like // 14 D1 // 68177 /// ENSMUST | 2.27 | CNTF up vs PBS |
| Cnot1 | NM_153164 | NM_153164 // Cnot1 // CCR4-NOT transcription complex, subunit 1 // 8 D1 // 23459 | 2.16 | CNTF up vs PBS |
| Usp25 | NM_013918 | NM_013918 // Usp25 // ubiquitin specific peptidase 25 // --- // 30940 /// ENSMUS | 2.23 | CNTF up vs PBS |
| Creb1 | NM_133828 | NM_133828 // Creb1 // cAMP responsive element binding protein 1 // 1 C2|1 31.0 c | 3.86 | CNTF up vs PBS |
| Prdx1 | NM_011034 | NM_011034 // Prdx1 // peroxiredoxin 1 // 4 D1|4 47.0 cM // 18477 /// ENSMUST0000 | 2.46 | CNTF up vs PBS |
| Mospd1 | NM_027409 | NM_027409 // Mospd1 // motile sperm domain containing 1 // X A4 // 70380 /// ENS | 4.24 | CNTF up vs PBS |
| Cryzl1 | NM_133679 | NM_133679 // Cryzl1 // crystallin, zeta (quinone reductase)-like 1 // 16 C4 // 6 | 2.53 | CNTF up vs PBS |
| Zufsp | NM_028287 | NM_028287 // Zufsp // zinc finger with UFM1-specific peptidase domain // 10 B1 / | 2.24 | CNTF up vs PBS |
| Zfp36 | NM_011756 | NM_011756 // Zfp36 // zinc finger protein 36 // 7 A3|7 10.2 cM // 22695 /// ENSM | 2.09 | CNTF up vs PBS |
| Clec3b | NM_011606 | NM_011606 // Clec3b // C-type lectin domain family 3, member b // 9 F1-F3|9 71.0 | -2.07 | CNTF down vs PBS |
| Gpr151 | NM_181543 | NM_181543 // Gpr151 // G protein-coupled receptor 151 // 18 B3 // 240239 /// ENS | -2.10 | CNTF down vs PBS |
| Stab1 | NM_138672 | NM_138672 // Stab1 // stabilin 1 // 14 B // 192187 /// ENSMUST00000036618 // Sta | 2.10 | CNTF up vs PBS |
| Olfr303 | NM_146619 | NM_146619 // Olfr303 // olfactory receptor 303 // --- // 258612 /// ENSMUST00000 | -2.57 | CNTF down vs PBS |
| BC031181 | BC016084 | BC016084 // BC031181 // cDNA sequence BC031181 // 18 E3 // 407819 /// ENSMUST000 | 2.33 | CNTF up vs PBS |
| B330016D10Rik | AK046532 | AK046532 // B330016D10Rik // RIKEN cDNA B330016D10 gene // 4 E1 // 320456 /// XR | -3.16 | CNTF down vs PBS |
| BC051142 | BC051142 | BC051142 // BC051142 // cDNA sequence BC051142 // 17 B1 // 407788 /// NM_0010011 | -2.54 | CNTF down vs PBS |
| Atp2c1 | NM_175025 | NM_175025 // Atp2c1 // ATPase, Ca++-sequestering // 9 F1 // 235574 /// ENSMUST00 | 2.24 | CNTF up vs PBS |
| Yipf6 | NM_207633 | NM_207633 // Yipf6 // Yip1 domain family, member 6 // X C2 // 77929 /// BC048712 | 2.47 | CNTF up vs PBS |
| Art2a | NM_007490 | NM_007490 // Art2a // ADP-ribosyltransferase 2a // 7 E3|7 49.0 cM // 11871 /// N | 4.75 | CNTF up vs PBS |
| Zfp536 | NM_172385 | NM_172385 // Zfp536 // zinc finger protein 536 // 7 B2 // 243937 /// ENSMUST0000 | -2.07 | CNTF down vs PBS |
| Chmp1b | NM_024190 | NM_024190 // Chmp1b // chromatin modifying protein 1B // 18 E1 // 67064 /// BC00 | 2.08 | CNTF up vs PBS |
| Arf2 | NM_007477 | NM_007477 // Arf2 // ADP-ribosylation factor 2 // 11 E1|11 62.0 cM // 11841 /// | 2.96 | CNTF up vs PBS |
| Atp8a1 | NM_001038999 | NM_001038999 // Atp8a1 // ATPase, aminophospholipid transporter (APLT), class I, | 2.09 | CNTF up vs PBS |
| Dad1 | NM_010015 | NM_010015 // Dad1 // defender against cell death 1 // 14 C2|14 24.0 cM // 13135 | 3.84 | CNTF up vs PBS |
| Sqle | NM_009270 | NM_009270 // Sqle // squalene epoxidase // 15 D1 // 20775 /// ENSMUST00000022977 | 2.14 | CNTF up vs PBS |
| Arrb2 | NM_145429 | NM_145429 // Arrb2 // arrestin, beta 2 // 11 B3|11 45.0 cM // 216869 /// ENSMUST | 2.61 | CNTF up vs PBS |
| Mrps18c | NM_026826 | NM_026826 // Mrps18c // mitochondrial ribosomal protein S18C // 5 E3 // 68735 // | 9.30 | CNTF up vs PBS |
| Tst | NM_009437 | NM_009437 // Tst // thiosulfate sulfurtransferase, mitochondrial // 15 E1|15 45. | -2.40 | CNTF down vs PBS |
| Csdc2 | NM_145473 | NM_145473 // Csdc2 // cold shock domain containing C2, RNA binding // 15 E1 // 1 | -2.20 | CNTF down vs PBS |
| Eif4a2 | NM_001123038 | NM_001123038 // Eif4a2 // eukaryotic translation initiation factor 4A2 // 16 B1| | 5.45 | CNTF up vs PBS |
| Evi2b | NM_146023 | NM_146023 // Evi2b // ecotropic viral integration site 2b // 11 B5 // 216984 /// | 2.23 | CNTF up vs PBS |
| Nhsl1 | NM_173390 | NM_173390 // Nhsl1 // NHS-like 1 // 10 A3|10 8.0 cM // 215819 /// ENSMUST0000003 | -2.01 | CNTF down vs PBS |
| Chst9 | NM_199055 | NM_199055 // Chst9 // carbohydrate (N-acetylgalactosamine 4-0) sulfotransferase | -2.44 | CNTF down vs PBS |
| Gm5124 | NM_198633 | NM_198633 // Gm5124 // predicted gene 5124 // X A2|X // 331392 /// ENSMUST000000 | -2.05 | CNTF down vs PBS |
| Frs2 | NM_177798 | NM_177798 // Frs2 // fibroblast growth factor receptor substrate 2 // 10 D2 // 3 | 2.30 | CNTF up vs PBS |
| Olfr1298 | NM_146886 | NM_146886 // Olfr1298 // olfactory receptor 1298 // --- // 258888 /// ENSMUST000 | -2.09 | CNTF down vs PBS |
| AI314976 | BC022574 | BC022574 // AI314976 // expressed sequence AI314976 // 17 C // 106821 /// NM_207 | 2.61 | CNTF up vs PBS |
| Creg1 | NM_011804 | NM_011804 // Creg1 // cellular repressor of E1A-stimulated genes 1 // 1 H2.3 // | 4.35 | CNTF up vs PBS |
| Gsto1 | NM_010362 | NM_010362 // Gsto1 // glutathione S-transferase omega 1 // 19 D1 // 14873 /// EN | 2.52 | CNTF up vs PBS |
| Serpina3n | NM_009252 | NM_009252 // Serpina3n // serine (or cysteine) peptidase inhibitor, clade A, mem | 4.17 | CNTF up vs PBS |
| Elmo1 | NM_080288 | NM_080288 // Elmo1 // engulfment and cell motility 1, ced-12 homolog (C. elegans | 2.46 | CNTF up vs PBS |
| Hfe | NM_010424 | NM_010424 // Hfe // hemochromatosis // 13 A2-A4|13 15.0 cM // 15216 /// ENSMUST0 | 2.19 | CNTF up vs PBS |
| Psmd14 | NM_021526 | NM_021526 // Psmd14 // proteasome (prosome, macropain) 26S subunit, non-ATPase, | 2.07 | CNTF up vs PBS |
| Fam76a | BC008163 | BC008163 // Fam76a // family with sequence similarity 76, member A // 4 D2.3 // | 3.00 | CNTF up vs PBS |
| V1rb3 | NM_053226 | NM_053226 // V1rb3 // vomeronasal 1 receptor, B3 // 6 D1 // 113853 /// ENSMUST00 | -3.02 | CNTF down vs PBS |
| V1rc27 | NM_134182 | NM_134182 // V1rc27 // vomeronasal 1 receptor, C27 // 6 B3 // 171200 /// ENSMUST | -2.46 | CNTF down vs PBS |
| Olfr736 | NM_146666 | NM_146666 // Olfr736 // olfactory receptor 736 // --- // 258660 /// ENSMUST00000 | -2.19 | CNTF down vs PBS |
| Pcdhb15 | NM_053140 | NM_053140 // Pcdhb15 // protocadherin beta 15 // 18 B3 // 93886 /// ENSMUST00000 | -2.59 | CNTF down vs PBS |
| Slc38a9 | NM_178746 | NM_178746 // Slc38a9 // solute carrier family 38, member 9 // 13 D2.2 // 268706 | 2.03 | CNTF up vs PBS |
| Lgals3 | NM_001145953 | NM_001145953 // Lgals3 // lectin, galactose binding, soluble 3 // 14 C1 // 16854 | 2.83 | CNTF up vs PBS |
| Clic4 | NM_013885 | NM_013885 // Clic4 // chloride intracellular channel 4 (mitochondrial) // 4 D3 / | 2.02 | CNTF up vs PBS |
| Aga | NM_001005847 | NM_001005847 // Aga // aspartylglucosaminidase // 8 B3 // 11593 /// ENSMUST00000 | 2.57 | CNTF up vs PBS |
| Cnbp | NM_013493 | NM_013493 // Cnbp // cellular nucleic acid binding protein // 6 D1-D2 // 12785 / | 2.02 | CNTF up vs PBS |
| Gnmt | NM_010321 | NM_010321 // Gnmt // glycine N-methyltransferase // 17 C|17 10.0 cM // 14711 /// | -2.00 | CNTF down vs PBS |
| Cd81 | NM_133655 | NM_133655 // Cd81 // CD81 antigen // 7 F5|7 69.3 cM // 12520 /// ENSMUST00000037 | 2.45 | CNTF up vs PBS |
| Speer4b | NM_028561 | NM_028561 // Speer4b // spermatogenesis associated glutamate (E)-rich protein 4b | -2.76 | CNTF down vs PBS |
| Lsm8 | NM_133939 | NM_133939 // Lsm8 // LSM8 homolog, U6 small nuclear RNA associated (S. cerevisia | 2.52 | CNTF up vs PBS |
| Il1a | NM_010554 | NM_010554 // Il1a // interleukin 1 alpha // 2 F|2 73.0 cM // 16175 /// NM_007619 | 8.28 | CNTF up vs PBS |
| Plbd1 | NM_025806 | NM_025806 // Plbd1 // phospholipase B domain containing 1 // 6 G1 // 66857 /// E | 5.04 | CNTF up vs PBS |
| Il17rd | NM_134437 | NM_134437 // Il17rd // interleukin 17 receptor D // 14 A3 // 171463 /// ENSMUST0 | -2.21 | CNTF down vs PBS |
| Vav1 | NM_011691 | NM_011691 // Vav1 // vav 1 oncogene // 17 D|17 32.7 cM // 22324 /// ENSMUST00000 | 2.51 | CNTF up vs PBS |
| Olfr902 | ENSMUST00000050733 | ENSMUST00000050733 // Olfr902 // olfactory receptor 902 // --- // 258798 | -2.32 | CNTF down vs PBS |
| Cpeb4 | NM_026252 | NM_026252 // Cpeb4 // cytoplasmic polyadenylation element binding protein 4 // 1 | 2.08 | CNTF up vs PBS |
| 1110003E01Rik | BC009097 | BC009097 // 1110003E01Rik // RIKEN cDNA 1110003E01 gene // 5 C3.1 // 68552 /// N | 2.42 | CNTF up vs PBS |
| Emb | NM_010330 | NM_010330 // Emb // embigin // 13 D2.3 // 13723 /// NM_011279 // Rnf7 // ring fi | 2.50 | CNTF up vs PBS |
| Esf1 | NM_001081090 | NM_001081090 // Esf1 // ESF1, nucleolar pre-rRNA processing protein, homolog (S. | 2.21 | CNTF up vs PBS |
| AF251705 | NM_134158 | NM_134158 // AF251705 // cDNA sequence AF251705 // 11 E2|11 78.0 cM // 140497 // | 2.98 | CNTF up vs PBS |
| Hmox1 | NM_010442 | NM_010442 // Hmox1 // heme oxygenase (decycling) 1 // 8 C1|8 35.0 cM // 15368 // | 8.89 | CNTF up vs PBS |
| Tnks | BC094602 | BC094602 // Tnks // tankyrase, TRF1-interacting ankyrin-related ADP-ribose polym | 4.06 | CNTF up vs PBS |
| Agtpbp1 | NM_023328 | NM_023328 // Agtpbp1 // ATP/GTP binding protein 1 // 13 B2 // 67269 /// NM_00104 | 3.46 | CNTF up vs PBS |
| Jmjd1c | NM_207221 | NM_207221 // Jmjd1c // jumonji domain containing 1C // 10 B5.1 // 108829 /// ENS | 2.15 | CNTF up vs PBS |
| Fzd3 | NM_021458 | NM_021458 // Fzd3 // frizzled homolog 3 (Drosophila) // 14 D1|14 27.0 cM // 1436 | 3.28 | CNTF up vs PBS |
| Cd48 | NM_007649 | NM_007649 // Cd48 // CD48 antigen // 1 H3|1 93.3 cM // 12506 /// ENSMUST00000068 | 5.17 | CNTF up vs PBS |
| Casd1 | NM_145398 | NM_145398 // Casd1 // CAS1 domain containing 1 // 6 A1|6 0.5 cM // 213819 /// EN | 2.25 | CNTF up vs PBS |
| Cebpg | NM_009884 | NM_009884 // Cebpg // CCAAT/enhancer binding protein (C/EBP), gamma // 7 B1 // 1 | 4.36 | CNTF up vs PBS |
| Slc15a3 | NM_023044 | NM_023044 // Slc15a3 // solute carrier family 15, member 3 // 19 B // 65221 /// | 3.11 | CNTF up vs PBS |
| Ube2v2 | NM_023585 | NM_023585 // Ube2v2 // ubiquitin-conjugating enzyme E2 variant 2 // 16 A1 // 706 | 4.23 | CNTF up vs PBS |
| Mocs2 | NM_013826 | NM_013826 // Mocs2 // molybdenum cofactor synthesis 2 // 13 D2.2 // 17434 /// NM | 2.22 | CNTF up vs PBS |
| 1810074P20Rik | NM_026194 | NM_026194 // 1810074P20Rik // RIKEN cDNA 1810074P20 gene // 4 A3 // 67490 /// EN | 2.24 | CNTF up vs PBS |
| Max | NM_008558 | NM_008558 // Max // Max protein // 12 D1-D3|12 32.0 cM // 17187 /// NM_001146176 | 2.11 | CNTF up vs PBS |
| Acyp1 | NM_025421 | NM_025421 // Acyp1 // acylphosphatase 1, erythrocyte (common) type // 12 D2 // 6 | 2.96 | CNTF up vs PBS |
| Mbnl2 | NM_175341 | NM_175341 // Mbnl2 // muscleblind-like 2 // 14 E4 // 105559 /// NM_207515 // Mbn | 2.32 | CNTF up vs PBS |
| Tmsb4x | NM_021278 | NM_021278 // Tmsb4x // thymosin, beta 4, X chromosome // X F5|X 72.5 cM // 19241 | 2.68 | CNTF up vs PBS |
| BC018507 | BC026613 | BC026613 // BC018507 // cDNA sequence BC018507 // 13 C1 // 218333 /// BC018507 / | 2.19 | CNTF up vs PBS |
| Olfr619 | NM_147076 | NM_147076 // Olfr619 // olfactory receptor 619 // --- // 259080 /// ENSMUST00000 | -3.52 | CNTF down vs PBS |
| 2310028O11Rik | NM_001163006 | NM_001163006 // 2310028O11Rik // RIKEN cDNA 2310028O11 gene // 4 D3 // 433771 // | 5.36 | CNTF up vs PBS |
| E330013P04Rik | NR_026942 | NR_026942 // E330013P04Rik // RIKEN cDNA E330013P04 gene // 19 D3 // 107376 /// | -2.81 | CNTF down vs PBS |
| Atf3 | NM_007498 | NM_007498 // Atf3 // activating transcription factor 3 // 1 H6|1 103.2 cM // 119 | 4.06 | CNTF up vs PBS |
| Tnpo3 | NM_177296 | NM_177296 // Tnpo3 // transportin 3 // 6 A3|6 7.2 cM // 320938 /// ENSMUST000000 | 2.46 | CNTF up vs PBS |
| Irak4 | NM_029926 | NM_029926 // Irak4 // interleukin-1 receptor-associated kinase 4 // 15 F1 // 266 | 2.52 | CNTF up vs PBS |
| Arr3 | NM_133205 | NM_133205 // Arr3 // arrestin 3, retinal // X C2 // 170735 /// ENSMUST0000011376 | -2.10 | CNTF down vs PBS |
| Gng5 | NM_010318 | NM_010318 // Gng5 // guanine nucleotide binding protein (G protein), gamma 5 // | 3.27 | CNTF up vs PBS |
| Ch25h | NM_009890 | NM_009890 // Ch25h // cholesterol 25-hydroxylase // 19 C1 // 12642 /// ENSMUST00 | 2.51 | CNTF up vs PBS |
| V1rf2 | NM_134199 | NM_134199 // V1rf2 // vomeronasal 1 receptor, F2 // 17 A3.2 // 171233 /// ENSMUS | -2.06 | CNTF down vs PBS |
| Olfr1151 | NM_146638 | NM_146638 // Olfr1151 // olfactory receptor 1151 // --- // 258631 /// ENSMUST000 | -2.17 | CNTF down vs PBS |
| Rbbp5 | NM_172517 | NM_172517 // Rbbp5 // retinoblastoma binding protein 5 // 1 E4 // 213464 /// ENS | 2.15 | CNTF up vs PBS |
| Dpp8 | NM_028906 | NM_028906 // Dpp8 // dipeptidylpeptidase 8 // 9 D // 74388 /// ENSMUST0000003496 | 2.30 | CNTF up vs PBS |
| Cobl | NM_172496 | NM_172496 // Cobl // cordon-bleu // 11 A1|11 6.5 cM // 12808 /// ENSMUST00000109 | -2.27 | CNTF down vs PBS |
| Smad2 | NM_010754 | NM_010754 // Smad2 // MAD homolog 2 (Drosophila) // 18 E3|18 48.0 cM // 17126 // | 2.44 | CNTF up vs PBS |
| Csf3r | NM_007782 | NM_007782 // Csf3r // colony stimulating factor 3 receptor (granulocyte) // 4 D2 | 2.44 | CNTF up vs PBS |
| Farsb | NM_011811 | NM_011811 // Farsb // phenylalanyl-tRNA synthetase, beta subunit // 1 C4 // 2387 | 2.34 | CNTF up vs PBS |
| Neurl3 | NM_153408 | NM_153408 // Neurl3 // neuralized homolog 3 homolog (Drosophila) // 1 B // 21485 | 2.94 | CNTF up vs PBS |
| Pcmtd1 | NM_183028 | NM_183028 // Pcmtd1 // protein-L-isoaspartate (D-aspartate) O-methyltransferase | 2.29 | CNTF up vs PBS |
| V1ra2 | NM_011684 | NM_011684 // V1ra2 // vomeronasal 1 receptor, A2 // 6 D1 // 22297 /// ENSMUST000 | -2.08 | CNTF down vs PBS |
| Golm1 | NM_027307 | NM_027307 // Golm1 // golgi membrane protein 1 // 13 B3 // 105348 /// NM_0010351 | 3.41 | CNTF up vs PBS |
| Rnf128 | NM_023270 | NM_023270 // Rnf128 // ring finger protein 128 // X F1 // 66889 /// ENSMUST00000 | 2.35 | CNTF up vs PBS |
| Trf | NM_133977 | NM_133977 // Trf // transferrin // 9 F1-F3|9 56.0 cM // 22041 /// ENSMUST0000003 | 3.38 | CNTF up vs PBS |
| Zfp330 | NM_145600 | NM_145600 // Zfp330 // zinc finger protein 330 // 8 C2 // 30932 /// ENSMUST00000 | 7.07 | CNTF up vs PBS |
| Olfr790 | NM_146933 | NM_146933 // Olfr790 // olfactory receptor 790 // --- // 258935 /// ENSMUST00000 | -2.39 | CNTF down vs PBS |
| Fam103a1 | BC091760 | BC091760 // Fam103a1 // family with sequence similarity 103, member A1 // 7 D3 / | -2.29 | CNTF down vs PBS |
| Parp12 | NM_172893 | NM_172893 // Parp12 // poly (ADP-ribose) polymerase family, member 12 // 6 B1 // | 2.45 | CNTF up vs PBS |
| Ptprb | NM_029928 | NM_029928 // Ptprb // protein tyrosine phosphatase, receptor type, B // 10 D2 // | -2.22 | CNTF down vs PBS |
| 4833439L19Rik | BC033445 | BC033445 // 4833439L19Rik // RIKEN cDNA 4833439L19 gene // 13 B1 // 97820 /// BC | 5.38 | CNTF up vs PBS |
| Abcg3 | NM_030239 | NM_030239 // Abcg3 // ATP-binding cassette, sub-family G (WHITE), member 3 // 5 | 2.03 | CNTF up vs PBS |
| Dnajb9 | NM_013760 | NM_013760 // Dnajb9 // DnaJ (Hsp40) homolog, subfamily B, member 9 // 12 B1 // 2 | 6.58 | CNTF up vs PBS |
| Mlec | NM_175403 | NM_175403 // Mlec // malectin // 5 F|5 60.0 cM // 109154 /// ENSMUST00000112121 | 2.73 | CNTF up vs PBS |
| Copb1 | NM_033370 | NM_033370 // Copb1 // coatomer protein complex, subunit beta 1 // 7 F1|7 53.3 cM | 2.57 | CNTF up vs PBS |
| Itgav | NM_008402 | NM_008402 // Itgav // integrin alpha V // 2 D|2 46.0 cM // 16410 /// ENSMUST0000 | 2.67 | CNTF up vs PBS |
| Foxn3 | BC029185 | BC029185 // Foxn3 // forkhead box N3 // 12 E // 71375 | 3.68 | CNTF up vs PBS |
| Cox6b1 | NM_025628 | NM_025628 // Cox6b1 // cytochrome c oxidase, subunit VIb polypeptide 1 // 7 B1|7 | 2.94 | CNTF up vs PBS |
| Cbr4 | NM_145595 | NM_145595 // Cbr4 // carbonyl reductase 4 // 8 B3.1 // 234309 /// ENSMUST0000003 | 2.27 | CNTF up vs PBS |
| Adap2 | NM_172133 | NM_172133 // Adap2 // ArfGAP with dual PH domains 2 // 11 B5|11 47.24 cM // 2169 | 3.52 | CNTF up vs PBS |
| Pop4 | NM_025390 | NM_025390 // Pop4 // processing of precursor 4, ribonuclease P/MRP family, (S. c | 2.24 | CNTF up vs PBS |
| Ranbp6 | NM_177721 | NM_177721 // Ranbp6 // RAN binding protein 6 // 19 C1 // 240614 /// ENSMUST00000 | 2.13 | CNTF up vs PBS |
| Parvg | NM_001162500 | NM_001162500 // Parvg // parvin, gamma // 15 E2 // 64099 /// NM_022321 // Parvg | 2.37 | CNTF up vs PBS |
| Olfr825 | NM_146677 | NM_146677 // Olfr825 // olfactory receptor 825 // --- // 258672 /// ENSMUST00000 | -2.00 | CNTF down vs PBS |
| Arl5a | NM_182994 | NM_182994 // Arl5a // ADP-ribosylation factor-like 5A // 2 C1.1 // 75423 /// ENS | 2.03 | CNTF up vs PBS |
| Olfr554 | NM_146325 | NM_146325 // Olfr554 // olfactory receptor 554 // --- // 258322 /// ENSMUST00000 | -2.55 | CNTF down vs PBS |
| Olfml3 | NM_133859 | NM_133859 // Olfml3 // olfactomedin-like 3 // 3 F2.2 // 99543 /// ENSMUST0000002 | 2.33 | CNTF up vs PBS |
| 1700045I19Rik | NR_003640 | NR_003640 // 1700045I19Rik // ring finger protein 138 pseudogene // X F5 // 7426 | -2.04 | CNTF down vs PBS |
| Olfr551 | NM_146755 | NM_146755 // Olfr551 // olfactory receptor 551 // --- // 258750 /// ENSMUST00000 | -2.00 | CNTF down vs PBS |
| Olfr355 | NM_146625 | NM_146625 // Olfr355 // olfactory receptor 355 // --- // 258618 /// ENSMUST00000 | -2.28 | CNTF down vs PBS |
| Rpl30 | NM_009083 | NM_009083 // Rpl30 // ribosomal protein L30 // 15 B3.1|15 14.7 cM // 19946 /// B | 3.73 | CNTF up vs PBS |
| Serpinb6a | NM_009254 | NM_009254 // Serpinb6a // serine (or cysteine) peptidase inhibitor, clade B, mem | 2.02 | CNTF up vs PBS |
| Zrsr1 | NM_011663 | NM_011663 // Zrsr1 // zinc finger (CCCH type), RNA binding motif and serine/argi | 2.26 | CNTF up vs PBS |
| Ly6g5c | NM_148947 | NM_148947 // Ly6g5c // lymphocyte antigen 6 complex, locus G5C // 17 B1 // 11465 | -2.07 | CNTF down vs PBS |
| Ik | NM_011879 | NM_011879 // Ik // IK cytokine // 18 B2 // 24010 /// ENSMUST00000007042 // Ik // | 2.47 | CNTF up vs PBS |
| Fam148b | NM_001081314 | NM_001081314 // Fam148b // family with sequence similarity 148, member B // 9 D | -2.20 | CNTF down vs PBS |
| Tec | NM_001113460 | NM_001113460 // Tec // tec protein tyrosine kinase // 5 C3.2|5 41.0 cM // 21682 | 4.12 | CNTF up vs PBS |
| Olfr168 | NM_146357 | NM_146357 // Olfr168 // olfactory receptor 168 // --- // 258354 /// NM_147068 // | -2.19 | CNTF down vs PBS |
| Vtn | NM_011707 | NM_011707 // Vtn // vitronectin // 11 B5|11 45.09 cM // 22370 /// ENSMUST0000001 | 3.12 | CNTF up vs PBS |
| Tipin | NM_025372 | NM_025372 // Tipin // timeless interacting protein // 9 D|9 36.0 cM // 66131 /// | 2.24 | CNTF up vs PBS |
| Ctxn1 | NM_183315 | NM_183315 // Ctxn1 // cortexin 1 // 8 A1.1|8 13.0 cM // 330695 /// ENSMUST000000 | -2.08 | CNTF down vs PBS |
| Timm9 | NM_013896 | NM_013896 // Timm9 // translocase of inner mitochondrial membrane 9 homolog (yea | -2.33 | CNTF down vs PBS |
| Uba2 | NM_016682 | NM_016682 // Uba2 // ubiquitin-like modifier activating enzyme 2 // 7 B1 // 5099 | 2.01 | CNTF up vs PBS |
| Zmym2 | NM_029498 | NM_029498 // Zmym2 // zinc finger, MYM-type 2 // 14 C3 // 76007 /// NM_017367 // | 2.00 | CNTF up vs PBS |
| AI504432 | ENSMUST00000070085 | ENSMUST00000070085 // AI504432 // expressed sequence AI504432 // 3 F2.3 // 22969 | 3.66 | CNTF up vs PBS |
| Zfand2a | NM_133349 | NM_133349 // Zfand2a // zinc finger, AN1-type domain 2A // 5 G2 // 100494 /// NM | 2.16 | CNTF up vs PBS |
| Olfr527 | NM_001011776 | NM_001011776 // Olfr527 // olfactory receptor 527 // 7 F4 // 257939 /// ENSMUST0 | -2.05 | CNTF down vs PBS |
| Fxn | NM_008044 | NM_008044 // Fxn // frataxin // 19 C1 // 14297 /// ENSMUST00000081333 // Fxn // | -2.41 | CNTF down vs PBS |
| Prei4 | NM_028802 | NM_028802 // Prei4 // preimplantation protein 4 // 2 F3|2 // 74182 /// NM_001042 | 2.02 | CNTF up vs PBS |
| Nefm | NM_008691 | NM_008691 // Nefm // neurofilament, medium polypeptide // 14 D1 // 18040 /// ENS | 2.06 | CNTF up vs PBS |
| Mobkl1b | ENSMUST00000055261 | ENSMUST00000055261 // Mobkl1b // MOB1, Mps One Binder kinase activator-like 1B ( | 2.60 | CNTF up vs PBS |
| Ubl3 | NM_011908 | NM_011908 // Ubl3 // ubiquitin-like 3 // 5 G2-3 // 24109 /// ENSMUST00000079324 | 2.72 | CNTF up vs PBS |
| Zfp60 | ENSMUST00000108336 | ENSMUST00000108336 // Zfp60 // zinc finger protein 60 // 7 A3|7 12.3 cM // 22718 | -3.60 | CNTF down vs PBS |
| Gcsh | NM_026572 | NM_026572 // Gcsh // glycine cleavage system protein H (aminomethyl carrier) // | -4.74 | CNTF down vs PBS |
| Htatip2 | NM_001146049 | NM_001146049 // Htatip2 // HIV-1 tat interactive protein 2, homolog (human) // 7 | 3.08 | CNTF up vs PBS |
| Ddx47 | NM_026360 | NM_026360 // Ddx47 // DEAD (Asp-Glu-Ala-Asp) box polypeptide 47 // 6 G1 // 67755 | 2.30 | CNTF up vs PBS |
| Tagap | NM_145968 | NM_145968 // Tagap // T-cell activation Rho GTPase-activating protein // 17 A1 / | 2.24 | CNTF up vs PBS |
| Elmod1 | NM_177769 | NM_177769 // Elmod1 // ELMO domain containing 1 // 9 A5.3 // 270162 /// ENSMUST0 | -2.21 | CNTF down vs PBS |
| Stambpl1 | NM_029682 | NM_029682 // Stambpl1 // STAM binding protein like 1 // 19 C1 // 76630 /// ENSMU | 2.22 | CNTF up vs PBS |
| Immt | NM_029673 | NM_029673 // Immt // inner membrane protein, mitochondrial // 6 C3 // 76614 /// | 2.04 | CNTF up vs PBS |
| Apod | NM_007470 | NM_007470 // Apod // apolipoprotein D // 16 B2|16 21.2 cM // 11815 /// ENSMUST00 | -2.92 | CNTF down vs PBS |
| Hist1h4d | NM_175654 | NM_175654 // Hist1h4d // histone cluster 1, H4d // --- // 319156 | 2.05 | CNTF up vs PBS |
| 2410075B13Rik | NM_146059 | NM_146059 // 2410075B13Rik // RIKEN cDNA 2410075B13 gene // 15 D3 // 223648 /// | -2.01 | CNTF down vs PBS |
| Zc3h11a | NM_144530 | NM_144530 // Zc3h11a // zinc finger CCCH type containing 11A // 1 E4 // 70579 // | 2.09 | CNTF up vs PBS |
| Olfr323 | NM_146376 | NM_146376 // Olfr323 // olfactory receptor 323 // --- // 258373 /// ENSMUST00000 | -2.09 | CNTF down vs PBS |
| Pf4 | NM_019932 | NM_019932 // Pf4 // platelet factor 4 // 5 E1 // 56744 /// ENSMUST00000031320 // | 12.50 | CNTF up vs PBS |
| Tbxas1 | NM_011539 | NM_011539 // Tbxas1 // thromboxane A synthase 1, platelet // 6 F1-pter|6 20.5 cM | 2.79 | CNTF up vs PBS |
| Palmd | NM_023245 | NM_023245 // Palmd // palmdelphin // 3 G1|3 52.0 cM // 114301 /// ENSMUST0000004 | -2.25 | CNTF down vs PBS |
| Olfr1163 | NM_146644 | NM_146644 // Olfr1163 // olfactory receptor 1163 // --- // 258638 /// ENSMUST000 | 2.10 | CNTF up vs PBS |
| Tmem126b | NM_026734 | NM_026734 // Tmem126b // transmembrane protein 126B // 7 E1 // 68472 /// ENSMUST | 2.61 | CNTF up vs PBS |
| Tmod3 | NM_016963 | NM_016963 // Tmod3 // tropomodulin 3 // 9 D|9 38.0 cM // 50875 /// ENSMUST000000 | 2.63 | CNTF up vs PBS |
| Bcl2l10 | NM_013479 | NM_013479 // Bcl2l10 // Bcl2-like 10 // 9 D // 12049 /// ENSMUST00000034709 // B | -2.17 | CNTF down vs PBS |
| Mctp1 | NM_030174 | NM_030174 // Mctp1 // multiple C2 domains, transmembrane 1 // 13 C1 // 78771 /// | 2.23 | CNTF up vs PBS |
| Ddx6 | NM_001110826 | NM_001110826 // Ddx6 // DEAD (Asp-Glu-Ala-Asp) box polypeptide 6 // 9 B|9 26.0 c | 2.95 | CNTF up vs PBS |
| Cxcl16 | NM_023158 | NM_023158 // Cxcl16 // chemokine (C-X-C motif) ligand 16 // 11 B4 // 66102 /// N | 2.92 | CNTF up vs PBS |
| P2ry13 | NM_028808 | NM_028808 // P2ry13 // purinergic receptor P2Y, G-protein coupled 13 // 3 D // 7 | 4.00 | CNTF up vs PBS |
| Pdcd6 | NM_011051 | NM_011051 // Pdcd6 // programmed cell death 6 // 13 C1 // 18570 /// ENSMUST00000 | 2.24 | CNTF up vs PBS |
| Olfr1219 | NM_146899 | NM_146899 // Olfr1219 // olfactory receptor 1219 // --- // 258901 /// ENSMUST000 | -2.51 | CNTF down vs PBS |
| Flywch2 | NM_029798 | NM_029798 // Flywch2 // FLYWCH family member 2 // 17 A3.3 // 76917 /// ENSMUST00 | -2.36 | CNTF down vs PBS |
| Sprr2d | NM_011470 | NM_011470 // Sprr2d // small proline-rich protein 2D // 3 F1|3 45.2 cM // 20758 | -2.59 | CNTF down vs PBS |
| Osbpl9 | NM_133885 | NM_133885 // Osbpl9 // oxysterol binding protein-like 9 // 4 C7 // 100273 /// NM | 2.41 | CNTF up vs PBS |
| Olfr66 | NM_013618 | NM_013618 // Olfr66 // olfactory receptor 66 // 7 E3 // 18367 /// ENSMUST0000007 | -2.03 | CNTF down vs PBS |
| Nanos2 | NM_194064 | NM_194064 // Nanos2 // nanos homolog 2 (Drosophila) // 7 A3 // 378430 /// ENSMUS | -3.03 | CNTF down vs PBS |
| Gm6725 | XM_891592 | XM_891592 // Gm6725 // predicted gene 6725 // 7 B1 // 626970 | -3.06 | CNTF down vs PBS |
| Myl4 | NM_010858 | NM_010858 // Myl4 // myosin, light polypeptide 4 // 11 E|11 65.0 cM // 17896 /// | -2.22 | CNTF down vs PBS |
| Glipr1 | NM_028608 | NM_028608 // Glipr1 // GLI pathogenesis-related 1 (glioma) // 10 D1 // 73690 /// | 3.55 | CNTF up vs PBS |
| Wbp5 | NM_011712 | NM_011712 // Wbp5 // WW domain binding protein 5 // X F1 // 22381 /// ENSMUST000 | 2.71 | CNTF up vs PBS |
| Ggta1 | NM_010283 | NM_010283 // Ggta1 // glycoprotein galactosyltransferase alpha 1, 3 // 2 B|2 25. | 2.55 | CNTF up vs PBS |
| Cdc123 | NM_133837 | NM_133837 // Cdc123 // cell division cycle 123 homolog (S. cerevisiae) // 2 A1 / | 2.28 | CNTF up vs PBS |
| Tulp4 | NM_054040 | NM_054040 // Tulp4 // tubby like protein 4 // 17 A1|17 3.2 cM // 68842 /// NM_00 | 2.09 | CNTF up vs PBS |
| Snx4 | NM_080557 | NM_080557 // Snx4 // sorting nexin 4 // 16 B3 // 69150 /// ENSMUST00000023502 // | 2.03 | CNTF up vs PBS |
| Senp3 | NM_030702 | NM_030702 // Senp3 // SUMO/sentrin specific peptidase 3 // 11 B4 // 80886 /// EN | 2.90 | CNTF up vs PBS |
| 1700020D05Rik | NM_023781 | NM_023781 // 1700020D05Rik // RIKEN cDNA 1700020D05 gene // 19 A // 75555 /// BC | -2.52 | CNTF down vs PBS |
| Sf3b1 | NM_031179 | NM_031179 // Sf3b1 // splicing factor 3b, subunit 1 // 1 C1.2|1 28.9 cM // 81898 | 2.10 | CNTF up vs PBS |
| Olfr1214 | NM_146897 | NM_146897 // Olfr1214 // olfactory receptor 1214 // --- // 258899 /// ENSMUST000 | -2.01 | CNTF down vs PBS |
| Zc3h3 | NM_172121 | NM_172121 // Zc3h3 // zinc finger CCCH type containing 3 // 15 D3 // 223642 /// | -2.14 | CNTF down vs PBS |
| Smurf2 | NM_025481 | NM_025481 // Smurf2 // SMAD specific E3 ubiquitin protein ligase 2 // 11 E1 // 6 | 2.19 | CNTF up vs PBS |
| Trhr2 | NM_133202 | NM_133202 // Trhr2 // thyrotropin releasing hormone receptor 2 // 8 E1 // 170732 | -2.05 | CNTF down vs PBS |
| Cd72 | NM_001110320 | NM_001110320 // Cd72 // CD72 antigen // 4 B1|4 22.5 cM // 12517 /// NM_007654 // | 3.36 | CNTF up vs PBS |
| Sap30 | NM_021788 | NM_021788 // Sap30 // sin3 associated polypeptide // 8 B2|8 31.0 cM // 60406 /// | 2.93 | CNTF up vs PBS |
| 2410091C18Rik | NM_028611 | NM_028611 // 2410091C18Rik // RIKEN cDNA 2410091C18 gene // 17 E3 // 73694 /// E | 2.08 | CNTF up vs PBS |
| Rnf213 | AK173199 | AK173199 // Rnf213 // ring finger protein 213 // 11 E2 // 672511 | 2.46 | CNTF up vs PBS |
| 0610007P08Rik | NM_023507 | NM_023507 // 0610007P08Rik // RIKEN cDNA 0610007P08 gene // 13 B3 // 76251 /// N | 2.22 | CNTF up vs PBS |
| Ms4a5 | NM_183190 | NM_183190 // Ms4a5 // membrane-spanning 4-domains, subfamily A, member 5 // 19 A | -2.02 | CNTF down vs PBS |
| Acbd5 | NM_001102437 | NM_001102437 // Acbd5 // acyl-Coenzyme A binding domain containing 5 // 2 A3 // | 2.07 | CNTF up vs PBS |
| Ddhd1 | NM_001042719 | NM_001042719 // Ddhd1 // DDHD domain containing 1 // 14 C1 // 114874 /// NM_1768 | 2.43 | CNTF up vs PBS |
| Tmem33 | NM_028975 | NM_028975 // Tmem33 // transmembrane protein 33 // 5 D // 67878 /// NM_030108 // | 2.37 | CNTF up vs PBS |
| Hamp | NM_032541 | NM_032541 // Hamp // hepcidin antimicrobial peptide // 7 B1|7 11.0 cM // 84506 / | -3.86 | CNTF down vs PBS |
| Lmbrd2 | NM_177178 | NM_177178 // Lmbrd2 // LMBR1 domain containing 2 // 15 A1 // 320506 /// ENSMUST0 | 2.47 | CNTF up vs PBS |
| Nupr1 | NM_019738 | NM_019738 // Nupr1 // nuclear protein 1 // --- // 56312 /// ENSMUST00000032961 / | 2.20 | CNTF up vs PBS |
| Dub1 | NM_007887 | NM_007887 // Dub1 // deubiquitinating enzyme 1 // 7 E3|7 51.5 cM // 13531 /// NM | -2.27 | CNTF down vs PBS |
| Fbxl5 | NM_001159963 | NM_001159963 // Fbxl5 // F-box and leucine-rich repeat protein 5 // 5 B3 // 2429 | 2.34 | CNTF up vs PBS |
| Nog | NM_008711 | NM_008711 // Nog // noggin // 11 C|11 50.5 cM // 18121 /// ENSMUST00000061728 // | -2.48 | CNTF down vs PBS |
| Ugt1a10 | NM_201641 | NM_201641 // Ugt1a10 // UDP glycosyltransferase 1 family, polypeptide A10 // 1 D | 2.11 | CNTF up vs PBS |
| Polr2c | NM_009090 | NM_009090 // Polr2c // polymerase (RNA) II (DNA directed) polypeptide C // 8 D1 | -2.35 | CNTF down vs PBS |
| Itgax | NM_021334 | NM_021334 // Itgax // integrin alpha X // 7 F3 // 16411 /// NM_001009949 // Mcar | 2.10 | CNTF up vs PBS |
| Atad1 | NM_026487 | NM_026487 // Atad1 // ATPase family, AAA domain containing 1 // 19 C3 // 67979 / | 2.03 | CNTF up vs PBS |
| LOC433461 | AK051942 | AK051942 // LOC433461 // hypothetical gene supported by AK051942 // 2 E3 // 4334 | -2.18 | CNTF down vs PBS |
| Fev | NM_153111 | NM_153111 // Fev // FEV (ETS oncogene family) // 1 C3 // 260298 /// ENSMUST00000 | -2.12 | CNTF down vs PBS |
| Plac8 | NM_139198 | NM_139198 // Plac8 // placenta-specific 8 // 5 E3|5 54.0 cM // 231507 /// ENSMUS | 3.05 | CNTF up vs PBS |
| Aytl1b | NM_027599 | NM_027599 // Aytl1b // acyltransferase like 1B // 5 E5 // 70902 /// ENSMUST00000 | -2.08 | CNTF down vs PBS |
| Thpo | NM_009379 | NM_009379 // Thpo // thrombopoietin // 16 B1|16 13.8 cM // 21832 /// ENSMUST0000 | -2.08 | CNTF down vs PBS |
| Nkd1 | NM_027280 | NM_027280 // Nkd1 // naked cuticle 1 homolog (Drosophila) // 8 C4 // 93960 /// E | -2.04 | CNTF down vs PBS |
| Tgs1 | NM_054089 | NM_054089 // Tgs1 // trimethylguanosine synthase homolog (S. cerevisiae) // 4 A1 | 2.81 | CNTF up vs PBS |
| Ppp1cb | NM_172707 | NM_172707 // Ppp1cb // protein phosphatase 1, catalytic subunit, beta isoform // | 3.22 | CNTF up vs PBS |
| Supt16h | NM_033618 | NM_033618 // Supt16h // suppressor of Ty 16 homolog (S. cerevisiae) // 14 C2|14 | 2.17 | CNTF up vs PBS |
| Nup98 | NM_022979 | NM_022979 // Nup98 // nucleoporin 98 // 7 E2 // 269966 /// ENSMUST00000070165 // | 4.80 | CNTF up vs PBS |
| Psmg2 | NM_134138 | NM_134138 // Psmg2 // proteasome (prosome, macropain) assembly chaperone 2 // 18 | 2.12 | CNTF up vs PBS |
| Cmc1 | NM_026442 | NM_026442 // Cmc1 // COX assembly mitochondrial protein homolog (S. cerevisiae) | 2.03 | CNTF up vs PBS |
| Dbpht2 | NM_198866 | NM_198866 // Dbpht2 // DNA binding protein with his-thr domain // 12 C3 // 38675 | -2.17 | CNTF down vs PBS |
| Ncbp2 | NM_026554 | NM_026554 // Ncbp2 // nuclear cap binding protein subunit 2 // 16 B2 // 68092 // | 2.48 | CNTF up vs PBS |
| Nkx2-2 | NM_001077632 | NM_001077632 // Nkx2-2 // NK2 transcription factor related, locus 2 (Drosophila) | -2.07 | CNTF down vs PBS |
| Eif4h | NM_033561 | NM_033561 // Eif4h // eukaryotic translation initiation factor 4H // 5 G2|5 74.0 | 2.03 | CNTF up vs PBS |
| Tiprl | NM_145513 | NM_145513 // Tiprl // TIP41, TOR signalling pathway regulator-like (S. cerevisia | 2.03 | CNTF up vs PBS |
| Sfrs5 | NM_001079695 | NM_001079695 // Sfrs5 // splicing factor, arginine/serine-rich 5 (SRp40, HRS) // | 3.60 | CNTF up vs PBS |
| Steap4 | NM_054098 | NM_054098 // Steap4 // STEAP family member 4 // 5 A1 // 117167 /// ENSMUST000001 | 3.03 | CNTF up vs PBS |
| Olfr12 | NM_206896 | NM_206896 // Olfr12 // olfactory receptor 12 // 1 D // 257890 /// ENSMUST0000008 | -3.08 | CNTF down vs PBS |
| Rsad2 | NM_021384 | NM_021384 // Rsad2 // radical S-adenosyl methionine domain containing 2 // 12 A3 | 3.20 | CNTF up vs PBS |
| Slfn5 | NM_183201 | NM_183201 // Slfn5 // schlafen 5 // 11 C // 327978 /// ENSMUST00000108158 // Slf | 5.29 | CNTF up vs PBS |
| Spcs2 | NM_025668 | NM_025668 // Spcs2 // signal peptidase complex subunit 2 homolog (S. cerevisiae) | 3.20 | CNTF up vs PBS |
| Lman1 | NM_027400 | NM_027400 // Lman1 // lectin, mannose-binding, 1 // 18 E1 // 70361 /// ENSMUST00 | 2.48 | CNTF up vs PBS |
| 4921511C20Rik | NR_003646 | NR_003646 // 4921511C20Rik // high density lipoprotein (HDL) binding protein pse | -2.20 | CNTF down vs PBS |
| Dnm1l | NM_152816 | NM_152816 // Dnm1l // dynamin 1-like // 16 A2 // 74006 /// NM_001025947 // Dnm1l | 2.03 | CNTF up vs PBS |
| Impg2 | NM_174876 | NM_174876 // Impg2 // interphotoreceptor matrix proteoglycan 2 // 16 C1.1 // 224 | 3.60 | CNTF up vs PBS |
| Olfr980 | NM_147106 | NM_147106 // Olfr980 // olfactory receptor 980 // --- // 259110 /// ENSMUST00000 | -2.84 | CNTF down vs PBS |
| Hrsp12 | NM_008287 | NM_008287 // Hrsp12 // heat-responsive protein 12 // 15 B3.3 // 15473 /// ENSMUS | 3.67 | CNTF up vs PBS |
| Polr3k | NM_025901 | NM_025901 // Polr3k // polymerase (RNA) III (DNA directed) polypeptide K // 2 H4 | 2.41 | CNTF up vs PBS |
| V1rc8 | NM_053238 | NM_053238 // V1rc8 // vomeronasal 1 receptor, C8 // 6 B3 // 113865 /// ENSMUST00 | -2.78 | CNTF down vs PBS |
| Ap3m1 | NM_018829 | NM_018829 // Ap3m1 // adaptor-related protein complex 3, mu 1 subunit // 14 A3|1 | 2.51 | CNTF up vs PBS |
| Tmem218 | NM_025464 | NM_025464 // Tmem218 // transmembrane protein 218 // 9 A4 // 66279 /// ENSMUST00 | 2.09 | CNTF up vs PBS |
| H2-T22 | NM_010397 | NM_010397 // H2-T22 // histocompatibility 2, T region locus 22 // 17 B1|17 19.74 | 4.12 | CNTF up vs PBS |
| Olfr353 | NM_146941 | NM_146941 // Olfr353 // olfactory receptor 353 // --- // 258943 /// ENSMUST00000 | -2.03 | CNTF down vs PBS |
| Stk4 | NM_021420 | NM_021420 // Stk4 // serine/threonine kinase 4 // 2 H3 // 58231 /// ENSMUST00000 | 2.10 | CNTF up vs PBS |
| 9030625A04Rik | BC116748 | BC116748 // 9030625A04Rik // RIKEN cDNA 9030625A04 gene // 14 D3 // 210808 /// N | 3.29 | CNTF up vs PBS |
| Wdr60 | NM_146039 | NM_146039 // Wdr60 // WD repeat domain 60 // 12 F2 // 217935 /// ENSMUST00000039 | -2.10 | CNTF down vs PBS |
| Chi3l4 | NM_145126 | NM_145126 // Chi3l4 // chitinase 3-like 4 // 3 F2.2 // 104183 /// NM_009892 // C | -2.17 | CNTF down vs PBS |
| Phpt1 | NM_029293 | NM_029293 // Phpt1 // phosphohistidine phosphatase 1 // 2 A3 // 75454 /// ENSMUS | 4.62 | CNTF up vs PBS |
| Olfr33 | NM_147073 | NM_147073 // Olfr33 // olfactory receptor 33 // 7 E3 // 18332 /// ENSMUST0000008 | -2.03 | CNTF down vs PBS |
| Rbbp6 | NM_175023 | NM_175023 // Rbbp6 // retinoblastoma binding protein 6 // --- // 19647 /// NM_01 | 3.43 | CNTF up vs PBS |
| Kpna4 | NM_008467 | NM_008467 // Kpna4 // karyopherin (importin) alpha 4 // 3 E2 // 16649 /// ENSMUS | 2.02 | CNTF up vs PBS |
| Fxyd5 | NM_008761 | NM_008761 // Fxyd5 // FXYD domain-containing ion transport regulator 5 // 7 B1 / | 2.75 | CNTF up vs PBS |
| Spg11 | NM_145531 | NM_145531 // Spg11 // spastic paraplegia 11 // 2 F1 // 214585 /// ENSMUST0000003 | 2.02 | CNTF up vs PBS |
| Epsti1 | NM_029495 | NM_029495 // Epsti1 // epithelial stromal interaction 1 (breast) // 14 D3 // 108 | 3.48 | CNTF up vs PBS |
| Cpne3 | NM_027769 | NM_027769 // Cpne3 // copine III // 4 A3 // 70568 /// ENSMUST00000029885 // Cpne | 2.46 | CNTF up vs PBS |
| Npm3-ps1 | NR_002702 | NR_002702 // Npm3-ps1 // nucleoplasmin 3, pseudogene 1 // 6 C3 // 108176 /// NM_ | -3.21 | CNTF down vs PBS |
| Adipor1 | NM_028320 | NM_028320 // Adipor1 // adiponectin receptor 1 // 1 E4 // 72674 /// ENSMUST00000 | 2.17 | CNTF up vs PBS |
| Rbms1 | NM_001141932 | NM_001141932 // Rbms1 // RNA binding motif, single stranded interacting protein | 2.14 | CNTF up vs PBS |
| Lsm3 | NM_026309 | NM_026309 // Lsm3 // LSM3 homolog, U6 small nuclear RNA associated (S. cerevisia | 6.34 | CNTF up vs PBS |
| Selk | NM_019979 | NM_019979 // Selk // selenoprotein K // 14 B // 80795 /// ENSMUST00000112268 // | -2.14 | CNTF down vs PBS |
| D16Ertd472e | NM_025967 | NM_025967 // D16Ertd472e // DNA segment, Chr 16, ERATO Doi 472, expressed // 16 | 3.64 | CNTF up vs PBS |
| Anxa1 | NM_010730 | NM_010730 // Anxa1 // annexin A1 // 19 B|19 18.0 cM // 16952 /// ENSMUST00000025 | 2.42 | CNTF up vs PBS |
| Timp1 | NM_001044384 | NM_001044384 // Timp1 // tissue inhibitor of metalloproteinase 1 // X A1.3|X 6.2 | 2.34 | CNTF up vs PBS |
| Olfr308 | NM_146621 | NM_146621 // Olfr308 // olfactory receptor 308 // --- // 258614 /// ENSMUST00000 | -2.16 | CNTF down vs PBS |
| Olfr54 | NM_010997 | NM_010997 // Olfr54 // olfactory receptor 54 // 11 B1.3 // 18354 /// ENSMUST0000 | -2.35 | CNTF down vs PBS |
| Polr2b | NM_153798 | NM_153798 // Polr2b // polymerase (RNA) II (DNA directed) polypeptide B // 5 C3. | 2.34 | CNTF up vs PBS |
| Isy1 | NM_133934 | NM_133934 // Isy1 // ISY1 splicing factor homolog (S. cerevisiae) // 6 D2 // 579 | 2.20 | CNTF up vs PBS |
| Apol10a | NM_177744 | NM_177744 // Apol10a // apolipoprotein L 10a // 15 E1 // 245282 /// NM_175391 // | -2.01 | CNTF down vs PBS |
| Ikzf1 | NM_001025597 | NM_001025597 // Ikzf1 // IKAROS family zinc finger 1 // 11 A1|11 6.0 cM // 22778 | 2.80 | CNTF up vs PBS |
| Olfr397 | NM_146346 | NM_146346 // Olfr397 // olfactory receptor 397 // --- // 258343 /// ENSMUST00000 | -2.08 | CNTF down vs PBS |
| Leprot | NM_175036 | NM_175036 // Leprot // leptin receptor overlapping transcript // 4 C6 // 230514 | 2.23 | CNTF up vs PBS |
| Tagap1 | NM_147155 | NM_147155 // Tagap1 // T-cell activation GTPase activating protein 1 // 17 A1 // | 13.80 | CNTF up vs PBS |
| Tmem126a | NM_025460 | NM_025460 // Tmem126a // transmembrane protein 126A // 7 E1 // 66271 /// ENSMUST | 2.55 | CNTF up vs PBS |
| Parp14 | NM_001039530 | NM_001039530 // Parp14 // poly (ADP-ribose) polymerase family, member 14 // 16 B | 2.14 | CNTF up vs PBS |
| Hist2h3c1 | NM_178216 | NM_178216 // Hist2h3c1 // histone cluster 2, H3c1 // 3 F1-F2 // 15077 /// NM_054 | -2.91 | CNTF down vs PBS |
| Rbm41 | NM_153586 | NM_153586 // Rbm41 // RNA binding motif protein 41 // X F1 // 237073 /// ENSMUST | 2.01 | CNTF up vs PBS |
